# Synthesis and Anticancer Activity of Hybrid Molecules Based on Lithocholic and (5*Z*,9*Z*)-Tetradeca-5,9-dienedioic Acids Linked via Mono(di,tri,tetra)ethylene Glycol and α,ω-Diaminoalkane Units

**DOI:** 10.3390/ph14020084

**Published:** 2021-01-23

**Authors:** Vladimir A. D’yakonov, Regina A. Tuktarova, Lilya U. Dzhemileva, Svetlana R. Ishmukhametova, Usein M. Dzhemilev

**Affiliations:** Institute of Petrochemistry and Catalysis, Russian Academy of Sciences, pr. Oktyabrya 141, 450075 Ufa, Russia; regina-tuktarova@yandex.ru (R.A.T.); IshmukhametovaSR@gmail.com (S.R.I.); dzhemilev@anrb.ru (U.M.D.)

**Keywords:** lithocholic acid, cross-cyclomagnesiation, anticancer activity, 5*Z*,9*Z*-dienoic acids, apoptosis, cell signaling

## Abstract

For the first time, hybrid molecules were synthesized on the basis of lithocholic and (5*Z*,9*Z*)-1,14-tetradeca-5,9-dienedicarboxylic acids, obtained in two stages using the homo-cyclomagnesiation reaction of 2-(hepta-5,6-diene-1-yloxy)tetrahydro-2H-pyran at the key stage. The resulting hybrid molecules containing 5*Z*,9*Z*-dienoic acids are of interest as novel synthetic biologically active precursors to create modern drugs for the treatment of human oncological diseases. The synthesized hybrid molecules were found to exhibit extremely high in vitro inhibitory activity against human topoisomerase I, which is 2–4 times higher than that of camptothecin, a known topoisomerase I inhibitor. Using flow cytometry and fluorescence microscopy, it was first shown that these new molecules are efficient apoptosis inducers in HeLa, U937, Jurkat, K562, and Hek293 cell cultures. In addition, the results of investigations into the effect of the synthesized acids on mitochondria and studies of possible DNA damage in Jurkat tumor cells are also presented.

## 1. Introduction

Despite the fact that cancer control measures around the world are rapidly developing and new low-toxic drugs differing in their mechanism of action are being created, chemotherapy remains a dangerous and difficult treatment with many side effects. One of the main problems of modern oncology is the fact that tumor cells almost always develop multidrug resistance, which leads to failures in the treatment of cancer [1,2,3]. Accordingly, the need for the development of more modern, low-toxic, and selective drugs remains an urgent task for medicinal chemistry in general.

Flora and fauna are readily available sources of structurally diverse bioactive compounds [4,5,6,7]. It is not surprising that chemical modifications have become one of the most promising trends in the search for physiologically active compounds.

Bile acids can serve as attractive objects for synthetic transformations due to their unique features, such as a chiral and rigid skeleton, as well as the presence of free acidic and hydroxyl groups [8].

Lithocholic acid (LA), one of the main bile acids found in the bile of mammals, is formed in the intestines under the influence of microflora. In addition, LA and its derivatives exhibit various biological activities. Many of these compounds are proteasome regulators [9,10], activate the vitamin D receptor, and enhance the interaction between cholecalciferols and the receptor [11,12,13], exhibit inhibitory activity toward DNA polymerases β (pol β) [14], and also have antibacterial [15] and antitumor effects [16,17,18,19,20,21,22].

Our recent studies in the search for new compounds aimed at treating oncological diseases have shown that the synthesized hybrid molecules based on steroids and cis-unsaturated acids are apoptosis inducers in Jurkat, K562, U937, HeLa tumor cell lines, as well as in a specific cell line originally derived from human embryonic kidney cells (HEK293), and can also inhibit in vitro the supercoiled DNA relaxation induced by topoisomerase I [23,24,25,26]. In the development of these studies, we prepared conjugates of LA and (5*Z*,9*Z*)-tetradeca-5,9-dienedioic acid, which are linked to each other through ethylene glycol and diaminoalkane spacers of different lengths. The antitumor activity of the synthesized compounds has also been investigated.

## 2. Results and Discussion

### 2.1. Chemistry

**Scheme 1:**
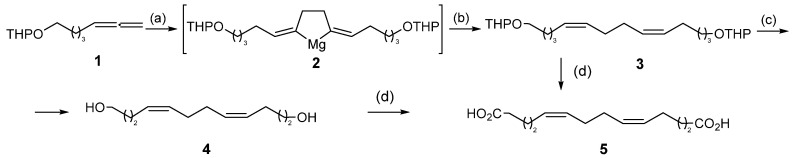
Synthesis of (5*Z*,9*Z*)-tetradeca-5,9-diene-1,14-diol 4 and (5*Z*,9*Z*)-tetradeca-5,9-dienedioic acid 5. (a) EtMgBr, Mg, Cp_2_TiCl_2_ (5 mol%), diethyl ether. (b) H_3_O^+^. (c) n-TsOH, CHCl_3_, MeOH. (d) H_2_CrO_4_/H_2_SO_4_, acetone, CH_2_Cl_2_.

The synthesis of target hybrid molecules based on lithocholic acid (LA, 6) and (5*Z*,9*Z*)-tetradeca-5,9-dienedioic acid was performed using two approaches. The first one involves the 3-*O*-acylation of acid 6 with a dicarboxylic acid. The second route represents the modification of the LA carboxyl group at the C-24 position.

(5*Z*,9*Z*)-Tetradeca-5,9-diene-1,14-diol 4 and (5*Z*,9*Z*)-tetradeca-5,9-dienedioic acid 5 were synthesized employing the previously developed homo-cyclomagnesiation reaction between tetrahydropyran ether of 5,6-hepta-5,6-dien-1-ol 1 and EtMgBr in the presence of Cp_2_TiCl_2_ (5 mol%) as the catalyst [23,24,25,26,27]. Subsequent deprotection of 1,14-bis-tetrahydropyranyl-5Z,9Z-diene-1,14-diol 3 formed after hydrolysis of magnesacyclopentane 2 in the presence of catalytic amounts of p-toluenesulfonic acid or its oxidation with Jones reagent led to compounds 4 and 5, respectively (Scheme 1).

**Scheme 2:**
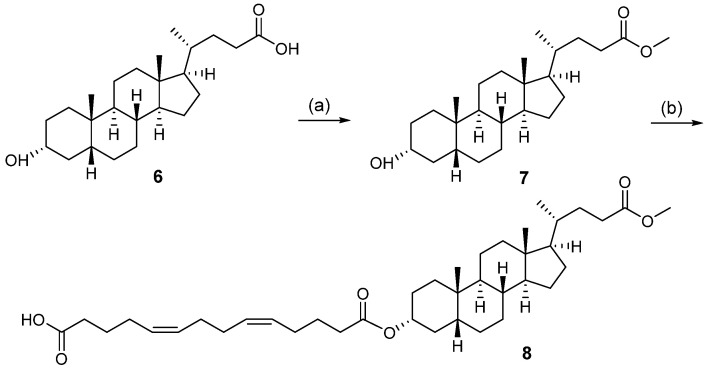
Synthesis of the LA derivative 8. (a) MeOH, AcCl, rt, 3 h. (b) (5*Z*,9*Z*)-tetradeca-5,9-dienedioic acid 5, EDCl·HCl, DMAP, CH_2_Cl_2_.

Condensation of 3-β-lithocholic acid methyl ester 7 with the corresponding fatty acid 5 was carried out by a well-known esterification procedure [23] using 1-ethyl-3-(3-dimethylaminopropyl)carbodiimide hydrochloride (EDC·HCl) in the presence of N,N-4-dimethylaminopyridine (DMAP) in CH_2_Cl_2_ to give 8 with the 82% yield (Scheme 2).

**Scheme 3:**
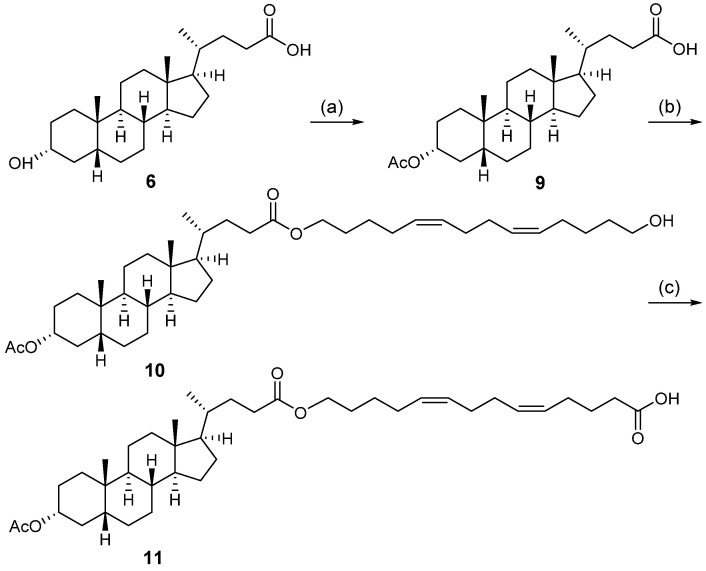
Synthesis of the LA derivative 11. (a) AcCl, pyridine, CH_2_Cl_2_, DMAP, rt, 16 h. (b) (5*Z*,9*Z*)-Тetradeca-5,9-diene-1,14-diol 4, EDCl·HCl, DMAP, CH_2_Cl_2_. (c) Jones reagent, acetone, CH_2_Cl_2_.

Scheme 3 shows the preparation of the LA derivative 11 bearing an acid fragment in the C-24 position, which includes the esterification reaction between (5*Z*,9*Z*)-tetradeca-5,9-diene-1,14-diol 4 and 3α-*O*-acetyllithocholic acid 9 and subsequent oxidation of the resulting compound 10 with Jones reagent.

In order to study the effect of ethylene glycol and diaminoalkane chains on the antitumor activity of the resulting LA derivatives, LA-fatty acid conjugates linked via ethylene glycol and diaminoalkane units of different lengths were synthesized (Scheme 4, Scheme 5). The esterification reaction of ethylene glycols with 3α-*O*-acetyllithocholic acid 9 and 3-oxocholan-24-oic acid 12 (synthesized as described in [23]), as well as the synthesis of the target (5*Z*,9*Z*)-dienoic acids of LA **15a**–**d** and **16a**–**d** occur in the presence of EDC·HCl and DMAP in CH_2_Cl_2_. Please see the Supplementary Figures S1–S88.

**Scheme 5:**
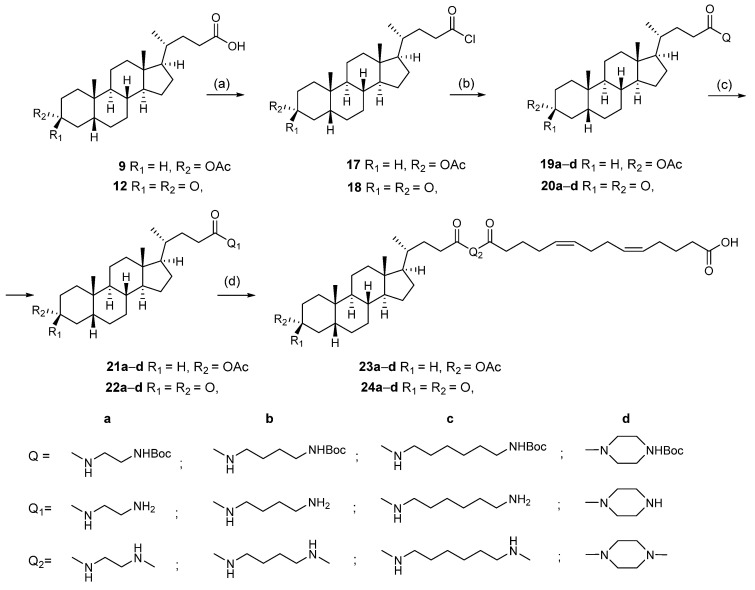
Synthesis of LA-fatty acid conjugates linked via diaminoalkane units. (a) Oxalyl chloride, CH_2_Cl_2_. (b) BOC-protected diaminoalkanes, diisopropylethylamine, CH_2_Cl_2_. (c) Trifluoroacetic acid, CHCl_3_. (d) (5*Z*,9*Z*)-Tetradeca-5,9-dienedioic acid **5**, EDCl·HCl, DMAP, CH_2_Cl_2_.

The synthesis of LA-fatty acid conjugates linked via diaminoalkane units of different lengths was carried out in several stages. Compounds **9** and **12** bearing the carboxyl group were first converted to chloroanhydrides **17** and **18** using oxalyl chloride in dichloromethane [28]. Chloroanhydrides **17** and **18** without preliminary purification were involved in reactions with diaminoalkanes to form amides **19a**–**d** and **20a**–**d**. The free terminal amino group in amides **19a**–**d** and **20a**–**d** was protected with the tert-butoxycarbonyl (BOC) group [29]. After removing the BOC protection from compounds **19a**–**d** and **20a**–**d** with trifluoroacetic acid in the chloroform, amides **21a**–**d** and **22a**–**d** were esterified with (5*Z*,9*Z*)-tetradeca-5,9-dienedioic acid **5** according to the described method [23] (Scheme 5).

### 2.2. Biological Evaluation

#### 2.2.1. Cytotoxic Activity In Vitro

In this work, for the first time, the in vitro antitumor activity for 18 synthesized compounds (**8**, **11**, **15a**–**d**, **16a**–**d**, **23a**–**d**, and **24a**–**d**) was assessed on Jurkat, K562, HEK293, HeLa, U937 cell lines and normal fibroblasts using Guava Nexin Reagent, Guava Cell Cycle, and Guava ViaCount (Millipore) reagent kits, including IC_50_ determination, cell viability studies, and cell cycle effects using flow cytofluorometry. Table 1 shows the cytotoxic activity of the synthesized compounds of hybrid molecules based on lithocholic acid against Jurkat, K562, U937, HeLa tumor cell lines, as well as a specific cell line derived from human embryonic kidneys cells (HEK293). Incubation time was 48 h. 

The results obtained in the course of studying cytotoxicity of the synthesized derivatives based on lithocholic acid **8**, **11**, **15a**–**d**, **16a**–**d**, **23a**–**d**, and **24a**–**d** presented in Table 1 showed that cell lines are, to varying degrees, sensitive to the action of the investigated hybrid molecules.

The pronounced heterogeneity of the IC_50_ values between different tumor cell lines in vitro is one of the main criteria evidencing in favor of specific antitumor activity rather than nonspecific toxicity, where the IC_50_ values similar to each other are obtained in different cell lines [30]. The original lithocholic acid 6 and camptothecin, a known inhibitor of human topoisomerase I, were taken as controls. It was experimentally shown that lithocholic acid 6 exhibits a cytotoxic effect comparable to *Camptothecium* in all the cell lines taken into the study, a significantly lower inhibition activity of the hTopI enzyme. At the same time, cytotoxicity of the synthesized hybrid molecules increases significantly and exceeds the cytotoxicity of LA by 2–100 times (Table 1). The Jurkat lymphocytic leukemia cell line was found to be the most sensitive to the synthesized compounds. Hybrid molecules **11**, **15c**,**d**, and **24c**,**d** exhibited the highest cytotoxic effect against all cancer tumor cell lines used. In addition, in a series of compounds of hybrid molecules with mono(di,tri,tetra)ethylene glycol and α,ω-diaminoalkane fragments as spacers, the pronounced increase in the cytotoxicity of molecules with increasing spacer length was observed.

For suspension (Jurkat, K562, and U937) and adhesion (HeLa, Hek293) cell lines, the index of therapeutic selectivity of cytotoxic action (SI) varies in the range of 2–10 with respect to normal fibroblasts (Table 1).

New derivatives of 5*Z*,9*Z*-dienoic acids based on lithocholic acid have been tested in vitro for the inhibitory activity of human topoisomerase I (hTopI) by studying the relaxation of supercoiled plasmid DNA under standard conditions. A known inhibitor of hTopI, camptothecin, was used as a control in all experiments. The inhibitory activity of hybrid molecules toward hTopI was shown to correlate well with their cytotoxic effect against tumor cells and varies within 0.01–2 µM (Table 1).

#### 2.2.2. Induction of Apoptosis

Based on the ability of the studied compounds to stimulate cell death at the initial stages of the investigation, it was decided to use two compound-leads (**24b** and **24c**) to study apoptosis in the Jurkat tumor cell culture.

**Figure 1:**
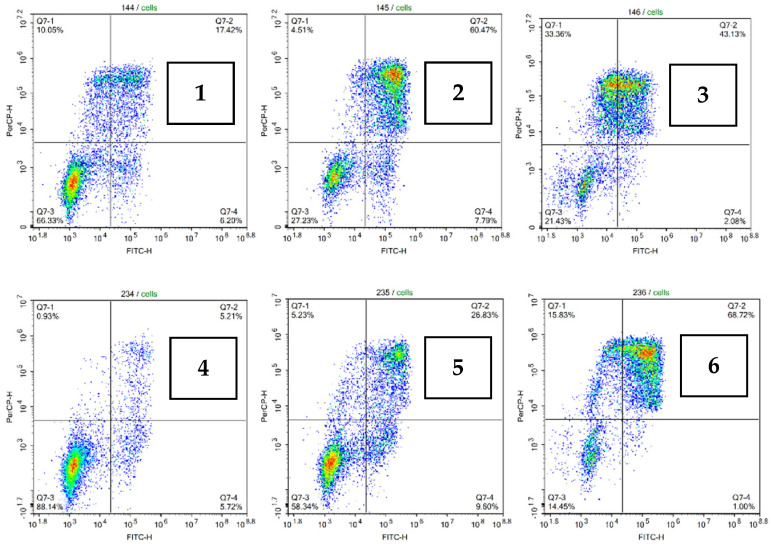
The Jurkat tumor cell line treated with various concentrations of compounds **24b** and **24c**. Cells were stained with annexin V/7-AAD and analyzed using flow cytometry. (**1**) **24c** (0.02 µМ); (**2**) **24c** (0.05 µМ); (**3**) **24c** (0.1 µМ); (**4**) **24d** (0.02 µМ); (**5**) **24d** (0.05 µМ); (**6**) **24d** (0.1 µМ). PerCP: Peridinin chlorophyll A protein; FITC: Fluorescein isothiocyanate.

The highest percentage of Jurkat cells in the early and late phases of apoptosis was observed at two concentrations of compound **24b** of 0.1 and 0.05 µM and constituted 64% and 83%, respectively (Figure 1, histograms 2 and 3). Compound 24c at a concentration of 0.1 µM was found to be somewhat less active in the induction of apoptosis. The early apoptosis was almost 68% in the Jurkat cell line (Figure 1, histogram 6), which is superior in action to camptothecin.

#### 2.2.3. Cell Cycle Analysis

**Figure 2:**
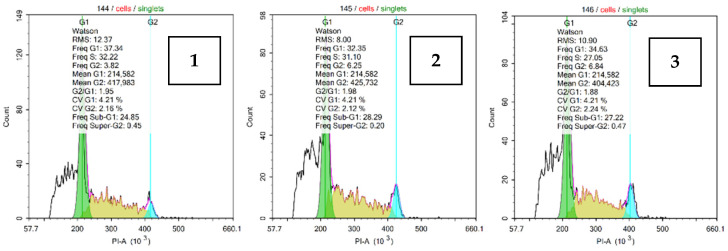
Effect of compound 24b on the cell cycle in Jurkat cells. Cells were analyzed using flow cytofluorometry. (**1**) **24c** (0.02 µМ); (**2**) **24c** (0.05 µМ); (**3**) **24c** (0.1 µМ). Data are presented as the means ± SEM calculated from results of at least four independent experiments. Statistically significant differences from the values in the control are indicated as *p* < 0.001.

Numerous studies show that DNA damage in cells induces the cell cycle arrest at the checkpoints. In particular, the cytotoxic quinoline alkaloid camptothecin, a well-known DNA-damaging agent, blocks the cell cycle in many tumor lines due to irreversible inhibition of topoisomerase I. Analysis of the cell cycle in Jurkat cells after incubation with compound 24b and the appearance of a hypodiploid DNA peak upon flow cytometry (Figure 2, histograms 1–3) indicate the inability to arrest the division cycle at checkpoints, eventually leading to cell death. In this case, the effect of compound **24b** on Jurkat cells differs from the action of camptothecin by a more pronounced blockade of the S-phase. Thus, compound **24b** induces the cell cycle arrest at the G1/S checkpoint.

#### 2.2.4. Studying the Effect on Mitochondria

**Figure 3:**
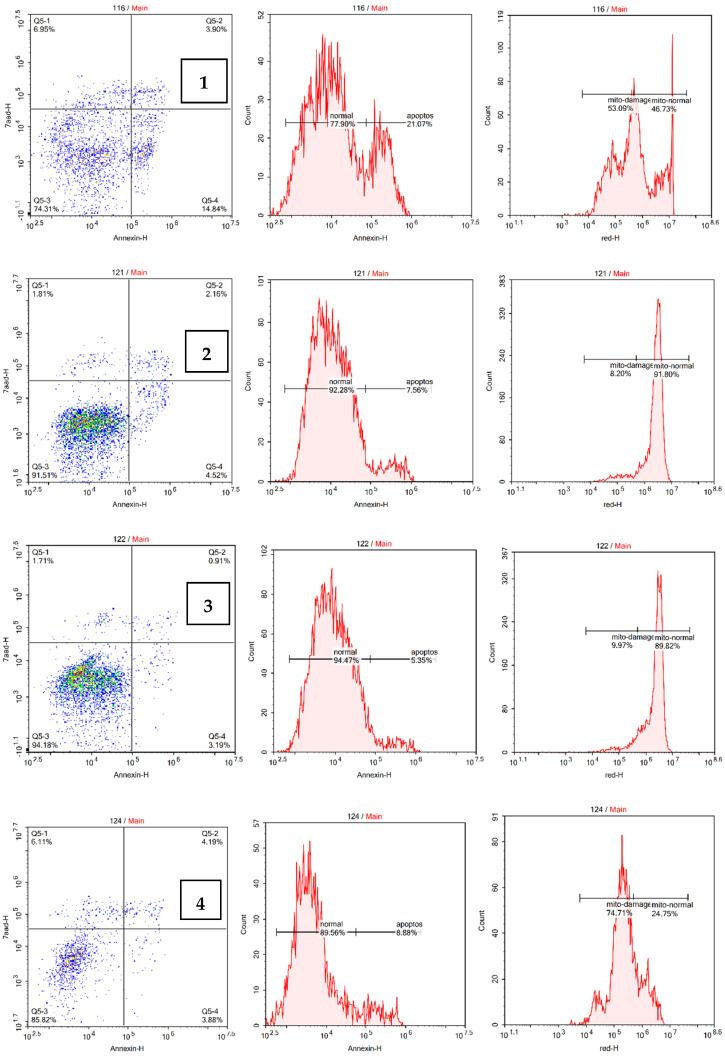

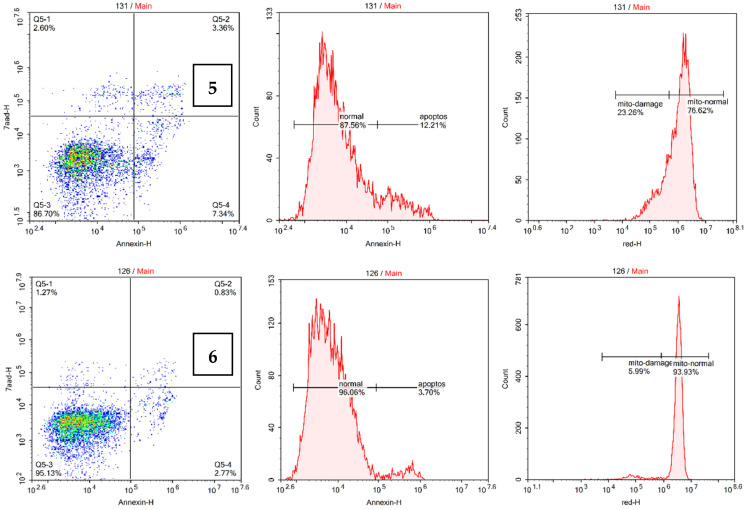
Detection of changes in mitochondrial membrane potential (ΔΨ) and apoptosis in Jurkat tumor cells treated with the studied compounds. The cells were treated with the tested compounds at their IC_50_ concentrations. Incubation time was 4 h. (**1**) LA, **6**; (**2**) **8**; (**3**) **11**; (**4**) **15d**; (**5**) **24d**; (**6**) control. Staurosporine at a concentration of 0.8 µM. The cells were stained with the following dyes: MitoSense Red, annexin V—CF488A and 7-AAD.

The next step in the analysis of biological activity of the studied compounds was the analysis of mitochondrial function, since the structure of such compounds suggests the possibility of their penetration through the mitochondrial membrane and the ability to uncouple the processes of oxidation and phosphorylation. Cells undergoing apoptosis due to mitochondrial damage exhibit depolarization of the electrochemical gradient of the inner mitochondrial membrane, mitochondrial release of apoptogenic molecules and activation of specific proteases called caspases, protrusion of cytosolic vesicles from the cell surface and loss of plasma membrane asymmetry, nuclear condensation, and finally DNA cleavage and rupture of the plasma membrane. A multivariate assessment of apoptotic markers along with detection of mitochondrial damage makes it possible to clearly determine the nature of cell death. Cell staining method with three dyes (annexin-Alexa Fluor488, 7-AAD, and MitoSense Red) allow simultaneously measuring three important parameters of a cell state, such as changes in mitochondrial potential and externalization of phosphatidylserine at the cell surface, which, during simultaneous detection, is a reliable sign of mitochondrial apoptosis. Normal cells with intact mitochondrial membrane potential exhibit high fluorescence of MitoSense Red, whereas cells in which the mitochondrial membrane potential is impaired, have much lower fluorescence parameters. Annexin V is a calcium-dependent phospholipid-binding protein with high affinity for phosphatidylserine usually localized on the inner surface of the cell membrane. Control cells without apoptosis contain phosphatidylserine on the inner surface of their membrane, while apoptotic cells show positive green fluorescence due to externalization of phosphatidylserine. DNA intercalator 7-aminoactinomycin (7-AAD), which does not penetrate a living cell, appears inside the cell only at the late stages of apoptosis upon membrane damage, commonly observed at late cellular apoptosis and necrosis. Thus, with identical colors during the cytometric analysis, we observed several cell populations: (1) living cells with intact mitochondrial membrane; (2) cells with diffusion membrane potential, but without annexin V or 7-AAD staining; (3) early apoptotic cells with diffusion membrane potential and annexin V binding; (4) late apoptotic cells or dead cells; (5) cells with diffusion membrane potential. We analyzed activity of the synthesized derivatives of lithocholic acid in relation to the mitochondria of Jurkat tumor cells. The cells were incubated with the tested compounds for 4 h (Figure 3).

To reveal the ability of the synthesized lithocholic acid derivatives to penetrate the mitochondrial membrane and uncouple oxidation and phosphorylation thus causing intramitochondrial stress and initiate mitochondrial apoptosis, compound-leads were selected not only in terms of cytotoxic activity, but also in terms of pronounced changes in the kinetics of the cell cycle from various series of hybrid molecules. Compounds **6**, **8**, **11**, **15d**, and **24b** have been analyzed in detail (Figure 3). All of the above derivatives of lithocholic acid exhibit the pronounced apoptosis-inducing activity against Jurkat cell lines, and their effect is comparable to classical mitochondrial apoptosis-inducing agents such as staurosporine. In this sense, compound **15d** exhibited the highest activity with respect to mitochondrial potential. This compound had an uncoupling effect even to a slightly greater extent than staurosporine, if we pay attention to the percentage of cells with damaged mitochondria. In this regard, when exposed to **15d**, we observed mitochondrial damage in 74.71% of the cells, whereas when exposed to staurosporine, the percentage of such cells was 22.83%. According to our data, lithocholic acid 6 itself belongs to compound-leads in terms of the effect on mitochondria. The percentage of cells with damaged mitochondria after treatment with lithocholic acid at a concentration of IC_50_ was 53.09%. Other compounds, **8**, **11**, and **24b**, also reduce ΔΨ, and the effect of these compounds, quite obviously, is a more extended process in time. For instance, after incubation of cells with the tested compounds for 4 h, compounds **8**, **11**, and **24b** demonstrated the percentage of mitochondrial damage of 8.20%, 9.97%, and 22.26%, respectively. Possibly, compounds **8**, **11**, and **24b** penetrate the mitochondrial membrane somewhat worse or the process of uncoupling proceeds more slowly, since these compounds can be the so-called “soft” uncouplers. Such compounds show themselves as substances that uncouple oxidation and phosphorylation on the inner mitochondrial membrane by reducing the mitochondrial potential ΔΨ. According to Mitchell’s chemiosmotic theory, the factor that couples oxidation with phosphorylation is the electrochemical proton potential ∆μH^+^ which appears on the inner mitochondrial membrane during electron transport. Proton gradient creating the difference in chemical and electrical potentials, is the source of energy required for the reaction of ATP formation [31]. Mild uncoupling is a reversible decrease in the membrane potential of mitochondria, in which there is no significant/physiologically significant suppression of respiration, damage to the components of the respiratory chain, or disruption of the physicochemical properties of mitochondrial membranes [32].

#### 2.2.5. Cytochrome C Release from Mitochondria

Cytochrome C is a soluble heme protein localized in the inner space of mitochondria which plays an irreplaceable role as an electron carrier in oxidative phosphorylation during respiration, in the transfer of electrons from the cytochrome bc1 complex to cytochrome oxidase on the inner mitochondrial membrane. During the induction of apoptosis and related events such as mitochondrial depolarization, cardiolipin peroxidation, cytochrome c, together with other pro-apoptotic proteins, namely, proCaspase-9, Smac/DIABLO, APF-1, and Endo F, is released into the cytosol, signaling the initiation of the intrinsic apoptosis pathway. When released, cytochrome c activates a caspase cascade thus promoting cell death. Quantitative and qualitative assessment of cytochrome c release from mitochondria can be used to characterize the mitochondrial pathway of apoptosis. 

**Figure 4:**
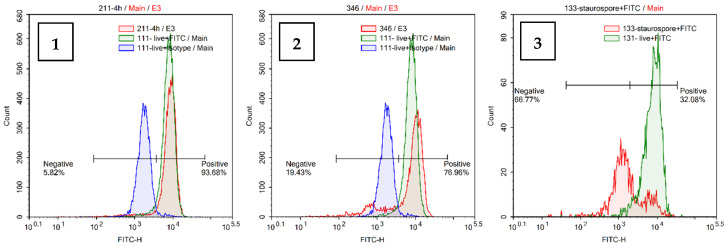
Histograms depicting cells stained using the FlowCellect Cytochrome C Kit. (**1**) shows untreated Jurkat cells; (**2**) shows cells treated with compound **24d**; (**3**) shows cells induced with staurosporine (1 μM) for 2 h. The graphs show the downward fluorescence shifts of cytochrome c levels for several cell lines and inducers for the FlowCellect Cytochrome c Kit. Histogram plots show live cells (negative control) and cells stained with cytochrome c-FITC. Cells stained with cytochrome c-FITC demonstrate high fluorescence. They are cells with intact mitochondria. Cells which exhibit reduced fluorescence undergo membrane disruption by the test compound, resulting in a loss of cytochrome C.

The process of the cytochrome C loss after treatment with the tested compound **24d** as compared to staurosporine was analyzed in Jurkat cells (Figure 4). Compound **24d** caused a loss of cytochrome C.

#### 2.2.6. Interaction with Histones and Topoisomerase II

For assessing the influence on topoisomerase II of the studied derivatives of hybrid molecules based on 5*Z*,9*Z*-diene acids, the investigation into possible DNA damage in Jurkat tumor cells was carried out. We had previously shown that various compounds containing a diene moiety can affect their ability to inhibit topoisomerase II [7,23,24,25,26,27,33,34,35,36,37,38]. Changes in the concentration of total and phosphorylated proteins H2A.X allow at the same time confirming the target specificity of the phosphorylation event of this histone class. Simultaneous detection of unphosphorylated and phosphorylated H2A. X protein is a sensitive tool for studying the effect of compounds on the genetic apparatus of a cell and allows one to investigate the relationship between DNA damage and the initiation of apoptosis. Many chemotherapeutic agents, such as etoposide, damage tumor cells by stimulating DNA double-strand breaks. Furthermore, the γ-H2A.X level, detected by flow cytometry, was shown to correlate with the amount of DNA double-strand breaks and tumor cell death [39].

**Figure 5:**
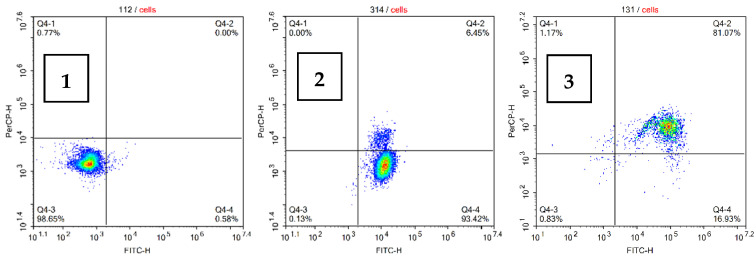
Detection of the phosphorylated H2A.X accumulation in the Jurkat cells exposed to compound **24d** compared with etoposide. Incubation with studied compounds was carried out for 2 h. Control (live cells, unstained) (**1**); cells treated with compound **24d** at its IC_50_ concentration (**2**); cells treated with etoposide at a concentration of 0.2 µM/mL (**3**); Q4-2—cells containing phosphorylated histones H2A.X (DNA damage marker—staining with phosphor-specific antibodies Anti-phospho-Histone H2A.x (Ser139)–PerCP); Q4-3—cells without staining (live cells); Q4-4—cells containing non-phosphorylated histones H2A.X (Anti-Histone H2A.X-FITC).

After stimulation of Jurkat cells with etoposide for 2 h, a pronounced increase in the phosphorylated fraction of histone H2A.X was observed (Figure 5). In the experiment, Jurkat cells were treated with compound **24d**, and it was shown that this compound affects the phosphorylation process of H2A.X insignificantly, virtually not increasing the population of cells with phosphorylated protein (Figure 5) compared with etoposide as the reference compound. When cells were exposed to etoposide, the phosphorylated fraction of the H2A.X protein was determined in all the cells (almost 99%), whereas upon exposure to compound **24d**, only 6.45% of the cells contained phosphorylated protein H2A.X. Therefore, we can declare that compound 24d virtually does not initiate accumulation of DNA strand breaks in a cell with subsequent apoptosis activation via the p53-dependent mechanism.

## 3. Materials and Methods

### 3.1. Chemistry

Lithocholic acid, 4-dimethylaminopyridine (DMAP), N-[3-(methylamino)propyl]-N’-ethylcarbodiimide hydrochloride (EDC·HCl) were obtained from Sigma-Aldrich and Acros Organics. Dichloromethane was freshly distilled before use. Optical rotations were measured on a PerkinElmer 341 polarimeter. Melting points were recorded on Stuart SMP3. IR spectra were recorded on Bruker VERTEX 70V using KBr discs over the range of 400–4000 cm^−1^. ^1^H and ^13^C NMR spectra were obtained using a Bruker Ascend 500 spectrometer in CDCl_3_ operating at 500 MHz for ^1^H and 125 MHz for ^13^C and a Bruker AVANCE 400 spectrometer in CDCl_3_ operating at 400 MHz for ^1^H and 100 MHz for ^13^C. Mass spectra of MALDI TOF (Matrix-Assisted Laser Desorption/Ionization Time-of-Flight Mass Spectrometry)/TOF positive ions (matrix of the sinapic acid) are recorded on a mass spectrometer Bruker Autoflex^TM^ III Smartbeam. Elemental analyses were measured on a 1106 Carlo Erba apparatus. Individuality and purity of the synthesized compounds were controlled using TLC on Sorbfil plates; anisic aldehyde in acetic acid was used as a developer. Column chromatography was carried out on the Acrus silica gel (0.060–0.200 mm).

### 3.2. Cell Culturing

Cell culturing was carried out following the known procedure [40]. Cells (Jurkat, K562, U937, HeLa, HEK293, and normal fibroblasts) were purchased from The Health Protection Agency (HPA) Culture Collections (HPA, Salisbury, UK) and cultured according to the standard mammalian tissue culture protocols and sterile technique. All cell lines used in the study were tested and shown to be free of mycoplasma and other viral contamination. HeLa and HEK293 cell lines were cultured as monolayers and maintained in Dulbecco’s modified Eagle’s medium (DMEM, Gibco BRL, Waltham, MA, USA) supplemented with 10% fetal bovine serum and 1% penicillin–streptomycin solution at 37 °C in a humidified incubator under a 5% CO_2_ atmosphere. The cells were maintained in RPMI 1640 (Jurkat, K562, U937) (Gibco, Waltham, MA, USA) supplemented with 4 mM glutamine, 10% FBS (Sigma, Burlington, MA, USA), and 100 units/mL penicillin–streptomycin (Sigma, Burlington, MA, USA). All types of cells were grown in an atmosphere of 5% CO_2_ at 37 °C. The cells were subcultured at intervals of 2–3 days. Adherent cells (HeLa, HEK293, and normal fibroblasts) were suspended using trypsin/EDTA and counted after they had reached 80% confluency. The cells were then seeded in 24-well plates at 5 × 10^4^ cells per well and incubated overnight. Jurkat, K562, U937 cells were subcultured at 2-day intervals with a seeding density of 1 × 10^5^ cells per well in 24-well plates in RPMI with 10% FBS.

### 3.3. DNA Topoisomerase I Assay

Inhibition of DNA topoisomerase I tests were carried out following the known procedure [40]. The inhibitory activity and the mechanism of inhibition of (5*Z*,9*Z*)-5,9-dienoic acids were determined using the Topoisomerase I Drug Screening Kit TG-1018-2, (Topogen, Buena Vista, CO, USA) (the tested compound was added before topoisomerase I). The relaxation of supercoiled DNA under the action of topoisomerase I was carried out as follows: the reaction mixture (20 µL) containing 0.25 µg of the DNA plasmid pHOT (TopoGen, Buena Vista, CO, USA), 1 unit of recombinant topoisomerase I (TopoGen, USA), and the tested compound was incubated in the buffer (35mM Tris-HCl, рН 8.0; 72 mM KСl, 5 mM MgCl, 5 mM dithiothreitol, 5 mM spermidine, and 0.01% bovine serum albumin) for 30 min at 37 °С using a Biosan thermostat (Riga, Latvia). The tested compound was introduced in the reaction mixture prior to the addition of the enzyme topoisomerase I. The inhibiting action on topoisomerase I was monitored using the alkaloid camptothecin (TopoGEN, Buena Vista, CO, USA). The reaction was terminated by adding sodium dodecyl sulfate up to a concentration of 1%. After addition of a solution (5 mg/mL) of proteinase K (Sigma Chemical Co., St. Louis, MO, USA) (1:10), the reaction mixture was incubated for 40 min at 37 °С. A 0.1% solution of bromophenol blue (1:10) was added and the samples were electrophoresed in the absence of ethidium bromide. The reaction products were separated in a 1% agarose gel (3 V/cm) for 4–6 h. After the electrophoresis, the gels were treated with a solution of ethidium bromide (0.5 µg/mL). The gels were visualized in the UV light in a Infinity VX2 1120/Blue X-Press gel documentation system (Vilber Lourmat, Paris, France). The possible action of the tested compounds on supercoiled DNA was checked by performing the reaction without topo I, the tested compounds being added in the same concentrations as in the reaction with the enzyme.

### 3.4. Cytotoxicity Assay

Cytotoxicity tests were carried out following a known procedure [40]. Viability (live/dead) assessment was performed by staining cells with 7-AAD (7-aminoactinomycin D) (Biolegend, San Diego, CA, USA). The cells were seeded in a 24-well plate (1 × 10^5^ cells/well). After treatment (24 h), the cells were harvested, washed 1–2 times with phosphate-buffered saline (PBS), and centrifuged at 400 g for 5 min. Cell pellets were resuspended in 200 mL of a flow cytometry staining buffer (PBS without Ca^2+^ and Mg^2+^, 2.5% FBS) and stained with 5 μL of a 7-AAD staining solution for 15 min at room temperature in the dark. Samples were acquired on a NovoCyteTM 2000 FlowCytometry System (ACEA, San Diego, CA, USA) equipped with a 488 nm argon laser. Detection of 7-AAD emission was done using a 675/30 nm filter in the FL4 channel.

### 3.5. Viability and Apoptosis

Induction of apoptosis tests was carried out following a known procedure [40]. Apoptosis was determined by flow cytometric analysis of annexin V and 7-aminoactinomycin D staining. After treatment (24 h), the cells were harvested, washed 1–2 times with phosphate-buffered saline (PBS), and centrifuged at 400 g for 5 min. Cell pellets were resuspended in 200 μL of a flow cytometry staining buffer (PBS without Ca^2+^and Mg^2+^, 2.5% FBS). Then, 200 μL of the Guava Nexin reagent (Millipore, Bedford, MA, USA) was added to 5 × 10^5^ cells in 200 μL, and the cells were incubated with the reagent for 20 min at room temperature in the dark. At the end of incubation, the cells were analyzed on a NovoCyteTM2000 FlowCytometry System (ACEA, San Diego, CA, USA).

### 3.6. Cell Cycle Analysis

Cell cycle analysis was carried out following a known procedure [40]. Cell cycle was analyzed using the method of propidium iodide staining. After treatment (24 h), the cells were harvested, washed 1–2 times with phosphate-buffered saline (PBS), and centrifuged at 400 g for 5 min. Cell pellets were resuspended in 200 μL of a flow cytometry staining buffer (PBS without Ca^2+^ and Mg^2+^, 2.5% FBS). Then, the cells were plated in 24-well round bottom plates at a density of 10 × 10^5^ cells per well, centrifuged at 450 g for 5 min, and fixed with ice-cold 70% ethanol for 24 h at 0 °C. The cells were then washed with PBS and incubated with 250 μL of the Guava Cell Cycle Reagent (Millipore, Burlington, MA, USA) for 30 min at room temperature in the dark. The samples were analyzed on a NovoCyteTM 2000FlowCytometry System (ACEA, San Diego, CA, USA).

### 3.7. Mitochondrial Damage

Mitochondrial damage tests were carried out following a known procedure [40]. In our work, we performed a cytometric assay which allowed multiparametric evaluation of three cell health markers: change in mitochondrial potential (early apoptosis and cellular stress), phosphatidylserine expression on the cell surface (late apoptosis), and membrane permeabilization (cell death). Millipore’s FlowCellect™ MitoDamage Kit’s use of the reagents allows researchers to obtain information on early and late apoptosis in one simple assay. The cells were treated with testing compounds at various concentrations and incubated (37 °C, 5% CO_2_) for 4 h. After this time, the cells were dissociated with the Accutase solution, stained, and analyzed by flow cytometry (NovoCyte Flow Cytometry ™, Burlington, MA, USA) according to the manufacturer’s protocols (FlowCellect™ MitoDamage Kit, Merck, and FlowCellect™ Oxidative Stress Characterization Kit, Merck, Burlington, MA, USA).

### 3.8. Histone H2A.X Analysis

Histone H2A.X analysis was carried out following a known procedure [40]. Cell preparation for phosphorylation of Histone H2A.X in the Jurkat cells analysis was grown in a culture flask for 48 h. The medium was changed at 24 h before drug treatment. Phosphorylation of histone H2A.X was measured with the FlowCellect™ DNA Damage Histone H2A.X Dual Detection Kit (FCCS025153, Millipore, MA, USA). Briefly, isolated and treated cells were counted and diluted with an assay buffer (5 × 10^6^ cells/2.5 mL). Paraformaldehyde (PFA, Sigma, Burlington, MA, USA) was added at a final concentration of 2% and the cells were fixed at 37 °C for 10 min. The cells were then immediately chilled on ice, centrifuged, washed three times with 0.5% bovine serum albumin (BSA; EMD Bioscience, La Jolla, CA, USA)–PBS, permeabilized with 70% ethanol in distilled water or 90% methanol in 0.5% BSA–PBS, and kept at 4 °C for ethanol samples and at −20 °C for methanol samples until further staining. After that, the cells were fixed, permeabilized, and incubated with Anti-phospho-Histone H2A.X (Ser139, Alexa Fluor^®^555, part number CS208203, Millipore, Billerica, MA, USA) and anti-H2A.X (PECy5, part number CS209202, Millipore, Billerica, MA, USA), and then analyzed by flow cytometry.

### 3.9. Chemical Experimental Data

^1^H NMR (CDCl_3_, 400 MHz) and ^13^C NMR (CDCl_3_, 100 MHz) spectral data and synthesis method for 2,2’-[(5*Z*,9*Z*)-tetradeca-5,9-diene-1,14-diylbis(oxy)]bistetrahydro-2H-pyran 3 and (5Z,9Z)-tetradeca-5.9-dienedioic acid 5 are described in the literature [23].

#### 3.9.1. General Procedure for the Synthesis of (5*Z*,9*Z*)-Tetradeca-5,9-diene-1,14-diol (**4**)

To a mixture of methanol (10 mL), CHCl_3_ (10 mL), and 1,14-tetrahydropyranyl-5Z,9Z-dien-1,14-diol 3 (0.39 g, 1.0 mmol), catalytic amounts of p-toluenesulfonic acid (p-TSA) were added, and the reaction mixture was stirred at 60 °C for 4 h. After this, the solvent was evaporated, and then the organic extract was washed with a saturated solution of sodium bicarbonate (3 × 25 mL). The organic layer was dried over MgSO_4_ and concentrated in vacuo. The residue was purified by column chromatography using hexane/ethyl acetate (3:1) as the elution solvent to afford 0.52 g (76% yield) of diol 4.

(5*Z*,9*Z*)-Tetradeca-5,9-diene-1,14-diol (**4**)

Colorless oil, 0.17 g, 76% yield. IR (KBr) ν_max_ 3006, 2924, 2852, 1657, 1466, 1408, 1384, 1214, 1165, 1100, 1034, 965, 911, 861, 755, 723, 665 cm^−1^; ^1^H NMR (CDCl_3_, 500 MHz) δ 5.42–5.34 (4H, m, H-5, H-6, H-9, H-10), 3.57 (4H, t, J = 6.5 Hz, H-1, H-14), 2.07–2.01 (8H, m, H-4, H-7, H-8, H-11), 1.58–1.52 (4H, m, H-2, H-13), 1.43–1.36 (4H, m, H-3, H-12); ^13^C NMR (CDCl_3_, 125 MHz) δ 129.9 (C-6, C-9), 129.5 (C-5, C-10), 62.6 (C-1, C-14), 32.3 (C-2, C-13), 27.4 (C-7, C-8), 26.9 (C-4, C-11), 25.8 (C-3, C-12).

#### 3.9.2. Reaction of Methyl Ester of Lithocholic Acid (**7**) with (5*Z*,9*Z*)-Tetradeca-5.9-dienedioic Acid (**5**)

Lithocholic acid derivative **8** was synthesized according to a modified literature procedure [26]. To a solution of methyl ester of lithocholic acid (0.39 g, 1.0 mmol) in CH_2_Cl_2_ (50 mL), (5*Z*,9*Z*)-tetradeca-5.9-dienedioic acid 4 (0.51 g, 2.0 mmol) was added, followed by EDC·HCl (0.48 g, 2.5 mmol) and DMAP (18 mg, 0.15 mmol) under argon. The mixture was stirred at room temperature for 12 h until the reaction was complete (TLC monitoring, hexane/ethyl acetate). The mixture was diluted with H_2_O (10 mL) and the CH_2_Cl_2_ layer was separated, dried over MgSO_4_, and concentrated. The crude product was purified by column chromatography (silica gel) using hexane/ethyl acetate (5:1) as the elution solvent to afford compound **8**.

(5*Z*,9*Z*)-14-{[(3α,5β)-24-Methoxy-24-oxocholan-3-yl]oxy}-14-oxotetradeca-5,9-dienoic acid (**8**)

Colorless waxy solid, 0.42 g, 67% yield. [α]_D_^21^ + 30.3 (c 0.36, CHCl_3_); IR (KBr) ν_max_ 2929, 2867, 1735, 1709, 1449, 1384, 1326, 1307, 1243, 1165, 1100, 1022, 802, 722 cm^−1^; ^1^H NMR (CDCl_3_, 500 MHz) δ 5.43–5.35 (4H, m, H-5’, H-6’, H-9’, H-10’), 4.79–4.71 (1H, m, H-3), 3.68 (3H, s, O-CH_3_), 2.37 (2H, t, J = 7.5 Hz, H-2’), 2.35‒1.02 (28H, m), 2.29 (2H, t, J = 7.5 Hz, H-13’), 2.12–2.06 (8H, m, H-4’, H-7’, H-8’, H-11’), 1.73–1.67 (4H, m, H-3’, H-12’), 0.94 (3H, s, H-19), 0.92 (3H, d, J = 6.4 Hz, H-21), 0.66 (3H, s, H-18); ^13^C NMR (CDCl_3_, 125 MHz) δ 178.8 (C-1’), 174.9 (C-24), 173.5 (C-14’), 130.4, 130.1 (C-6’, C-9’), 129.1, 128.8 (C-5’, C-10’), 74.3 (C-3), 56.5 (C-14), 55.9 (C-17), 51.5 (O-CH_3_), 42.7 (C-13), 41.9 (C-5), 40.4 (C-9), 40.1 (C-12), 35.8 (C-8), 35.4 (C-20), 35.0 (C-1), 34.6 (C-10), 34.2 (C-13’), 33.3 (С-2’), 32.3 (C-4), 31.1 (C-23), 31.0 (C-22), 28.2 (C-16), 27.4, 27.3 (C-7’, C-8’), 27.0 (C-6), 26.7 (C-2), 26.6 (C-11’), 26.5 (C-4’), 26.3 (C-7), 25.0 (C-12’), 24.6 (C-3’), 24.2 (C-15), 23.3 (C-19), 20.8 (C-11), 18.3 (C-21), 12.0 (C-18); anal. calcd for C_39_H_62_O_6_: C, 74.72; H, 9.97; found C, 74.67; H, 9.92.

#### 3.9.3. Reaction of 3α-Acetoxy-5β-cholan-24-oic Acid with (5Z,9Z)-Tetradeca-5,9-diene-1,14-diol (**4**)

Lithocholic acid derivative **10** was synthesized according to a modified literature procedure [38]. To a solution of 3α-acetoxy-5β-cholan-24-oic acid (0.42 g, 1.0 mmol) in CH_2_Cl_2_ (50 mL), (5Z,9Z)-tetradeca-5,9-diene-1,14-diol (0.46 g, 2.0 mmol) was added, followed by EDC·HCl (0.48 g, 2.5 mmol) and DMAP (18 mg, 0.15 mmol) under argon. The mixture was stirred at room temperature for 12 h until the reaction was complete (TLC monitoring, hexane/ethyl acetate). The mixture was diluted with H_2_O (10 mL) and the CH_2_Cl_2_ layer was separated, dried over MgSO_4_, and concentrated. The crude product was purified by column chromatography (silica gel) using hexane/ethyl acetate (5:1) as the elution solvent to afford compound **10**.

(5*Z*,9*Z*)-14-Hydroxytetradeca-5,9-dien-1-yl (3α,5β)-3-(acetyloxy)cholan-24-oate (**10**)

Colorless waxy solid, 0.44 g, 71% yield. [α]_D_^19^ + 25.0 (c 0.94, CHCl_3_); IR (KBr) ν_max_ 2933, 2865, 1736, 1451, 1382, 1363, 1242, 1166, 1028, 911, 811, 756 cm^−1^; ^1^H NMR (CDCl_3_, 500 MHz) δ 5.40–5.31 (4H, m, H-5’, H-6’, H-9’, H-10’), 4.73–4.66 (1H, m, H-3), 4.05 (2H, t, J = 6.8 Hz, H-1’), 3.62 (2H, t, J = 6.4 Hz, H-14’), 2.35‒1.00 (28H, m), 2.11–2.03 (8H, m, H-4’, H-7’, H-8’, H-11’), 2.01 (3H, s, COCH_3_), 1.68–1.65 (2H, m, H-2’), 1.57–1.51 (2H, m, H-13’), 1.44–1.36 (4H, m, H-3’, H-12’), 0.92 (3H, s, H-19), 0.90 (3H, d, J = 6.4 Hz, H-21), 0.63 (3H, s, H-18); ^13^C NMR (CDCl_3_, 125 MHz) δ 174.4 (C-24), 170.6 (COCH_3_), 129.9, 129.7, 129.6, 129.4 (C-5’, C-6’, C-9’, C-10’), 74.4 (C-3), 64.2 (C-1’), 62.7 (C-14’), 56.5 (C-14), 55.9 (C-17), 42.7 (C-13), 41.9 (C-5), 40.4 (C-9), 40.1 (C-12), 35.8 (C-8), 35.3 (C-20), 35.0 (C-1), 34.6 (C-10), 32.4 (C-13’), 32.2 (C-4), 31.3 (C-23), 31.0 (C-22), 28.2 (C-2’, C-16), 27.4, 27.3 (C-7’, C-8’), 27.0 (C-2), 26.9, 26.8 (C-4’, C-11’), 26.6 (C-6), 26.3 (C-7), 25.9 (C-3’), 25.8 (C-12’), 24.2 (C-15), 23.3 (C-19), 21.4 (COCH_3_), 20.8 (C-11), 18.3 (C-21), 12.0 (C-18); anal. calcd for C_40_H_66_O_5_: C, 76.63; H, 10.61; found C, 76.51; H, 10.53.

#### 3.9.4. Oxidation of the Esterification Product 10 with Jones Reagent

To a solution of compound **10** (0.44 g, 0.7 mmol) in acetone (25 mL) and CH_2_Cl_2_ (5 mL) at room temperature, Jones reagent (0.75 mL) was added dropwise. The reaction mixture was stirred at room temperature for 0.5 h, then quenched with water (10 mL), concentrated under reduced pressure to remove the excess of acetone and CH_2_Cl_2_, and the aqueous layer was extracted with ethyl acetate (3 × 20 mL). The organic layer was dried over MgSO_4_ and concentrated in vacuo. The residue was purified by column chromatography using hexane/ethyl acetate (3:1) as the elution solvent to afford dienoic acid **11**.

(5*Z*,9*Z*)-14-{[(3α,5β)-3-(Acetyloxy)-24-oxocholan-24-yl]oxy}tetradeca-5,9-dienoic Acid (**11**)

Colorless waxy solid, 0.37 g, 83% yield. [α]_D_^23^ + 26.4 (c 0.66, CHCl_3_); IR (KBr) ν_max_ 2931, 2866, 1736, 1709, 1450, 1382, 1363, 1242, 1167, 1096, 1027, 980, 803, 726 cm^−1^; ^1^H NMR (CDCl_3_, 500 MHz) δ 5.44–5.34 (4H, m, H-5’, H-6’, H-9’, H-10’), 4.76–4.70 (1H, m, H-3), 4.07 (2H, t, J = 6.4 Hz, H-14’), 2.39–2.33 (2H, m, H-2’), 2.35‒1.02 (28H, m), 2.13–2.05 (8H, m, H-4’, H-7’, H-8’, H-11’), 2.04 (3H, s, COCH_3_), 1.73–1.67 (2H, m, H-3’), 1.66–1.61 (2H, m, H-13’), 1.47–1.41 (2H, m, H-12’), 0.93 (3H, s, H-19), 0.92 (3H, d, J = 6.4 Hz, H-21), 0.65 (3H, s, H-18); ^13^C NMR (CDCl_3_, 125 MHz) δ 179.2 (C-1’), 174.5 (C-24), 170.8 (COCH_3_), 130.4, 129.7 (C-6’, C-9’), 129.6, 128.8 (C-5’, C-10’), 74.4 (C-3), 64.3 (C-14’), 56.5 (C-14), 56.0 (C-17), 42.7 (C-13), 41.9 (C-5), 40.4 (C-9), 40.1 (C-12), 35.8 (C-8), 35.4 (C-20), 35.0 (C-1), 34.6 (C-10), 33.4 (C-2’), 32.2 (C-4), 31.3 (C-23), 31.0 (C-22), 28.2 (C-13’, C-16), 27.3 (C-7’, C-8’), 27.0 (C-2), 26.8, 26.5 (C-4’, C-11’), 26.6 (C-6), 26.3 (C-7), 25.9 (C-12’), 24.6 (C-3’), 24.2 (C-15), 23.3 (C-19), 21.5 (COCH_3_), 20.8 (C-11), 18.3 (C-21), 12.0 (C-18); anal. calcd for C_40_H_64_O_6_: C, 74.96; H, 10.06; found C, 74.84; H, 9.99.

#### 3.9.5. General Procedure for the Synthesis of Ethylene Glycol Derivatives of 3a-Acetoxy-5β-cholan-24-oic Acid and 3-Oxo-cholan-24-oic Acid **13a**–**d**, **14a**–**d**

To a solution of 3α-acetoxy-5β-cholan-24-oic acid 9 or 3-oxo-cholan-24-oic acid 12 (1.0 mmol) in CH_2_Cl_2_ (50 mL), ethylene glycol (2.5 mmol) was added, followed by EDC·HCl (0.58 g, 3.0 mmol) and DMAP (18 mg, 0.15 mmol) under argon. The mixture was stirred at room temperature for 16 h until the reaction was complete (TLC monitoring, hexane/ethyl acetate). The mixture was diluted with H_2_O (10 mL) and the CH_2_Cl_2_ layer was separated, dried over MgSO_4_, and concentrated. The crude product was isolated by column chromatography (silica gel) using hexane/ethyl acetate (1:1) as the elution solvent for 13a, 14a and hexane/ethyl acetate (1:3) for **13b**–**d**, **14b**–**d**.

2-Hydroxyethyl (3α,5β)-3-(Acetyloxy)cholan-24-oate (**13a**)

White crystals, 0.35 g, 76% yield. MP 130–132 °С, [α]_D_^19^ + 24.9 (c 1.05, CHCl_3_); IR (KBr) ν_max_ 3016, 2938, 2868, 1736, 1449, 1381, 1363, 1243, 1170, 1086, 1027, 981, 949, 887, 756, 667, 615 cm^−1^; ^1^H NMR (CDCl_3_, 500 MHz) δ 4.75–4.69 (1H, m, H-3), 4.23–4.19 (2H, m, СH_2_O), 3.84–3.80 (2H, m, СH_2_OH), 2.42‒1.02 (28H, m), 2.03 (3H, s, COCH_3_), 0.93 (3H, s, H-19), 0.92 (3H, d, J = 6.4 Hz, H-21), 0.65 (3H, s, H-18); ^13^C NMR (CDCl_3_, 125 MHz) δ 174.7 (C-24), 170.7 (COCH_3_), 74.4 (C-3), 65.9 (СH_2_O), 61.2 (СH_2_OH), 56.5 (C-14), 55.9 (C-17), 42.7 (C-13), 41.9 (C-5), 40.4 (C-9), 40.1 (C-12), 35.8 (C-8), 35.4 (C-20), 35.0 (C-1), 34.6 (C-10), 32.2 (C-4), 31.1 (C-23), 30.9 (C-22), 28.2 (C-16), 27.0 (C-6), 26.6 (C-2), 26.3 (C-7), 24.2 (C-15), 23.3 (C-19), 21.5 (COCH_3_), 20.8 (C-11), 18.2 (C-21), 12.0 (C-18); anal. calcd for C_28_H_46_O_5_: C, 72.69; H, 10.02; found C, 72.57; H, 9.96.

2-(2-Hydroxyethoxy)ethyl (3α,5β)-3-(Acetyloxy)cholan-24-oate (**13b**)

White crystals, 0.38 g, 74% yield. MP 72–74 °С, [α]_D_^19^ + 17.0 (c 0.82, CHCl_3_); IR (KBr) ν_max_ 2938, 2868, 1736, 1449, 1381, 1363, 1243, 1171, 1132, 1066, 1028, 982, 949, 889, 755, 666, 616 cm^−1^; ^1^H NMR (CDCl_3_, 500 MHz) δ 4.63–4.57 (1H, m, H-3), 4.15–4.12 (2H, m, СH_2_O), 3.65–3.57 (4H, m, СH_2_O, СH_2_OH), 3.51–3.48 (2H, m, СH_2_O), 2.71 (1H, br s, OH), 2.32‒0.90 (28H, m), 1.91 (3H, s, COCH_3_), 0.83 (3H, s, H-19), 0.81 (3H, d, J = 6.4 Hz, H-21), 0.55 (3H, s, H-18); ^13^C NMR (CDCl_3_, 125 MHz) δ 174.0 (C-24), 170.4 (COCH_3_), 74.2 (C-3), 72.4 (СH_2_O), 69.0 (СH_2_O), 63.2 (СH_2_O), 61.5 (СH_2_OH), 56.4 (C-14), 55.9 (C-17), 42.6 (C-13), 41.8 (C-5), 40.3 (C-9), 40.0 (C-12), 35.7 (C-8), 35.2 (C-20), 34.9 (C-1), 34.5 (C-10), 32.1 (C-4), 30.9 (C-23), 30.8 (C-22), 28.1 (C-16), 26.9 (C-6), 26.5 (C-2), 26.2 (C-7), 24.1 (C-15), 23.2 (C-19), 21.3 (COCH3), 20.7 (C-11), 18.2 (C-21), 11.9 (C-18); anal. calcd for C_30_H_50_O_6_: C, 71.11; H, 9.95; found C, 71.01; H, 9.86.

2-[2-(2-Hydroxyethoxy)ethoxy]ethyl (3α,5β)-3-(Acetyloxy)cholan-24-oate (**13c**)

Colorless waxy solid, 0.39 g, 71% yield. [α]_D_^19^ + 17.0 (c 0.82, CHCl_3_); IR (KBr) ν_max_ 2938, 2867, 1735, 1635, 1450, 1384, 1243, 1171, 1124, 1066, 1026, 945, 880, 805, 722 cm^−1^; ^1^H NMR (CDCl_3_, 500 MHz) δ 4.66–4.60 (1H, m, H-3), 4.17–4.15 (2H, m, СH_2_O), 3.64–3.58 (8H, m, СH_2_O, СH_2_OH), 3.56–3.53 (2H, m, СH_2_O), 2.84 (1H, br s, OH), 2.35‒0.92 (28H, m), 1.95 (3H, s, COCH_3_), 0.86 (3H, s, H-19), 0.84 (3H, d, J = 6.5 Hz, H-21), 0.58 (3H, s, H-18); ^13^C NMR (CDCl_3_, 125 MHz) δ 174.1 (C-24), 170.5 (COCH_3_), 74.3 (C-3), 72.5 (СH_2_O), 70.5, 70.3 (СH_2_O), 69.1 (СH_2_O), 63.2 (СH_2_O), 61.6 (СH_2_OH), 56.4 (C-14), 55.9 (C-17), 42.7 (C-13), 41.8 (C-5), 40.3 (C-9), 40.1 (C-12), 35.7 (C-8), 35.3 (C-20), 34.9 (C-1), 34.5 (C-10), 32.2 (C-4), 31.0 (C-23), 30.8 (C-22), 28.1 (C-16), 26.9 (C-6), 26.6 (C-2), 26.3 (C-7), 24.1 (C-15), 23.3 (C-19), 21.4 (COCH_3_), 20.8 (C-11), 18.2 (C-21), 11.9 (C-18); anal. calcd for C_32_H_54_O_7_: C, 69.78; H, 9.88; found C, 69.61; H, 9.85.

2-{2-[2-(2-Hydroxyethoxy)ethoxy]ethoxy}ethyl (3α,5β)-3-(Acetyloxy)cholan-24-oate (**13d**)

Colorless waxy solid, 0.41 g, 70% yield. [α]_D_^19^ + 21.8 (c 0.64, CHCl_3_); IR (KBr) ν_max_ 2938, 2867, 1735, 1636, 1450, 1381, 1243, 1171, 1124, 1066, 1028, 949, 914, 887, 845, 803, 733, 661, 615 cm^−1^; ^1^H NMR (CDCl_3_, 500 MHz) δ 4.70–4.64 (1H, m, H-3), 4.20–4.18 (2H, m, СH_2_O), 3.71–3.59 (12H, m, СH_2_O, СH_2_OH), 3.57–3.55 (2H, m, СH_2_O), 2.87 (1H, br s, OH), 2.35‒0.95 (28H, m), 1.98 (3H, s, COCH_3_), 0.89 (3H, s, H-19), 0.87 (3H, d, J = 6.5 Hz, H-21), 0.60 (3H, s, H-18); ^13^C NMR (CDCl_3_, 125 MHz) δ 174.2 (C-24), 170.6 (COCH_3_), 74.3 (C-3), 72.6 (СH_2_O), 70.6, 70.5, 70.3, (СH_2_O), 69.2 (СH_2_O), 63.3 (СH_2_O), 61.6 (СH_2_OH), 56.4 (C-14), 55.9 (C-17), 42.7 (C-13), 41.8 (C-5), 40.6 (C-9), 40.1 (C-12), 35.7 (C-8), 35.3 (C-20), 34.9 (C-1), 34.5 (C-10), 32.2 (C-4), 31.1 (C-23), 30.8 (C-22), 28.1 (C-16), 26.9 (C-6), 26.6 (C-2), 26.3 (C-7), 24.1 (C-15), 23.3 (C-19), 21.4 (COCH_3_), 20.8 (C-11), 18.2 (C-21), 12.0 (C-18); anal. calcd for C_34_H_58_O_8_: C, 68.65; H, 9.83; found C, 68.55; H, 9.74.

2-Hydroxyethyl (5β)-3-Oxocholan-24-oate (**14a**)

White crystals, 0.32 g, 77% yield. MP 134–136 °С, [α]_D_^19^ + 27.0 (c 0.79, CHCl_3_); IR (KBr) ν_max_ 2936, 2865, 1734, 1713, 1446, 1381, 1299, 1249, 1179, 1084, 1035, 946, 887, 756, 667, 615 cm^−1^; ^1^H NMR (CDCl_3_, 500 MHz) δ 4.16–4.10 (2H, m, СH_2_O), 3.77–3.73 (2H, m, СH_2_OH), 2.87 (1H, br s, OH), 2.70‒1.02 (28H, m), 0.96 (3H, s, H-19), 0.87 (3H, d, J = 6.5 Hz, H-21), 0.62 (3H, s, H-18); ^13^C NMR (CDCl_3_, 125 MHz) δ 213.6 (C-3), 174.7 (C-24), 65.9 (СH_2_O), 60.9 (СH_2_OH), 56.4 (C-14), 55.9 (C-17), 44.3 (C-5), 42.7 (C-13), 42.3 (C-4), 40.6 (C-9), 39.9 (C-12), 37.1 (C-2), 36.9 (C-1), 35.5 (C-8), 35.3 (C-20), 34.8 (C-10), 31.1 (C-23), 30.8 (C-22), 28.1 (C-16), 26.6 (C-6), 25.7 (C-7), 24.1 (C-15), 22.6 (C-19), 21.1 (C-11), 18.3 (C-21), 12.0 (C-18); anal. calcd for C_26_H_42_O_4_: C, 74.60; H, 10.11; found C, 74.51; H, 10.02.

2-(2-Hydroxyethoxy)ethyl (5β)-3-Oxocholan-24-oate (**14b**)

Colorless waxy solid, 0.35 g, 75% yield. [α]_D_^19^ + 20.0 (c 0.81, CHCl_3_); IR (KBr) ν_max_ 2934, 2866, 1735, 1714, 1451, 1379, 1298, 1254, 1173, 1132, 1072, 966, 946, 889, 769, 615 cm^−1^; ^1^H NMR (CDCl_3_, 500 MHz) δ 4.24–4.20 (2H, m, СH_2_O), 3.74–3.67 (4H, m, СH_2_O, СH_2_OH), 3.60–3.58 (2H, m, СH_2_O), 2.71‒1.03 (28H, m), 0.99 (3H, s, H-19), 0.90 (3H, d, J = 6.5 Hz, H-21), 0.66 (3H, s, H-18); ^13^C NMR (CDCl_3_, 125 MHz) δ 213.4 (C-3), 174.2 (C-24), 72.3 (СH_2_O), 69.1 (СH_2_O), 63.3 (СH_2_O), 61.7 (СH_2_OH), 56.4 (C-14), 55.9 (C-17), 44.3 (C-5), 42.7 (C-13), 42.3 (C-4), 40.7 (C-9), 40.0 (C-12), 37.2 (C-2), 36.9 (C-1), 35.5 (C-8), 35.3 (C-20), 34.9 (C-10), 31.1 (C-23), 30.9 (C-22), 28.1 (C-16), 26.9 (C-6), 25.7 (C-7), 24.1 (C-15), 22.6 (C-19), 21.2 (C-11), 18.3 (C-21), 12.1 (C-18); anal. calcd for C_28_H_46_O_5_: C, 72.69; H, 10.02; found C, 72.56; H, 9.97.

2-[2-(2-Hydroxyethoxy)ethoxy]ethyl (5β)-3-Oxocholan-24-oateb (**14c**)

Colorless waxy solid, 0.37 g, 73% yield. [α]_D_^17^ + 20.5 (c 0.89, CHCl_3_); IR (KBr) ν_max_ 2930, 2866, 1735, 1714, 1619, 1450, 1383, 1352, 1298, 1255, 1173, 1124, 1104, 1068, 945, 805, 530 cm^−1^; ^1^H NMR (CDCl_3_, 500 MHz) δ 4.20–4.18 (2H, m, СH_2_O), 3.69–3.61 (8H, m, СH_2_O, СH_2_OH), 3.56–3.54 (2H, m, СH_2_O), 2.72 (1H, br s, OH), 2.67‒1.03 (28H, m), 0.97 (3H, s, H-19), 0.87 (3H, d, J = 6.5 Hz, H-21), 0.63 (3H, s, H-18); ^13^C NMR (CDCl_3_, 125 MHz) δ 213.2 (C-3), 174.1 (C-24), 72.5 (СH_2_O), 70.5, 70.3 (СH_2_O), 69.1 (СH_2_O), 63.2 (СH_2_O), 61.6 (СH_2_OH), 56.4 (C-14), 55.9 (C-17), 44.3 (C-5), 42.7 (C-13), 42.3 (C-4), 40.6 (C-9), 39.9 (C-12), 37.1 (C-2), 36.9 (C-1), 35.5 (C-8), 35.3 (C-20), 34.8 (C-10), 31.0 (C-23), 30.8 (C-22), 28.1 (C-16), 26.6 (C-6), 25.7 (C-7), 24.1 (C-15), 22.6 (C-19), 21.1 (C-11), 18.2 (C-21), 12.0 (C-18); anal. calcd for C_30_H_50_O_6_: C, 71.11; H, 9.95; found C, 71.06; H, 9.89.

2-{2-[2-(2-Hydroxyethoxy)ethoxy]ethoxy}ethyl (5β)-3-Oxocholan-24-oate (**14d**)

Сolorless waxy solid, 0.39 g, 70% yield. [α]_D_^18^ + 17.2 (c 0.99, CHCl_3_); IR (KBr) ν_max_ 2931, 2866, 1734, 1714, 1619, 1451, 1383, 1298, 1259, 1173, 1125, 1105, 945, 887, 801, 531 cm^−1^; ^1^H NMR (CDCl_3_, 500 MHz) δ 4.19–4.16 (2H, m, СH_2_O), 3.70–3.60 (12H, m, СH_2_O, СH_2_OH), 3.56–3.54 (2H, m, СH_2_O), 2.83 (1H, br s, OH), 2.67‒1.03 (28H, m), 0.96 (3H, s, H-19), 0.87 (3H, d, J = 6.5 Hz, H-21), 0.63 (3H, s, H-18); ^13^C NMR (CDCl_3_, 125 MHz) δ 213.2 (C-3), 174.1 (C-24), 72.5 (СH_2_O), 70.6, 70.5, 70.3, (СH_2_O), 69.2 (СH_2_O), 63.3 (СH_2_O), 61.6 (СH_2_OH), 56.4 (C-14), 55.9 (C-17), 44.3 (C-5), 42.7 (C-13), 42.3 (C-4), 40.6 (C-9), 39.9 (C-12), 37.1 (C-2), 36.9 (C-1), 35.5 (C-8), 35.3 (C-20), 34.8 (C-10), 31.0 (C-23), 30.8 (C-22), 28.1 (C-16), 26.6 (C-6), 25.7 (C-7), 24.1 (C-15), 22.6 (C-19), 21.1 (C-11), 18.2 (C-21), 12.0 (C-18); anal. calcd for C_32_H_54_O_7_: C, 69.78; H, 9.88; found C, 69.72; H, 9.83.

#### 3.9.6. Reaction of Ethylene Glycol Derivatives of 3a-Acetoxy-5β-cholan-24-oic Acid and 3-Oxo-cholan-24-oic Acid **13a**–**d**, **14a**–**d** with (5*Z*,9*Z*)-Tetradeca-5,9-dienedioic Acid (**5**)

Lithocholic acid derivative **15a**–**d** and **16a**–**d** was synthesized according to a modified literature procedure [26]. To a solution of ethylene glycol derivatives of 3α-acetoxy-5β-cholan-24-oic acid **13a**–**d** or 3-oxo-cholan-24-oic acid **14a**–**d** (1.0 mmol) in CH_2_Cl_2_ (50 mL), (5*Z*,9*Z*)-tetradeca-5.9-dienedioic acid (**5**) (0.51 g, 2.0 mmol) was added, followed by EDC·HCl (0.48 g, 2.5 mmol) and DMAP (18 mg, 0.15 mmol) under argon. The mixture was stirred at room temperature for 16 h until the reaction was complete (TLC monitoring, hexane/ethyl acetate). The mixture was diluted with H_2_O (10 mL) and the CH_2_Cl_2_ layer was separated, dried over MgSO_4_, and concentrated. The crude product was purified by column chromatography (silica gel) using hexane/ethyl acetate (2:1) as the elution solvent to afford compounds **15a**–**d** or **16a**–**d**.

(5*Z*,9*Z*)-14-(2-{[(3α,5β)-3-(Acetyloxy)-24-oxocholan-24-yl]oxy}ethoxy)-14-oxotetradeca-5,9-dienoic acid (**15a**)

Colorless waxy solid, 0.44 g, 64% yield. [α]_D_^19^ + 15.9 (c 0.76, CHCl_3_); IR (KBr) ν_max_ 3065, 2937, 2865, 1947, 1735, 1679, 1450, 1385, 1244, 1141, 1105, 1026, 870, 803, 750, 690, 613 cm^−1^; ^1^H NMR (CDCl_3_, 500 MHz) δ 5.45–5.32 (4H, m, H-5’, H-6’, H-9’, H-10’), 4.75–4.71 (1H, m, H-3), 4.41–4.39 (4H, m, СH_2_O), 2.40‒1.02 (28H, m), 2.39–2.33 (4H, m, H-2’, H-13’), 2.13–2.05 (8H, m, H-4’, H-7’, H-8’, H-11’), 2.04 (3H, s, COCH_3_), 1.74–1.67 (4H, m, H-3’, H-12’), 0.94 (3H, s, H-19), 0.92 (3H, d, J = 6.5 Hz, H-21), 0.66 (3H, s, H-18); ^13^C NMR (CDCl_3_, 125 MHz) δ 178.7 (C-1’), 174.1 (C-24), 173.6 (C-14’), 170.7 (COCH_3_), 130.4, 130.3 (C-6’, C-9’), 128.9 (C-5’, C-10’), 74.4 (C-3), 62.1 (СH_2_O), 62.0 (СH_2_O), 56.5 (C-14), 56.0 (C-17), 42.7 (C-13), 41.9 (C-5), 40.4 (C-9), 40.1 (C-12), 35.8 (C-8), 35.3 (C-20), 35.0 (C-1), 34.6 (C-10), 33.5 (C-13’), 33.2 (С-2’), 32.2 (C-4), 31.1 (C-23), 30.9 (C-22), 28.2 (C-16), 27.3 (C-7’, C-8’), 27.0 (C-6), 26.6 (C-4’, C-11’), 26.5 (C-2), 26.3 (C-7), 24.8 (C-12’), 24.6 (C-3’), 24.2 (C-15), 23.3 (C-19), 21.5 (COCH_3_), 20.8 (C-11), 18.3 (C-21), 12.0 (C-18); anal. calcd for C_42_H_66_O_8_: C, 72.17; H, 9.52; found C, 72.11; H, 9.45.

(5*Z*,9*Z*)-14-[2-(2-{[(3α,5β)-3-(Acetyloxy)-24-oxocholan-24-yl]oxy}ethoxy)ethoxy]-14-oxotetradeca-5,9-dienoic Acid (**15b**)

Colorless waxy solid, 0.46 g, 63% yield. [α]_D_^19^ + 20.0 (c 0.95, CHCl_3_); IR (KBr) ν_max_ 3063, 2938, 2863, 1947, 1734, 1679, 1451, 1383, 1244, 1139, 1103, 1026, 870, 801, 750, 693, 614 cm^−1^; ^1^H NMR (CDCl_3_, 500 MHz) δ 5.42–5.31 (4H, m, H-5’, H-6’, H-9’, H-10’), 4.72–4.68 (1H, m, H-3), 4.23–4.20 (4H, m, СH_2_O), 3.70–3.67 (4H, m, СH_2_O), 2.40‒0.95 (28H, m), 2.36–2.30 (4H, m, H-2’, H-13’), 2.10–2.03 (8H, m, H-4’, H-7’, H-8’, H-11’), 2.01 (3H, s, COCH_3_), 1.73–1.66 (4H, m, H-3’, H-12’), 0.91 (3H, s, H-19), 0.89 (3H, d, J = 6.5 Hz, H-21), 0.63 (3H, s, H-18); ^13^C NMR (CDCl_3_, 125 MHz) δ 178.8 (C-1’), 174.2 (C-24), 173.7 (C-14’), 170.7 (COCH_3_), 130.3, 130.2 (C-6’, C-9’), 128.9, 128.8 (C-5’, C-10’), 74.4 (C-3), 69.0 (СH_2_O), 63.2 (СH_2_O), 56.5 (C-14), 55.9 (C-17), 42.7 (C-13), 41.9 (C-5), 40.4 (C-9), 40.1 (C-12), 35.8 (C-8), 35.3 (C-20), 35.0 (C-1), 34.6 (C-10), 33.5 (C-13’), 33.3 (С-2’), 32.2 (C-4), 31.1 (C-23), 30.9 (C-22), 28.2 (C-16), 27.3, 27.2 (C-7’, C-8’), 26.9 (C-6), 26.6 (C-11’), 26.5 (C-2, C-4’), 26.3 (C-7), 24.8 (C-12’), 24.6 (C-3’), 24.2 (C-15), 23.3 (C-19), 21.4 (COCH_3_), 20.8 (C-11), 18.3 (C-21), 12.0 (C-18); anal. calcd for C_44_H_70_O_9_: C, 71.12; H, 9.50; found C, 71.01; H, 9.43.

(5*Z*,9*Z*)-14-{2-[2-(2-{[(3α,5β)-3-(Acetyloxy)-24-oxocholan-24-yl]oxy}ethoxy)ethoxy]ethoxy}-14-oxotetradeca-5,9-dienoic Acid (**15c**)

Colorless waxy solid, 0.48 g, 61% yield. [α]_D_^19^ + 15.5 (c 0.85, CHCl_3_); IR (KBr) ν_max_ 3065, 2938, 2867, 1946, 1736, 1681, 1454, 1381, 1245, 1100, 1027, 981, 873, 802, 735, 698, 615 cm^−1^; ^1^H NMR (CDCl_3_, 500 MHz) δ 5.45–5.30 (4H, m, H-5’, H-6’, H-9’, H-10’), 4.77–4.72 (1H, m, H-3), 4.26–4.23 (4H, m, СH_2_O), 3.74–3.71 (4H, m, СH_2_O), 3.69–3.67 (4H, m, СH_2_O), 2.40‒1.00 (28H, m), 2.39–2.33 (4H, m, H-2’, H-13’), 2.13–2.06 (8H, m, H-4’, H-7’, H-8’, H-11’), 2.04 (3H, s, COCH_3_), 1.74–1.67 (4H, m, H-3’, H-12’), 0.94 (3H, s, H-19), 0.92 (3H, d, J = 6.5 Hz, H-21), 0.65 (3H, s, H-18); ^13^C NMR (CDCl_3_, 125 MHz) δ 178.3 (C-1’), 174.3 (C-24), 173.8 (C-14’), 170.7 (COCH_3_), 130.4, 130.2 (C-6’, C-9’), 128.9 (C-5’, C-10’), 74.4 (C-3), 70.5 (СH_2_O), 69.3 (СH_2_O), 63.3 СH_2_O), 56.5 (C-14), 56.0 (C-17), 42.7 (C-13), 41.9 (C-5), 40.4 (C-9), 40.1 (C-12), 35.8 (C-8), 35.4 (C-20), 35.0 (C-1), 34.6 (C-10), 33.6 (C-13’), 33.2 (С-2’), 32.2 (C-4), 31.1 (C-23), 30.9 (C-22), 28.2 (C-16), 27.3 (C-7’, C-8’), 27.0 (C-6), 26.6 (C-4’, C-11’), 26.5 (C-2), 26.3 (C-7), 24.8 (C-12’), 24.6 (C-3’), 24.2 (C-15), 23.3 (C-19), 21.5 (COCH_3_), 20.8 (C-11), 18.3 (C-21), 12.0 (C-18); anal. calcd for C_46_H_74_O_10_: C, 70.20; H, 9.48; found C, 70.13; H, 9.43.

(5*Z*,9*Z*)-14-{2-[2-(2-{[(3α,5β)-3-(Acetyloxy)-24-oxocholan-24-yl]oxy}ethoxy)ethoxy]ethoxy}-14-oxotetradeca-5,9-dienoic Acid (**15d**)

Colorless waxy solid, 0.50 g, 61% yield. [α]_D_^19^ + 14.0 (c 0.90, CHCl_3_); IR (KBr) ν_max_ 2929, 2868, 1736, 1619, 1451, 1383, 1363, 1243, 1142, 1100, 1028, 802, 755, 673, 614 cm^−1^; ^1^H NMR (CDCl_3_, 500 MHz) δ 5.44–5.33 (4H, m, H-5’, H-6’, H-9’, H-10’), 4.76–4.70 (1H, m, H-3), 4.26–4.23 (4H, m, СH_2_O), 3.74–3.71 (4H, m, СH_2_O), 3.69–3.67 (8H, m, СH_2_O), 2.40‒1.00 (28H, m), 2.39–2.33 (4H, m, H-2’, H-13’), 2.13–2.05 (8H, m, H-4’, H-7’, H-8’, H-11’), 2.04 (3H, s, COCH_3_), 1.74–1.67 (4H, m, H-3’, H-12’), 0.94 (3H, s, H-19), 0.92 (3H, d, J = 6.5 Hz, H-21), 0.65 (3H, s, H-18); ^13^C NMR (CDCl_3_, 125 MHz) δ 177.3 (C-1’), 174.3 (C-24), 173.8 (C-14’), 170.7 (COCH_3_), 130.3, 130.2 (C-6’, C-9’), 128.9 (C-5’, C-10’), 74.4 (C-3), 70.6 (СH_2_O), 70.5 (СH_2_O), 69.32 (СH_2_O), 63.4 (СH_2_O), 56.5 (C-14), 56.0 (C-17), 42.7 (C-13), 41.9 (C-5), 40.4 (C-9), 40.1 (C-12), 35.8 (C-8), 35.4 (C-20), 35.0 (C-1), 34.6 (C-10), 33.6 (C-13’), 33.1 (С-2’), 32.3 (C-4), 31.1 (C-23), 30.9 (C-22), 28.2 (C-16), 27.3 (C-7’, C-8’), 27.0 (C-6), 26.6 (C-2, C-11’), 26.5 (C-4’), 26.3 (C-7), 24.8 (C-12’), 24.6 (C-3’), 24.2 (C-15), 23.3 (C-19), 21.5 (COCH_3_), 20.8 (C-11), 18.3 (C-21), 12.0 (C-18); anal. calcd for C_48_H_78_O_11_: C, 69.37; H, 9.46; found C, 69.30; H, 9.39.

(5*Z*,9*Z*)-14-(2-{[(5β)-3,24-Dioxocholan-24-yl]oxy}ethoxy)-14-oxotetradeca-5,9-dienoic Acid (**16a**)

Colorless waxy solid, 0.42 g, 65% yield. [α]_D_^19^ + 16.9 (c 0.73, CHCl_3_); IR (KBr) ν_max_ 3063, 2938, 2865, 1953, 1729, 1679, 1443, 1385, 1239, 1125, 1105, 1011, 875, 803, 750, 690, 615 cm^−1^; ^1^H NMR (CDCl_3_, 500 MHz) δ 5.44–5.33 (4H, m, H-5’, H-6’, H-9’, H-10’), 4.31–4.26 (4H, m, СH_2_O), 2.73‒1.06 (28H, m), 2.39–2.32 (4H, m, H-2’, H-13’), 2.14–2.06 (8H, m, H-4’, H-7’, H-8’, H-11’), 1.74–1.67 (4H, m, H-3’, H-12’), 1.02 (3H, s, H-19), 0.92 (3H, d, J = 6.5 Hz, H-21), 0.69 (3H, s, H-18); ^13^C NMR (CDCl_3_, 125 MHz) δ 213.6 (C-3), 178.3 (C-1’), 174.0 (C-24), 173.6 (C-14’), 130.3 (C-6’, C-9’), 128.9 (C-5’, C-10’), 62.1 (СH_2_O), 62.0 (СH_2_O), 56.4 (C-14), 55.9 (C-17), 44.3 (C-5), 42.8 (C-13), 42.3 (C-4), 40.7 (C-9), 40.0 (C-12), 37.2 (C-2), 36.9 (C-1), 35.5 (C-8), 35.3 (C-20), 34.9 (C-10), 33.5 (C-13’), 33.2 (С-2’), 31.1 (C-22), 30.9 (C-23), 28.2 (C-16), 27.3 (C-7’, C-8’), 26.6 (C-6), 26.5 (C-4’, C-11’), 25.8 (C-7), 24.8 (C-12’), 24.6 (C-3’), 24.2 (C-15), 22.6 (C-19), 21.2 (C-11), 18.3 (C-21), 12.1 (C-18); anal. calcd for C_40_H_62_O_7_: C, 73.36; H, 9.54; found C, 73.29; H, 9.50.

(5*Z*,9*Z*)-14-[2-(2-{[(5β)-3,24-Dioxocholan-24-yl]oxy}ethoxy)ethoxy]-14-oxotetradeca-5,9-dienoic Acid (**16b**)

Colorless waxy solid, 0.44 g, 64% yield. [α]_D_^19^ + 17.9 (c 0.78, CHCl_3_); IR (KBr) ν_max_ 3066, 2938, 2863, 1951, 1731, 1675, 1441, 1385, 1235, 1120, 1101, 1011, 875, 801, 750, 693, 615 cm^−1^; ^1^H NMR (CDCl_3_, 500 MHz) δ 5.45–5.33 (4H, m, H-5’, H-6’, H-9’, H-10’), 4.26–4.22 (4H, m, СH_2_O), 3.74–3.69 (4H, m, СH_2_O), 2.73‒1.06 (28H, m), 2.39–2.32 (4H, m, H-2’, H-13’), 2.14–2.06 (8H, m, H-4’, H-7’, H-8’, H-11’), 1.74–1.66 (4H, m, H-3’, H-12’), 1.03 (3H, s, H-19), 0.93 (3H, d, J = 6.5 Hz, H-21), 0.69 (3H, s, H-18); ^13^C NMR (CDCl_3_, 125 MHz) δ 213.6 (C-3), 178.3 (C-1’), 174.2 (C-24), 173.8 (C-14’), 130.3, 130.2 (C-6’, C-9’), 128.9 (C-5’, C-10’), 69.1 (СH_2_O), 63.3 (СH_2_O), 56.4 (C-14), 55.9 (C-17), 44.3 (C-5), 42.8 (C-13), 42.3 (C-4), 40.7 (C-9), 40.0 (C-12), 37.2 (C-2), 37.0 (C-1), 35.5 (C-8), 35.3 (C-20), 34.9 (C-10), 33.6 (C-13’), 33.2 (С-2’), 31.1 (C-22), 30.9 (C-23), 28.2 (C-16), 27.3 (C-7’, C-8’), 26.6 (C-6), 26.5 (C-4’, C-11’), 25.8 (C-7), 24.8 (C-12’), 24.6 (C-3’), 24.2 (C-15), 22.6 (C-19), 21.2 (C-11), 18.3 (C-21), 12.1 (C-18); anal. calcd for C_42_H_66_O_8_: C, 72.17; H, 9.52; found C, 72.07; H, 9.45.

(5*Z*,9*Z*)-14-{2-[2-(2-{[(5β)-3,24-Dioxocholan-24-yl]oxy}ethoxy)ethoxy]ethoxy}-14-oxotetradeca-5,9-dienoic Acid (**16c**)

Colorless waxy solid, 0.45 g, 61% yield. [α]_D_^18^ + 12.5 (c 0.93, CHCl_3_); IR (KBr) ν_max_ 2929, 2866, 1736, 1712, 1619, 1452, 1383, 1298, 1245, 1173, 1143, 1106, 1044, 965, 860, 731, 530 cm^−1^; ^1^H NMR (CDCl_3_, 500 MHz) δ 5.40–5.31 (4H, m, H-5’, H-6’, H-9’, H-10’), 4.24–4.20 (4H, m, СH_2_O), 3.69–3.66 (4H, m, СH_2_O), 3.65–3.63 (4H, m, СH_2_O), 2.70‒1.04 (28H, m), 2.37–2.30 (4H, m, H-2’, H-13’), 2.13–2.03 (8H, m, H-4’, H-7’, H-8’, H-11’), 1.71–1.65 (4H, m, H-3’, H-12’), 0.99 (3H, s, H-19), 0.90 (3H, d, J = 6.5 Hz, H-21), 0.66 (3H, s, H-18); ^13^C NMR (CDCl_3_, 125 MHz) δ 213.7 (C-3), 178.5 (C-1’), 174.2 (C-24), 173.7 (C-14’), 130.3, 130.2 (C-6’, C-9’), 128.9, 128.8 (C-5’, C-10’), 70.5 (СH_2_O), 69.3 (СH_2_O), 63.3 (СH_2_O), 56.4 (C-14), 55.9 (C-17), 44.3 (C-5), 42.7 (C-13), 42.3 (C-4), 40.7 (C-9), 40.0 (C-12), 37.1 (C-2), 36.9 (C-1), 35.5 (C-8), 35.3 (C-20), 34.9 (C-10), 33.6 (C-13’), 33.3 (С-2’), 31.1 (C-23), 30.8 (C-22), 28.1 (C-16), 27.3, 27.2 (C-7’, C-8’), 26.6 (C-6), 26.5 (C-4’, C-11’), 25.7 (C-7), 24.8 (C-12’), 24.6 (C-3’), 24.1 (C-15), 22.6 (C-19), 21.2 (C-11), 18.3 (C-21), 12.1 (C-18); anal. calcd for C_44_H_70_O_9_: C, 71.12; H, 9.50; found C, 71.02; H, 9.44.

(5*Z*,9*Z*)-14-{2-[2-(2-{[(5β)-3,24-Dioxocholan-24-yl]oxy}ethoxy)ethoxy]ethoxy}-14-oxotetradeca-5,9-dienoic Acid (**16d**)

Colorless waxy solid, 0.48 g, 60% yield. [α]_D_^18^ + 10.4 (c 0.75, CHCl_3_); IR (KBr) ν_max_ 2929, 2866, 1735, 1715, 1619, 1456, 1384, 1298, 1246, 1172, 1143, 1104, 1041, 947, 859, 825, 732, 530 cm^−1^; ^1^H NMR (CDCl_3_, 500 MHz) δ 5.45–5.34 (4H, m, H-5’, H-6’, H-9’, H-10’), 4.26–4.23 (4H, m, СH_2_O), 3.74–3.69 (4H, m, СH_2_O), 3.68–3.66 (8H, m, СH_2_O), 2.74‒1.06 (28H, m), 2.40–2.33 (4H, m, H-2’, H-13’), 2.14–2.05 (8H, m, H-4’, H-7’, H-8’, H-11’), 1.75–1.67 (4H, m, H-3’, H-12’), 1.03 (3H, s, H-19), 0.93 (3H, d, J = 6.5 Hz, H-21), 0.69 (3H, s, H-18); ^13^C NMR (CDCl_3_, 125 MHz) δ 213.6 (C-3), 177.4 (C-1’), 174.3 (C-24), 173.9 (C-14’), 130.3, 130.2 (C-6’, C-9’), 128.9 (C-5’, C-10’), 70.6 (СH_2_O), 70.5 (СH_2_O), 69.22 (СH_2_O), 63.4 (СH_2_O), 56.4 (C-14), 55.9 (C-17), 44.3 (C-5), 42.8 (C-13), 42.4 (C-4), 40.7 (C-9), 40.1 (C-12), 37.2 (C-2), 37.0 (C-1), 35.5 (C-8), 35.3 (C-20), 34.9 (C-10), 33.6 (C-13’), 33.1 (С-2’), 31.1 (C-23), 30.9 (C-22), 28.2 (C-16), 27.4, 27.3 (C-7’, C-8’), 26.6 (C-6), 26.5 (C-4’, C-11’), 25.8 (C-7), 24.8 (C-12’), 24.6 (C-3’), 24.2 (C-15), 22.7 (C-19), 21.2 (C-11), 18.3 (C-21), 12.1 (C-18); anal. calcd for C_46_H_74_O_10_: C, 70.20; H, 9.48; found C, 70.11; H, 9.41.

#### 3.9.7. General Procedure for the Synthesis of BOC-Protected Diaminoalkane Derivatives of 3a-Acetoxy-5β-cholan-24-oic Acid and 3-Oxo-cholan-24-oic acid **19a**–**d**, **20a**–**d**

Oxalyl chloride (2 mL) was added to a solution of 9 or 12 (1.2 mmol) in CH_2_Cl_2_ (20 mL) and stirred at room temperature for 24 h excluding air humidity, affording the crude 24-acylchloride 17 or 18. After evaporation to dryness, another portion of CH_2_Cl_2_ (10 mL) was added, followed by diisopropylethylamine (0.5 mL, 3.0 mmol) and tert-butyl (2-aminoalcane)carbamate (1.8 mmol), stirred at room temperature for an additional 16 h, then evaporated, and the residue was purified by chromatography on silica gel using hexane/ethyl acetate (1:2) as the mobile phase, affording the product **19a**–**d** or **20a**–**d**.

(3α,5β)-24-({2-[(tert-Butoxycarbonyl)amino]ethyl}amino)-24-oxocholan-3-yl Acetate (**19a**)

White crystals, 0.60 g, 90% yield. MP 118–120 °С, [α]_D_^19^ + 32.4 (c 0.76, CHCl_3_); IR (KBr) ν_max_ 2934, 2867, 1719, 1696, 1649, 1450, 1382, 1365, 1244, 1172, 1028, 755, 665, 610 cm^−1^; ^1^H NMR (CDCl_3_, 500 MHz) δ 6.62 (1H, br s, NH), 5.31 (1H, t, J = 6.5 Hz, NH), 4.69–4.63 (1H, m, H-3), 3.32–3.28 (2H, m, CH_2_N), 3.23–3.17 (2H, m, CH_2_N), 2.22‒0.95 (28H, m), 1.98 (3H, s, CH_3_CO), 1.39 (9H, s, CH_3_), 0.88 (3H, s, H-19), 0.86 (3H, d, J = 6.5 Hz, H-21), 0.59 (3H, s, H-18); ^13^C NMR (CDCl_3_, 125 MHz) δ 174.4 (C-24), 170.6 (CH_3_CO), 156.9 (COO), 79.4 (C(CH_3_)_3_), 74.4 (C-3), 56.4 (C-14), 56.0 (C-17), 42.7 (C-13), 41.8 (C-5), 40.5 (CH_2_N), 40.4 (C-9), 40.3 (CH_2_N), 40.1 (C-12), 35.7 (C-8), 35.5 (C-20), 34.9 (C-1), 34.5 (C-10), 33.5 (C-23), 32.2 (C-4), 31.7 (C-22), 28.4 (CH_3_), 28.2 (C-16), 26.9 (C-6), 26.6 (C-2), 26.3 (C-7), 24.1 (C-15), 23.3 (C-19), 21.4 (CH_3_CO), 20.8 (C-11), 18.4 (C-21), 12.0 (C-18); anal. calcd for C_33_H_56_N_2_O_5_: C, 70.68; H, 10.06; found C, 70.59; H, 9.98.

(3α,5β)-24-({4-[(tert-Butoxycarbonyl)amino]butyl}amino)-24-oxocholan-3-yl Acetate (**19b**)

White crystals, 0.61 g, 87% yield. MP 88–90 °С, [α]_D_^21^ + 26.9 (c 0.86, CHCl_3_); IR (KBr) ν_max_ 2932, 2866, 1736, 1696, 1649, 1534, 1449, 1383, 1364, 1244, 1172, 1027, 754, 666, 608 cm^−1^; ^1^H NMR (CDCl_3_, 500 MHz) δ 6.21 (1H, br s, NH), 4.84 (1H, br s, NH), 4.66–4.60 (1H, m, H-3), 3.21–3.16 (2H, m, CH_2_N), 3.08–3.03 (2H, m, CH_2_N), 2.19‒0.93 (28H, m), 1.97 (3H, s, CH_3_CO), 1.48–1.44 (4H, m, CH_2_), 1.37 (9H, s, CH_3_), 0.86 (3H, s, H-19), 0.85 (3H, d, J = 6.5 Hz, H-21), 0.58 (3H, s, H-18); ^13^C NMR (CDCl_3_, 125 MHz) δ 173.7 (C-24), 170.6 (CH_3_CO), 156.1 (COO), 79.4 (C(CH_3_)_3_), 74.3 (C-3), 56.4 (C-14), 56.0 (C-17), 42.7 (C-13), 41.8 (C-5), 40.4(C-9), 40.1 (C-12, CH_2_N), 39.0 (CH_2_N), 35.7 (C-8), 35.5 (C-20), 34.9 (C-1), 34.5 (C-10), 33.5 (C-23), 32.2 (C-4), 31.8 (C-22), 28.4 (CH_3_), 28.2 (C-16), 27.6 (CH_2_), 26.9 (C-6), 26.6 (C-2, CH_2_), 26.3 (C-7), 24.1 (C-15), 23.3 (C-19), 21.4 (CH_3_CO), 20.8 (C-11), 18.3 (C-21), 12.0 (C-18); anal. calcd for C_35_H_60_N_2_O_5_: C, 71.39; H, 10.27; found C, 71.32; H, 10.21.

(3α,5β)-24-({6-[(tert-Butoxycarbonyl)amino]hexyl}amino)-24-oxocholan-3-yl Acetate (**19c**)

Colorless waxy solid, 0.61 g, 84% yield. [α]_D_^21^ + 26.9 (c 0.86, CHCl_3_); IR (KBr) ν_max_ 2935, 2863, 1734, 1696, 1645, 1534, 1447, 1383, 1363, 1244, 1169, 1027, 753, 665, 608 cm^−1^; ^1^H NMR (CDCl_3_, 500 MHz) δ 5.84 (1H, br s, NH), 4.66–4.62 (1H, m, H-3), 4.64 (1H, br s, NH), 3.20–3.15 (2H, m, CH_2_N), 3.08–3.02 (2H, m, CH_2_N), 2.21‒0.95 (28H, m), 1.99 (3H, s, CH_3_CO), 1.48–1.40 (4H, m, CH_2_), 1.39 (9H, s, CH_3_), 1.31–1.25 (4H, m, CH_2_), 0.88 (3H, s, H-19), 0.87 (3H, d, J = 6.5 Hz, H-21), 0.59 (3H, s, H-18); ^13^C NMR (CDCl_3_, 125 MHz) δ 173.6 (C-24), 170.6 (CH_3_CO), 156.1 (CO), 78.9 (C(CH_3_)_3_), 74.4 (C-3), 56.5 (C-14), 56.1 (C-17), 42.7 (C-13), 41.9 (C-5), 40.4(C-9), 40.1 (C-12, CH_2_N), 39.1 (CH_2_N), 35.8 (C-8), 35.5 (C-20), 35.0 (C-1), 34.6 (C-10), 33.6 (C-23), 32.2 (C-4), 31.9 (C-22), 29.9 (CH_2_), 29.5 (CH_2_), 28.4 (CH_3_), 28.2 (C-16), 26.9 (C-6), 26.6 (C-2), 26.3 (C-7), 26.2 (CH_2_), 26.1 (CH_2_), 24.2 (C-15), 23.3 (C-19), 21.4 (CH_3_CO), 20.8 (C-11), 18.4 (C-21), 12.0 (C-18); anal. calcd for C_37_H_64_N_2_O_5_: C, 72.04; H, 10.46; found C, 71.96; H, 10.41.

tert-Butyl 4-[(3α,5β)-3(acetyloxy)-24-oxocholan-3-yl]piperazine-1-carboxylate Acetate (**19d**)

Colorless waxy solid, 0.58 g, 83% yield. [α]_D_^21^ + 25.0 (c 0.82, CHCl_3_); IR (KBr) ν_max_ 2933, 2866, 1734, 1698, 1649, 1455, 1419, 1381, 1364, 1285, 1242, 1168, 1127, 1028, 997, 863, 754, 665, 614 cm^−1^; ^1^H NMR (CDCl_3_, 500 MHz) δ 4.72–4.66 (1H, m, H-3), 3.58–3.54 (2H, m, CH_2_N), 3.44–3.36 (6H, m, CH_2_N), 2.38‒0.96 (28H, m), 2.01 (3H, s, CH_3_CO), 1.45 (9H, s, CH_3_), 0.93 (3H, s, H-19), 0.92 (3H, d, J = 6.5 Hz, H-21), 0.63 (3H, s, H-18); ^13^C NMR (CDCl_3_, 125 MHz) δ 172.3 (C-24), 170.6 (CH_3_CO), 154.5 (COO), 80.2 (C(CH_3_)_3_), 74.3 (C-3), 56.5 (C-14), 56.1 (C-17), 45.4 (CH_2_N), 42.7 (C-13), 41.9 (C-5), 41.3 (CH_2_N), 40.4(C-9), 40.1 (C-12), 35.8 (C-8), 35.6 (C-20), 35.0 (C-1), 34.6 (C-10), 32.2 (C-4), 31.4 (C-23), 30.3 (C-22), 28.4 (CH_3_), 28.3 (C-16), 26.9 (C-6), 26.6 (C-2), 26.3 (C-7), 24.2 (C-15), 23.3 (C-19), 21.4 (CH_3_CO), 20.8 (C-11), 18.5 (C-21), 12.0 (C-18); anal. calcd for C_35_H_58_N_2_O_5_: C, 71.63; H, 9.96; found C, 71.58; H, 9.91.

tert-Butyl (2-{[(5β)-3,24-dioxocholan-24-yl)amino]ethyl}carbamate (**20a**)

White waxy solid, 0.55 g, 89% yield. [α]_D_^17^ + 19.0 (c 0.88, CHCl_3_); IR (KBr) ν_max_ 2934, 2867, 1707, 1652, 1531, 1454, 1366, 1269, 1252, 1172, 1028, 754, 665, 531 cm^−1^; ^1^H NMR (CDCl_3_, 500 MHz) δ 6.36 (1H, br s, NH), 5.07 (1H, br s, NH), 3.37–3.33 (2H, m, CH_2_N), 3.29–3.26 (2H, m, CH_2_N), 2.72‒1.05 (28H, m), 1.44 (9H, s, CH_3_), 1.02 (3H, s, H-19), 0.92 (3H, d, J = 6.5 Hz, H-21), 0.68 (3H, s, H-18); ^13^C NMR (CDCl_3_, 125 MHz) δ 213.5 (C-3), 174.3 (C-24), 156.9 (COO), 79.6 (C(CH_3_)_3_), 56.4 (C-14), 56.0 (C-17), 44.3 (C-5), 42.8 (C-13), 42.4 (C-4), 40.7 (C-9, CH_2_N), 40.3 (CH_2_N), 40.1 (C-12), 37.2 (C-2), 37.0 (C-1), 35.5 (C-8, C-20), 34.9 (C-10), 33.5 (C-23), 31.7 (C-22), 28.4 (CH_3_), 28.2 (C-16), 26.6 (C-6), 25.8 (C-7), 24.1 (C-15), 22.6 (C-19), 21.2 (C-11), 18.4 (C-21), 12.1 (C-18); anal. calcd for C_31_H_52_N_2_O_4_: C, 72.05; H, 10.14; found C, 72.00; H, 10.09. MALDI TOF: *m*/*z* 539.387 ([M + Na]^+^, calcd 539.382), 555.362 ([M + K]^+^, calcd 555.356).

tert-Butyl Butyl (4-{[(5β)-3,24-Dioxocholan-24-yl)amino]butyl}carbamate (**20b**)

White waxy solid, 0.55 g, 85% yield. [α]_D_^16^ + 19.3 (c 0.97, CHCl_3_); IR (KBr) ν_max_ 2929, 2865, 1711, 1650, 1536, 1452, 1379, 1269, 1252, 1173, 756, 666 cm^−1^; ^1^H NMR (CDCl_3_, 500 MHz) δ 6.11 (1H, br s, NH), 4.71 (1H, br s, NH), 3.27–3.23 (2H, m, CH_2_N), 3.14–3.10 (2H, m, CH_2_N), 2.71‒1.05 (28H, m), 1.49–1.44 (4H, m, CH_2_), 1.43 (9H, s, CH_3_), 1.01 (3H, s, H-19), 0.92 (3H, d, J = 6.5 Hz, H-21), 0.67 (3H, s, H-18); ^13^C NMR (CDCl_3_, 125 MHz) δ 213.4 (C-3), 173.9 (C-24), 156.2 (COO), 79.2 (C(CH_3_)_3_), 56.4 (C-14), 56.0 (C-17), 44.3 (C-5), 42.8 (C-13), 42.4 (C-4), 40.7 (C-9, CH_2_N), 40.1 (C-12), 39.2 (CH_2_N), 37.2 (C-2), 37.0 (C-1), 35.5 (C-8, C-20), 34.9 (C-10), 33.5 (C-23), 31.9 (C-22), 28.4 (CH_3_), 28.2 (C-16), 27.7 (CH_2_), 26.6 (C-6, CH_2_), 25.8 (C-7), 24.1 (C-15), 22.6 (C-19), 21.2 (C-11), 18.4 (C-21), 12.1 (C-18); anal. calcd for C_33_H_56_N_2_O_4_: C, 72.75; H, 10.36; found C, 72.69; H, 10.33. MALDI TOF: *m*/*z* 689.415 ([M + Na]^+^, calcd 689.512), 705.377 ([M + K]^+^, calcd 705.486).

tert-Butyl (6-{[(5β)-3,24-Dioxocholan-24-yl)amino]hexyl}carbamate (**20c**)

Colorless waxy solid, 0.58 g, 85% yield. [α]_D_^14^ + 14.0 (c 0.95, CHCl_3_); IR (KBr) ν_max_ 2932, 2864, 1710, 1649, 1539, 1454, 1378, 1271, 1251, 1173, 754, 666 cm^−1^; ^1^H NMR (CDCl_3_, 500 MHz) δ 5.95 (1H, br s, NH), 4.68 (1H, br s, NH), 3.20–3.16 (2H, m, CH_2_N), 3.08–3.04 (2H, m, CH_2_N), 2.68‒1.02 (28H, m), 1.47–1.40 (4H, m, CH_2_), 1.39 (9H, s, CH_3_), 1.30–1.26 (4H, m, CH_2_), 0.97 (3H, s, H-19), 0.89 (3H, d, J = 6.5 Hz, H-21), 0.64 (3H, s, H-18); ^13^C NMR (CDCl_3_, 125 MHz) δ 213.4 (C-3), 173.6 (C-24), 156.1 (COO), 78.9 (C(CH_3_)_3_), 56.4 (C-14), 56.0 (C-17), 44.3 (C-5), 42.7 (C-13), 42.3 (C-4), 40.7 (C-9), 40.2 (CH_2_N), 40.0 (C-12), 39.1 (CH_2_N), 37.2 (C-2), 36.9 (C-1), 35.5 (C-8, C-20), 34.8 (C-10), 33.6 (C-23), 31.8 (C-22), 29.9 (CH_2_), 29.5 (CH_2_), 28.4 (CH_3_), 28.2 (C-16), 26.6 (C-6), 26.2 (CH_2_), 26.1 (CH_2_), 25.7 (C-7), 24.1 (C-15), 22.6 (C-19), 21.2 (C-11), 18.4 (C-21), 12.1 (C-18); anal. calcd for C_35_H_60_N_2_O_4_: C, 73.38; H, 10.56; found C, 73.29; H, 10.53. MALDI TOF: *m*/*z* 595.475 ([M + Na]^+^, calcd 595.445), 611.439 ([M + K]^+^, calcd 611.419).

tert-Butyl 4-(3,24-Dioxocholan-24-yl)piperazine-1-carboxylate (**20d**)

Colorless waxy solid, 0.53 g, 83% yield. [α]_D_^21^ + 16.6 (c 0.86, CHCl_3_); IR (KBr) ν_max_ 2932, 2865, 1699, 1647, 1455, 1419, 1365, 1285, 1253, 1168, 1129, 1026, 997, 754, 665 cm^−1^; ^1^H NMR (CDCl_3_, 500 MHz) δ 3.60–3.55 (2H, m, CH_2_N), 3.48–3.40 (6H, m, CH_2_N), 2.73‒1.06 (28H, m), 1.48 (9H, s, CH_3_), 1.02 (3H, s, H-19), 0.95 (3H, d, J = 6.5 Hz, H-21), 0.69 (3H, s, H-18); ^13^C NMR (CDCl_3_, 125 MHz) δ 213.4 (C-3), 172.3 (C-24), 154.6 (COO), 80.3 (C(CH_3_)_3_), 56.4 (C-14), 56.1 (C-17), 45.5 (CH_2_N), 44.3 (C-5), 42.8 (C-13), 42.4 (C-4), 41.3 (CH_2_N), 40.7 (C-9), 40.1 (C-12), 37.2 (C-2), 37.0 (C-1), 35.6 (C-8), 35.5 (C-20), 34.9 (C-10), 31.4 (C-22), 30.4 (C-23), 28.4 (CH_3_), 28.3 (C-16), 26.6 (C-6), 25.8 (C-7), 24.2 (C-15), 22.7 (C-19), 21.2 (C-11), 18.5 (C-21), 12.1 (C-18); anal. calcd for C_33_H_54_N_2_O_4_: C, 73.02; H, 10.03; found C, 72.93; H, 9.99. MALDI TOF: *m*/*z* 565.379 ([M + Na]^+^, calcd 565.398), 581.346 ([M + K]^+^, calcd 581.372).

#### 3.9.8. General Procedure for Removing BOC-Protected Groups

Trifluoroacetic acid (3.4 mL) was added to a solution of **19a**–**d** or **20a**–**d** (1.0 mmol) in CHCl_3_ (20 mL), and the mixture was stirred at room temperature for 1 h. The solvent was then evaporated under reduced pressure, and the residue was purified by chromatography on silica gel using CHCl_3_/MeOH (10:1) as the mobile phase, affording the product **21a**–**d** or **22a**–**d**.

(3α,5β)-24-[(2-Aminoethyl)amino]-24-oxocholan-3-yl Acetate (**21a**)

White crystals, 0.45 g, 98% yield. MP 34–36 °С, [α]_D_^19^ + 23.9 (c 0.69, CHCl_3_); IR (KBr) ν_max_ 2931, 2868, 1681, 1541, 1449, 1383, 1364, 1247, 1203, 1181, 1138, 1028, 981, 837, 799, 756, 666, 615 cm^−1^; ^1^H NMR (CDCl_3_, 500 MHz) δ 8.11 (2H, br s, NH_2_), 7.55 (1H, br s, NH), 4.73–7.68 (1H, m, H-3), 3.56–3.46 (2H, m, CH_2_NH), 3.15–3.06 (2H, m, CH_2_NH_2_), 2.27‒1.02 (28H, m), 2.01 (3H, s, CH_3_CO), 0.92 (3H, s, H-19), 0.91 (3H, d, J = 6.5 Hz, H-21), 0.63 (3H, s, H-18); ^13^C NMR (CDCl_3_, 125 MHz) δ 176.6 (C-24), 170.7 (CH_3_CO), 74.4 (C-3), 56.4 (C-14), 56.1 (C-17), 42.7 (C-13), 41.8 (C-5), 40.4 (C-9), 40.1 (C-12), 40.0 (CH_2_NH_2_), 37.3 (CH_2_NH), 35.8 (C-8), 35.6 (C-20), 35.0 (C-1), 34.6 (C-10), 33.2 (C-23), 32.2 (C-4), 31.6 (C-22), 28.2 (C-16), 27.0 (C-6), 26.6 (C-2), 26.3 (C-7), 24.2 (C-15), 23.3 (C-19), 21.4 (CH_3_CO), 20.8 (C-11), 18.2 (C-21), 11.9 (C-18); anal. calcd for C_28_H_48_N_2_O_3_: C, 73.00; H, 10.50; found C, 72.95; H, 10.46.

(3α,5β)-24-[(4-Aminobutyl)amino]-24-oxocholan-3-yl Acetate (**21b**)

White solid, 0.48 g, 98% yield. [α]_D_^17^ + 18.0 (c 0.89, CHCl_3_); IR (KBr) ν_max_ 2937, 2868, 1678, 1647, 1550, 1448, 1381, 1363, 1245, 1203, 1179, 1135, 1028, 836, 799, 755, 722, 665 cm^−1^; ^1^H NMR (CDCl_3_, 500 MHz) δ 7.97 (2H, br s, NH_2_), 6.77 (1H, br s, NH), 4.73–4.68 (1H, m, H-3), 3.23–3.17 (2H, m, CH_2_NH), 2.99–2.93 (2H, m, CH_2_NH_2_), 2.25‒1.00 (28H, m), 2.02 (3H, s, CH_3_CO), 1.72–1.66 (2H, m, CH_2_), 1.58–1.52 (2H, m, CH_2_), 0.93 (3H, s, H-19), 0.90 (3H, d, J = 6.5 Hz, H-21), 0.64 (3H, s, H-18); ^13^C NMR (CDCl_3_, 125 MHz) δ 174.9 (C-24), 170.6 (CH_3_CO), 74.4 (C-3), 56.5 (C-14), 56.1 (C-17), 42.7 (C-13), 41.9 (C-5), 40.4 (C-9), 40.2 (C-12), 39.4 (CH_2_NH_2_), 38.6 (CH_2_NH), 35.8 (C-8), 35.6 (C-20), 35.0 (C-1), 34.6 (C-10), 33.4 (C-23), 32.3 (C-4), 31.6 (C-22), 28.2 (C-16), 27.0 (C-6), 26.6 (C-2), 26.4 (C-7), 26.2 (CH_2_), 24.4 (CH_2_), 24.2 (C-15), 23.3 (C-19), 21.4 (CH_3_CO), 20.8 (C-11), 18.3 (C-21), 12.0 (C-18); anal. calcd for C_30_H_52_N_2_O_3_: C, 73.72; H, 10.72; found C, 73.67; H, 10.69.

(3α,5β)-24-[(6-Aminohexyl)amino]-24-oxocholan-3-yl Acetate (**21c**)

White solid, 0.50 g, 97% yield. [α]_D_^24^ + 17.3 (c 0.96, CHCl_3_); IR (KBr) ν_max_ 2937, 2866, 1680, 1644, 1548, 1448, 1380, 1363, 1244, 1202, 1179, 1137, 1041, 979, 933, 909, 835, 799, 755, 722, 667, 549 cm^−1^; ^1^H NMR (CDCl_3_, 500 MHz) δ 7.97 (2H, br s, NH_2_), 6.41 (1H, br s, NH), 4.73–4.68 (1H, m, H-3), 3.18–3.15 (2H, m, CH_2_NH), 2.95–2.90 (2H, m, CH_2_NH_2_), 2.26‒0.96 (28H, m), 2.02 (3H, s, CH_3_CO), 1.70–1.65 (2H, m, CH_2_), 1.50–1.45 (2H, m, CH_2_), 1.36–1.29 (4H, m, CH_2_), 0.93 (3H, s, H-19), 0.91 (3H, d, J = 6.5 Hz, H-21), 0.64 (3H, s, H-18); ^13^C NMR (CDCl_3_, 125 MHz) δ 174.5 (C-24), 170.6 (CH_3_CO), 74.4 (C-3), 56.5 (C-14), 56.1 (C-17), 42.7 (C-13), 41.9 (C-5), 40.4 (C-9), 40.2 (C-12), 39.7 (CH_2_NH_2_), 39.2 (CH_2_NH), 35.8 (C-8), 35.6 (C-20), 35.0 (C-1), 34.6 (C-10), 33.5 (C-23), 32.2 (C-4), 31.9 (C-22), 28.9 (CH_2_), 28.2 (C-16), 27.0 (C-6, CH_2_), 26.6 (C-2), 26.3 (C-7), 25.9 (CH_2_), 25.6 (CH_2_), 24.2 (C-15), 23.3 (C-19), 21.5 (CH_3_CO), 20.8 (C-11), 18.3 (C-21), 12.0 (C-18); anal. calcd for C_32_H_56_N_2_O_3_: C, 74.37; H, 10.92; found C, 74.32; H, 10.89.

(3α,5β)-24-Oxo-24-piperazin-1-ylcholan-3-yl Acetate (**21d**)

White solid; 0.47 g, 97% yield. [α]_D_^24^ + 20.5 (c 0.99, CHCl_3_); IR (KBr) ν_max_ 2938, 2867, 1727, 1674, 1447 1431, 1383, 1363, 1246, 1202, 1180, 1134, 1028, 833, 799, 755, 722, 666, 564 cm^−1^; ^1^H NMR (CDCl_3_, 500 MHz) δ 10.02 (1H, br s, NH), 4.71–4.67 (1H, m, H-3), 3.88–3.77 (4H, m, CH_2_N), 3.22–3.17 (4H, m, CH_2_NH), 2.38‒0.98 (28H, m), 2.00 (3H, s, CH_3_CO), 0.91 (3H, s, H-19), 0.91 (3H, d, J = 6.5 Hz, H-21), 0.63 (3H, s, H-18); ^13^C NMR (CDCl_3_, 125 MHz) δ 172.3 (C-24), 170.6 (CH_3_CO), 74.4 (C-3), 56.4 (C-14), 55.9 (C-17), 43.4 (CH_2_NH), 42.7 (C-13), 42.3 (CH_2_N), 41.8 (C-5), 40.4 (C-9), 40.1 (C-12), 38.2 (CH_2_N), 35.8 (C-8), 35.5 (C-20), 35.0 (C-1), 34.5 (C-10), 32.2 (C-4), 31.1 (C-22), 29.9 (C-23), 28.3 (C-16), 26.9 (C-6), 26.6 (C-2), 26.3 (C-7), 24.2 (C-15), 23.3 (C-19), 21.4 (CH_3_CO), 20.8 (C-11), 18.4 (C-21), 12.0 (C-18); anal. calcd for C_30_H_50_N_2_O_3_: C, 74.03; H, 10.35; found C, 73.96; H, 10.32.

(5β)-*N*-(2-Aminoethyl)-3-oxocholan-24-amide (**22a**)

Colorless waxy solid, 0.41 g, 98% yield. [α]_D_^19^ + 17.0 (c 0.88, CHCl_3_); IR (KBr) ν_max_ 2927, 2866, 1678, 1547, 1454, 1379, 1339, 1256, 1202, 1181, 1136, 836, 799, 755 cm^−1^; ^1^H NMR (CDCl_3_, 500 MHz) δ 8.27 (2H, br s, NH_2_), 7.67 (1H, br s, NH), 3.54–3.48 (2H, m, CH_2_NH), 3.15–3.10 (2H, m, CH_2_NH_2_), 2.71‒1.04 (28H, m), 1.00 (3H, s, H-19), 0.90 (3H, d, J = 6.5 Hz, H-21), 0.67 (3H, s, H-18); ^13^C NMR (CDCl_3_, 125 MHz) δ 212.1 (C-3), 175.7 (C-24), 56.3 (C-14), 56.0 (C-17), 44.3 (C-5), 42.7 (C-13), 42.2 (C-4), 40.6 (C-9), 40.0 (C-12, CH_2_NH_2_), 37.2 (C-2), 36.9 (C-1, CH_2_NH), 35.5 (C-8, C-20), 34.8 (C-10), 32.9 (C-23), 31.5 (C-22), 28.1 (C-16), 26.6 (C-6), 25.7 (C-7), 24.1 (C-15), 22.4 (C-19), 21.1 (C-11), 18.1 (C-21), 11.8 (C-18); anal. calcd for C_26_H_44_N_2_O_2_: C, 74.95; H, 10.64; found C, 74.89; H, 10.60.

(5β)-*N*-(4-Aminobutyl)-3-oxocholan-24-amide (**22b**)

Colorless waxy solid, 0.43 g, 97% yield. [α]_D_^14^ + 11.0 (c 0.98, CHCl_3_); IR (KBr) ν_max_ 2936, 2867, 1682, 1646, 1557, 1542, 1456, 1379, 1339, 1268, 1202, 1179, 1134, 835, 799, 753, 722 cm^−1^; ^1^H NMR (CDCl_3_, 500 MHz) δ 8.14 (2H, br s, NH_2_), 6.79 (1H, br s, NH), 3.23–3.20 (2H, m, CH_2_NH), 2.99–2.95 (2H, m, CH_2_NH_2_), 2.73‒1.05 (28H, m), 1.73–1.69 (2H, m, CH_2_), 1.59–1.55 (2H, m, CH_2_), 1.03 (3H, s, H-19), 0.92 (3H, d, J = 6.5 Hz, H-21), 0.68 (3H, s, H-18); ^13^C NMR (CDCl_3_, 125 MHz) δ 213.6 (C-3), 174.9 (C-24), 56.4 (C-14), 55.9 (C-17), 44.2 (C-5), 42.8 (C-13), 42.3 (C-4), 40.7 (C-9), 40.0 (C-12), 39.4 (CH_2_NH_2_), 38.5 (CH_2_NH), 37.2 (C-2), 36.9 (C-1), 35.5 (C-8, C-20), 34.9 (C-10), 33.2 (C-23), 31.8 (C-22), 28.2 (C-16), 26.6 (C-6), 26.2 (CH_2_), 25.8 (C-7), 24.5 (CH_2_), 24.2 (C-15), 22.6 (C-19), 21.2 (C-11), 18.3 (C-21), 12.1 (C-18); anal. calcd for C_28_H_48_N_2_O_2_: C, 75.63; H, 10.88; found C, 75.57; H, 10.83. MALDI TOF: *m*/*z* 445.377 ([M + H]^+^, calcd 445.379).

(5β)-*N*-(6-Aminohexyl)-3-oxocholan-24-amide (**22c**)

Colorless waxy solid, 0.45 g, 97% yield. [α]_D_^21^ + 15.5 (c 0.82, CHCl_3_); IR (KBr) ν_max_ 2935, 2865, 1682, 1638, 1557, 1542, 1456, 1380, 1268, 1202, 1180, 1135, 835, 799, 755, 722 cm^−1^; ^1^H NMR (CDCl_3_, 500 MHz) δ 8.07 (2H, br s, NH_2_), 6.42 (1H, br s, NH), 3.19–3.16 (2H, m, CH_2_NH), 2.95–2.91 (2H, m, CH_2_NH_2_), 2.70‒1.05 (28H, m), 1.69–1.65 (2H, m, CH_2_), 1.49–1.46 (2H, m, CH_2_), 1.37–1.31 (4H, m, CH_2_), 1.03 (3H, s, H-19), 0.93 (3H, d, J = 6.5 Hz, H-21), 0.69 (3H, s, H-18); ^13^C NMR (CDCl_3_, 125 MHz) δ 213.6 (C-3), 174.4 (C-24), 56.4 (C-14), 55.9 (C-17), 44.3 (C-5), 42.8 (C-13), 42.3 (C-4), 40.7 (C-9), 40.1 (C-12), 39.6 (CH_2_NH_2_), 39.1 (CH_2_NH), 37.2 (C-2), 36.9 (C-1), 35.5 (C-8, C-20), 34.9 (C-10), 33.4 (C-23), 31.9 (C-22), 29.0 (CH_2_), 28.2 (C-16), 27.1 (CH_2_), 26.6 (C-6), 25.9 (CH_2_), 25.8 (C-7), 25.6 (CH_2_), 24.1 (C-15), 22.6 (C-19), 21.2 (C-11), 18.3 (C-21), 12.1 (C-18); anal. calcd for C_30_H_52_N_2_O_2_: C, 76.22; H, 11.09; found C, 76.17; H, 11.05. MALDI TOF: *m*/*z* 511.362 ([M + K]^+^, calcd 511.367).

(5β)-24-Oxo-24-piperazin-1-ylcholan-3-one (**22d**)

Colorless waxy solid, 0.42 g, 96% yield. [α]_D_^17^ +10.0 (c 0.89, CHCl_3_); IR (KBr) ν_max_ 2934, 2866, 2498, 1681, 1443 1378, 1203, 1181, 1135, 1026, 835, 800, 753, 721, 667 cm^−1^; ^1^H NMR (CDCl_3_, 500 MHz) δ 9.59 (1H, br s, NH), 3.91–3.77 (4H, m, CH_2_N), 3.28–3.22 (4H, m, CH_2_NH), 2.71‒1.04 (28H, m), 1.02 (3H, s, H-19), 0.93 (3H, d, J = 6.5 Hz, H-21), 0.68 (3H, s, H-18); ^13^C NMR (CDCl_3_, 125 MHz) δ 213.8 (C-3), 172.8 (C-24), 56.4 (C-14), 55.8 (C-17), 44.2 (C-5), 43.5 (CH_2_NH), 42.8 (C-13), 42.3 (C-4), 42.3, 38.4 (CH_2_N), 40.7 (C-9), 40.0 (C-12), 37.2 (C-2), 36.9 (C-1), 35.5 (C-8, C-20), 34.8 (C-10), 31.0 (C-22), 29.8 (C-23), 28.2 (C-16), 26.6 (C-6), 25.7 (C-7), 24.1 (C-15), 22.6 (C-19), 21.2 (C-11), 18.4 (C-21), 12.0 (C-18); anal. calcd for C_28_H_46_N_2_O_2_: C, 75.97; H, 10.47; found C, 75.95; H, 10.46. MALDI TOF: *m*/*z* 465.341 ([M + Na]^+^, calcd 465.346), 481.301 ([M + Na]^+^, calcd 481.319).

#### 3.9.9. Reaction of Diaminoalkane Derivatives of 3a-Acetoxy-5β-cholan-24-oic Acid and 3-Oxo-cholan-24-oic Acid **21a**–**d**, **22a**–**d** with (5*Z*,9*Z*)-Tetradeca-5,9-dienedioic Acid (**5**)

Lithocholic acid derivative **23a**–**d** and **24a**–**d** was synthesized according to a modified literature procedure [41]. To a solution of diaminoalkane derivatives of 3α-acetoxy-5β-cholan-24-oic acid **21a**–**d** or 3-oxo-cholan-24-oic acid **22a**–**d** (1.0 mmol) in CH_2_Cl_2_ (50 mL), (5*Z*,9*Z*)-tetradeca-5,9-dienedioic acid (**5**) (0.51 g, 2.0 mmol) was added, followed by EDC·HCl (0.48 g, 2.5 mmol) and DMAP (18 mg, 0.15 mmol) under argon. The mixture was stirred at room temperature for 12 h until the reaction was complete (TLC monitoring, hexane/ethyl acetate). The mixture was diluted with H_2_O (10 mL) and the CH_2_Cl_2_ layer was separated, dried over MgSO_4_, and concentrated. The crude product was purified by column chromatography (silica gel) using CHCl_3_/MeOH (10:1) as the elution solvent to afford compounds **23a**–**d** or **24a**–**d**.

(5Z,9Z)-14-[(2-{[(3α,5β)-3-(Acetyloxy)-24-oxocholan-24-yl]amino}ethyl)amino]-14-oxotetradeca-5,9-dienoic Acid (**23a**)

Colorless waxy solid, 0.47 g, 68% yield. [α]_D_^19^ + 16.1 (c 0.76, CHCl_3_); IR (KBr) ν_max_ 2929, 2866, 1735, 1647, 1546, 1449, 1379, 1363, 1243, 1065, 1028, 756, 666, 608 cm^−1^; ^1^H NMR (CDCl_3_, 500 MHz) δ 7.08 (1H, br s, NH), 6.85 (1H, br s, NH), 5.43–5.33 (4H, m, H-5’, H-6’, H-9’, H-10’), 4.73–4.68 (1H, m, H-3), 3.39–3.33 (4H, m, CH_2_NH), 2.37‒1.00 (28H, m), 2.37–2.34 (2H, m, H-2’), 2.23–2.19 (2H, m, H-13’), 2.10–2.04 (8H, m, H-4’, H-7’, H-8’, H-11’), 2.03 (3H, s, CH_3_CO), 1.73–1.66 (4H, m, H-3’, H-12’), 0.93 (3H, s, H-19), 0.92 (3H, d, J = 6.5 Hz, H-21), 0.64 (3H, s, H-18); ^13^C NMR (CDCl_3_, 125 MHz) δ 176.2 (C-1’), 175.1 (C-24), 174.5 (C-14’), 170.7 (CH_3_CO), 130.2, 130.1 (C-6’, C-9’), 129.1 (C-5’, C-10’), 74.4 (C-3), 56.5 (C-14), 56.0 (C-17), 42.7 (C-13), 41.9 (C-5), 40.4 (C-9), 40.1 (C-12), 39.9, 39.7 (CH_2_NH), 35.9 (C-13’), 35.8 (C-8), 35.5 (C-20), 35.0 (C-1), 34.6 (C-10), 33.5 (C-23), 33.1 (C-2’), 32.2 (C-4), 31.8 (C-22), 28.2 (C-16), 27.7, 27.5 (C-7’, C-8’), 27.3 (C-11’), 27.0 (C-6), 26.7 (C-4’), 26.6 (C-2), 26.3 (C-7), 25.6 (C-12’), 24.7 (C-3’), 24.2 (C-15), 23.3 (C-19), 21.4 (CH_3_CO), 20.8 (C-11), 18.4 (C-21), 12.1 (C-18); anal. calcd for C_42_H_68_N_2_O_6_: C, 72.37; H, 9.83; found C, 72.30; H, 9.79; MALDI TOF: *m*/*z* 719.428 ([M + Na]^+^, calcd 719.498), 735.508 ([M + K]^+^, calcd 735.471).

(5Z,9Z)-14-[(4-{[(3α,5β)-3-(Acetyloxy)-24-oxocholan-24-yl]amino}butyl)amino]-14-oxotetradeca-5,9-dienoic Acid (**23b**)

Colorless waxy solid, 0.49 g, 68% yield. [α]_D_^19^ + 13.5 (c 0.37, CHCl_3_); IR (KBr) ν_max_ 2932, 2864, 1734, 1647, 1541, 1456, 1383, 1363, 1243, 1165, 1029, 800, 753 cm^−1^; ^1^H NMR (CDCl_3_, 500 MHz) δ 6.49 (1H, br s, NH), 6.11 (1H, br s, NH), 5.47–5.35 (4H, m, H-5’, H-6’, H-9’, H-10’), 4.73–4.68 (1H, m, H-3), 3.30–3.25 (4H, m, CH_2_NH), 2.36 (2H, t, J = 7.2 Hz, H-2’), 2.30‒1.00 (28H, m), 2.24 (2H, t, J = 7.2 Hz, H-13’), 2.11–2.05 (8H, m, H-4’, H-7’, H-8’, H-11’), 2.04 (3H, s, CH_3_CO), 1.74–1.66 (4H, m, H-3’, H-12’), 1.57–1.51 (4H, m, CH_2_), 0.94 (3H, s, H-19), 0.92 (3H, d, J = 6.8 Hz, H-21), 0.65 (3H, s, H-18); ^13^C NMR (CDCl_3_, 125 MHz) δ 176.1 (C-1’), 174.6 (C-24), 174.3 (C-14’), 170.7 (CH_3_CO), 130.3, 130.1 (C-6’, C-9’), 129.2 (C-5’, C-10’), 74.4 (C-3), 56.5 (C-14), 56.1 (C-17), 42.7 (C-13), 41.9 (C-5), 40.4 (C-9), 40.2 (C-12), 39.9 (С-25, С-28), 36.1 (C-13’), 35.8 (C-8), 35.5 (C-20), 35.0 (C-1), 34.6 (C-10), 33.6 (C-23), 33.3 (C-2’), 32.3 (C-4), 31.9 (C-22), 28.2 (C-16), 27.6, 27.5 (C-7’, C-8’), 27.0 (C-6), 26.9, 26.8 (C-4’, C-11’), C-26, C-27), 26.6 (C-2), 26.3 (C-7), 25.9 (C-12’), 24.8 (C-3’), 24.2 (C-15), 23.3 (C-19), 21.4 (CH_3_CO), 20.8 (C-11), 18.4 (C-21), 12.1 (C-18); anal. calcd for C_44_H_72_N_2_O_6_: C, 72.89; H, 10.01; found C, 72.88; H, 9.98; MALDI TOF: *m*/*z* 747.440 ([M + Na]^+^, calcd 747.529), 763.422 ([M + K]^+^, calcd 763.503).

(5Z,9Z)-14-[(4-{[(3α,5β)-3-(Acetyloxy)-24-oxocholan-24-yl]amino}hexyl)amino]-14-oxotetradeca-5,9-dienoic Acid (**23c**)

Colorless waxy solid, 0.48 g, 65% yield. [α]_D_^19^ + 15.3 (c 0.95, CHCl_3_); IR (KBr) ν_max_ 2933, 2864, 1734, 1646, 1549, 1455, 1379, 1363, 1243, 1163, 1111, 1046, 979, 933, 909, 729, 668, 549 cm^−1^; ^1^H NMR (CDCl_3_, 500 MHz) δ 6.36 (1H, br s, NH), 6.12 (1H, br s, NH), 5.40–5.32 (4H, m, H-5’, H-6’, H-9’, H-10’), 4.72–4.68 (1H, m, H-3), 3.20 (4H, q, J = 6.5 Hz, CH_2_NH), 2.31 (2H, t, J = 7.5 Hz, H-2’), 2.27‒1.00 (28H, m), 2.19 (2H, J = 7.5 Hz, H-13’), 2.11–2.03 (8H, m, H-4’, H-7’, H-8’, H-11’), 2.00 (3H, s, CH_3_CO), 1.69–1.64 (4H, m, H-3’, H-12’), 1.49–1.45 (4H, m, CH_2_), 1.33–1.28 (4H, m, CH_2_), 0.90 (3H, s, H-19), 0.89 (3H, d, J = 6.8 Hz, H-21), 0.61 (3H, s, H-18); ^13^C NMR (CDCl_3_, 125 MHz) δ 176.5 (C-1’), 174.3 (C-24), 173.9 (C-14’), 170.7 (CH_3_CO), 130.3, 130.2 (C-6’, C-9’), 129.1 (C-5’, C-10’), 74.4 (C-3), 56.5 (C-14), 56.0 (C-17), 42.7 (C-13), 41.9 (C-5), 40.4 (C-9), 40.1 (C-12), 39.1, 39.0 (CH_2_NH), 36.1 (C-13’), 35.8 (C-8), 35.5 (C-20), 35.0 (C-1), 34.6 (C-10), 33.6 (C-23), 33.5 (C-2’), 32.2 (C-4), 31.9 (C-22), 29.3, 29.2 (CH_2_), 28.2 (C-16), 27.5, 27.4 (C-7’, C-8’), 26.9 (C-6), 26.8 (C-11’), 26.6 (C-2), 26.4 (C-4’), 26.3 (C-7), 25.9 (C-12’, CH_2_), 24.8 (C-3’), 24.2 (C-15), 23.3 (C-19), 21.4 (CH_3_CO), 20.8 (C-11), 18.4 (C-21), 12.0 (C-18); anal. calcd for C_46_H_76_N_2_O_6_: C, 73.36; H, 10.17; found C, 73.30; H, 10.12; MALDI TOF: *m*/*z* 775.579 ([M + Na]^+^, calcd 775.560), 791.556 ([M + K]^+^, calcd 791.534).

(5Z,9Z)-14-{4-[(3α,5β)-3-(Acetyloxy)-24-oxocholan-24-yl]piperazin-1-yl}-14-oxotetradeca-5,9-dienoic Acid (**23d**)

Colorless waxy solid, 0.46 g, 64% yield. [α]_D_^19^ + 18.0 (c 0.94, CHCl_3_); IR (KBr) ν_max_ 2935, 2866, 1732, 1647, 1432, 1379, 1363, 1243, 1179, 1026, 983, 888, 753, 666, 615 cm^−1^; ^1^H NMR (CDCl_3_, 500 MHz) δ 5.40–5.30 (4H, m, H-5’, H-6’, H-9’, H-10’), 4.67 (1H, m, H-3), 3.62–3.56 (4H, m, CH_2_N), 3.47–3.43 (4H, m, CH_2_N), 2.33–2.28 (4H, m, H-2’, H-13’), 2.38‒0.96 (28H, m), 2.10–2.01 (8H, m, H-4’, H-7’, H-8’, H-11’), 1.99 (3H, s, CH_3_CO), 1.68–1.64 (4H, m, H-3’, H-12’), 0.90 (3H, d, J = 6.8 Hz, H-21), 0.89 (3H, s, H-19), 0.61 (3H, s, H-18); ^13^C NMR (CDCl_3_, 125 MHz) δ 176.8 (C-1’), 172.8 (C-24), 172.4 (C-14’), 170.7 (CH_3_CO), 130.3, 130.1 (C-6’, C-9’), 129.1, 128.9 (C-5’, C-10’), 74.4 (C-3), 56.4 (C-14), 55.9 (C-17), 45.6, 45.4 (CH_2_N), 42.7 (C-13), 41.8 (C-5), 41.6, 41.3 (CH_2_N), 40.4 (C-9), 40.1 (C-12), 35.7 (C-8), 35.6 (C-20), 34.9 (C-1), 34.5 (C-10), 33.3 (C-13’), 32.6 (C-2’), 32.3 (C-4), 31.3 (C-22), 30.2 (C-23), 28.3 (C-16), 27.4, 27.3 (C-7’, C-8’), 26.9 (C-6), 26.7 (C-4’), 26.6 (C-2), 26.4 (C-11’), 26.3 (C-7), 25.2 (C-3’), 24.7 (C-12’), 24.2 (C-15), 23.3 (C-19), 21.4 (CH_3_CO), 20.8 (C-11), 18.5 (C-21), 12.0 (C-18); anal. calcd for C_44_H_70_N_2_O_6_: C, 69.34; H, 9.26; found C, 69.29; H, 9.20; MALDI TOF: *m*/*z* 745.544 ([M + Na]^+^, calcd 745.513), 761.515 ([M + K]^+^, calcd 761.487).

(5Z,9Z)-14-[(2-{[(5β)-3,24-Dioxocholan-24-yl]amino}ethyl)amino]-14-oxotetradeca-5,9-dienoic Acid (**24a**)

Colorless waxy solid, 0.44 g, 67% yield. [α]_D_^16^ + 11.9 (c 0.91, CHCl_3_); IR (KBr) ν_max_ 2929, 2865, 1713, 1651, 1550, 1455, 1379, 1257, 1165, 1150, 1076, 1023, 754, 665 cm^−1^; ^1^H NMR (CDCl_3_, 500 MHz) δ 7.04 (1H, br s, NH), 6.85 (1H, br s, NH), 5.44–5.30 (4H, m, H-5’, H-6’, H-9’, H-10’), 3.38–3.34 (4H, m, CH_2_NH), 2.70‒1.05 (28H, m), 2.38–2.34 (2H, m, H-2’), 2.22–2.18 (2H, m, H-13’), 2.10–2.04 (8H, m, H-4’, H-7’, H-8’, H-11’), 1.70–1.64 (4H, m, H-3’, H-12’), 1.01 (3H, s, H-19), 0.91 (3H, d, J = 6.5 Hz, H-21), 0.67 (3H, s, H-18); ^13^C NMR (CDCl_3_, 125 MHz) δ 213.6 (C-3), 176.3 (C-1’), 175.6 (C-24), 175.0 (C-14’), 130.2, 130.1 (C-6’, C-9’), 129.2, 129.0 (C-5’, C-10’), 56.4 (C-14), 55.9 (C-17), 44.3 (C-5), 42.7 (C-13), 42.4 (C-4), 40.7 (C-9), 40.0 (C-12), 39.7, 39.6 (CH_2_NH), 37.2 (C-2), 36.9(C-1), 35.9 (C-13’), 35.5 (C-8, C-20), 34.9 (C-10), 33.4 (C-23), 33.2 (C-2’), 31.8 (C-22), 28.2 (C-16), 27.6, 27.5 (C-7’, C-8’), 26.7 (C-11’), 26.6 (C-6), 26.4 (C-4’), 25.8 (C-7), 25.7 (C-12’), 24.7 (C-3’), 24.2 (C-15), 22.6 (C-19), 21.2 (C-11), 18.4 (C-21), 12.1 (C-18); anal. calcd for C_40_H_64_N_2_O_5_: C, 73.58; H, 9.88; found C, 73.54; H, 9.84; MALDI TOF: *m*/*z* 675.307 ([M + Na]^+^, calcd 675.482), 691.276 ([M + K]^+^, calcd 691.482).

(5Z,9Z)-14-[(4-{[(5β)-3,24-Dioxocholan-24-yl]amino}butyl)amino]-14-oxotetradeca-5,9-dienoic Acid (**24b**)

Colorless waxy solid, 0.45 g, 66% yield. [α]_D_^17^ + 15.0 (c 0.77, CHCl_3_); IR (KBr) ν_max_ 2932, 2865, 1712, 1645, 1551, 1446, 1379, 1230, 1181, 1149, 1077, 1022, 754, 666 cm^−1^; ^1^H NMR (CDCl_3_, 500 MHz) δ 6.54 (1H, br s, NH), 6.25 (1H, br s, NH), 5.42–5.33 (4H, m, H-5’, H-6’, H-9’, H-10’), 3.28–3.24 (4H, m, CH_2_NH), 2.72‒1.05 (28H, m), 2.37–2.33 (2H, m, H-2’), 2.22–2.18 (2H, m, H-13’), 2.11–2.04 (8H, m, H-4’, H-7’, H-8’, H-11’), 1.71–1.66 (4H, m, H-3’, H-12’), 1.55–1.51 (4H, m, CH_2_), 1.02 (3H, s, H-19), 0.93 (3H, d, J = 6.5 Hz, H-21), 0.68 (3H, s, H-18); ^13^C NMR (CDCl_3_, 125 MHz) δ 213.6 (C-3), 176.2 (C-1’), 174.5 (C-24), 174.2 (C-14’), 130.3, 130.1 (C-6’, C-9’), 129.2 (C-5’, C-10’), 56.4 (C-14), 56.0 (C-17), 44.3 (C-5), 42.8 (C-13), 42.4 (C-4), 40.7 (C-9), 40.1 (C-12), 39.1, 39.0 (CH_2_NH), 37.2 (C-2), 36.9 (C-1), 36.1 (C-13’), 35.5 (C-8, C-20), 34.9 (C-10), 33.6 (C-23), 33.4 (C-2’), 31.9 (C-22), 28.2 (C-16), 27.6, 27.5 (C-7’, C-8’), 26.9, 26.8 (CH_2_), 26.7 (C-11’), 26.6 (C-6), 26.4 (C-4’), 25.9 (C-12’), 25.8 (C-7), 24.7 (C-3’), 24.2 (C-15), 22.6 (C-19), 21.2 (C-11), 18.4 (C-21), 12.1 (C-18); anal. calcd for C_42_H_68_N_2_O_5_: C, 74.07; H, 10.06; found C, 74.01; H, 10.02; MALDI TOF: *m*/*z* 681.472 ([M + H]^+^, calcd 681.521), 703.444 ([M + Na]^+^, calcd 703.503), 719.407 ([M + K]^+^, calcd 719.477).

(5Z,9Z)-14-[(6-{[(5β)-3,24-Dioxocholan-24-yl]amino}hexyl)amino]-14-oxotetradeca-5,9-dienoic Acid (**24c**)

Colorless waxy solid, 0.45 g, 64% yield. [α]_D_^23^ + 11.0 (c 0.75, CHCl_3_); IR (KBr) ν_max_ 2929, 2861, 1713, 1645, 1551, 1454, 1378, 1260, 1181, 1149, 1103, 1077, 1022, 991, 801, 755 cm^−1^; ^1^H NMR (CDCl_3_, 500 MHz) δ 6.16 (1H, br s, NH), 5.88 (1H, br s, NH), 5.47–5.34 (4H, m, H-5’,H-6’, H-9’, H-10’), 3.28–3.24 (4H, m, CH_2_NH), 2.73‒1.05 (28H, m), 2.38–2.33 (2H, m, H-2’), 2.25–2.21 (2H, m, H-13’), 2.12–2.05 (8H, m, H-4’, H-7’, H-8’, H-11’), 1.74–1.67 (4H, m, H-3’, H-12’), 1.53–1.48 (4H, m, CH_2_), 1.38–1.33 (4H, m, CH_2_), 1.03 (3H, s, H-19), 0.94 (3H, d, J = 6.5 Hz, H-21), 0.69 (3H, s, H-18); ^13^C NMR (CDCl_3_, 125 MHz) δ 213.6 (C-3), 176.5 (C-1’), 174.2 (C-14’, C-24), 130.2, 130.1 (C-6’, C-9’), 129.1 (C-5’, C-10’), 56.4 (C-14), 56.0 (C-17), 44.3 (C-5), 42.8 (C-13), 42.4 (C-4), 40.7 (C-9), 40.1 (C-12), 39.1, 38.9 (CH_2_NH), 37.2 (C-2), 37.0 (C-1), 36.2 (C-13’), 35.5 (C-8, C-20), 34.9 (C-10), 33.6 (C-23), 33.4 (C-2’), 31.9 (C-22), 29.3, 29.1 (CH_2_), 28.2 (C-16), 27.6, 27.5 (C-7’, C-8’), 26.8 (C-11’), 26.6 (C-6), 26.4 (C-4’), 26.0, 25.8, (C-7, C-12’, CH_2_), 24.8 (C-3’), 24.2 (C-15), 22.7 (C-19), 21.2 (C-11), 18.4 (C-21), 12.1 (C-18); anal. calcd for C_44_H_72_N_2_O_5_: C, 74.53; H, 10.24; found C, 74.48; H, 10.19; MALDI TOF: *m*/*z* 709.589 ([M + H]^+^, calcd 709.552), 731.571 ([M + Na]^+^, calcd 731.534), 747.541 ([M + K]^+^, calcd 747.508).

(5Z,9Z)-{4-[(5β)-3,24-Dioxocholan-24-yl]piperazin-1-yl}-14-oxotetradeca-5,9-dienoic Acid (**24d**)

Colorless waxy solid, 0.43 g, 63% yield. [α]_D_^18^ + 13.1 (c 0.95, CHCl_3_); IR (KBr) ν_max_ 2931, 2864, 1715, 1647, 1434, 1363, 1245, 1221, 1180, 1077, 1019, 802, 753 cm^−1^; ^1^H NMR (CDCl_3_, 500 MHz) δ 5.44–5.31 (4H, m, H-5’, H-6’, H-9’, H-10’), 3.66–3.60 (4H, m, CH_2_N), 3.52–3.45 (4H, m, CH_2_N), 2.40‒0.96 (28H, m), 2.39–2.33 (4H, m, H-2’, H-13’), 2.14–2.03 (8H, m, H-4’, H-7’, H-8’, H-11’), 1.72–1.66 (4H, m, H-3’, H-12’), 1.02 (3H, s, H-19), 0.95 (3H, d, J = 6.8 Hz, H-21), 0.69 (3H, s, H-18); ^13^C NMR (CDCl_3_, 125 MHz) δ 213.6 (C-3), 176.7 (C-1’), 172.6 (C-14’, C-24), 130.4, 130.2 (C-6’, C-9’), 129.1, 128.9 (C-5’, C-10’), 56.4 (C-14), 55.9 (C-17), 45.6, 45.4 (CH_2_N), 44.3 (C-5), 42.8 (C-13), 42.3 (C-4), 41.7, 41.6 (CH_2_N), 40.4 (C-9), 40.0 (C-12), 37.2 (C-2), 36.9 (C-1), 35.6 (C-8), 35.5 (C-20), 34.9 (C-10), 33.2 (C-2’), 32.6 (C-13’), 31.3 (C-22), 30.3 (C-23), 28.3 (C-16), 27.5, 27.4 (C-7’, C-8’), 26.8 (C-4’), 26.6 (C-6), 26.4 (C-11’), 25.8 (C-7), 25.3 (C-3’), 24.7 (C-12’), 24.2 (C-15), 22.6 (C-19), 21.2 (C-11), 18.5 (C-21), 12.1 (C-18); anal. calcd for C_42_H_66_N_2_O_5_: C, 74.29; H, 9.80; found C, 74.23; H, 9.77; MALDI TOF: *m*/*z* 679.503 ([M + H]^+^, calcd 679.497), 701.477 ([M + Na]^+^, calcd 701.497), 717.443 ([M + K]^+^, calcd 717.497).

## 4. Conclusions

In summary, for the first time, we synthesized hybrid molecules being synthetic analogs of natural 5*Z*,9*Z*-dienoic acids on the basis of lithocholic acid and (5*Z*,9*Z*)-1,14-tetradeca-5,9-dienedicarboxylic acid obtained in two stages using the homo-cyclomagnesiation reaction of 2-(hepta-5,6-diene-1-yloxy)tetrahydro-2H-pyran at the key stage. It was shown that all the synthesized hybrid molecules exhibited high cytotoxicity against a panel of five tumor cell lines (Jurkat, K562, HEK293, HeLa, and U937) as compared with the original lithocholic acid and also, possibly, to a small extent affected topoisomerase II, initiated mitochondrial apoptosis in Jurkat tumor cells, thus causing loss of cytochrome C and activation of caspases. The studied derivatives of lithocholic acid induced G1/S cell cycle arrest, which indicates their high antitumor activity and allows considering these compounds as promising drug candidates.

## Figures and Tables

**Scheme 4:**
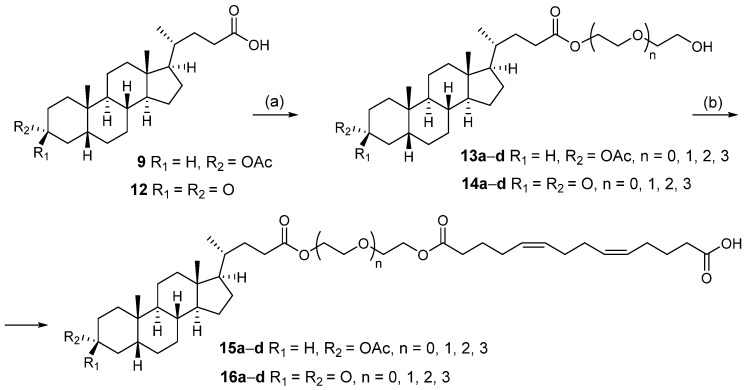
Synthesis of the LA-fatty acid conjugates linked via ethylene glycol units. (a) Ethylene glycols of different lengths, EDCl·HCl, DMAP, CH_2_Cl_2_. (b) (5*Z*,9*Z*)-Tetradeca-5,9-dienedioic acid 5, EDCl·HCl, DMAP, CH_2_Cl_2_.

**Table 1:** Cytotoxic activity of compounds **8**, **11**, **15a**–**d**, **16a**–**d**, **23a**–**d**, and **24a**–**d** (48 h).

Compound	Jurkat, IC_50_, µМ	K562, IC_50_, µМ	U937, IC_50_, µМ	HEK293, IC_50_, µМ	HeLa, IC_50_, µМ	Fibroblasts, IC_50_, µМ	hTopI inhibition, µМ
**6**	1.06 ± 0.11	1.49 ± 0.14	1.71 ± 0.16	1.84 ± 0.17	1.79 ± 0.18	3.96 ± 0.34	2
**8**	0.13 ± 0.01	0.56 ± 0.04	0.78 ± 0.06	0.95 ± 0.08	0.92 ± 0.07	0.63 ± 0.05	0.04
**11**	0.01 ± 0.00	0.16 ± 0.01	0.05 ± 0.00	0.21 ± 0.02	0.19 ± 0.02	0.05 ± 0.01	0.01
**15а**	0.14 ± 0.01	0.25 ± 0.02	0.17 ± 0.01	0.34 ± 0.03	0.31 ± 0.03	0.73 ± 0.06	0.04
**15b**	0.13 ± 0.01	0.23 ± 0.01	0.16 ± 0.02	0.29 ± 0.02	0.37 ± 0.02	0.69 ± 0.05	0.04
**15c**	0.06 ± 0.00	0.19 ± 0.02	0.17 ± 0.01	0.21 ± 0.02	0.25 ± 0.02	0.29 ± 0.02	0.02
**15d**	0.04 ± 0.00	0.11 ± 0.01	0.09 ± 0.01	0.14 ± 0.01	0.19 ± 0.02	0.21 ± 0.03	0.02
**16a**	0.42 ± 0.03	0.64 ± 0.04	0.51 ± 0.05	0.73 ± 0.06	0.78 ± 0.07	1.68 ± 0.15	1
**16b**	0.09 ± 0.01	0.14 ± 0.01	0.12 ± 0.01	0.16 ± 0.01	0.22 ± 0.02	0.64 ± 0.07	0.06
**16c**	0.12 ± 0.01	0.20 ± 0.02	0.16 ± 0.01	0.23 ± 0.02	0.25 ± 0.02	0.61 ± 0.06	0.04
**16d**	0.13 ± 0.01	0.21 ± 0.01	0.15 ± 0.01	0.26 ± 0.02	0.24 ± 0.02	0.77 ± 0.08	0.04
**23а**	0.32 ± 0.02	0.23 ± 0.01	0.34 ± 0.03	0.53 ± 0.04	0.41 ± 0.04	1.42 ± 0.15	0.08
**23b**	0.46 ± 0.04	0.19 ± 0.02	0.26 ± 0.02	0.54 ± 0.03	0.48 ± 0.05	2.11 ± 0.24	0.08
**23c**	0.21 ± 0.01	0.22 ± 0.02	0.25 ± 0.02	0.80 ± 0.07	0.38 ± 0.03	1.43 ± 0.12	0.06
**23d**	0.14 ± 0.01	0.09 ± 0.03	0.21 ± 0.03	0.98 ± 0.07	0.22 ± 0.01	0.81 ± 0.09	0.04
**24a**	0.14 ± 0.02	0.18 ± 0.03	0.24 ± 0.01	0.67 ± 0.05	0.55 ± 0.04	0.96 ± 0.08	0.04
**24b**	0.11 ± 0.02	0.09 ± 0.01	0.13 ± 0.01	0.31 ± 0.06	0.22 ± 0.04	0.68 ± 0.06	0.04
**24c**	0.08 ± 0.03	0.18 ± 0.06	0.28 ± 0.04	0.58 ± 0.05	0.38 ± 0.07	0.84 ± 0.05	0.04
**24d**	0.07 ± 0.01	0.09 ± 0.01	0.09 ± 0.01	0.44 ± 0.03	0.39 ± 0.03	0.69 ± 0.04	0.04
DMSO (1%)	No dead cells were observed						
Campt.	1.12 ± 0.12	1.54 ± 0.13	1.32 ± 0.12	3.18 ± 0.16	2.21 ± 0.25	7.86 ± 0.69	0.40

## Data Availability

The data presented in this study are available on request from the corresponding author.

## Supplementary Materials

The following are available online at https://www.mdpi.com/1424-8247/14/2/84/s1, ^1^H NMR and ^13^C NMR spectra of all compounds **8**, **11**, **13**–**16**, **21**–**24**.

## References

[1] Bukowski K., Kciuk M., Kontek R. (2020). Mechanisms of Multidrug Resistance in Cancer Chemotherapy. Int. J. Mol. Sci..

[2] Nooter K., Stoter G. (1996). Molecular mechanisms of multidrug resistance in cancer chemotherapy. Pathol. Res. Pract..

[3] Patel N.H., Rothenberg M.L. (1994). Multidrug resistance in cancer chemotherapy. Invest. New Drugs..

[4] Kikuchi H., Yuan B., Hu X., Okazaki M. (2019). Chemopreventive and anticancer activity of flavonoids and its possibility for clinical use by combining with conventional chemotherapeutic agents. Am. J. Cancer Res..

[5] Lichota A., Gwozdzinski K. (2018). Anticancer Activity of Natural Compounds from Plant and Marine Environment. Int. J. Mol. Sci..

[6] Ye Q., Liu K., Shen Q., Li Q., Hao J., Han F., Jiang R.-W. (2019). Reversal of Multidrug Resistance in Cancer by Multi-Functional Flavonoids. Front. Oncol..

[7] D’yakonov V.A., Dzhemileva L.U., Dzhemilev U.M. (2020). Natural Compounds with bis-Methylene-Interrupted Z-Double Bonds: Plant Sources, Strategies of Total Synthesis, Biological Activity, and Perspectives. Phytochem. Rev..

[8] Hofmann A.F., Hagey L.R. (2008). Bile acids: Chemistry, pathochemistry, biology, pathobiology, and therapeutics. Cell Mol. Life Sci..

[9] Dang Z., Lin A., Ho P., Soroka D., Lee K.-H., Huang L., Chen C.-H. (2011). Synthesis and proteasome inhibition of lithocholic acid derivatives. Bioorg. Med. Chem. Lett..

[10] Dang Z., Jung K., Qian K., Lee K.-H., Huang L., Chen C.-H. (2012). Synthesis of Lithocholic Acid Derivatives as Proteasome Regulators. ACS Med. Chem. Lett.

[11] Ishizawa M., Matsunawa M., Adachi R., Uno S., Ikeda K., Masuno H., Shimizu M., Iwasaki K., Yamada S., Makishima M. (2008). Lithocholic acid derivatives act as selective vitamin D receptor modulators without inducing hypercalcemia. J. Lipid Res..

[12] Adachi R., Honma Y., Masuno H., Kawana K., Shimomura I., Yamada S., Makishima M. (2005). Selective activation of vitamin D receptor by lithocholic acid acetate, a bile acid derivative. J. Lipid Res..

[13] Cheng J., Fang Z.Z., Kim J.H., Krausz K.W., Tanaka N., Chiang J.Y., Gonzalez F.J. (2014). Intestinal CYP3A4 protects against lithocholic acid-induced hepatotoxicity in intestine-specific VDR-deficient mice. J. Lipid Res..

[14] Mizushina Y., Kasai N., Miura K., Hanashima S., Takemura M., Yoshida H., Sugawara F., Sakaguchi K. (2004). Structural Relationship of Lithocholic Acid Derivatives Binding to the N-Terminal 8-kDa Domain of DNA Polymerase β. Biochemestry.

[15] do Nascimento P.G.G., Lemo T.L.G., Almeida M.C.S., de Souza J.M.O., Bizerra A.M.C., Santiago G.M.P., da Costa J.G.M., Coutinho H.D.M. (2015). Lithocholic acid and derivatives: Antibacterial activity. Steroids.

[16] Schneider H., Fiander H., Harrison K.A., Watson M., Burton G.W., Arya P. (1996). Inhibitory potency of lithocholic acid analogs and other bile acids on glucuronosyltransferase activity in a colon cancer cell line. Bioorg. Med. Chem. Lett..

[17] Vogel S.M., Bauer M.R., Joerger A.C., Wilcken R., Brandt T., Veprintsev D.B., Rutherford T.J., Fersht A.R., Boeckler F.M. (2012). Lithocholic acid is an endogenous inhibitor of MDM4 and MDM2. Proc. Natl. Acad. Sci. USA.

[18] Agarwal D.S., Anantaraju H.S., Sriram D., Yogeeswari P., Nanjegowda S.H., Mallu P., Sakhuja R. (2016). Synthesis, characterization and biological evaluation of bile acid-aromatic/heteroaromatic amides linked via amino acids as anti-cancer agents. Steroids.

[19] Bjedov S., Jakimov D., Pilipović A., Poša M., Sakač M. (2017). Antitumor activity of newly synthesized oxo and ethylidene derivatives of bile acids and their amides and oxazolines. Steroids.

[20] He X.-L., Xing Y., Gu X.-Z., Xiao J.-X., Wang Y.-Y., Yi Z., Qiu W.-W. (2017). The synthesis and antitumor activity of lithocholic acid and its derivatives. Steroids.

[21] Samadi M., Nury T., Khalafi-Nezhad A., Lizard G. (2017). Protecting group-free radical decarboxylation of bile acids: Synthesis of novel steroidal substituted maleic anhydrides and maleimides and evaluation of their cytotoxicity on C6 rat glioma cells. Steroids.

[22] Goldberg A.A., Beach A., Davies G.F., Harkness T.A., Leblanc A., Titorenko V.I. (2011). Lithocholic bile acid selectively kills neuroblastoma cells, while sparing normal neuronal cells. Oncotarget.

[23] D’yakonov V.A., Dzhemileva L.U., Tuktarova R.A., Makarov A.A., Islamov I.I., Mulyukova A.R., Dzhemilev U.M. (2015). Catalytic cyclometallation in steroid chemistry III: Synthesis of steroidal derivatives of 5Z,9Z-dienoic acid and their human topoisomerase I inhibitory activity. Steroids.

[24] D’yakonov V.A., Tuktarova R.A., Dzhemileva L.U., Ishmukhametova S.R., Yunusbaeva M.M., Ramazanov I.R., Dzhemilev U.M. (2017). Novel Hybrid Molecules on the Basis of Steroids and (5Z,9Z)-Tetradeca-5,9-diene-1,14-dioic Acid: Synthesis, Anti-Cancer Studies and Human Topoisomerase I Inhibitory Activity. Anti-Cancer Agents Med. Chem..

[25] D’yakonov V.A., Tuktarova R.A., Dzhemileva L.U., Ishmukhametova S.R., Yunusbaeva M.M., Dzhemilev U.M. (2018). Catalytic cyclometallation in steroid chemistry V: Synthesis of hybrid molecules based on steroid oximes and (5Z,9Z)-tetradeca-5,9-dienedioic acid as potential anticancer agents. Steroids.

[26] D’yakonov V.A., Tuktarova R.A., Dzhemileva L.U., Ishmukhametova S.R., Yunusbaeva M.M., Dzhemilev U.M. (2018). Catalytic cyclometallation in steroid chemistry VI: Targeted synthesis of hybrid molecules based on steroids and tetradeca-5Z,9Z-diene-1,14-dicarboxylic acid and study of their antitumor activity. Steroids.

[27] D’yakonov V.A., Makarov A.A., Andreev E.N., Dzhemileva L.U., Dzhemilev U.M. (2020). Catalytic cycloalumination of 1,2-dienes in the total synthesis of natural grenadamide and lyngbyoic acid. Russ. Chem. Bull..

[28] Mohammadkhani L., Heravi M.M. (2019). Oxalyl Chloride: A Versatile Reagent in Organic Transformations. ChemistrySelect.

[29] Green T.W., Wuts P.G.M. (1999). Protective Groups in Organic Synthesis.

[30] Freshney R.I. (2000). Culture of Animal Cells: A Manual of Basic Technique.

[31] Mitchell P. (1961). Coupling of phosphorylation to electron and hydrogen transfer by a chemi-osmotic type of mechanism. Nature.

[32] Lyamzaev K.G., Sumbatyan N.V., Nesterenko A.M., Kholina E.G., Voskoboynikova N., Steinhoff H.J., Mulkidjanian A.Y., Chernyak B.V. (2019). MitoCLox: A Novel Mitochondria-Targeted Fluorescent Probe for Tracing Lipid Peroxidation. Oxid Med. Cell. Longev..

[33] D’yakonov V.A., Makarov A.A., Dzhemileva L.U., Makarova E.Kh., Khusnutdinova E.K., Dzhemilev U.M. (2013). The facile synthesis of the 5Z,9Z-dienoic acids and their topoisomerase I inhibitory activity. Chem. Commun..

[34] D’yakonov V.A., Dzhemileva L.U., Makarov A.A., Mulyukova A.R., Baev D.S., Khusnutdinova E.K., Tolstikova T.G., Dzhemilev U.M. (2015). Stereoselective Synthesis of 11-Phenylundeca-5Z,9Z-dienoic Acid and Investigation of its Human Topoisomerase I and IIα Inhibitory Activity. Bioorg. Med. Chem. Lett..

[35] D’yakonov V.A., Dzhemileva L.U., Makarov A.A., Mulyukova A.R., Baev D.S., Khusnutdinova E.K., Tolstikova T.G., Dzhemilev U.M. (2016). nZ,(n+4)Z-Dienoic Fatty Acids: A New Method for the Synthesis and Inhibitory Action on Topoisomerase I and Iiα. Med. Chem. Res..

[36] D’yakonov V.А., Dzhemileva L.U., Dil’mukhametova L.К., Dzhemilev U.М. (2019). Synthesis of new Cu complex based on natural 5Z,9Z-eicosadienoic acid-effective topoisomerase I inhibitor and cytotoxin against the cisplatin-resistant cell line. ACS Omega.

[37] D’yakonov V.A., Dzhemileva L.U., Dzhemilev U.M. (2017). Advances in the Chemistry of Natural and Semisynthetic Topoisomerase I/II Inhibitors. Stud. Nat. Prod. Chem..

[38] Makarov A.A., Dzhemileva L.U., Salimova A.R., Makarova E.Kh., Ramazanov I.R., D’yakonov V.A., Dzhemilev U.M. (2020). New Synthetic Derivatives of Natural 5Z,9Z-Dienoic Acids: Stereoselective Synthesis and Study of the Antitumor Activity. Bioorg. Chem..

[39] Muslimovic A., Ismail I.H., Gao Y., Hammarsten O. (2008). An optimized method for measurement of gamma-H2AX in blood mononuclear and cultured cells. Nat. Protoc..

[40] Dzhemileva L.U., D’yakonov V.A., Islamov I.I., Yunusbaeva M.M., Dzhemilev U.M. (2020). New 1Z,5Z-diene macrodiolides: Catalytic synthesis, anticancer activity, induction of mitochondrial apoptosis, and effect on the cell cycle. Bioorg. Chem..

[41] Bildziukevich U., Vida N., Rárová L., Kolář M., Šaman D., Havlíček L., Drašar P., Wimmer Z. (2015). Polyamine derivatives of betulinic acid and β-sitosterol: A comparative investigation. Steroids.

