# Supplementary material for: Synthesis and Anticancer Activity of Hybrid Molecules Based on Lithocholic and (5Z,9Z)-Tetradeca-5,9-dienedioic Acids Linked via Mono(di,tri,tetra)ethylene Glycol and α,ω-Diaminoalkane Units
Source: Pharmaceuticals (Basel). 2021 Jan 23;14(2):84. doi: 10.3390/ph14020084 (PMC7911507; doi:10.3390/ph14020084)

Supplementary Materials

Synthesis and Anticancer Activity of Hybrid Molecules Based on Lithocholic and (5*Z*,9*Z*)-Tetradeca-5,9-dienedioic Acids Linked via Mono(di,tri,tetra)ethylene Glycol and α,ω-Diaminoalkane Units

Vladimir A. D'yakonov [1,*], Regina A. Tuktarova [1], Lilya U. Dzhemileva [1,*], Svetlana R. Ishmukhametova [1] and Usein M. Dzhemilev [1]

[1] Institute of Petrochemistry and Catalysis, Russian Academy of Sciences, pr. Oktyabrya 141, 450075 Ufa, Russia; regina-tuktarova@yandex.ru (R.A.T.); IshmukhametovaSR@gmail.com (S.R.I.); dzhemilev@anrb.ru (U.M.D.)

* Correspondence: DyakonovVA@gmail.com (V.A.D.), Dzhemilev@mail.ru (L.U.D.); Tel.: +7-347-284-2750 (V.A.D.)

Table of contents

Figure S1. ^{13}C NMR Spectrum of compound **4** 4
Figure S2. ^{1}H NMR Spectrum of compound **4** 4
Figure S3. ^{13}C NMR Spectrum of compound **5** 5
Figure S4. ^{1}H NMR Spectrum of compound **8** 5
Figure S5. ^{13}C NMR Spectrum of compound **10** 6
Figure S6. ^{1}H NMR Spectrum of compound **10** 6
Figure S7. ^{13}C NMR Spectrum of compound **11** 7
Figure S8. ^{1}H NMR Spectrum of compound **11** 7
Figure S9. ^{13}C NMR Spectrum of compound **13a** 8
Figure S10. ^{1}H NMR Spectrum of compound **13a** 8
Figure S11. ^{13}C NMR Spectrum of compound **13b** 9
Figure S12. ^{1}H NMR Spectrum of compound **13b** 9
Figure S13. ^{13}C NMR Spectrum of compound **13c** 10
Figure S14. ^{1}H NMR Spectrum of compound **13c** 10
Figure S15. ^{13}C NMR Spectrum of compound **13d** 11
Figure S16. ^{1}H NMR Spectrum of compound **13d** 11
Figure S17. ^{13}C NMR Spectrum of compound **14a** 12
Figure S18. ^{1}H NMR Spectrum of compound **14a** 12
Figure S19. ^{13}C NMR Spectrum of compound **14b** 13
Figure S20. ^{1}H NMR Spectrum of compound **14b** 13
Figure S21. ^{13}C NMR Spectrum of compound **14c** 14
Figure S22. ^{1}H NMR Spectrum of compound **14c** 14
Figure S23. ^{13}C NMR Spectrum of compound **14d** 15
Figure S24. ^{1}H NMR Spectrum of compound **14d** 15
Figure S25. ^{13}C NMR Spectrum of compound **15a** 16
Figure S26. ^{1}H NMR Spectrum of compound **15a** 16
Figure S27. ^{13}C NMR Spectrum of compound **15b** 17
Figure S28. ^{1}H NMR Spectrum of compound **15b** 17
Figure S29. ^{13}C NMR Spectrum of compound **15c** 18
Figure S30. ^{1}H NMR Spectrum of compound **15c** 18
Figure S31. ^{13}C NMR Spectrum of compound **15d** 19
Figure S32. ^{1}H NMR Spectrum of compound **15d** 19
Figure S33. ^{13}C NMR Spectrum of compound **16a** 20
Figure S34. ^{1}H NMR Spectrum of compound **16a** 20
Figure S35. ^{13}C NMR Spectrum of compound **16b** 21
Figure S36. ^{1}H NMR Spectrum of compound **16b** 21
Figure S37. ^{13}C NMR Spectrum of compound **16c** 22
Figure S38. ^{1}H NMR Spectrum of compound **16c** 22
Figure S39. ^{13}C NMR Spectrum of compound **16d** 23
Figure S40. ^{1}H NMR Spectrum of compound **16d** 23
Figure S41. ^{13}C NMR Spectrum of compound **19a** 24
Figure S42. ^{1}H NMR Spectrum of compound **19a** 24
Figure S43. ^{13}C NMR Spectrum of compound **19b** 25
Figure S44. ^{1}H NMR Spectrum of compound **19b** 25

Figure S45. ^{13}C NMR Spectrum of compound **19c** 26
Figure S46. ^{1}H NMR Spectrum of compound **19c** 26
Figure S47. ^{13}C NMR Spectrum of compound **19d** 27
Figure S48. ^{1}H NMR Spectrum of compound **19d** 27
Figure S49. ^{13}C NMR Spectrum of compound **20a** 28
Figure S50. ^{1}H NMR Spectrum of compound **20a** 28
Figure S51. ^{13}C NMR Spectrum of compound **20b** 29
Figure S52. ^{1}H NMR Spectrum of compound **20b** 29
Figure S53. ^{13}C NMR Spectrum of compound **20c** 30
Figure S54. ^{1}H NMR Spectrum of compound **20c** 30
Figure S55. ^{13}C NMR Spectrum of compound **20d** 31
Figure S56. ^{1}H NMR Spectrum of compound **20d** 31
Figure S57. ^{13}C NMR Spectrum of compound **21a** 32
Figure S58. ^{1}H NMR Spectrum of compound **21a** 32
Figure S59. ^{13}C NMR Spectrum of compound **21b** 33
Figure S60. ^{1}H NMR Spectrum of compound **21b** 33
Figure S61. ^{13}C NMR Spectrum of compound **21c** 34
Figure S62. ^{1}H NMR Spectrum of compound **21c** 34
Figure S63. ^{13}C NMR Spectrum of compound **21d** 35
Figure S64. ^{1}H NMR Spectrum of compound **21d** 35
Figure S65. ^{13}C NMR Spectrum of compound **22a** 36
Figure S66. ^{1}H NMR Spectrum of compound **22a** 36
Figure S67. ^{13}C NMR Spectrum of compound **22b** 37
Figure S68. ^{1}H NMR Spectrum of compound **22b** 37
Figure S69. ^{13}C NMR Spectrum of compound **22c** 38
Figure S70. ^{1}H NMR Spectrum of compound **22c** 38
Figure S71. ^{13}C NMR Spectrum of compound **22d** 39
Figure S72. ^{1}H NMR Spectrum of compound **22d** 39
Figure S73. ^{13}C NMR Spectrum of compound **23a** 40
Figure S74. ^{1}H NMR Spectrum of compound **23a** 40
Figure S75. ^{13}C NMR Spectrum of compound **23b** 41
Figure S76. ^{1}H NMR Spectrum of compound **23b** 41
Figure S77. ^{13}C NMR Spectrum of compound **23c** 42
Figure S78. ^{1}H NMR Spectrum of compound **23c** 42
Figure S79. ^{13}C NMR Spectrum of compound **23d** 43
Figure S80. ^{1}H NMR Spectrum of compound **23d** 43
Figure S81. ^{13}C NMR Spectrum of compound **24a** 44
Figure S82. ^{1}H NMR Spectrum of compound **24a** 44
Figure S83. ^{13}C NMR Spectrum of compound **24b** 45
Figure S84. ^{1}H NMR Spectrum of compound **24b** 45
Figure S85. ^{13}C NMR Spectrum of compound **24c** 46
Figure S86. ^{1}H NMR Spectrum of compound **24c** 46
Figure S87. ^{13}C NMR Spectrum of compound **24d** 47
Figure S88. ^{1}H NMR Spectrum of compound **24d** 47

Figure S1. ^{13}C NMR Spectrum of compound **4** (100 MHz, $CDCl_3$)

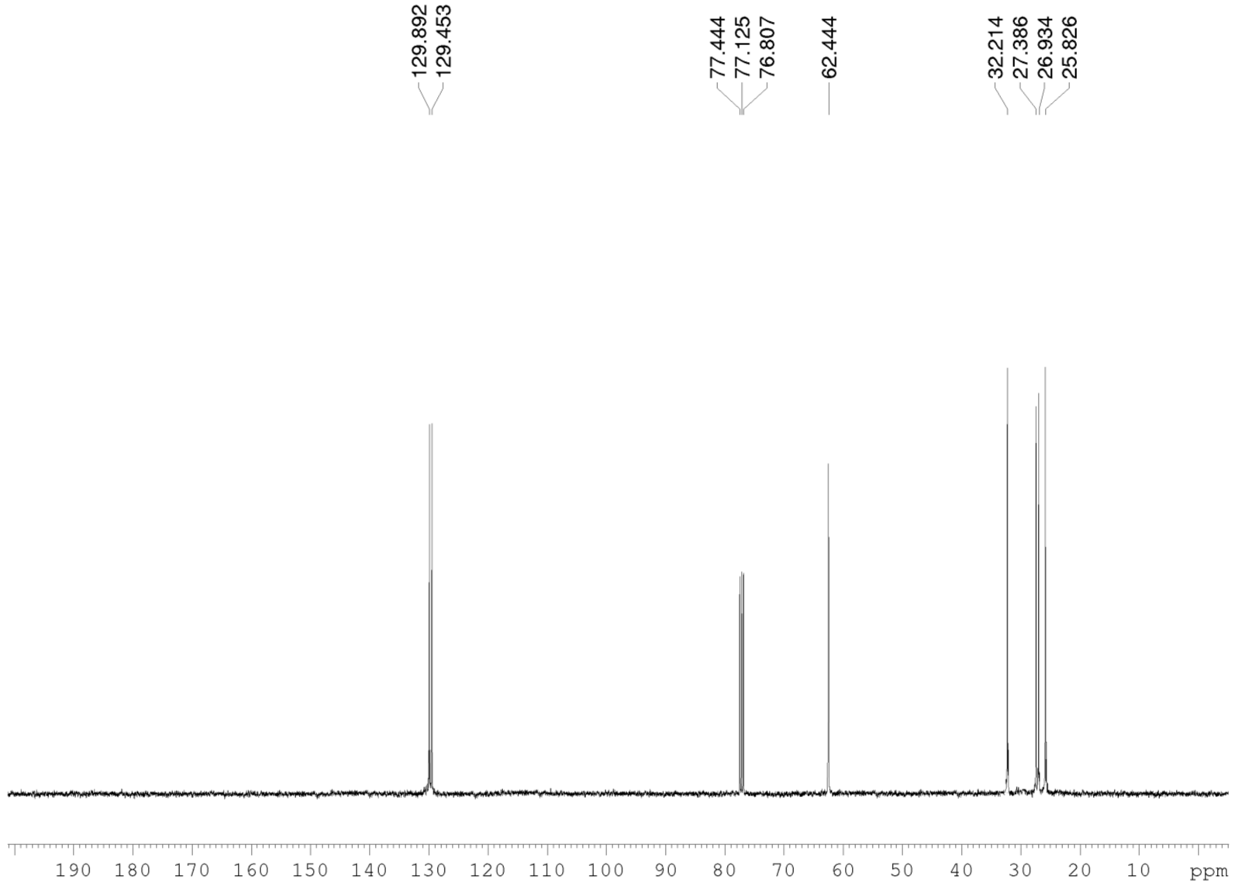

Figure S2. ^{1}H NMR Spectrum of compound **4** (400 MHz, $CDCl_3$)

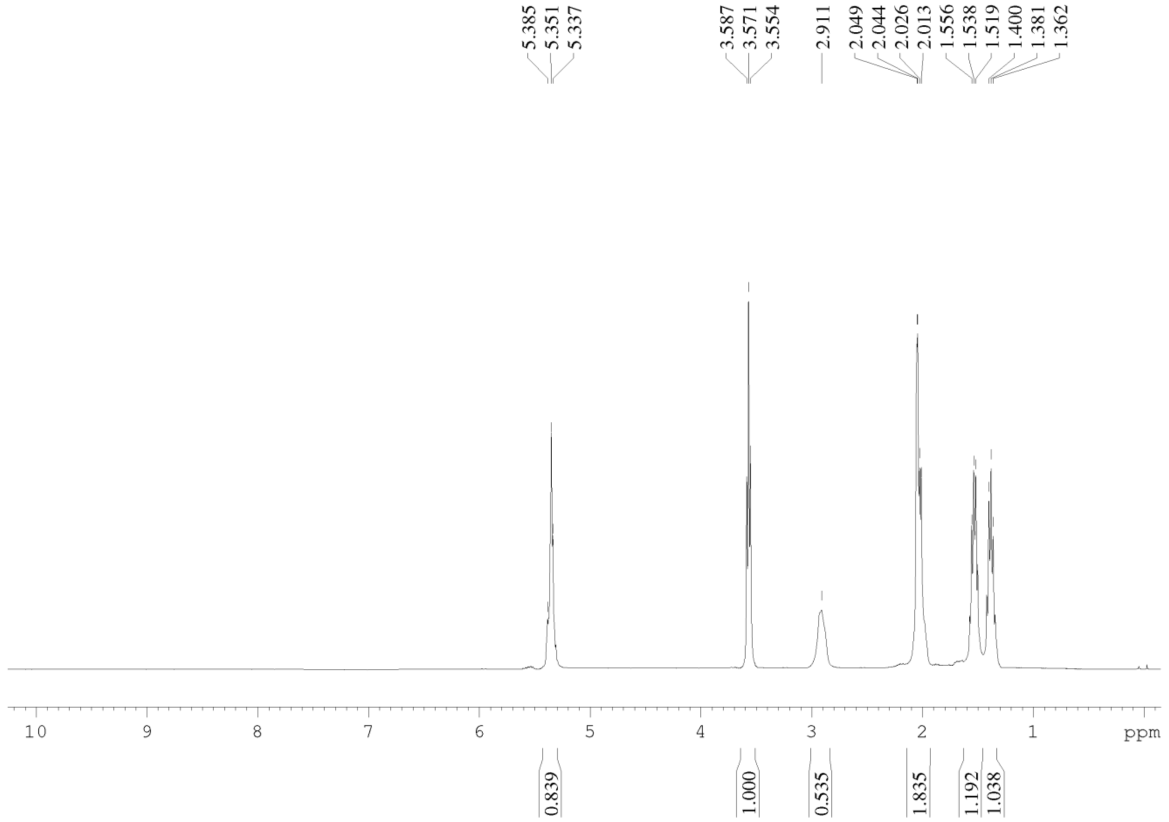

Figure S3. ^{13}C NMR Spectrum of compound **5** (125 MHz, $CDCl_3$)

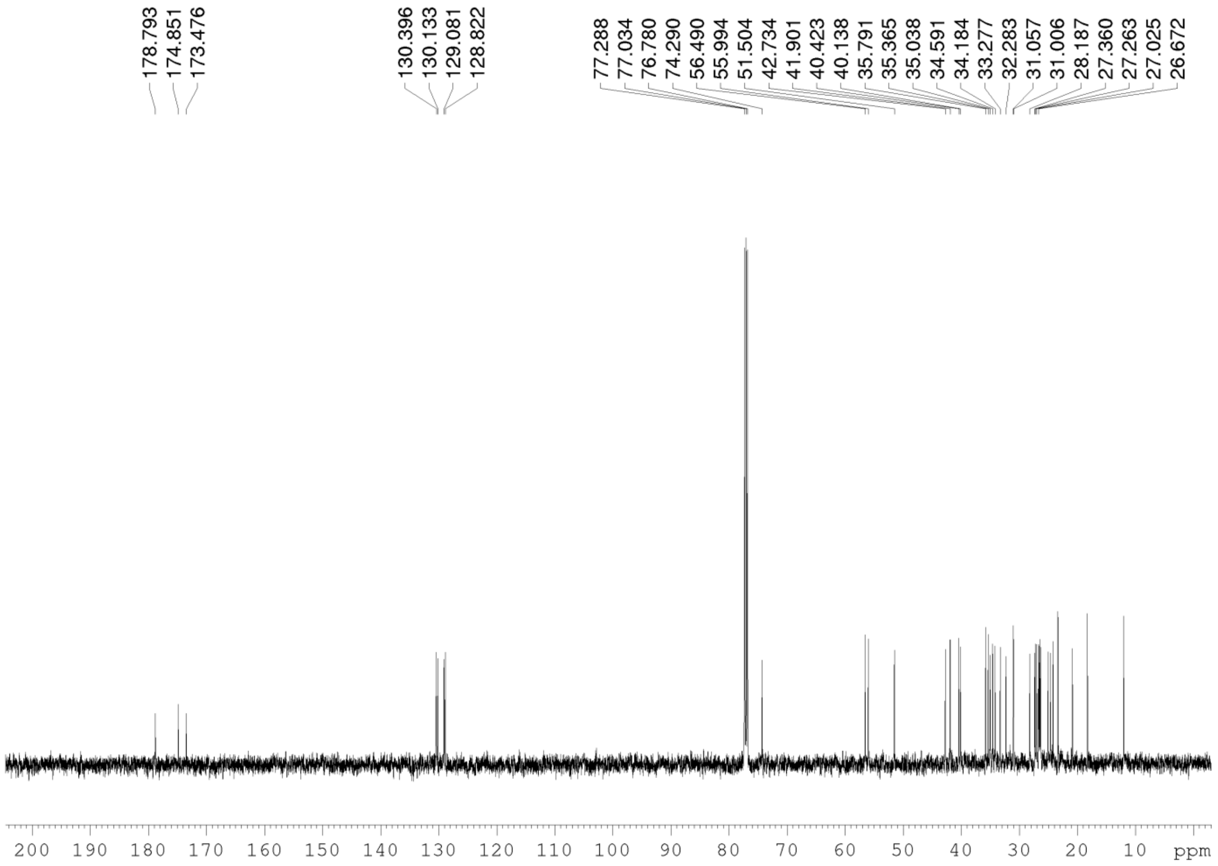

Figure S4. ^{1}H NMR Spectrum of compound **8** (500 MHz, $CDCl_3$)

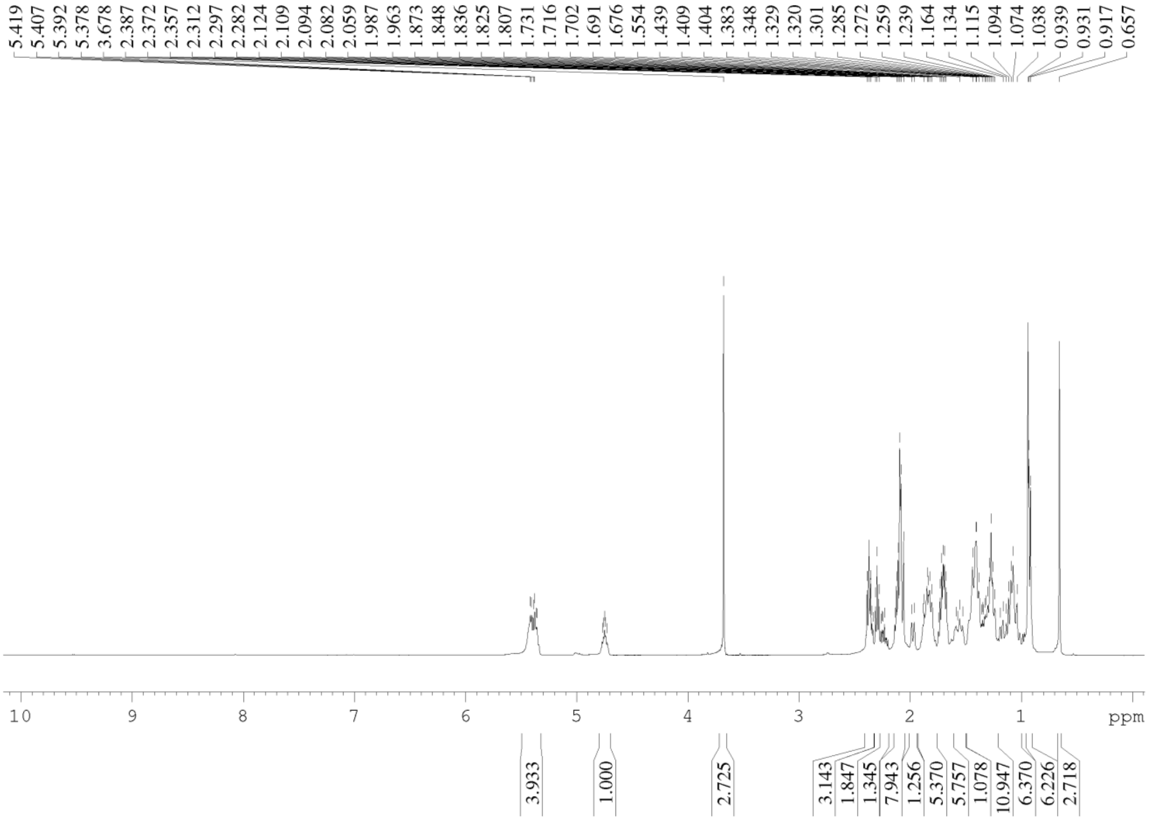

Figure S5. ^{13}C NMR Spectrum of compound **10** (125 MHz, $CDCl_3$)

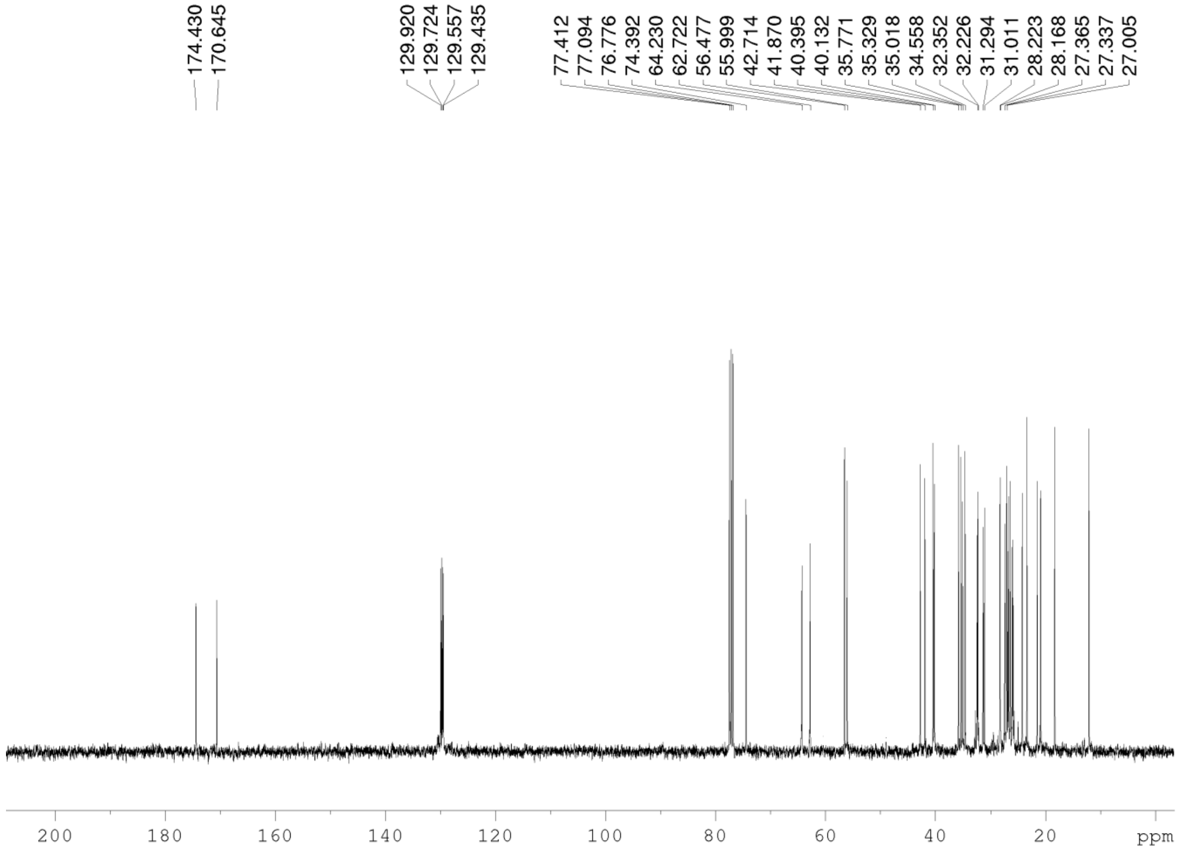

Figure S6. ^{1}H NMR Spectrum of compound **10** (500 MHz, $CDCl_3$)

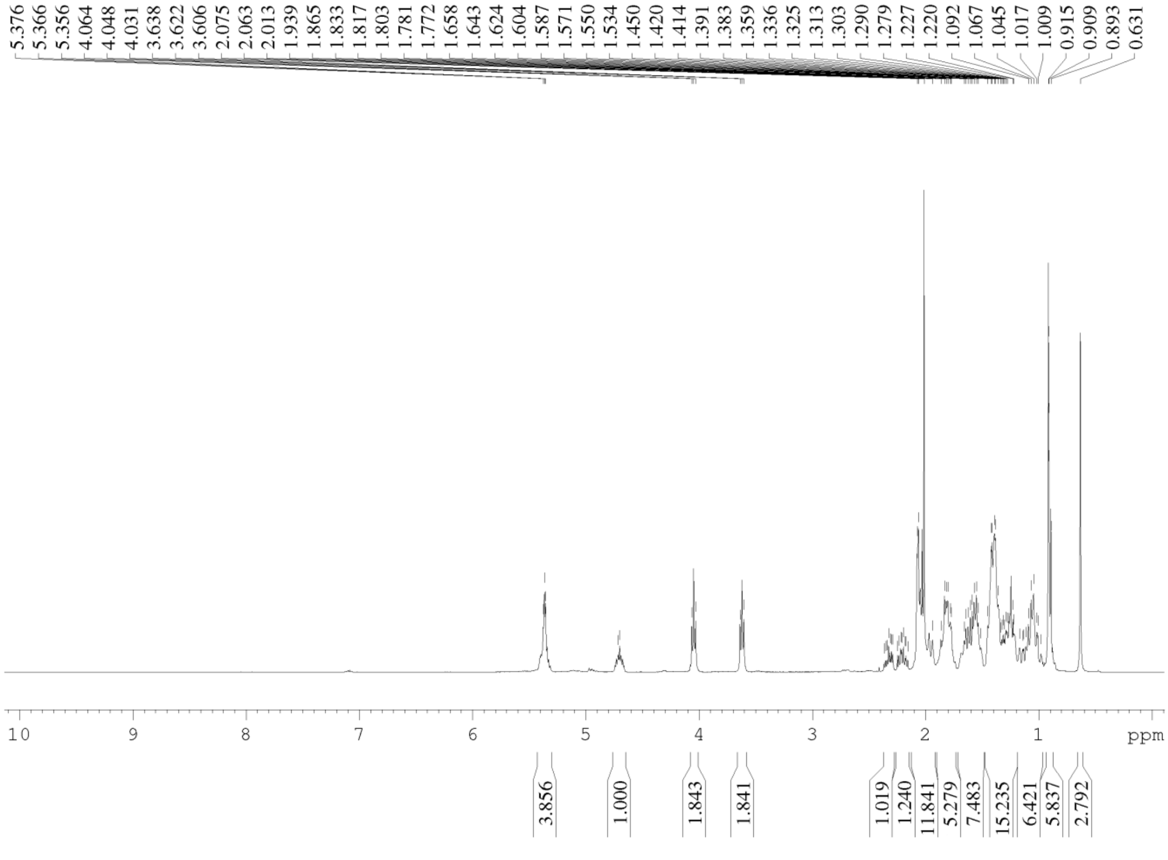

Figure S7. ^{13}C NMR Spectrum of compound **11** (125 MHz, $CDCl_3$)

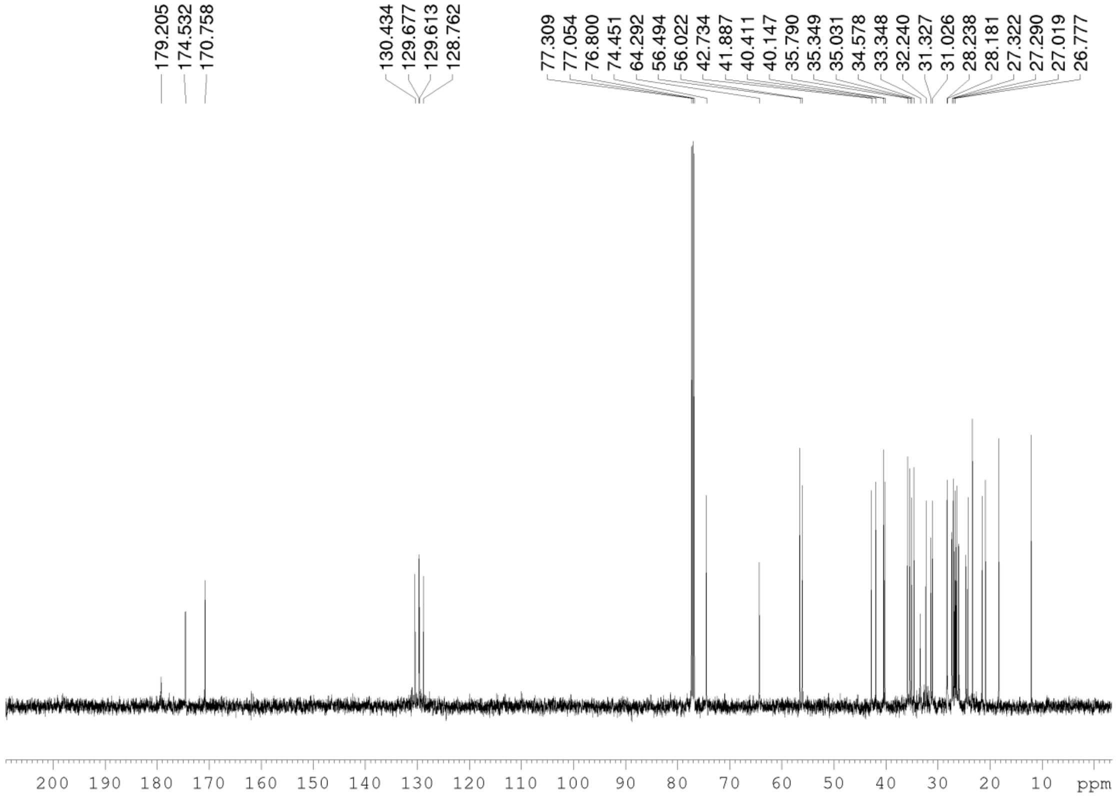

Figure S8. ^{1}H NMR Spectrum of compound **11** (500 MHz, $CDCl_3$)

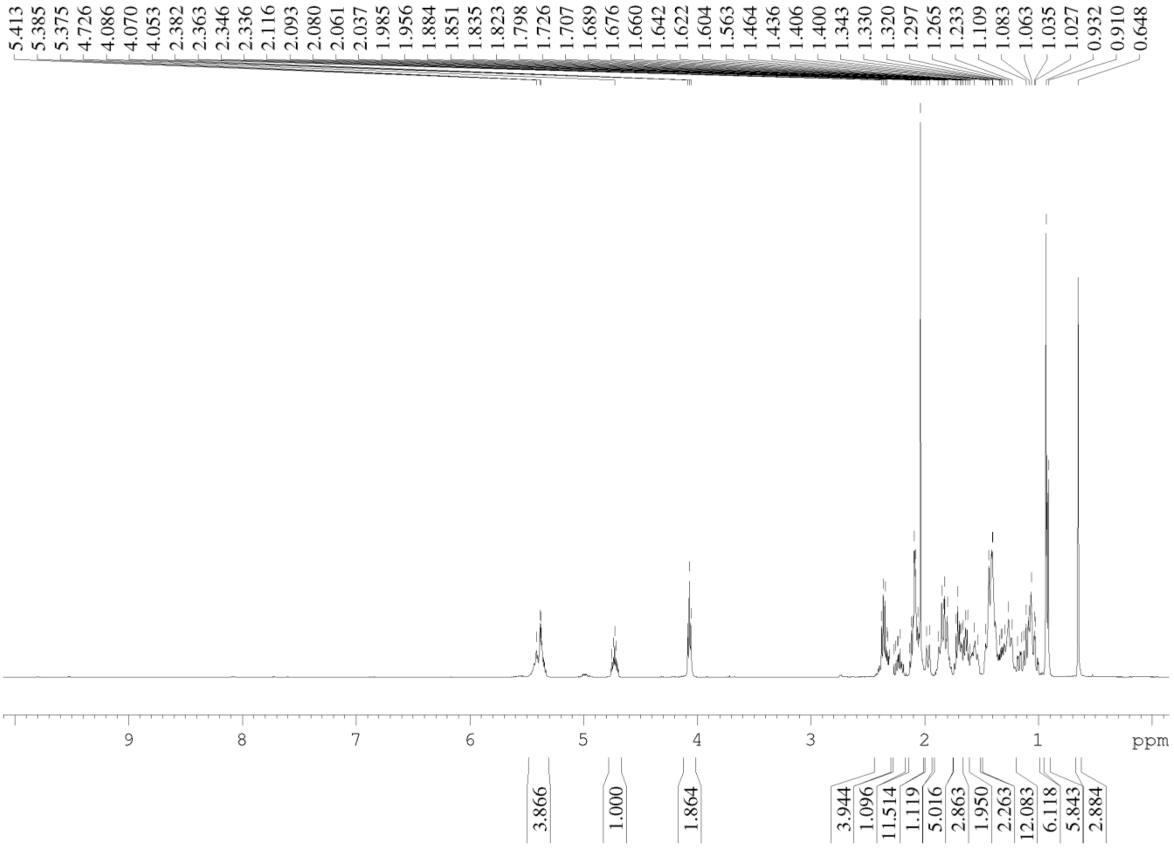

Figure S9. ^{13}C NMR Spectrum of compound **13a** (125 MHz, $CDCl_3$)

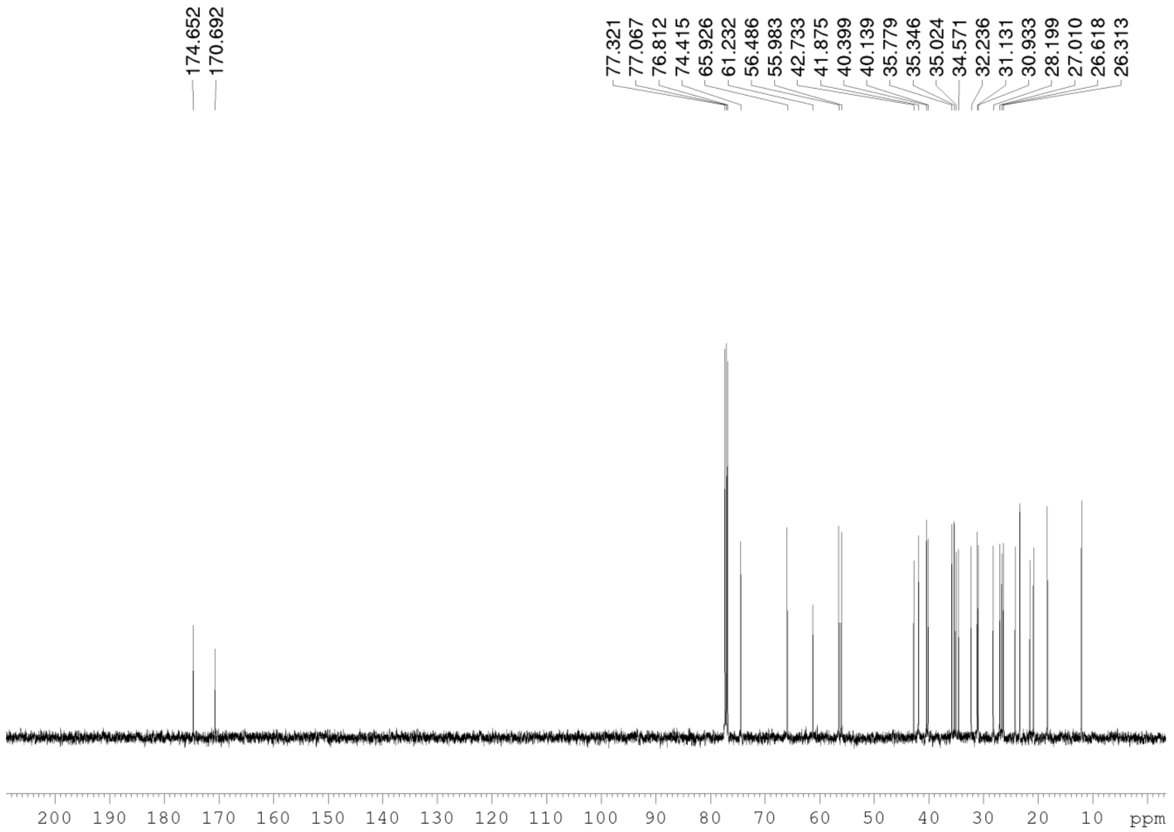

Figure S10. ^{1}H NMR Spectrum of compound **13a** (500 MHz, $CDCl_3$)

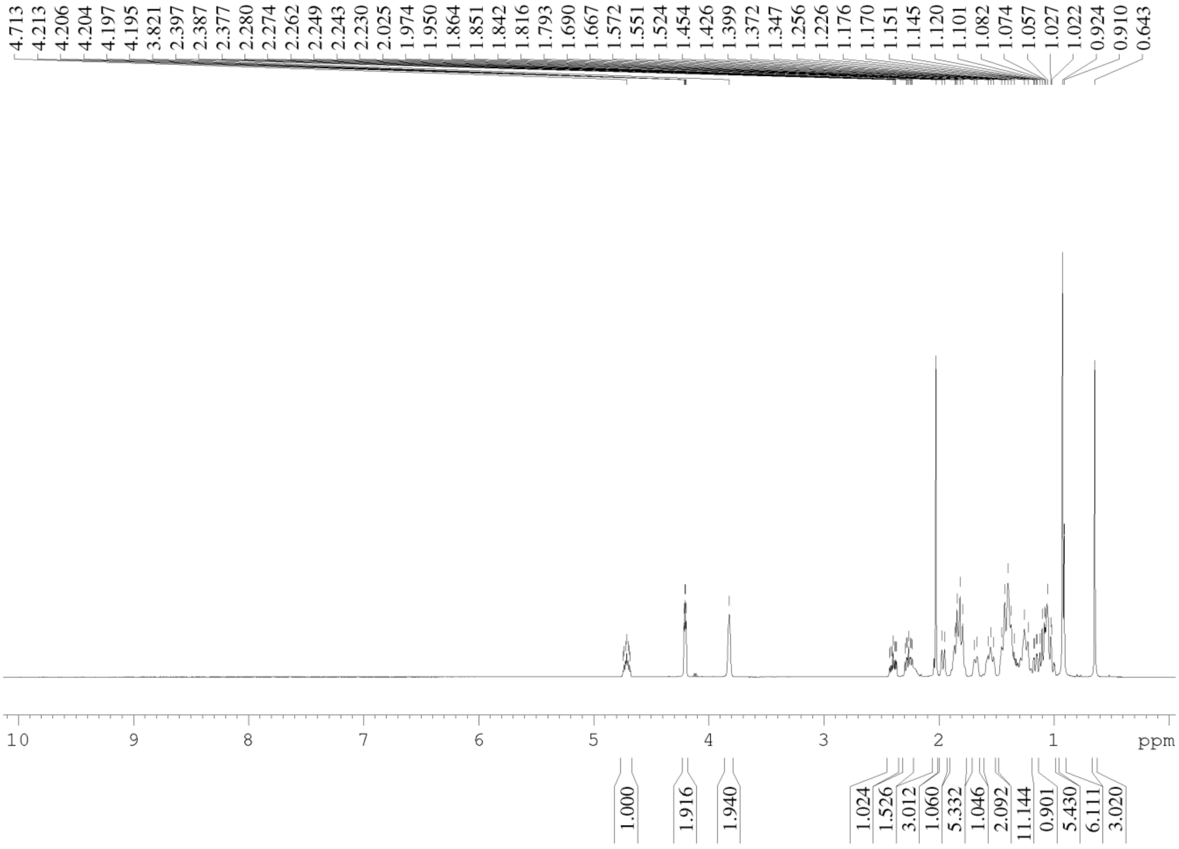

Figure S11. ^{13}C NMR Spectrum of compound **13b** (100 MHz, $CDCl_3$)

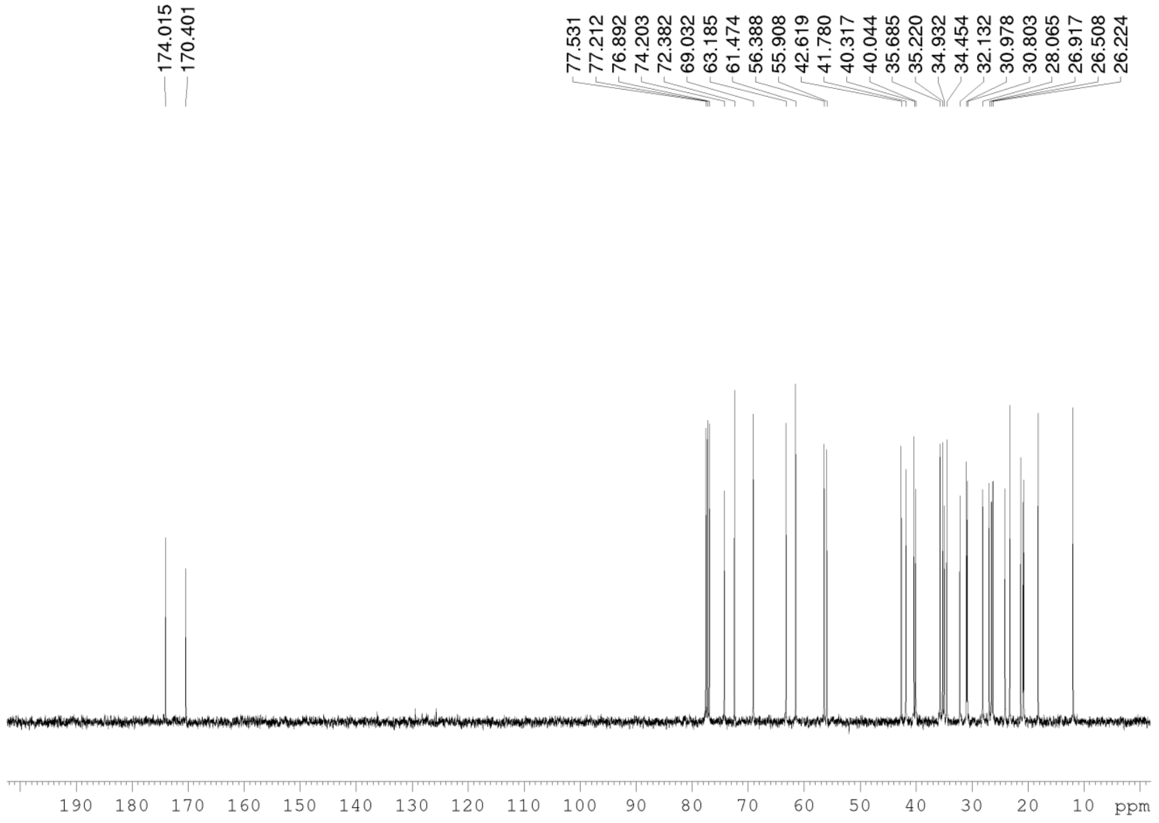

Figure S12. ^{1}H NMR Spectrum of compound **13b** (400 MHz, $CDCl_3$)

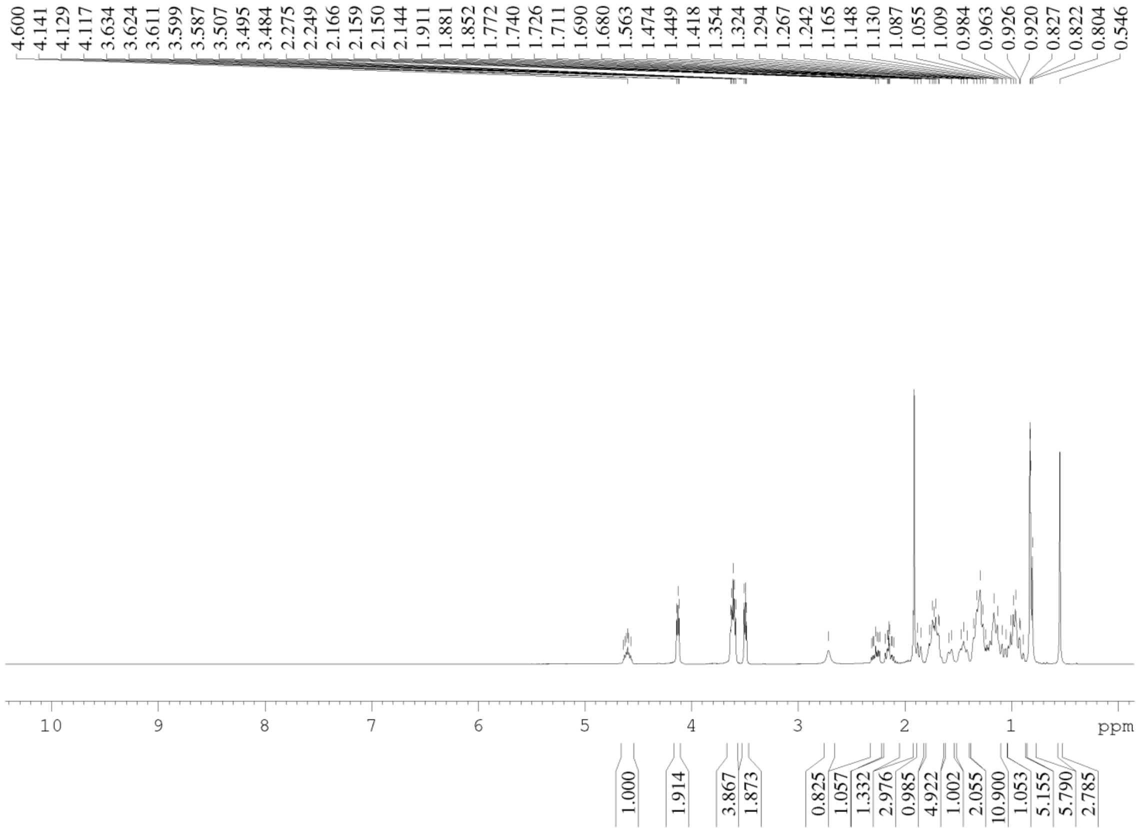

Figure S13. ^{13}C NMR Spectrum of compound **13c** (125 MHz, $CDCl_3$)

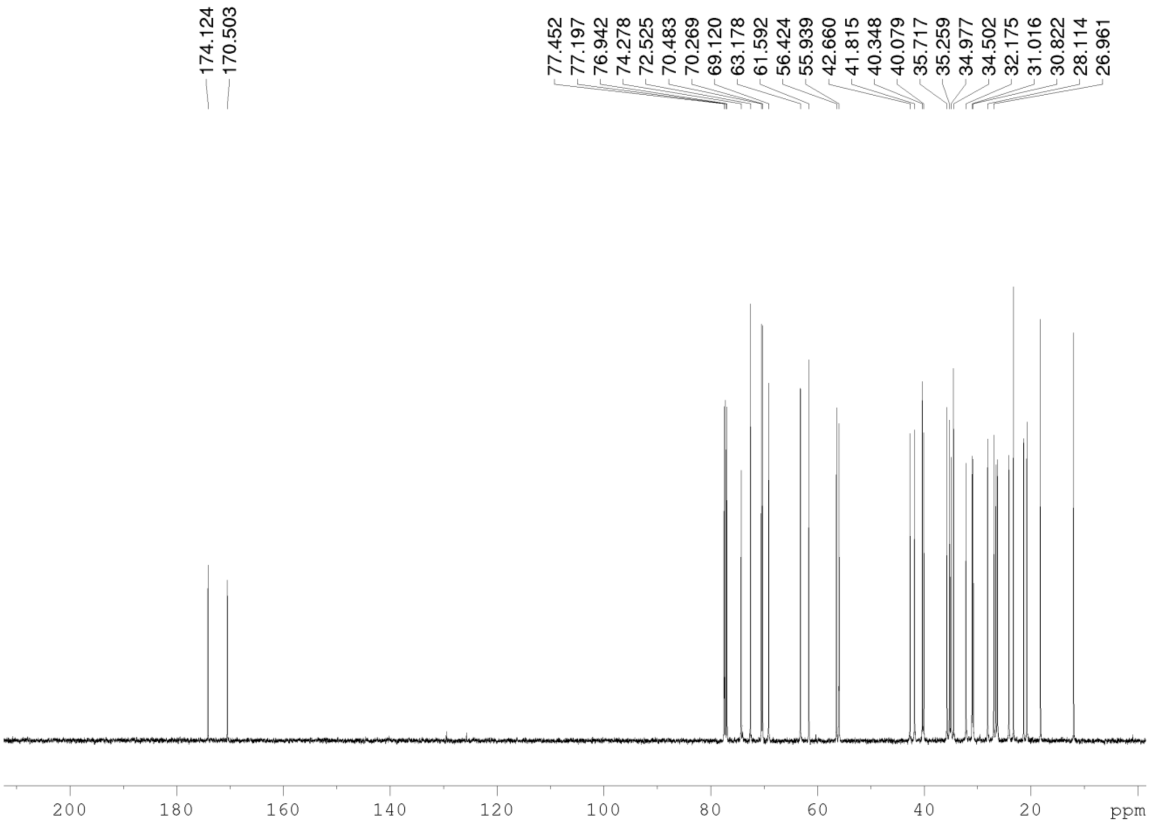

Figure S14. ^{1}H NMR Spectrum of compound **13c** (500 MHz, $CDCl_3$)

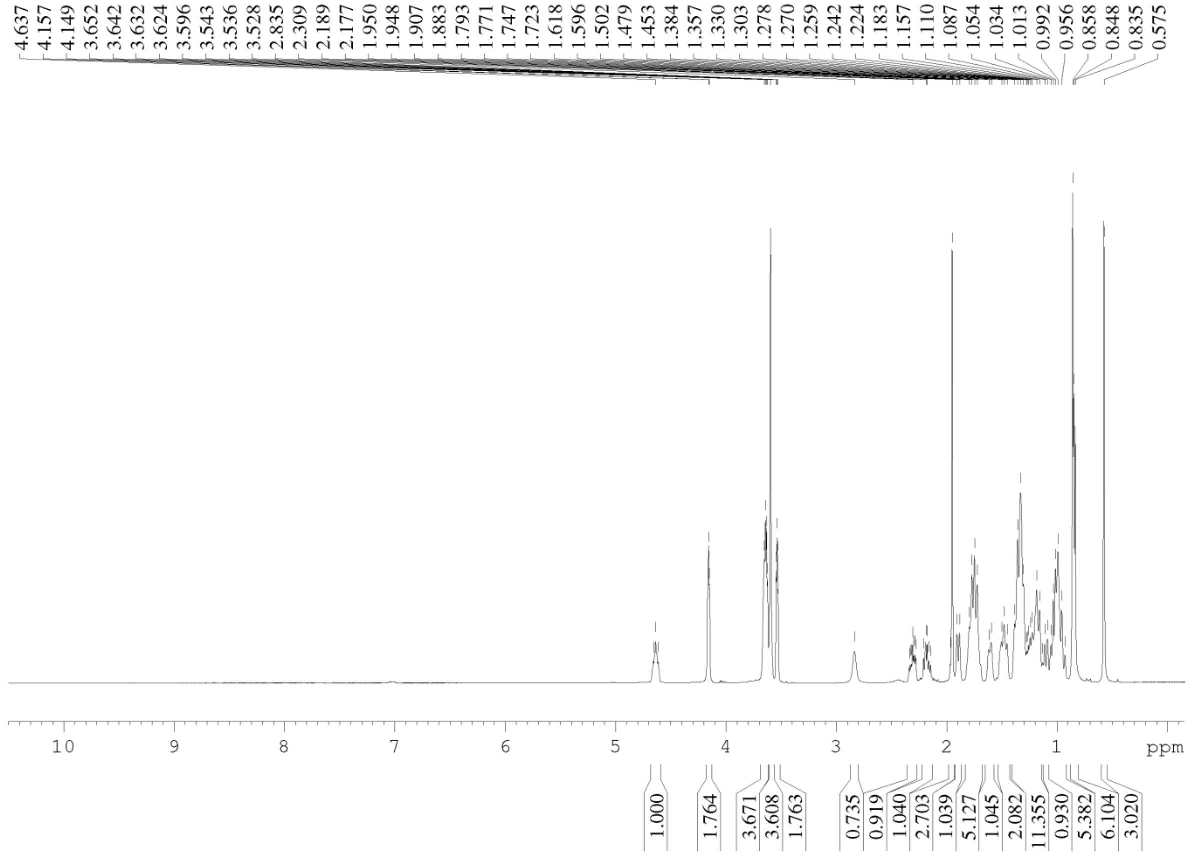

Figure S15. ^{13}C NMR Spectrum of compound **13d** (125 MHz, $CDCl_3$)

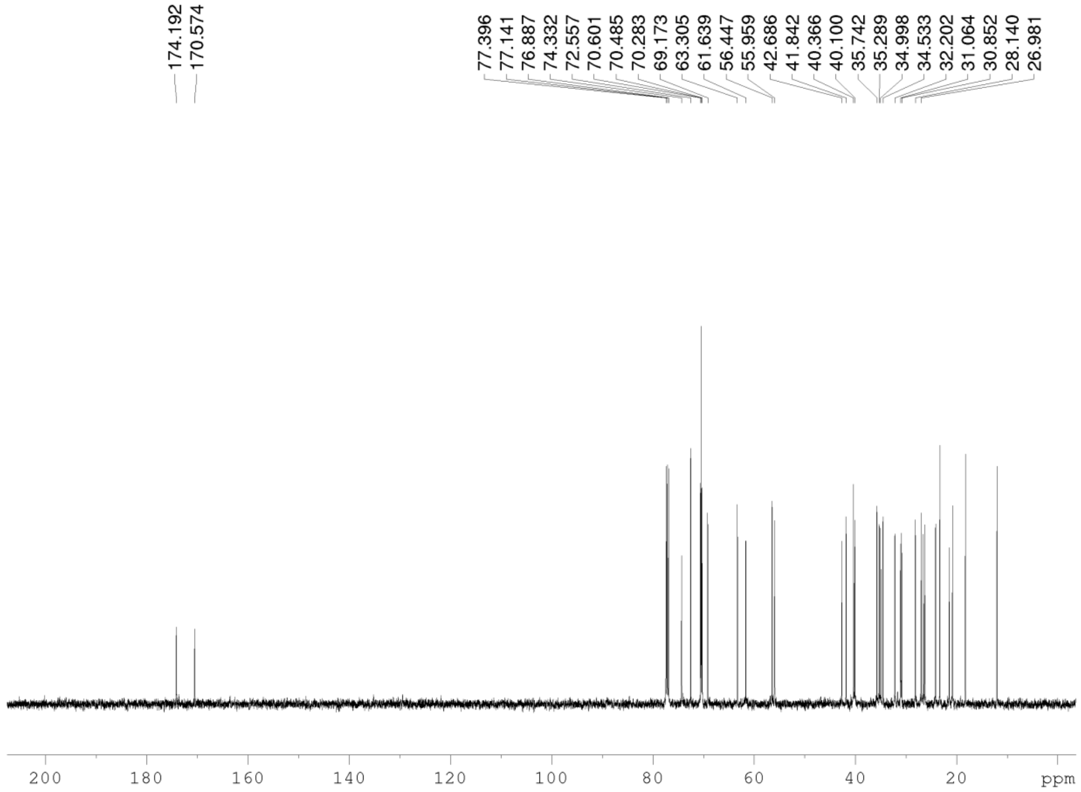

Figure S16. ^{1}H NMR Spectrum of compound **13d** (500 MHz, $CDCl_3$)

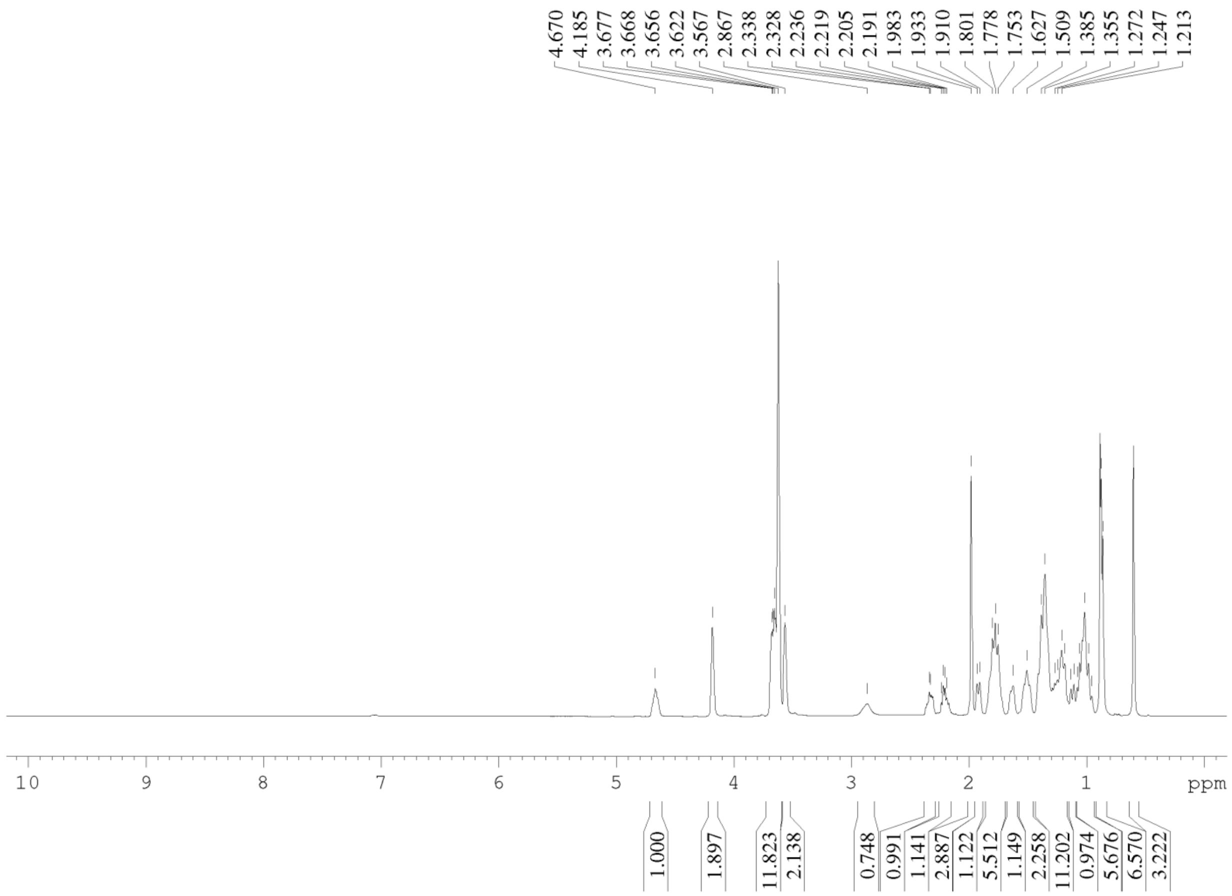

Figure S17. ^{13}C NMR Spectrum of compound **14a** (125 MHz, $CDCl_3$)

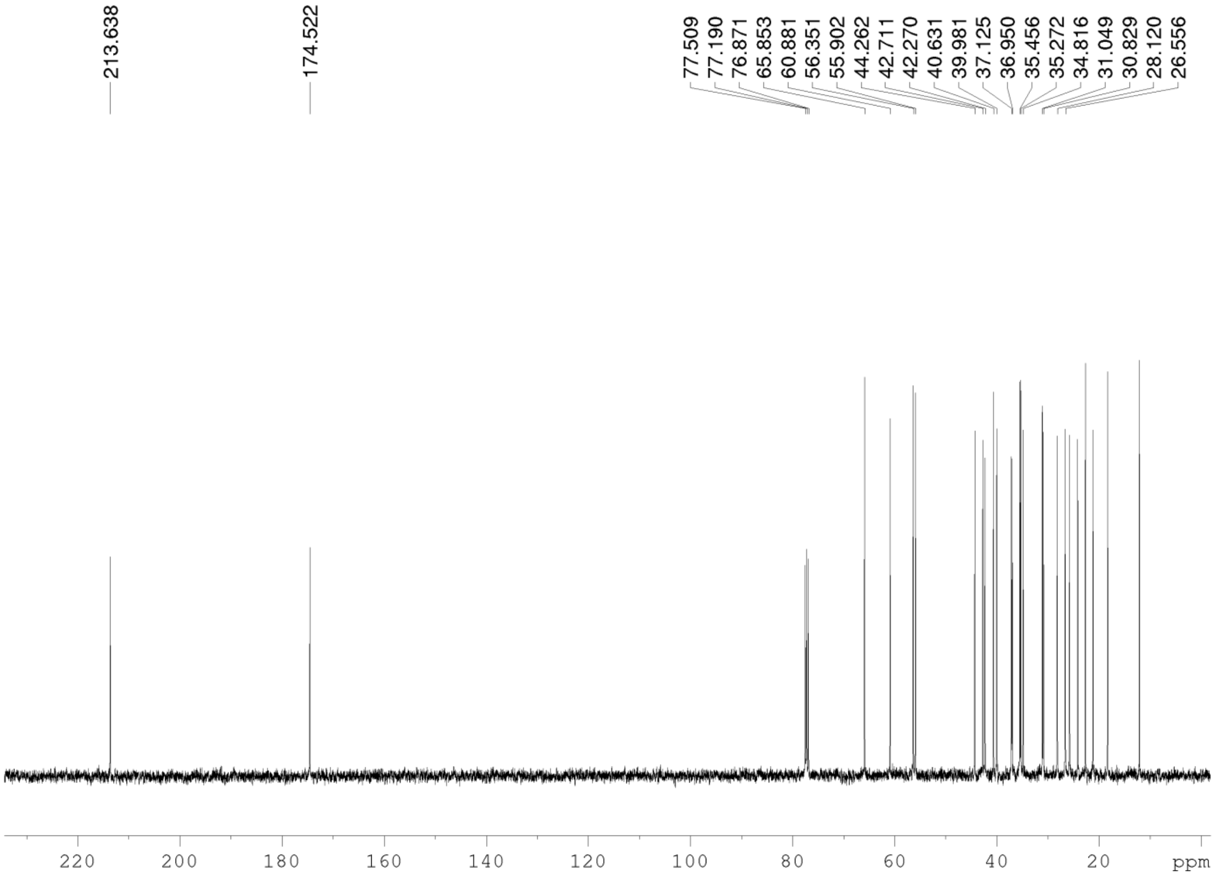

Figure S18. ^{1}H NMR Spectrum of compound **14a** (500 MHz, $CDCl_3$)

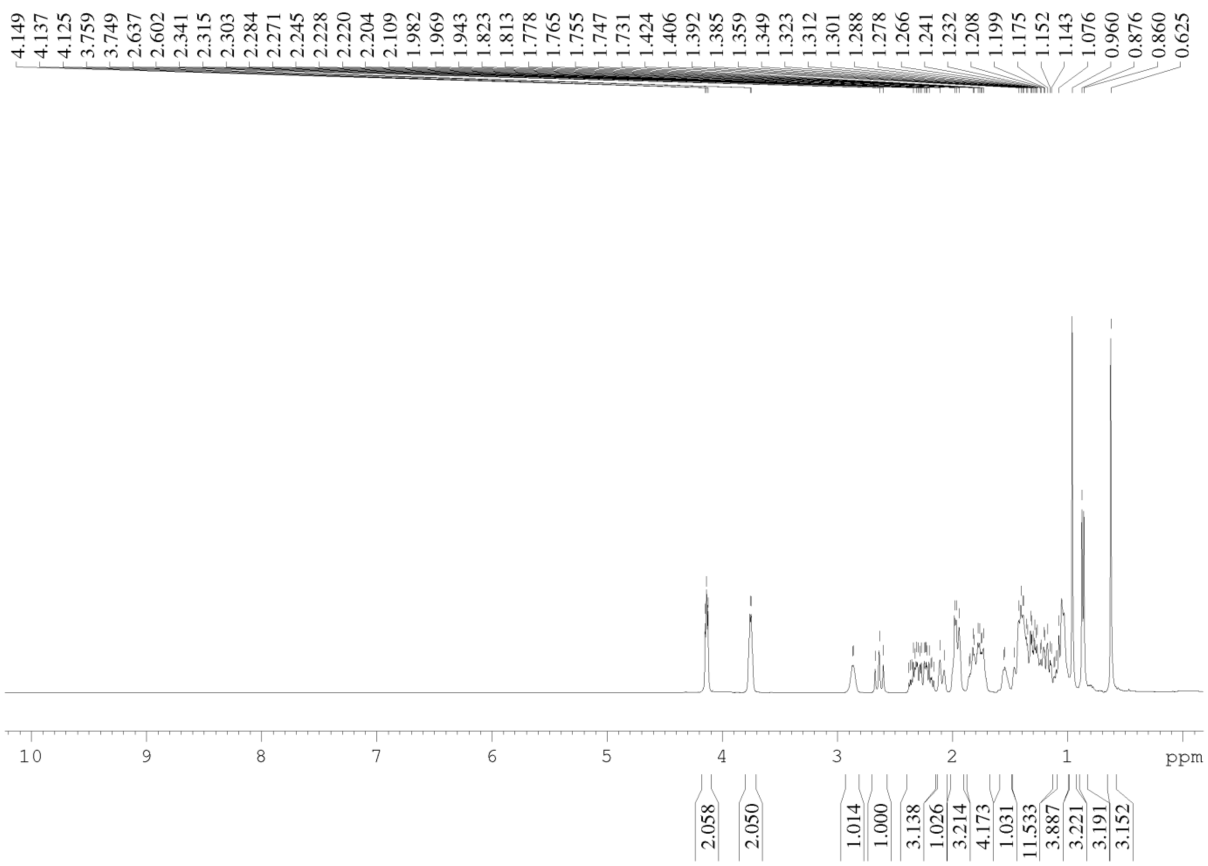

Figure S19. ^{13}C NMR Spectrum of compound **14b** (125 MHz, $CDCl_3$)

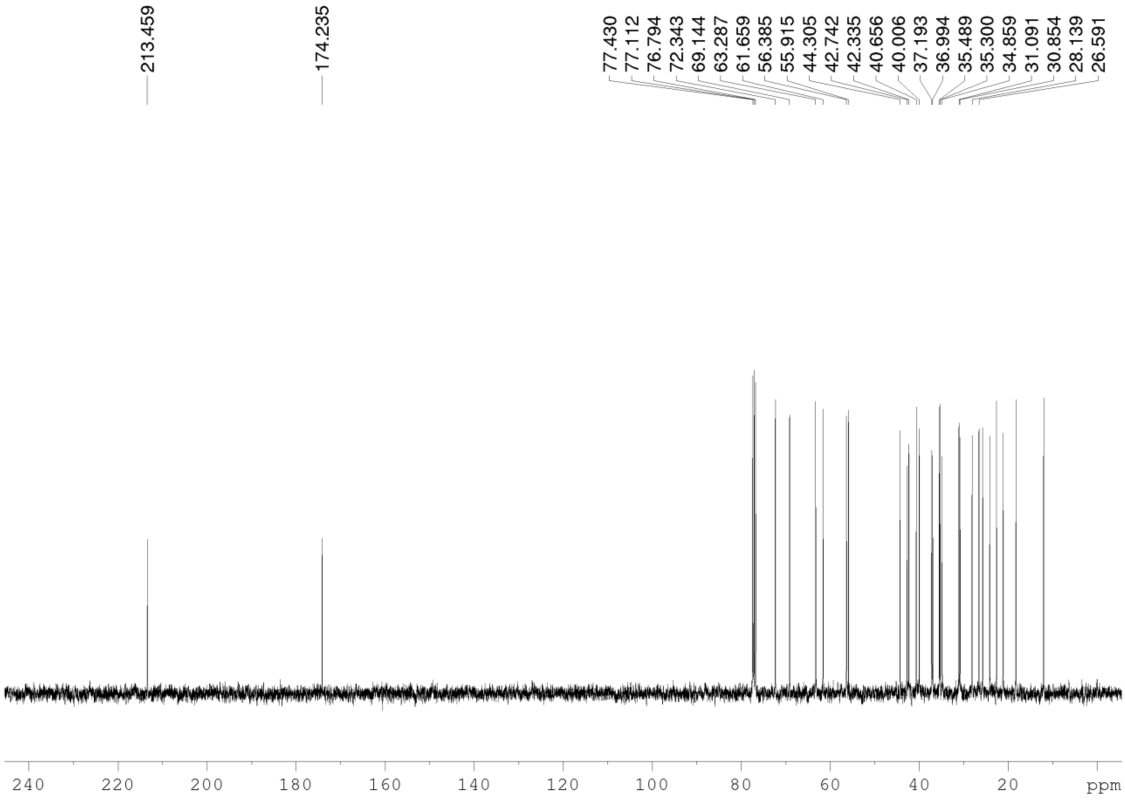

Figure S20. ^{1}H NMR Spectrum of compound **14b** (500 MHz, $CDCl_3$)

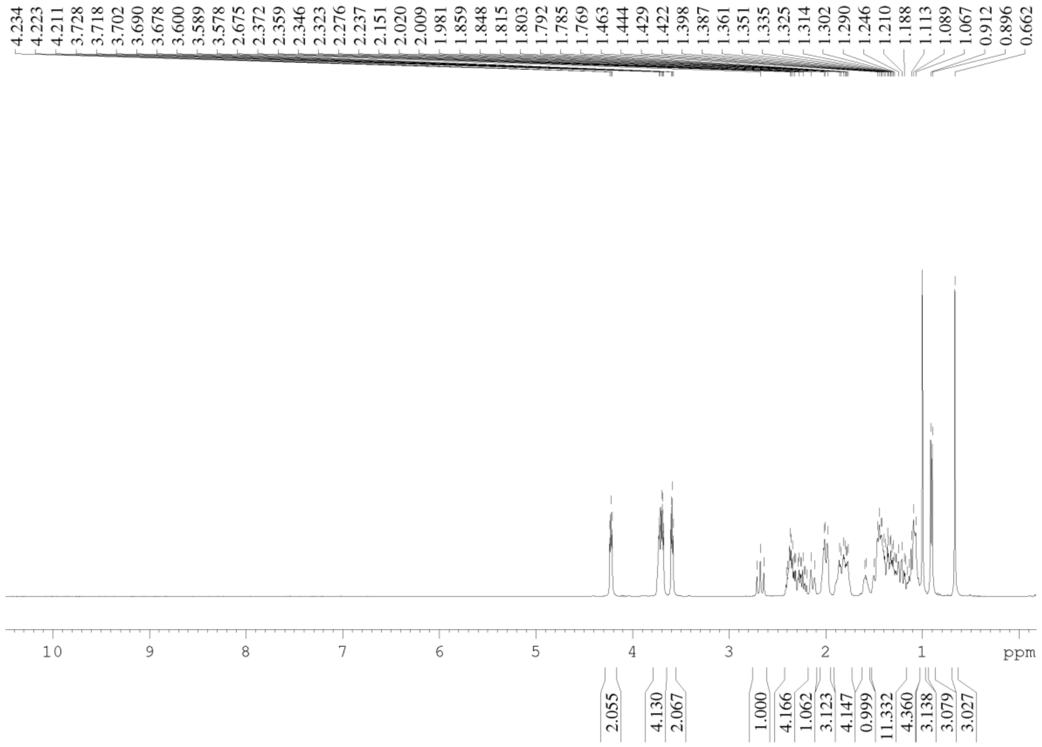

Figure S21. ^{13}C NMR Spectrum of compound **14c** (125 MHz, $CDCl_3$)

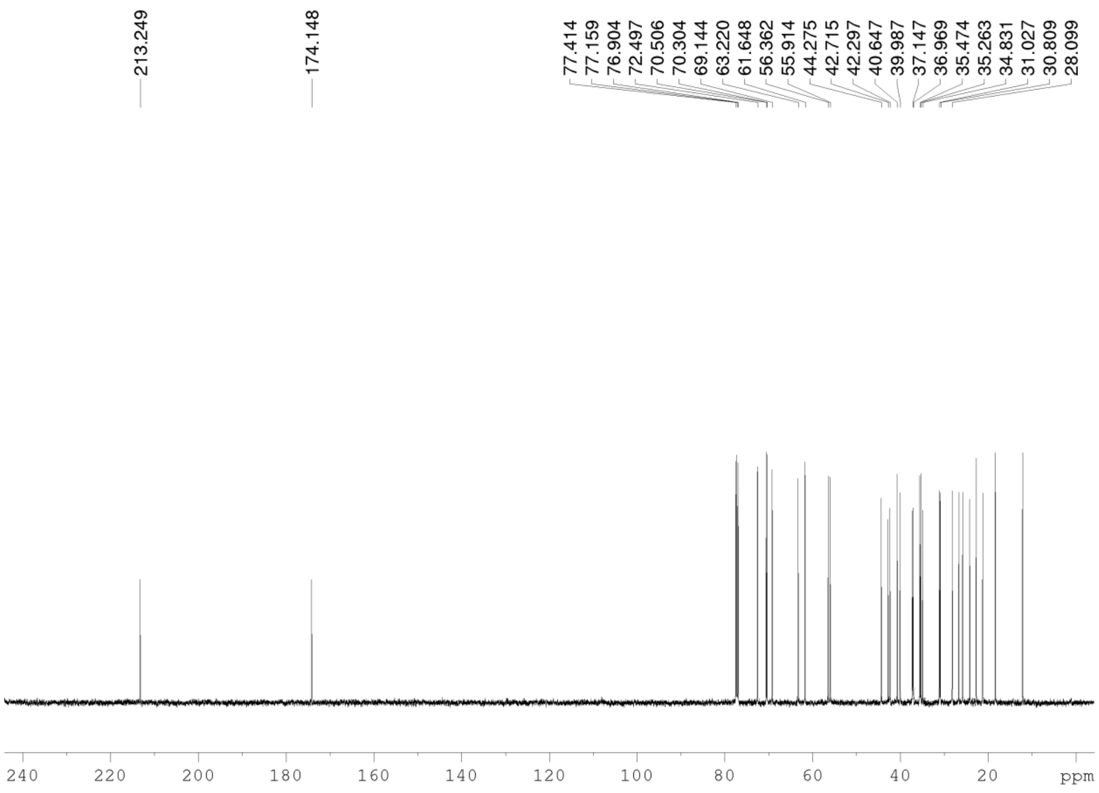

Figure S22. ^{1}H NMR Spectrum of compound **14c** (500 MHz, $CDCl_3$)

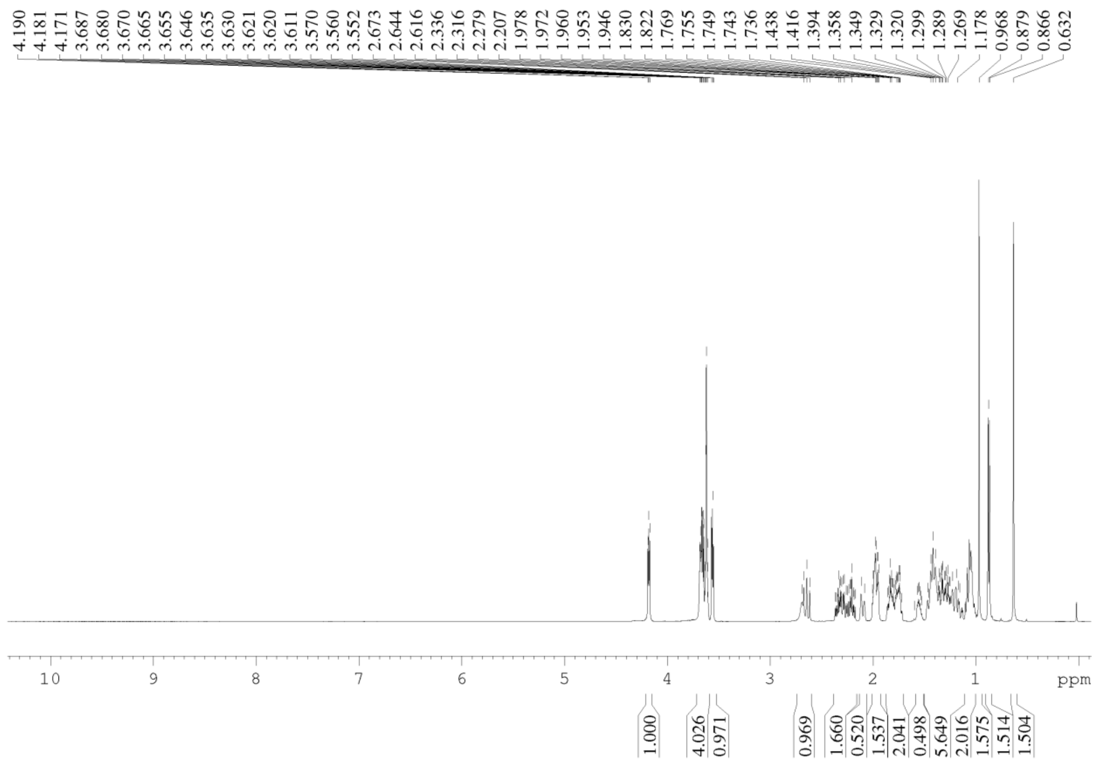

Figure S23. ^{13}C NMR Spectrum of compound **14d** (125 MHz, $CDCl_3$)

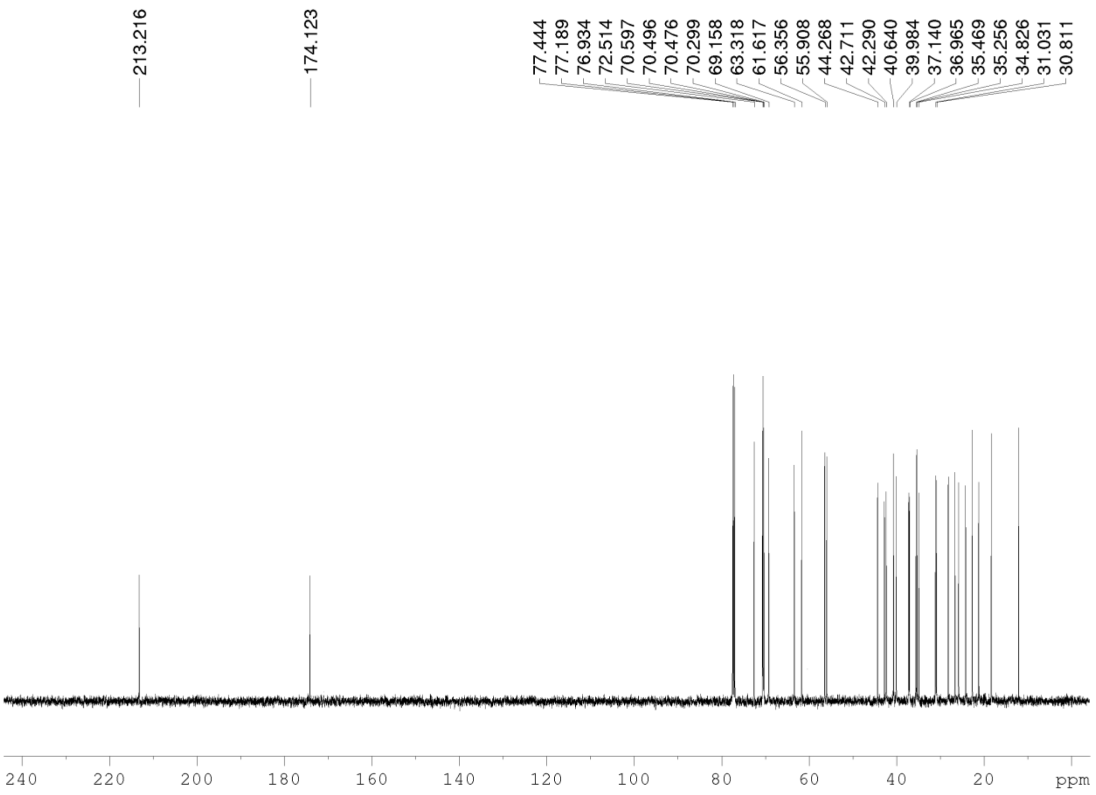

Figure S24. ^{1}H NMR Spectrum of compound **14d** (500 MHz, $CDCl_3$)

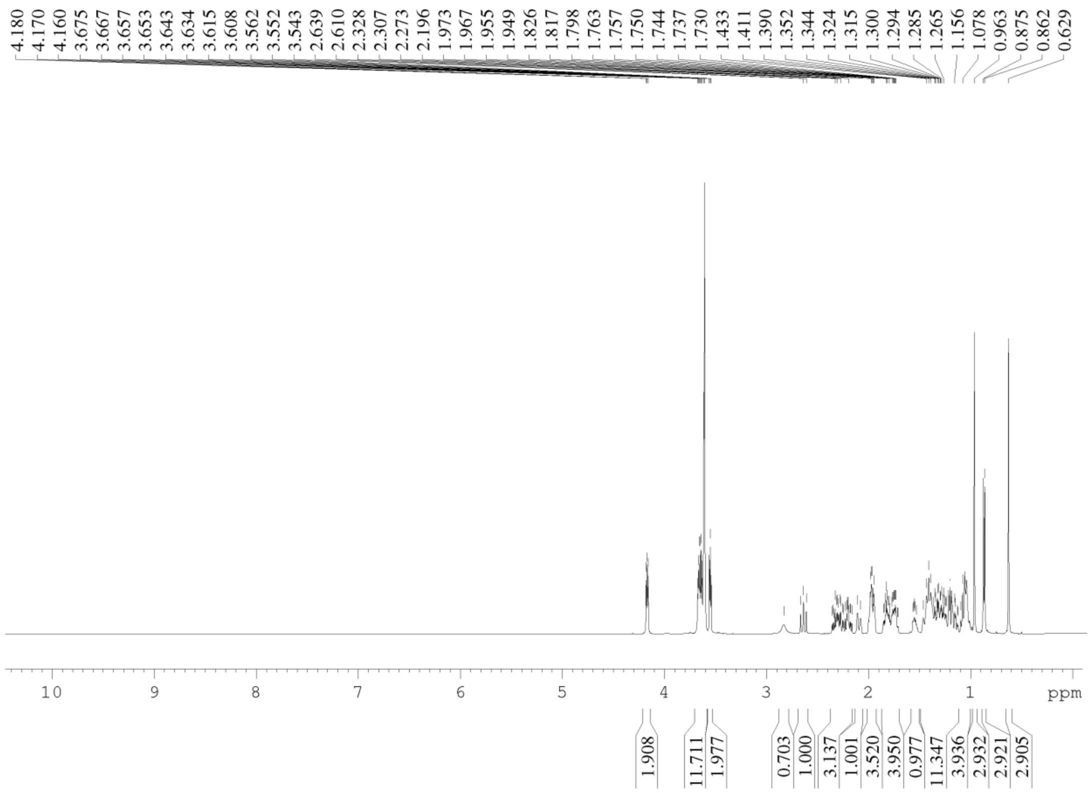

Figure S25. ^{13}C NMR Spectrum of compound **15a** (125 MHz, $CDCl_3$)

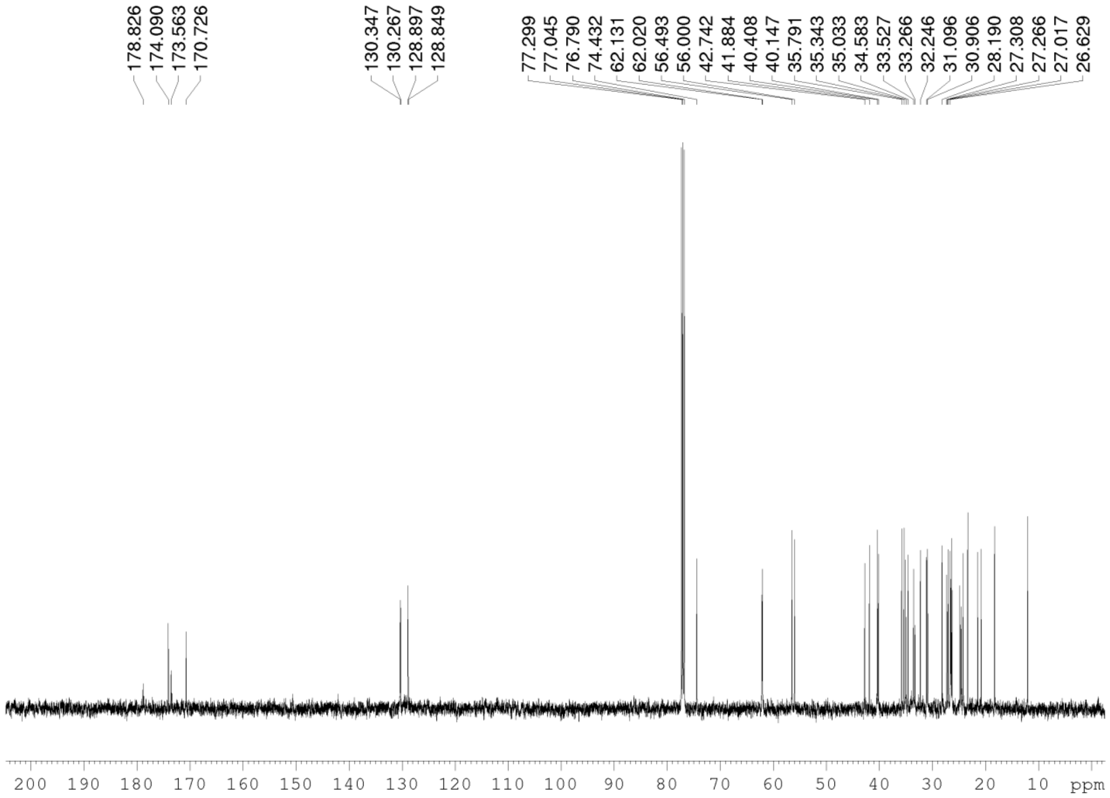

Figure S26. ^{1}H NMR Spectrum of compound **15a** (500 MHz, $CDCl_3$)

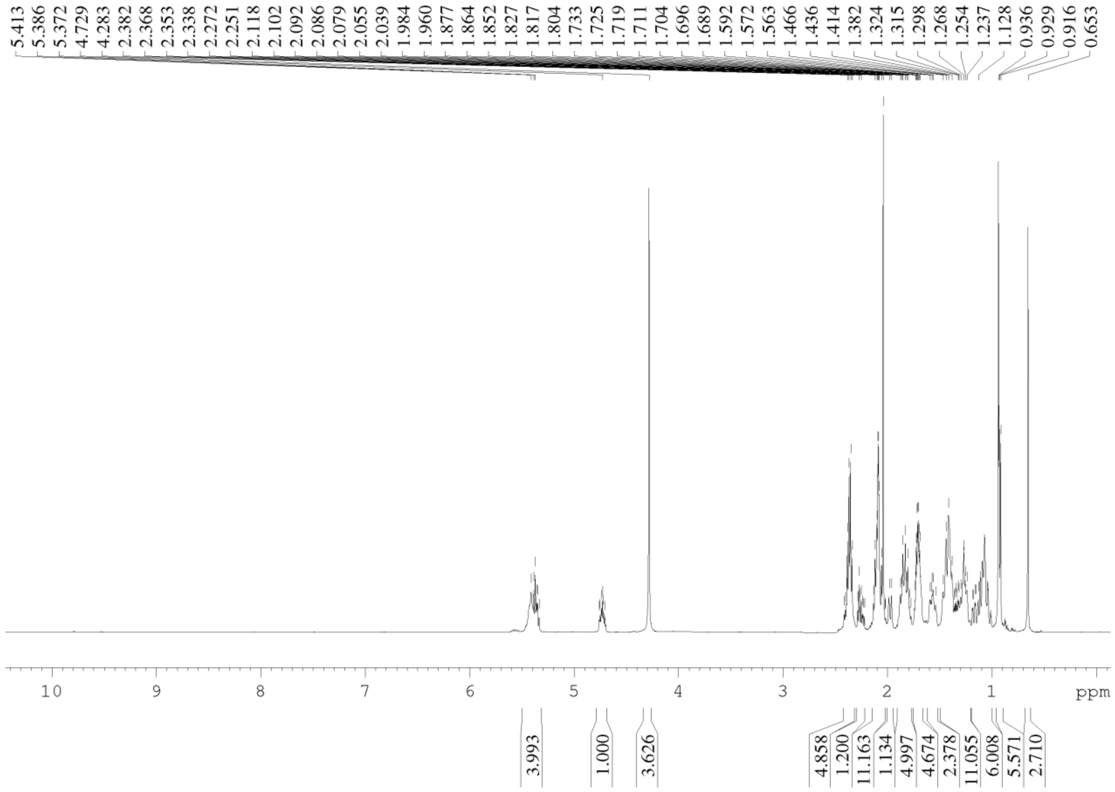

Figure S27. ^{13}C NMR Spectrum of compound **15b** (125 MHz, $CDCl_3$)

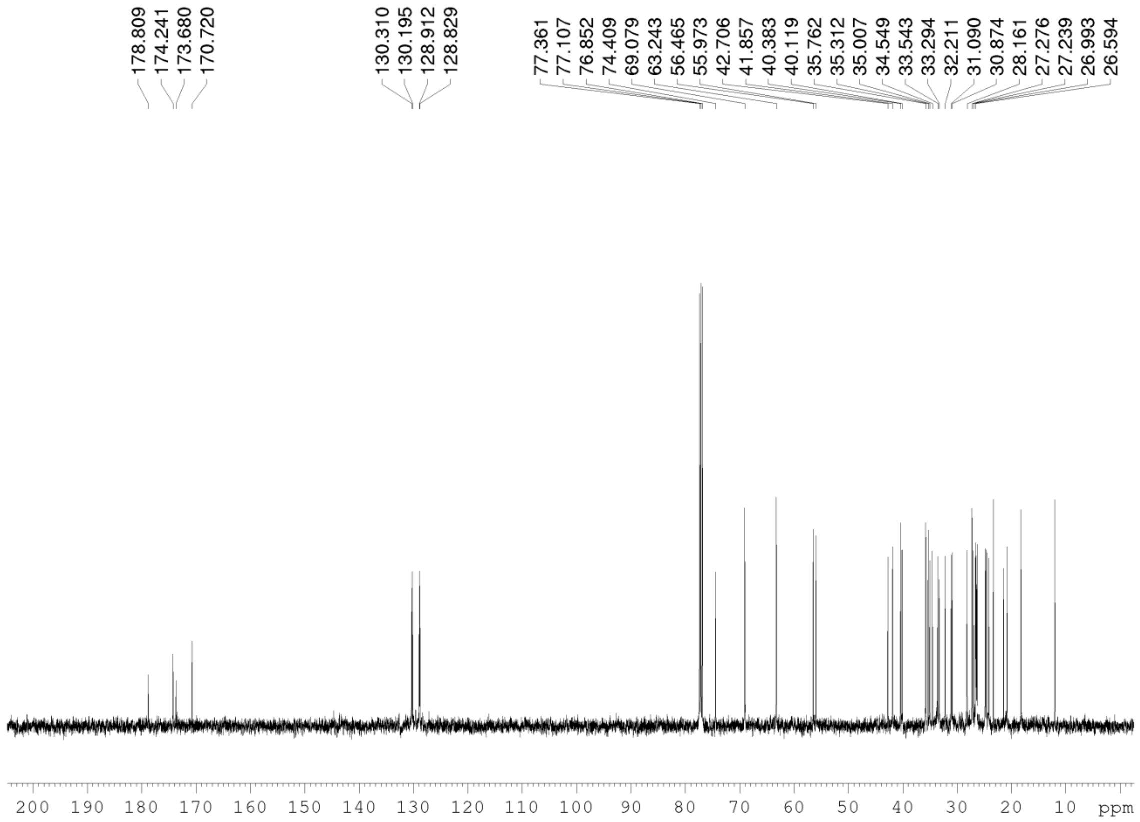

Figure S28. ^{1}H NMR Spectrum of compound **15b** (500 MHz, $CDCl_3$)

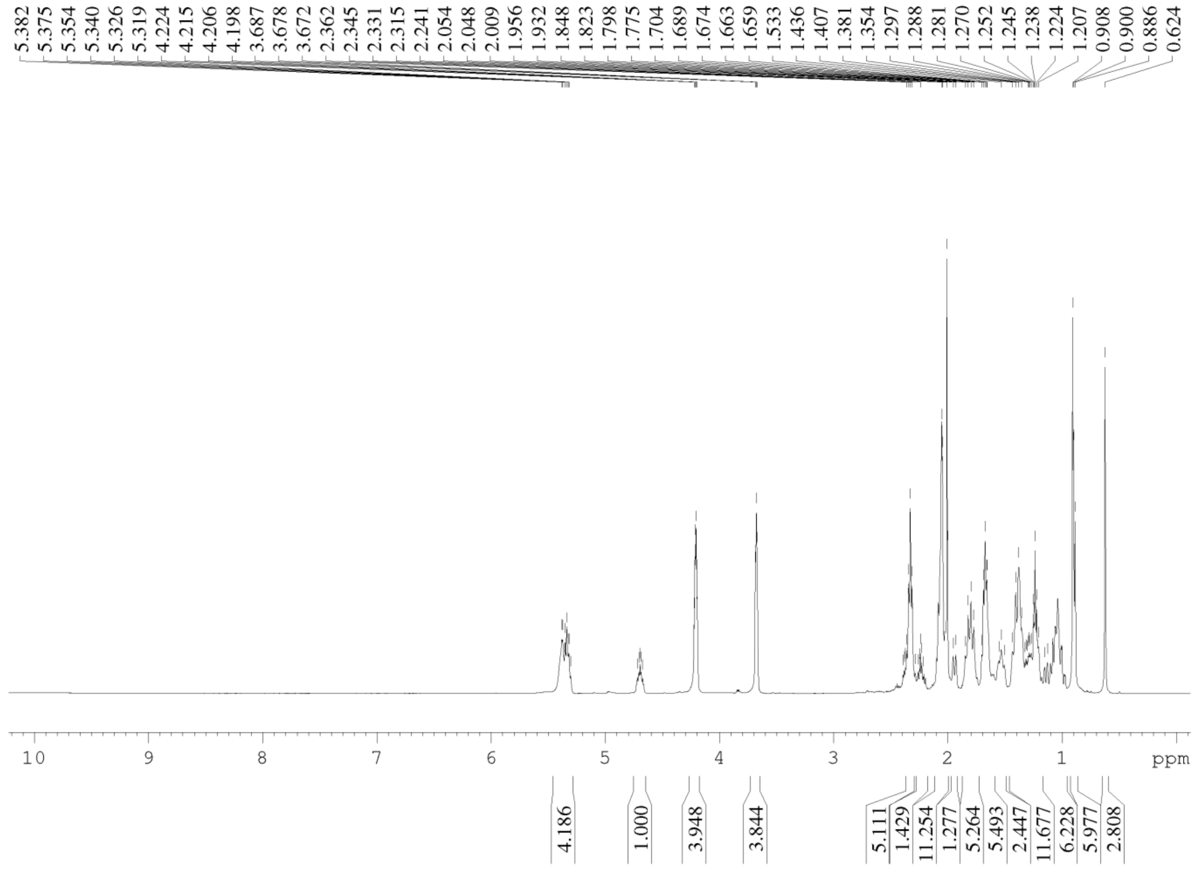

Figure S29. ^{13}C NMR Spectrum of compound **15c** (125 MHz, $CDCl_3$)

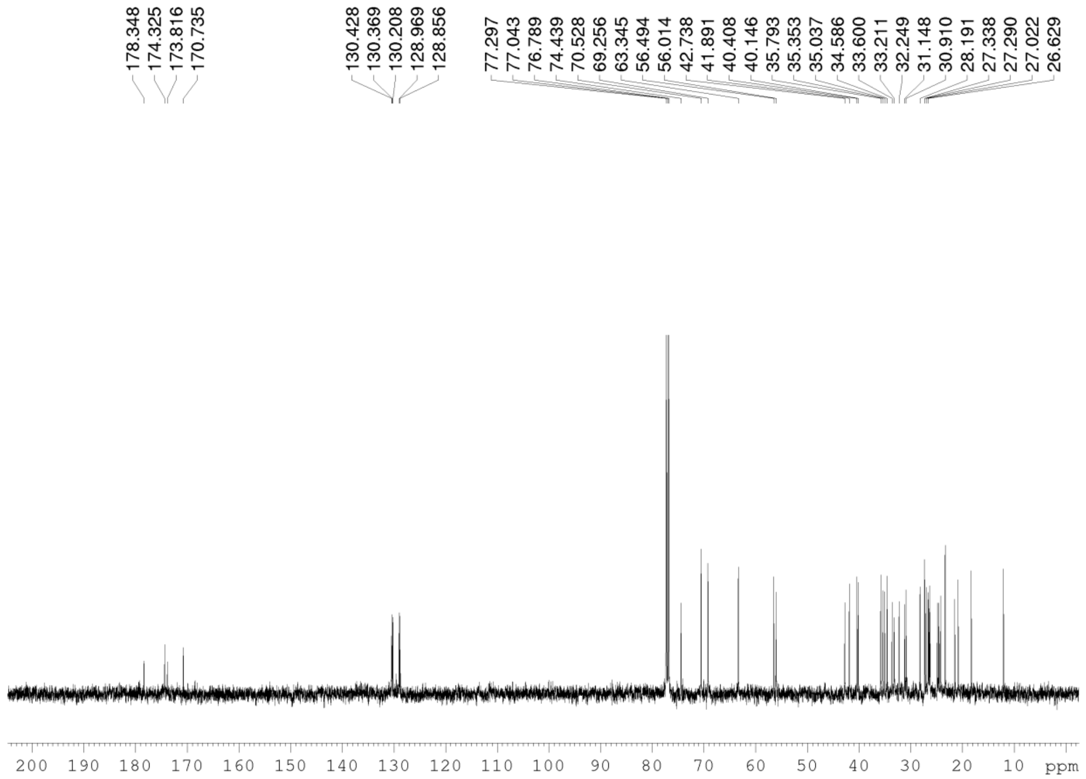

Figure S30. ^{1}H NMR Spectrum of compound **15c** (500 MHz, $CDCl_3$)

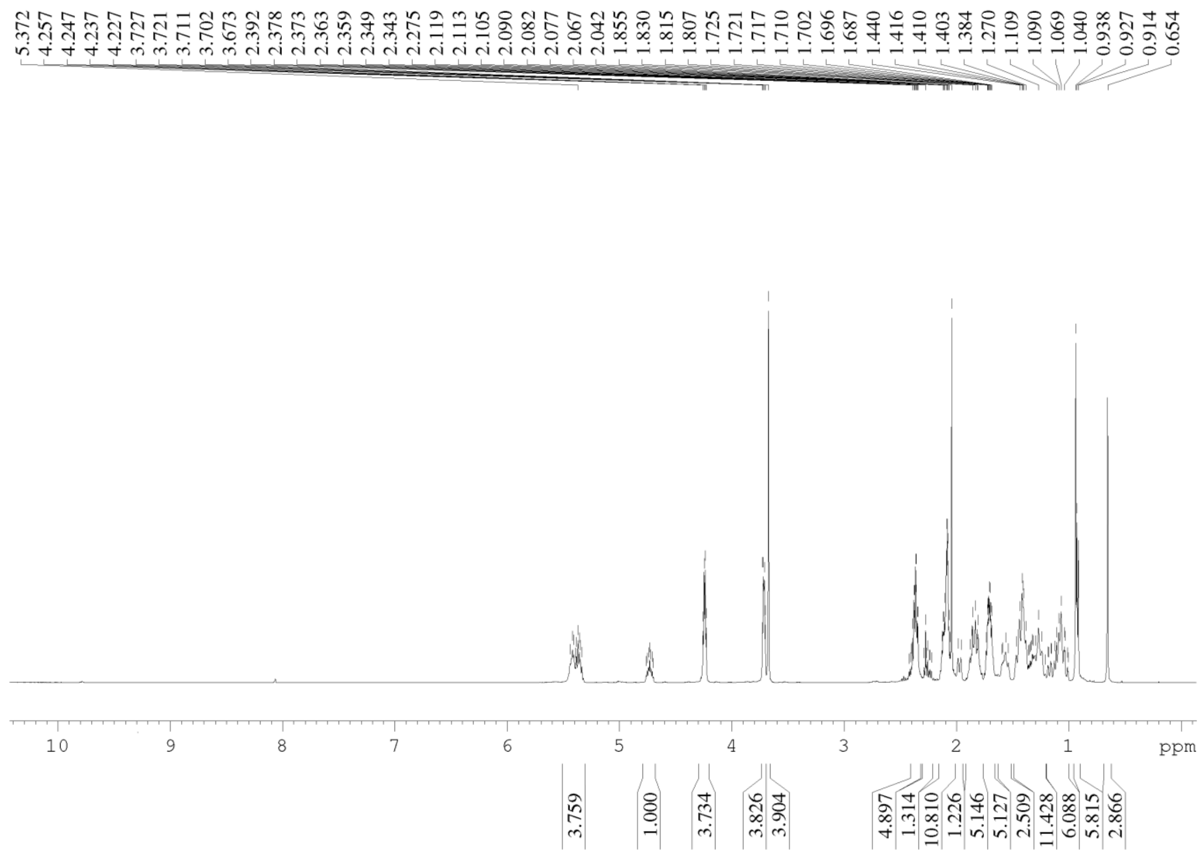

Figure S31. ^{13}C NMR Spectrum of compound **15d** (125 MHz, $CDCl_3$)

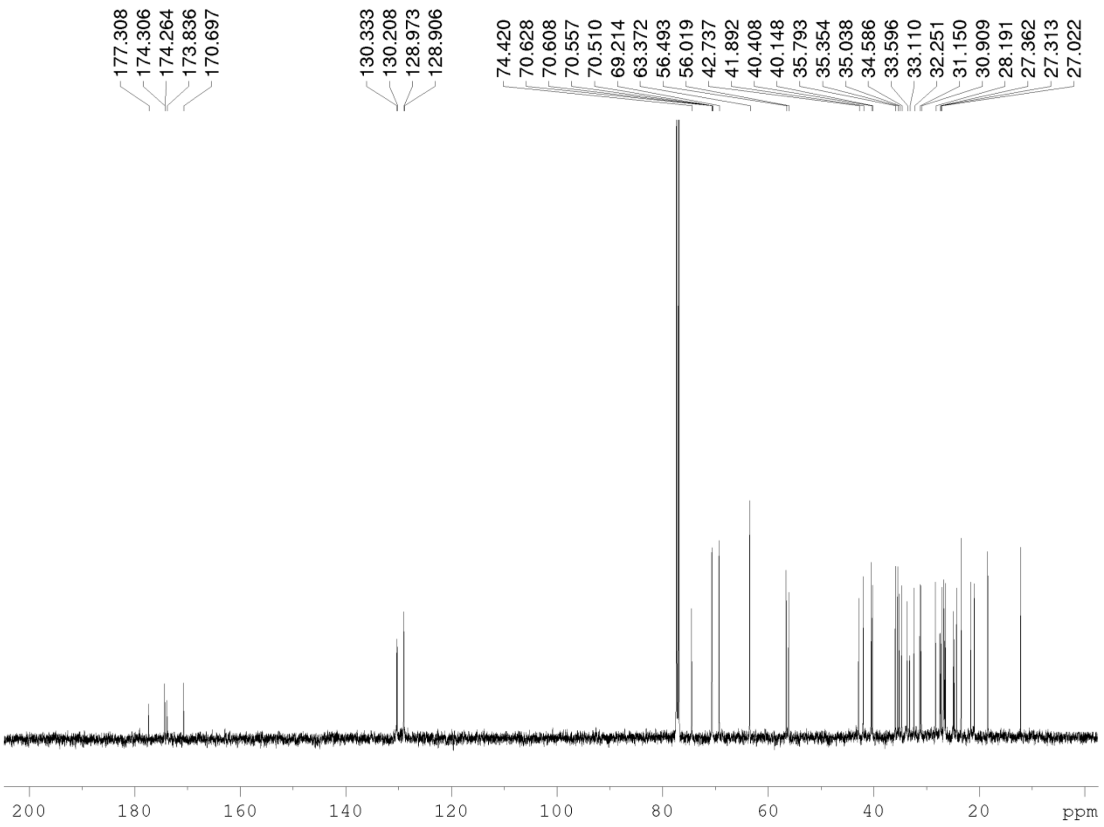

Figure S32. ^{1}H NMR Spectrum of compound **15d** (500 MHz, $CDCl_3$)

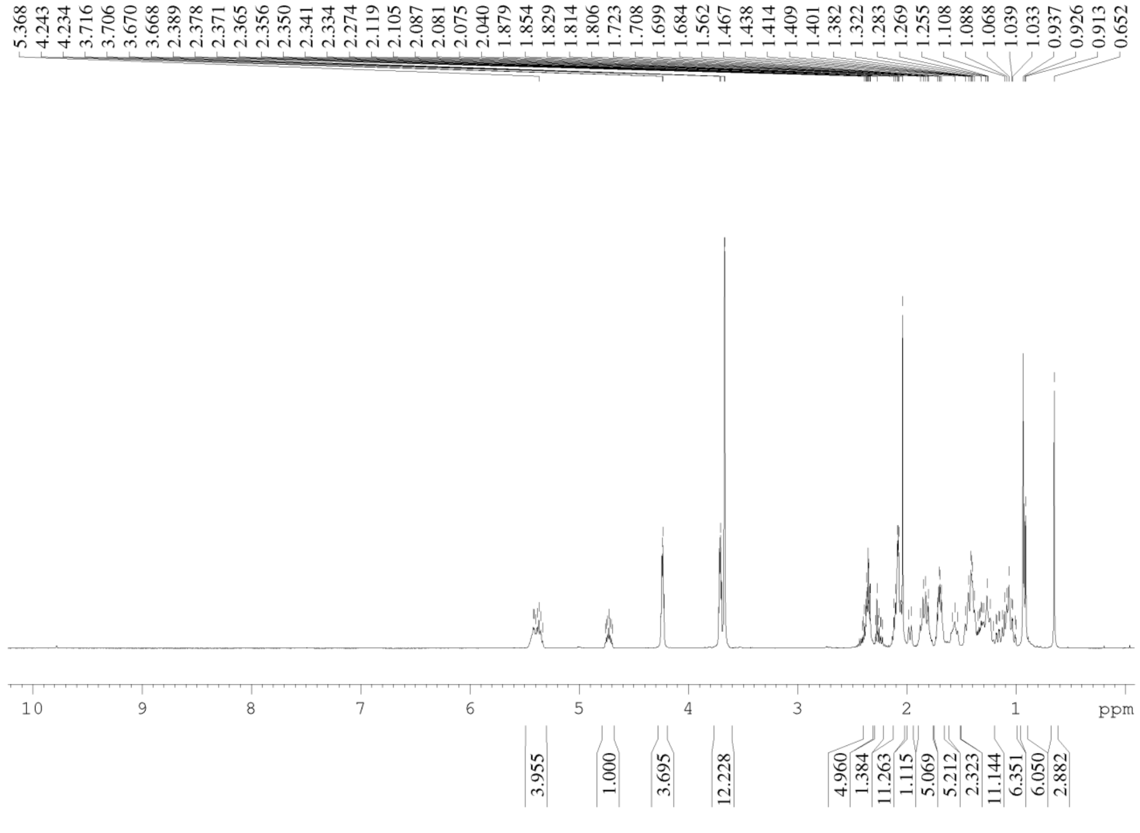

Figure S33. ^{13}C NMR Spectrum of compound **16a** (125 MHz, $CDCl_3$)

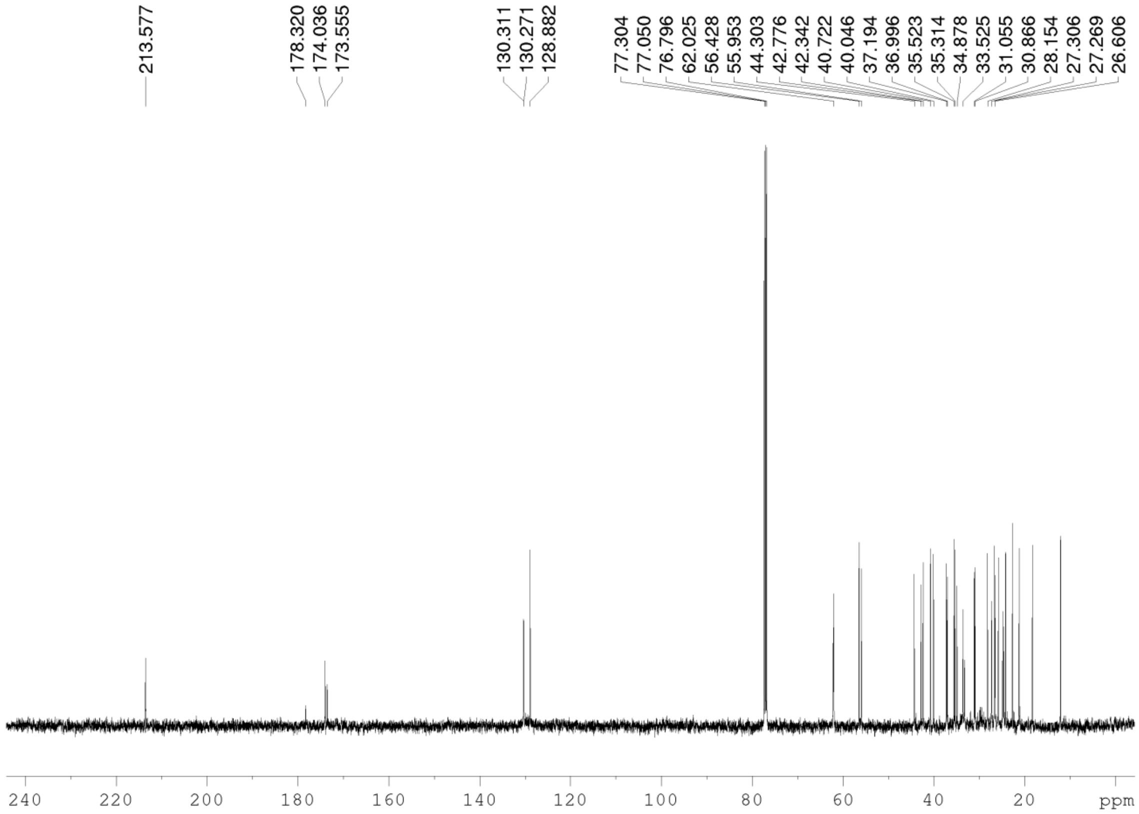

Figure S34. ^{1}H NMR Spectrum of compound **16a** (500 MHz, $CDCl_3$)

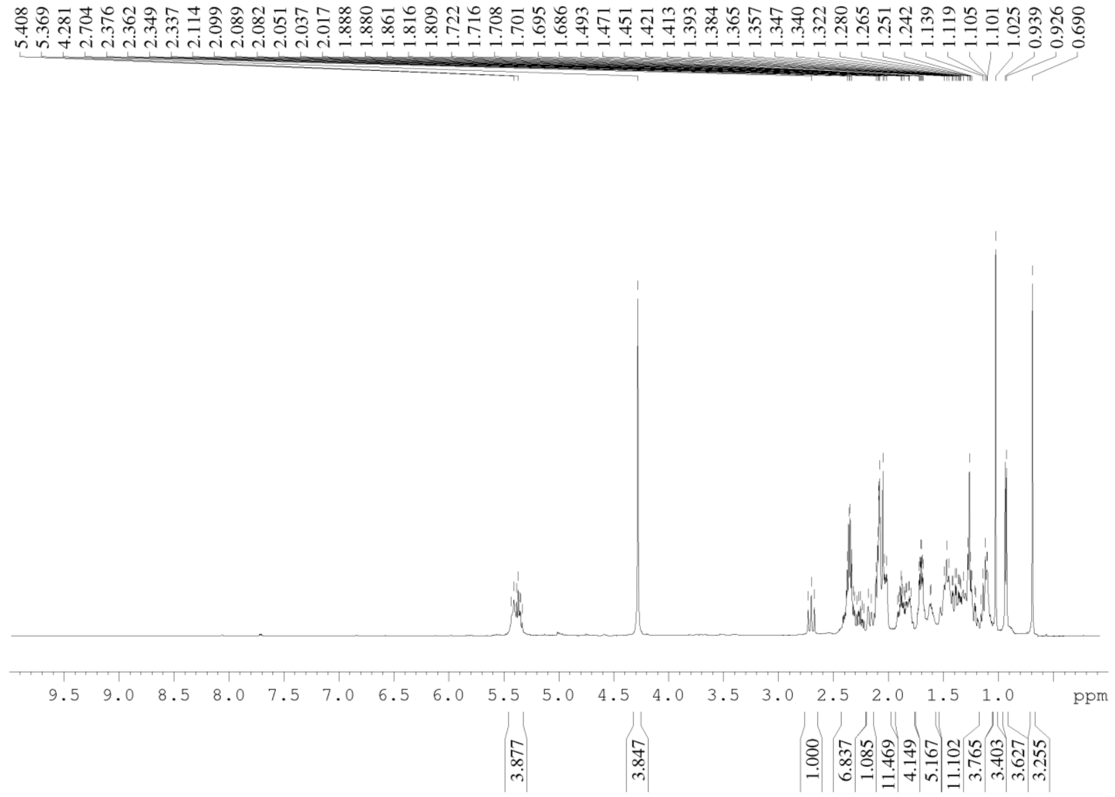

Figure S35. ^{13}C NMR Spectrum of compound **16b** (125 MHz, $CDCl_3$)

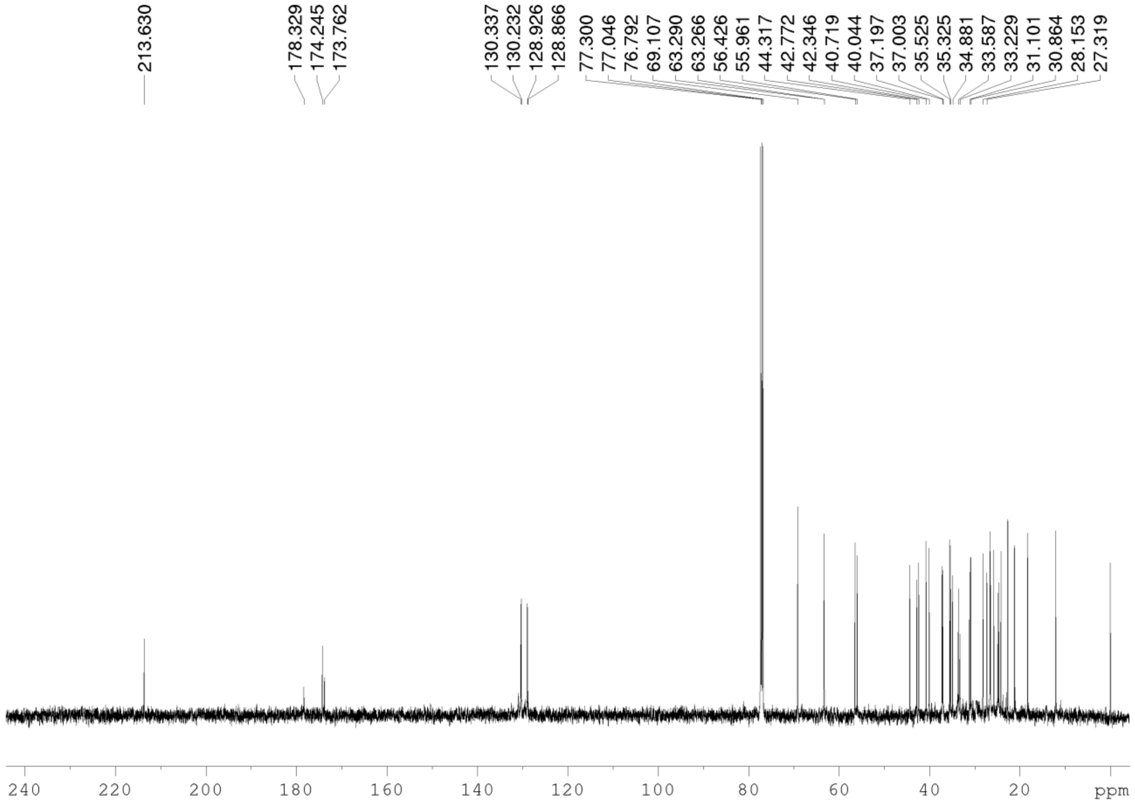

Figure S36. ^{1}H NMR Spectrum of compound **16b** (500 MHz, $CDCl_3$)

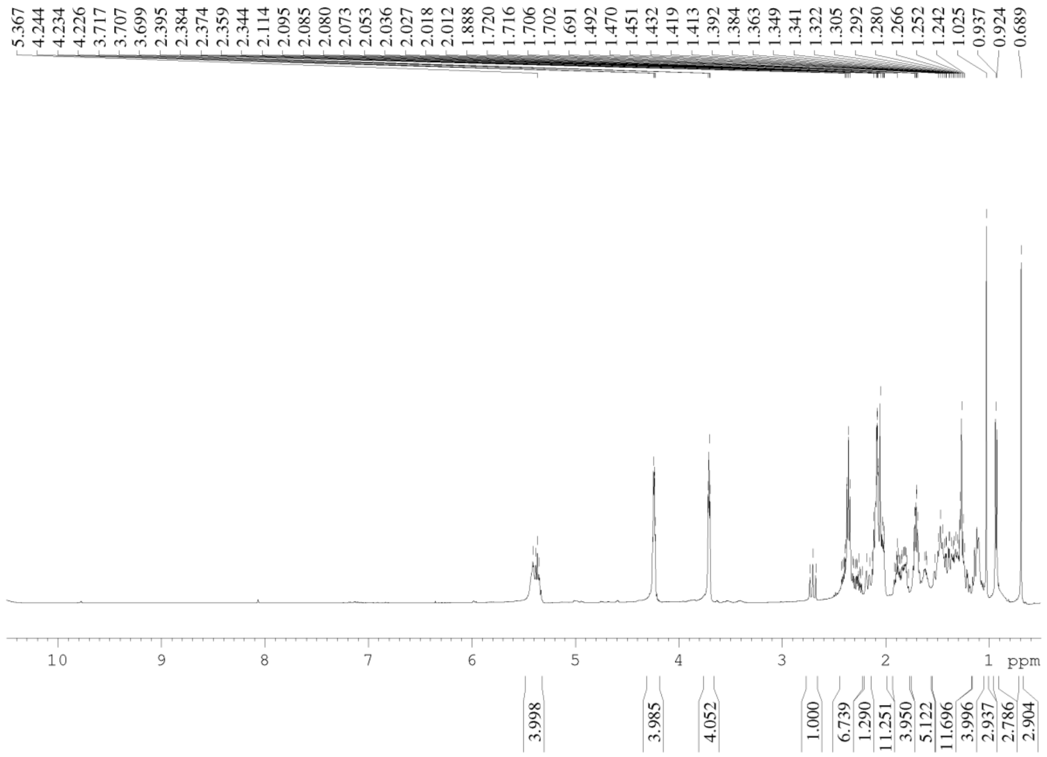

Figure S37. ^{13}C NMR Spectrum of compound **16c** (125 MHz, $CDCl_3$)

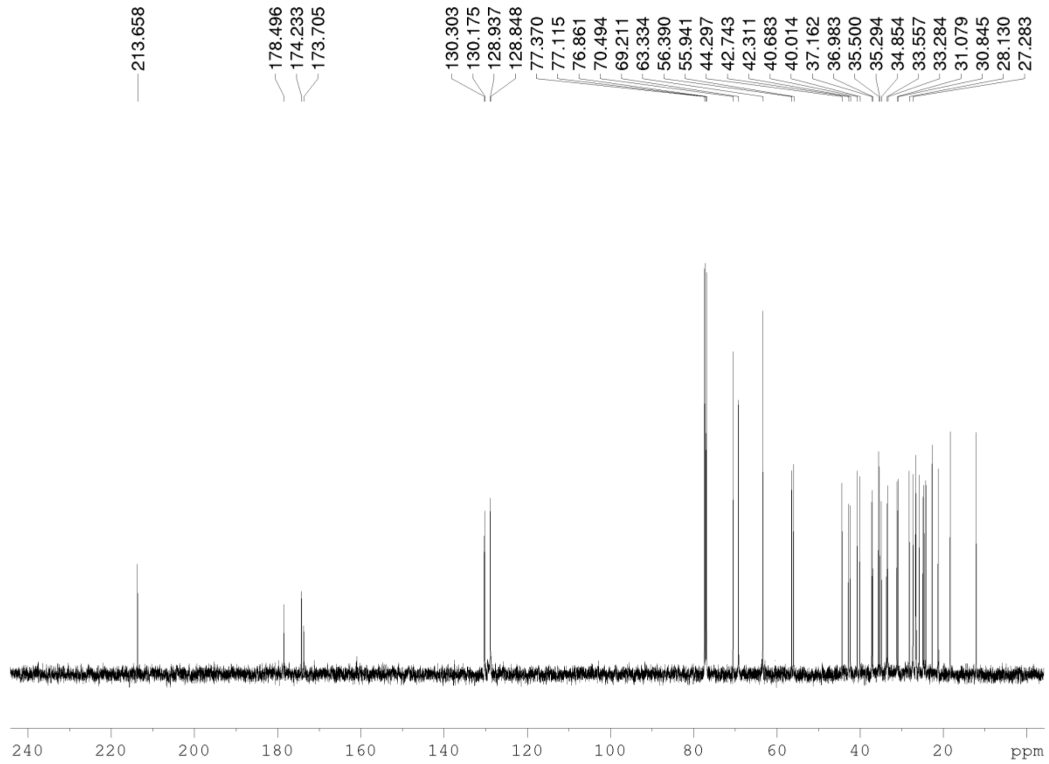

Figure S38. ^{1}H NMR Spectrum of compound **16c** (500 MHz, $CDCl_3$)

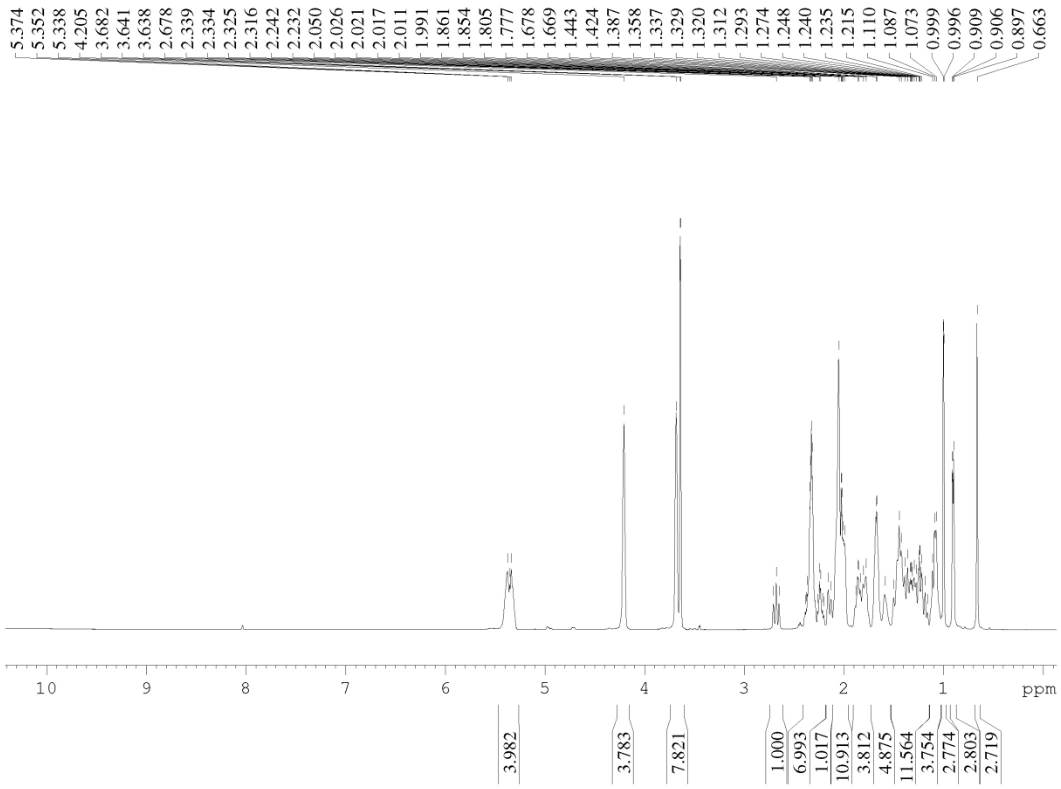

Figure S39. ^{13}C NMR Spectrum of compound **16d** (125 MHz, $CDCl_3$)

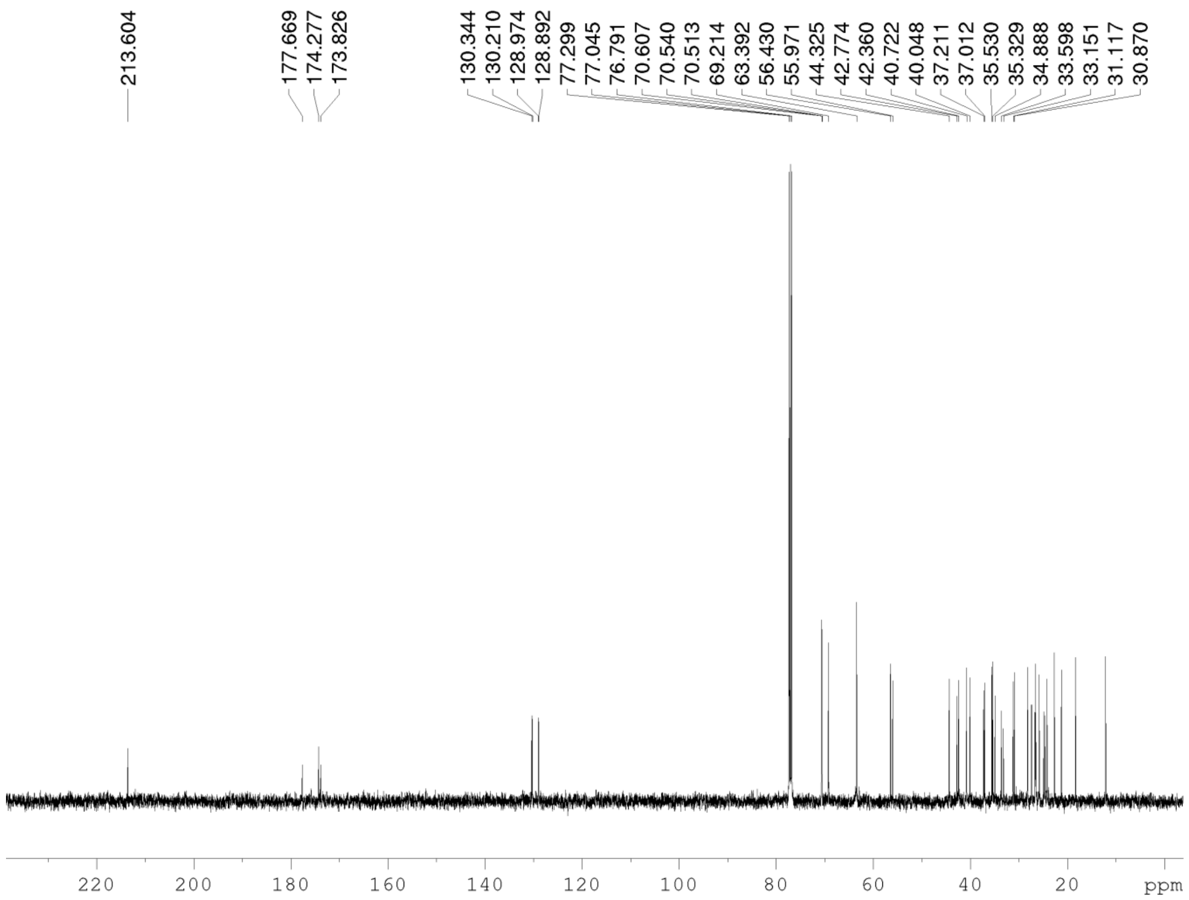

Figure S40. ^{1}H NMR Spectrum of compound **16d** (500 MHz, $CDCl_3$)

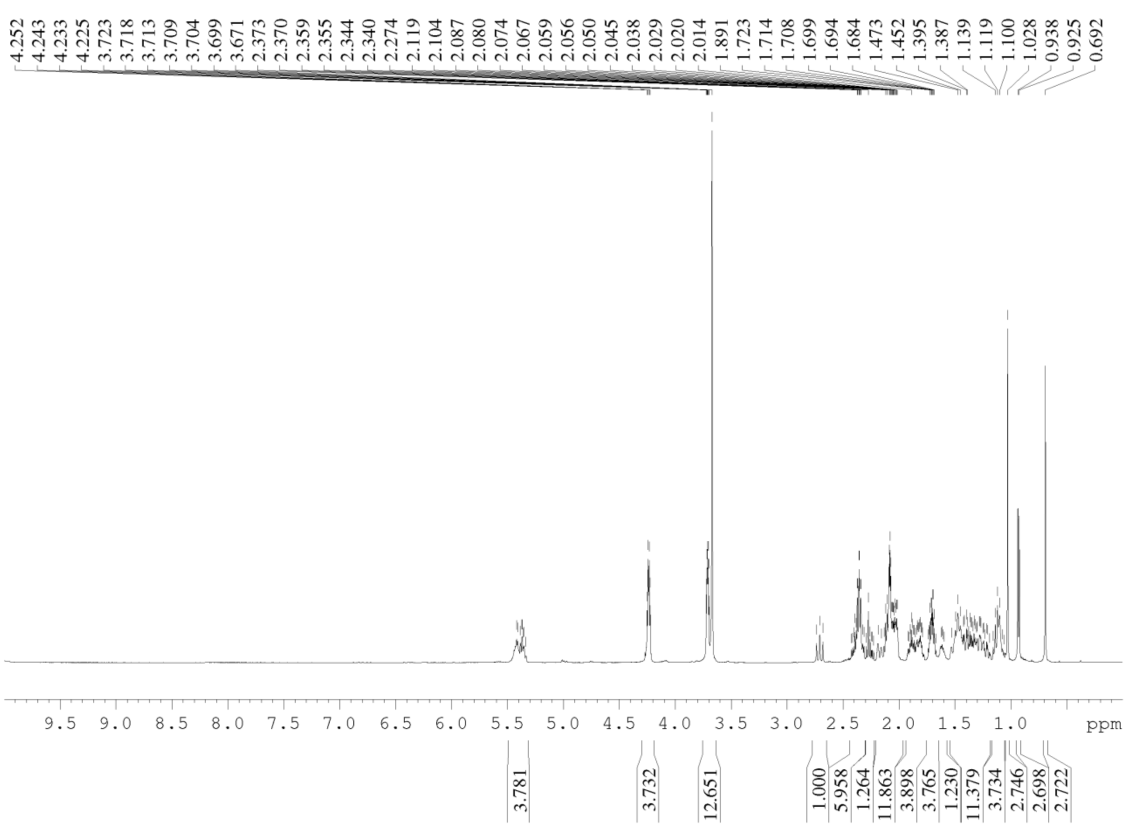

Figure S41. ^{13}C NMR Spectrum of compound **19a** (125 MHz, $CDCl_3$)

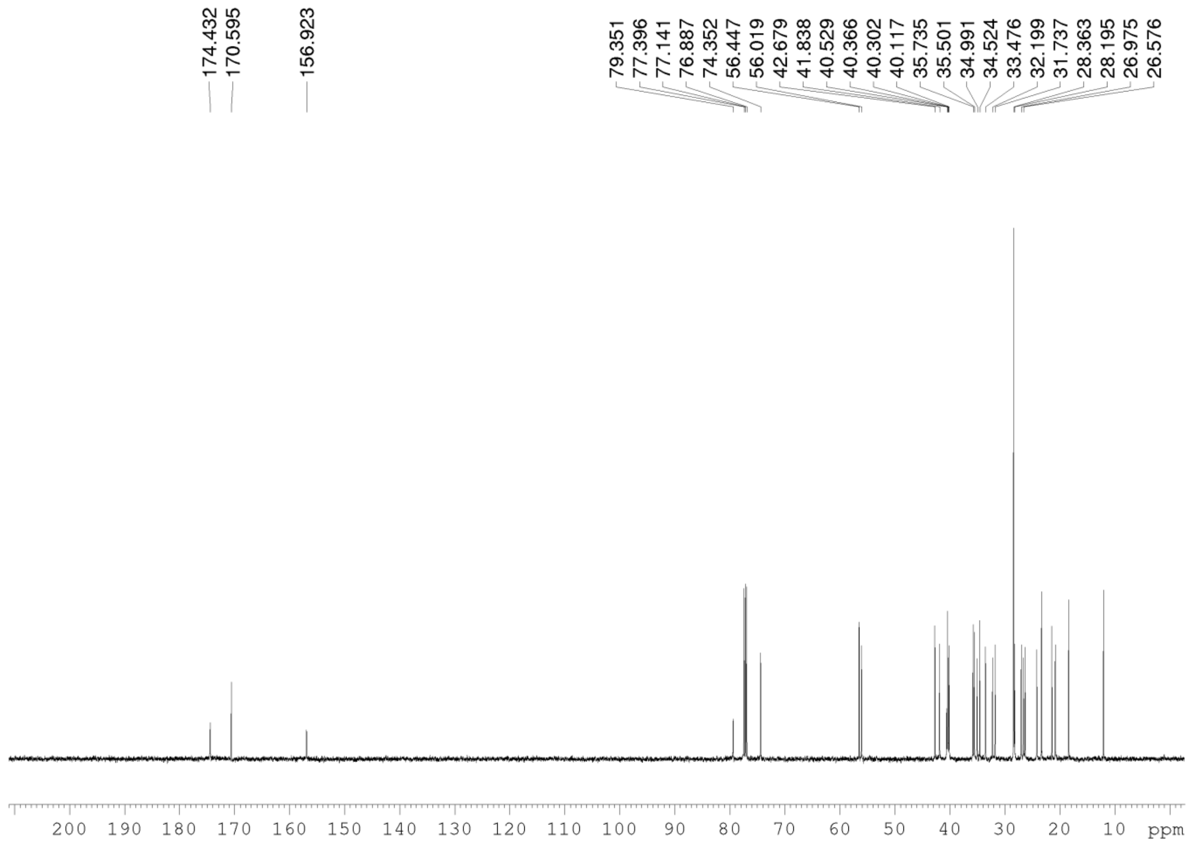

Figure S42. ^{1}H NMR Spectrum of compound **19a** (500 MHz, $CDCl_3$)

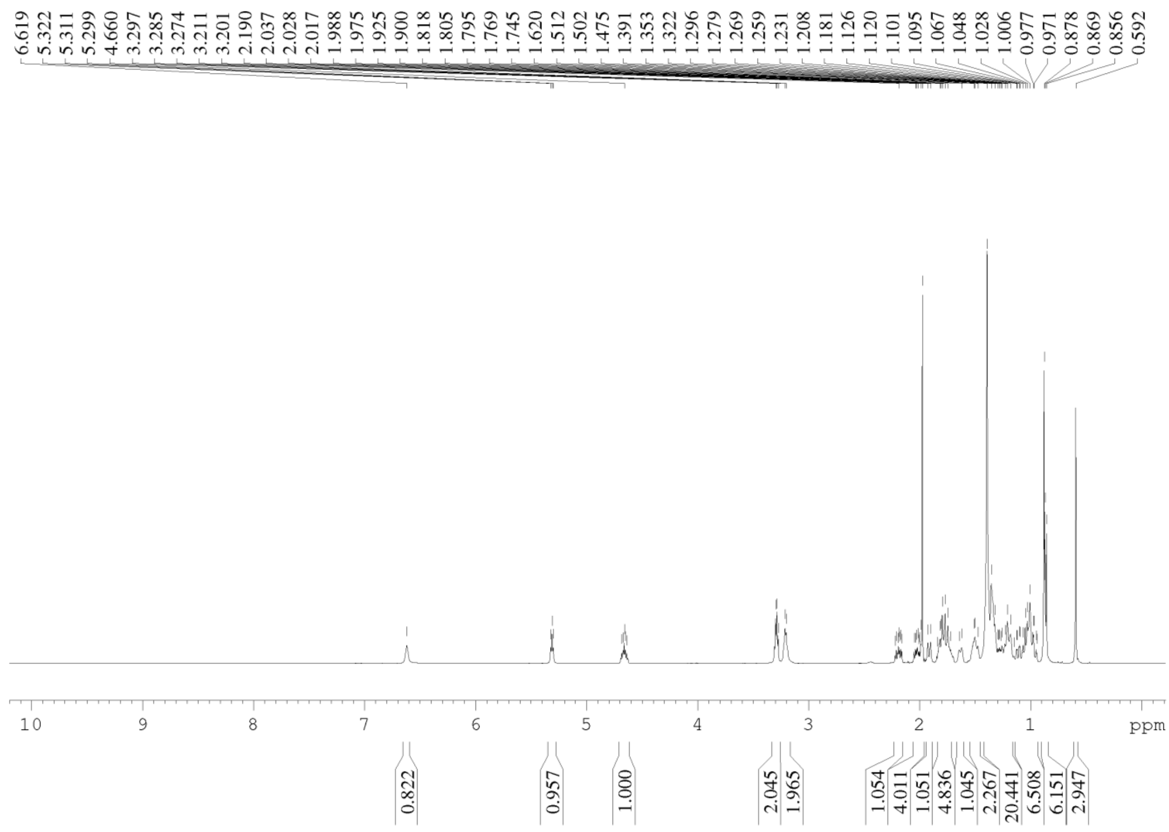

Figure S43. ^{13}C NMR Spectrum of compound **19b** (125 MHz, $CDCl_3$)

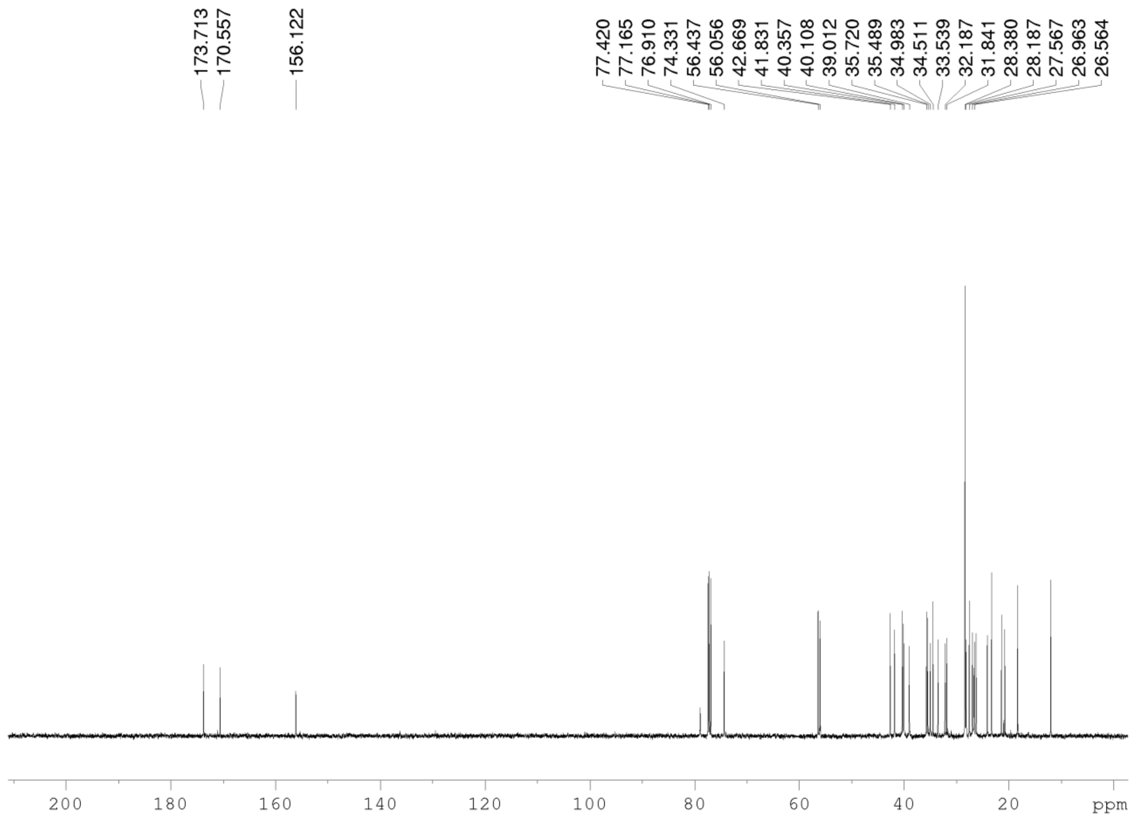

Figure S44. ^{1}H NMR Spectrum of compound **19b** (500 MHz, $CDCl_3$)

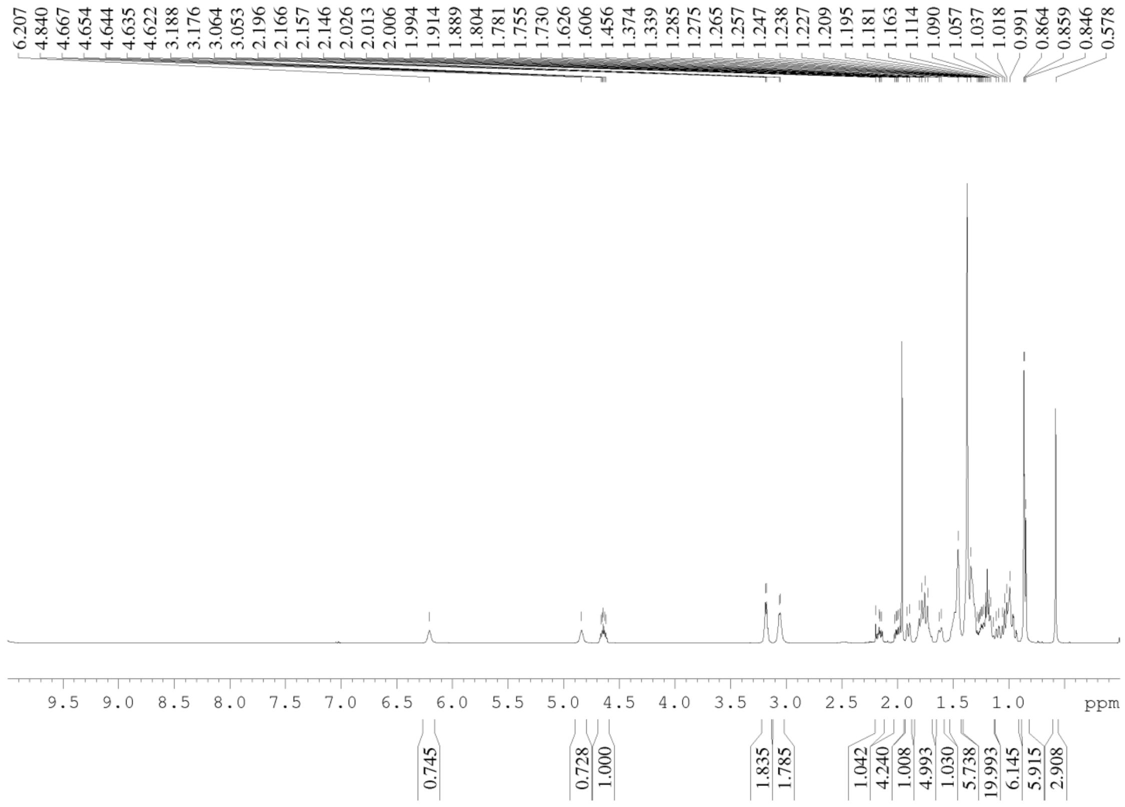

Figure S45. ^{13}C NMR Spectrum of compound **19c** (125 MHz, $CDCl_3$)

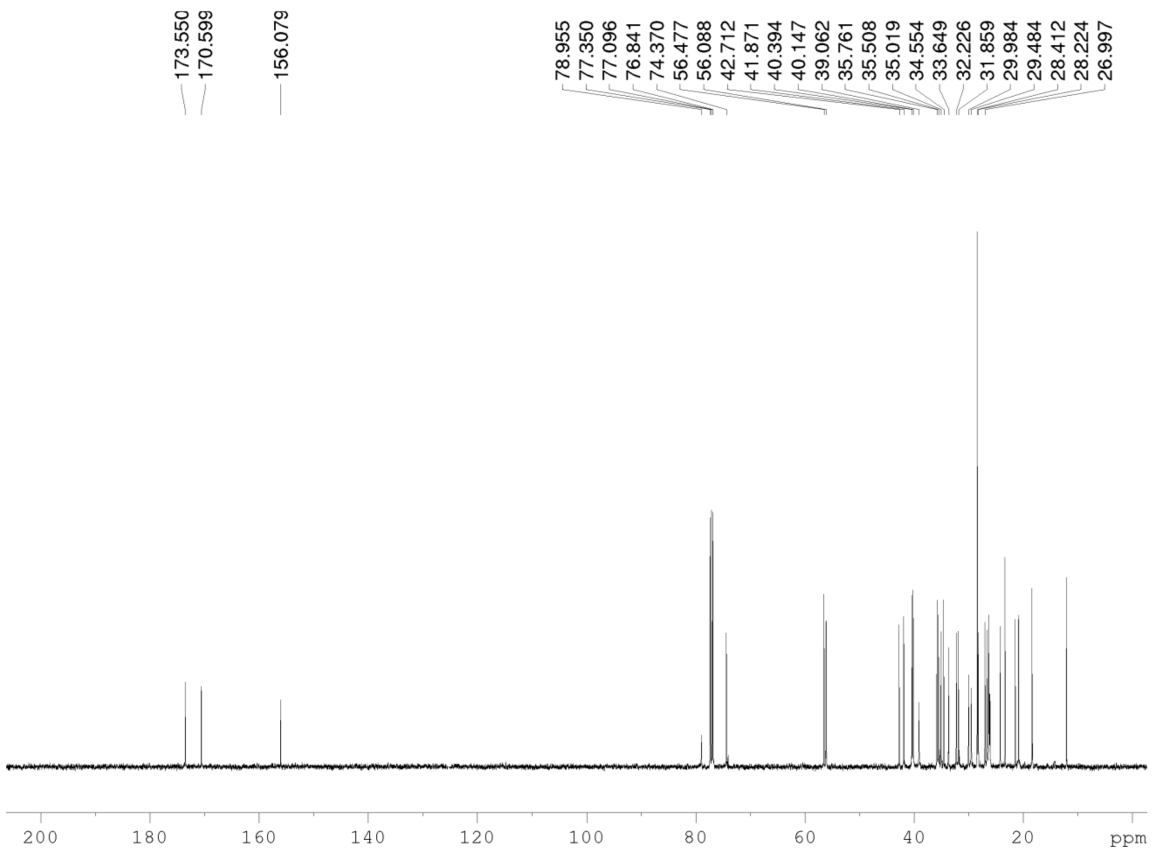

Figure S46. ^{1}H NMR Spectrum of compound **19c** (500 MHz, $CDCl_3$)

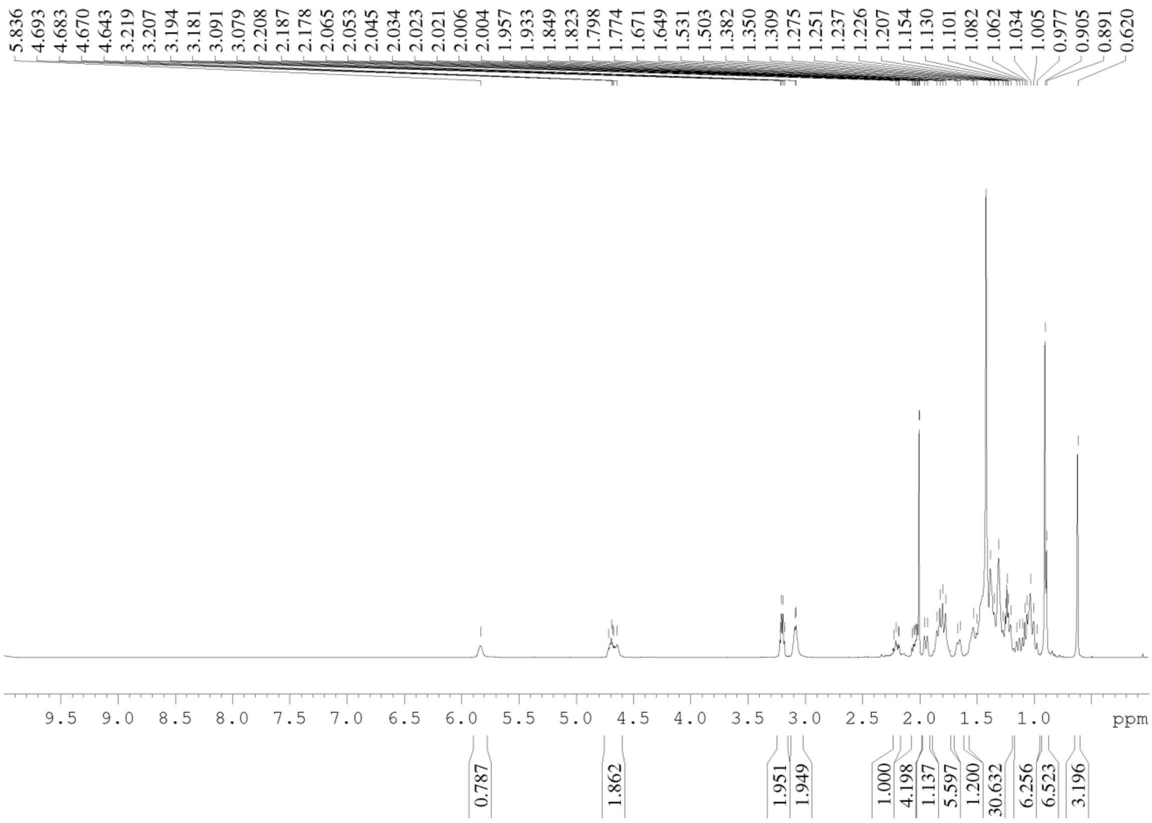

Figure S47. ^{13}C NMR Spectrum of compound **19d** (125 MHz, $CDCl_3$)

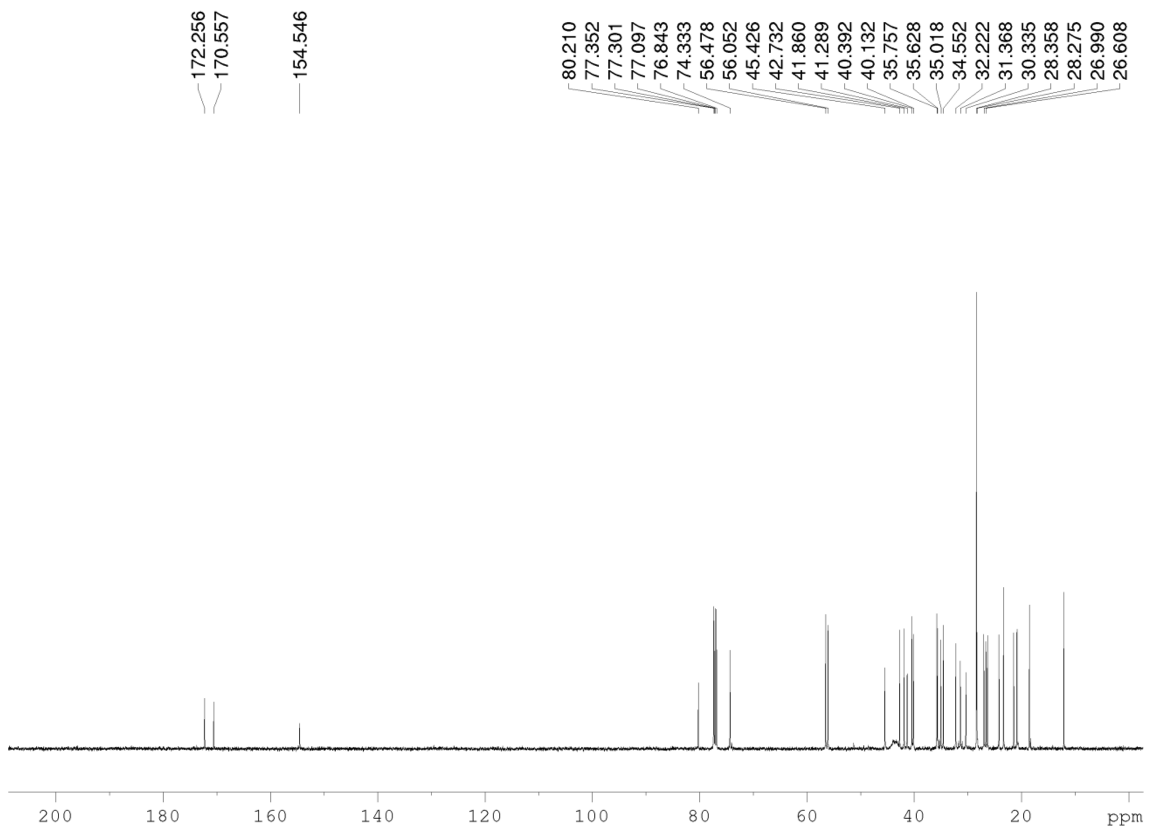

Figure S48. ^{1}H NMR Spectrum of compound **19d** (500 MHz, $CDCl_3$)

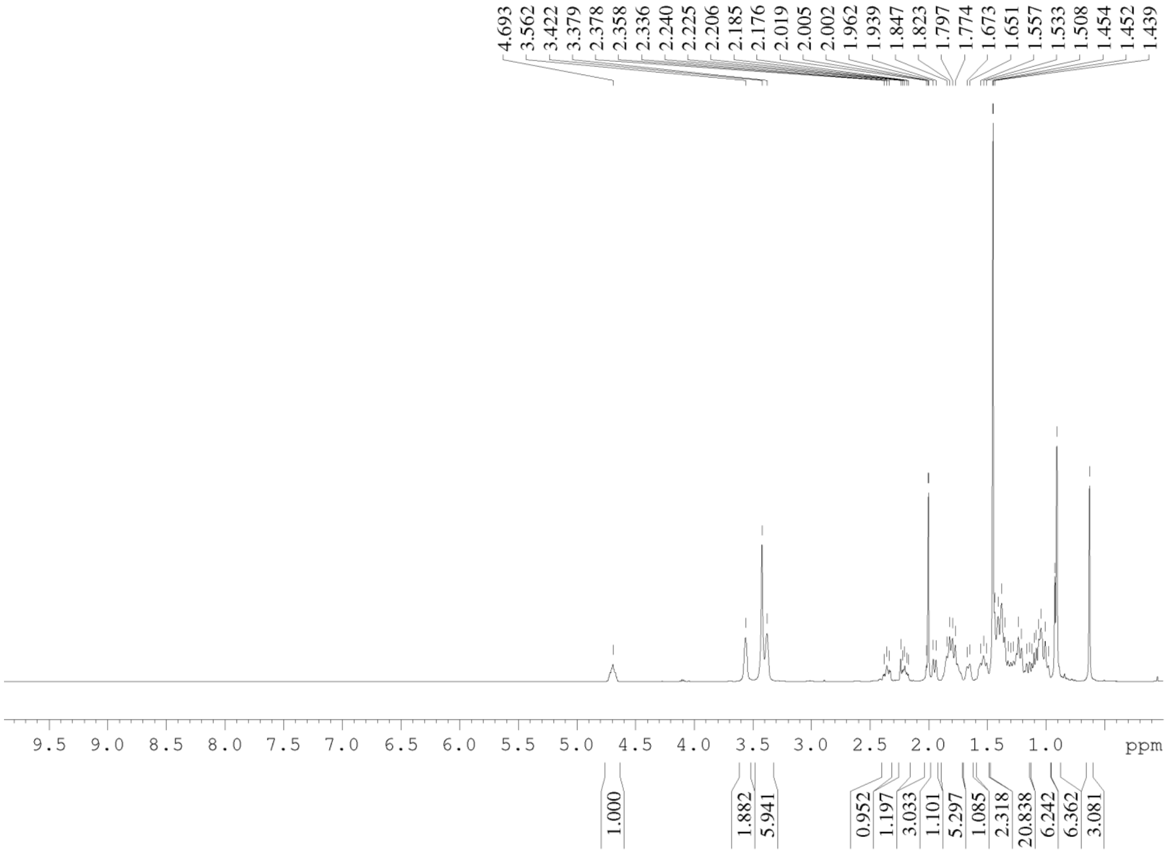

Figure S49. ^{13}C NMR Spectrum of compound **20a** (125 MHz, $CDCl_3$)

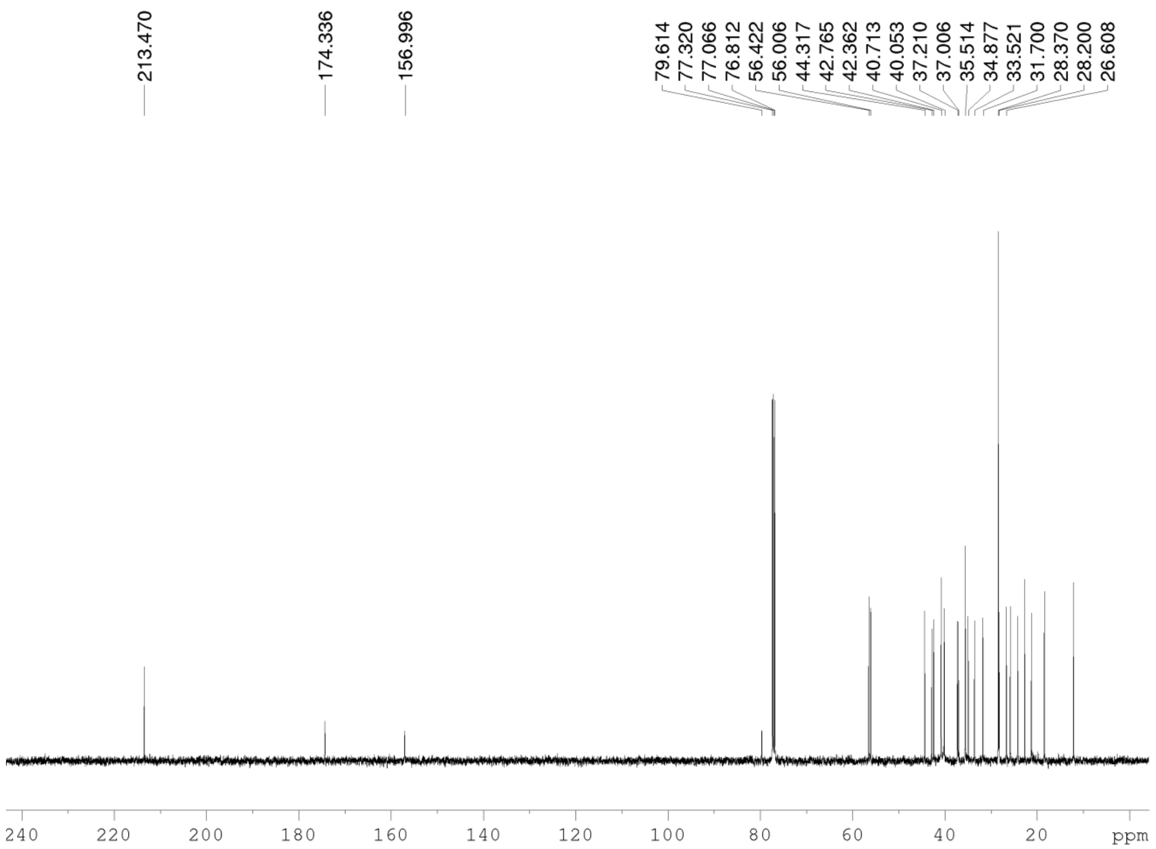

Figure S50. ^{1}H NMR Spectrum of compound **20a** (500 MHz, $CDCl_3$)

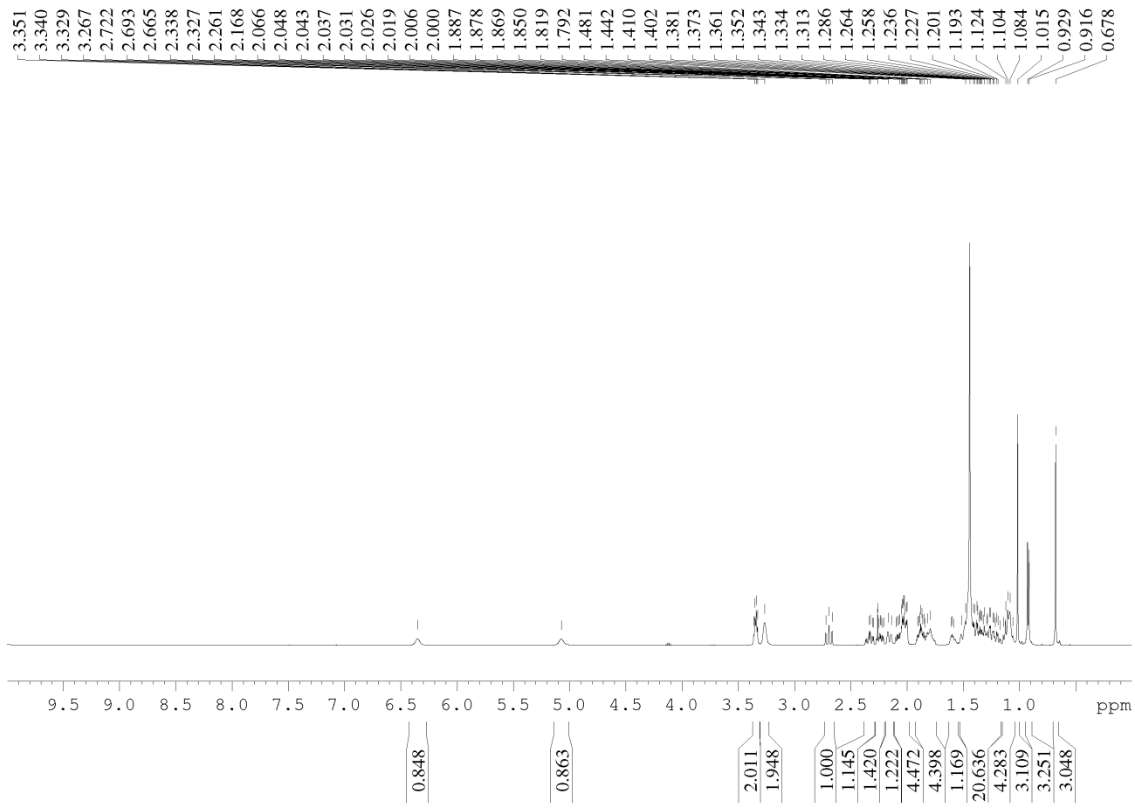

Figure S51. ^{13}C NMR Spectrum of compound **20b** (125 MHz, $CDCl_3$)

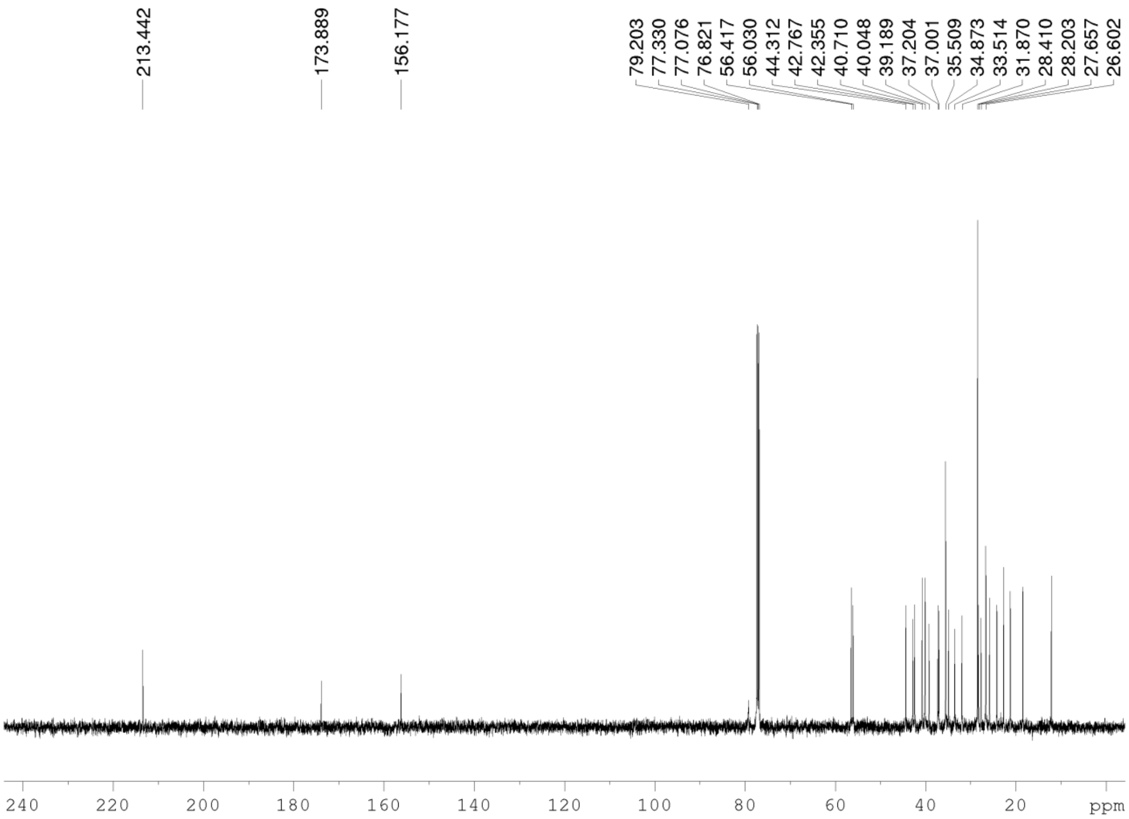

Figure S52. ^{1}H NMR Spectrum of compound **20b** (500 MHz, $CDCl_3$)

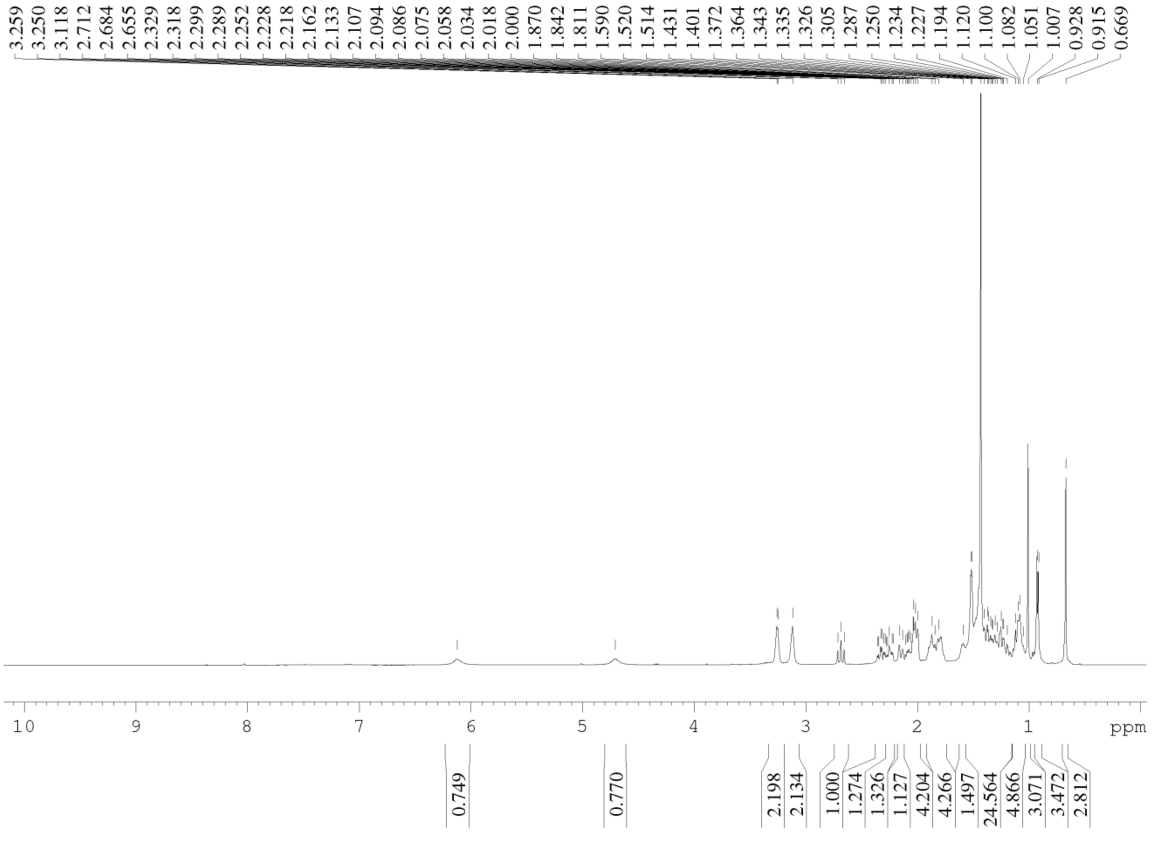

Figure S53. ^{13}C NMR Spectrum of compound **20c** (125 MHz, $CDCl_3$)

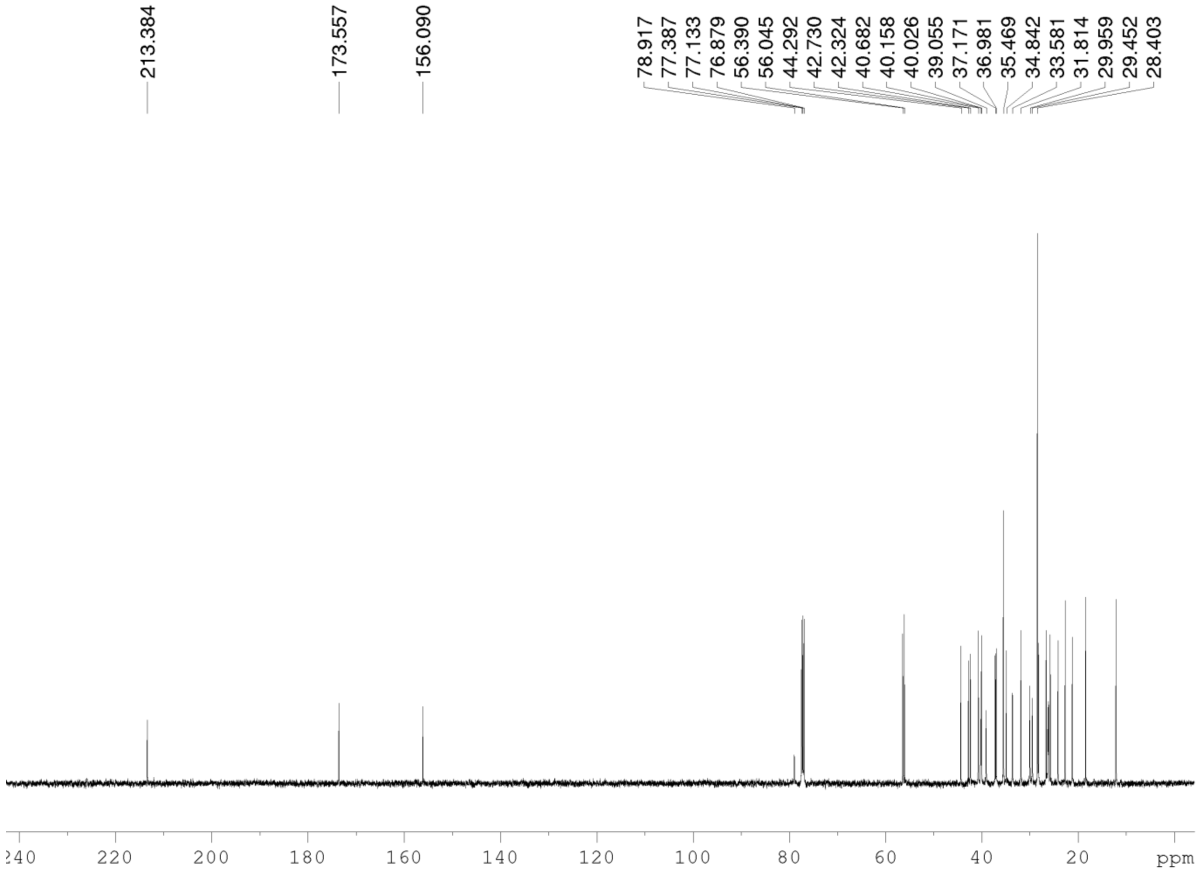

Figure S54. ^{1}H NMR Spectrum of compound **20c** (500 MHz, $CDCl_3$)

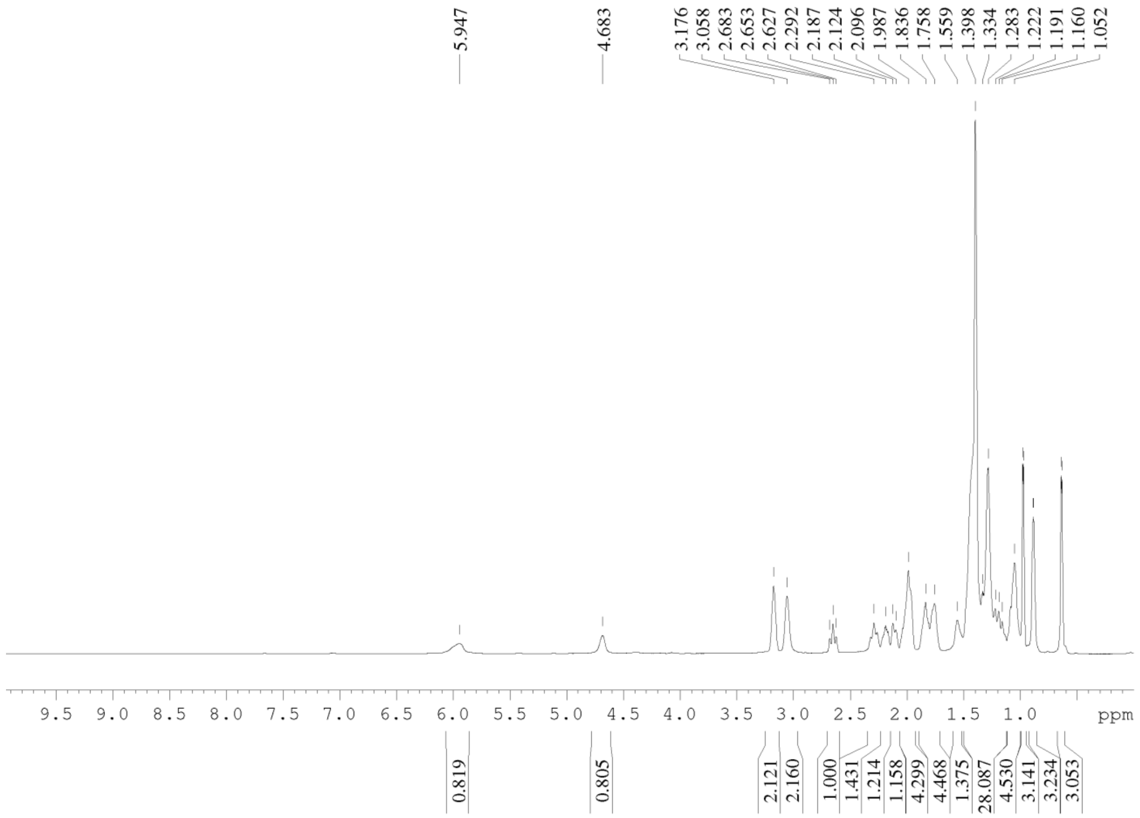

Figure S55. ^{13}C NMR Spectrum of compound **20d** (125 MHz, $CDCl_3$)

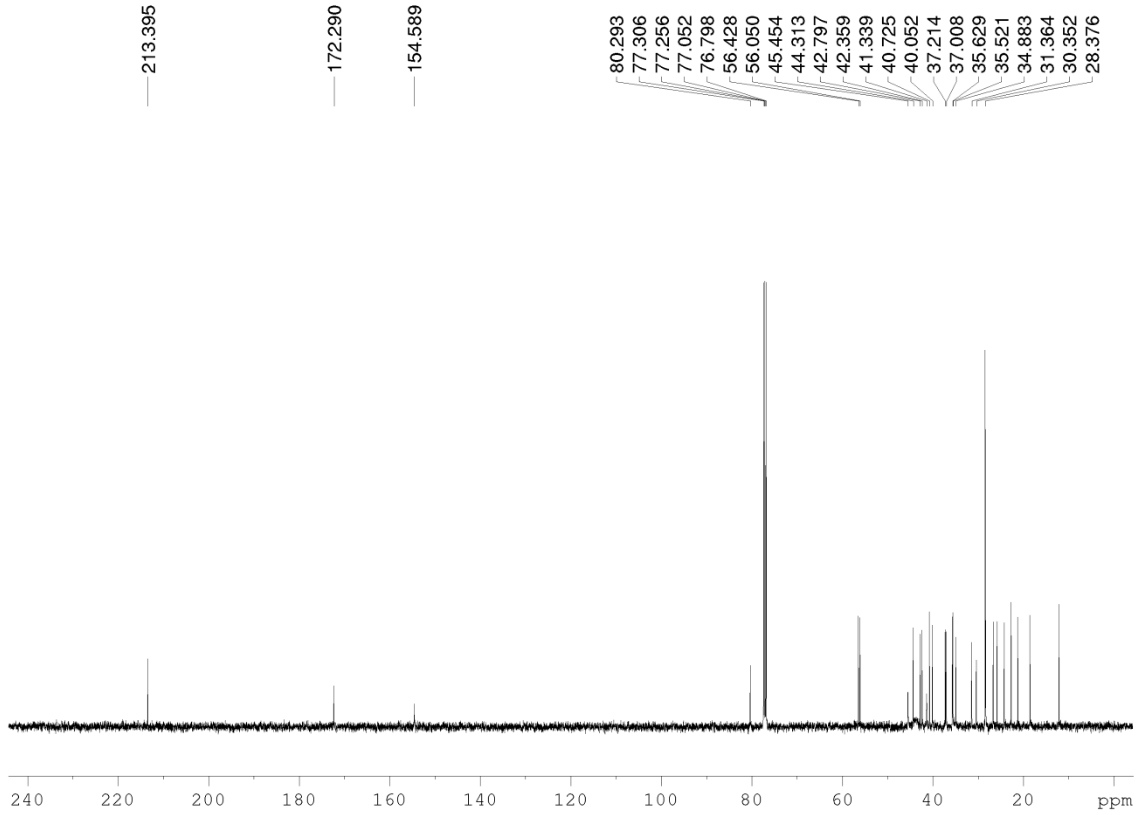

Figure S56. ^{1}H NMR Spectrum of compound **20d** (500 MHz, $CDCl_3$)

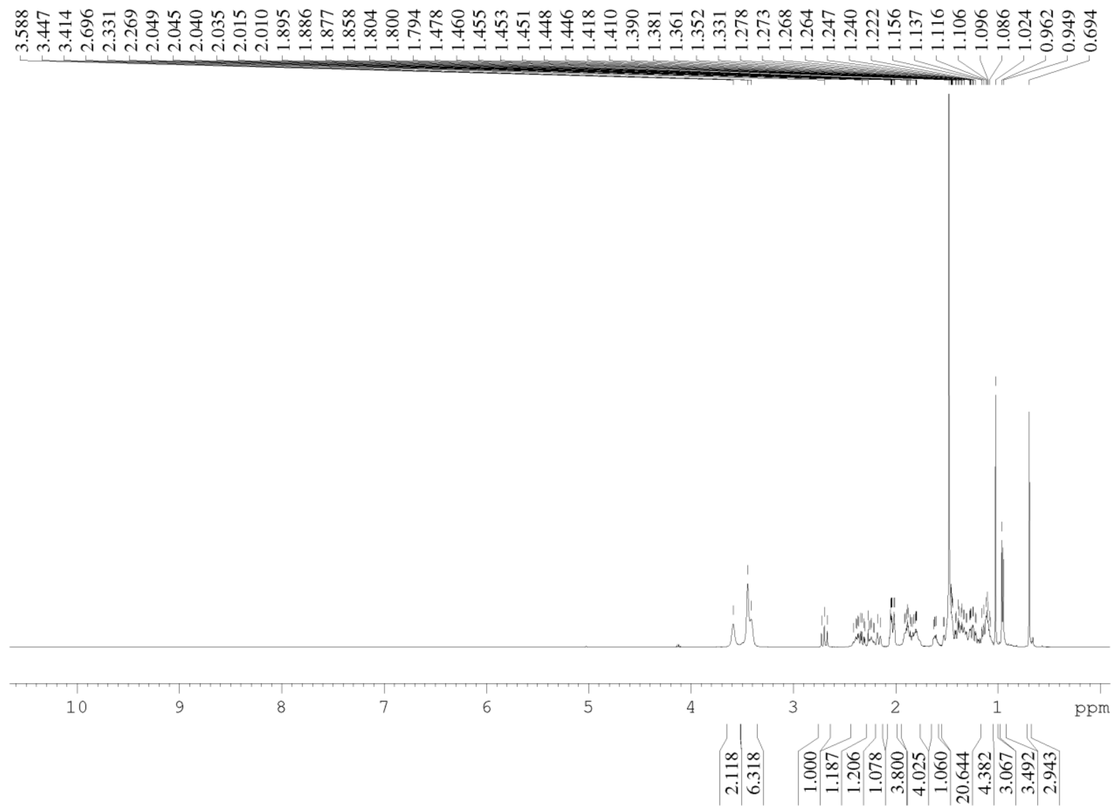

Figure S57. ^{13}C NMR Spectrum of compound **21a** (125 MHz, $CDCl_3$)

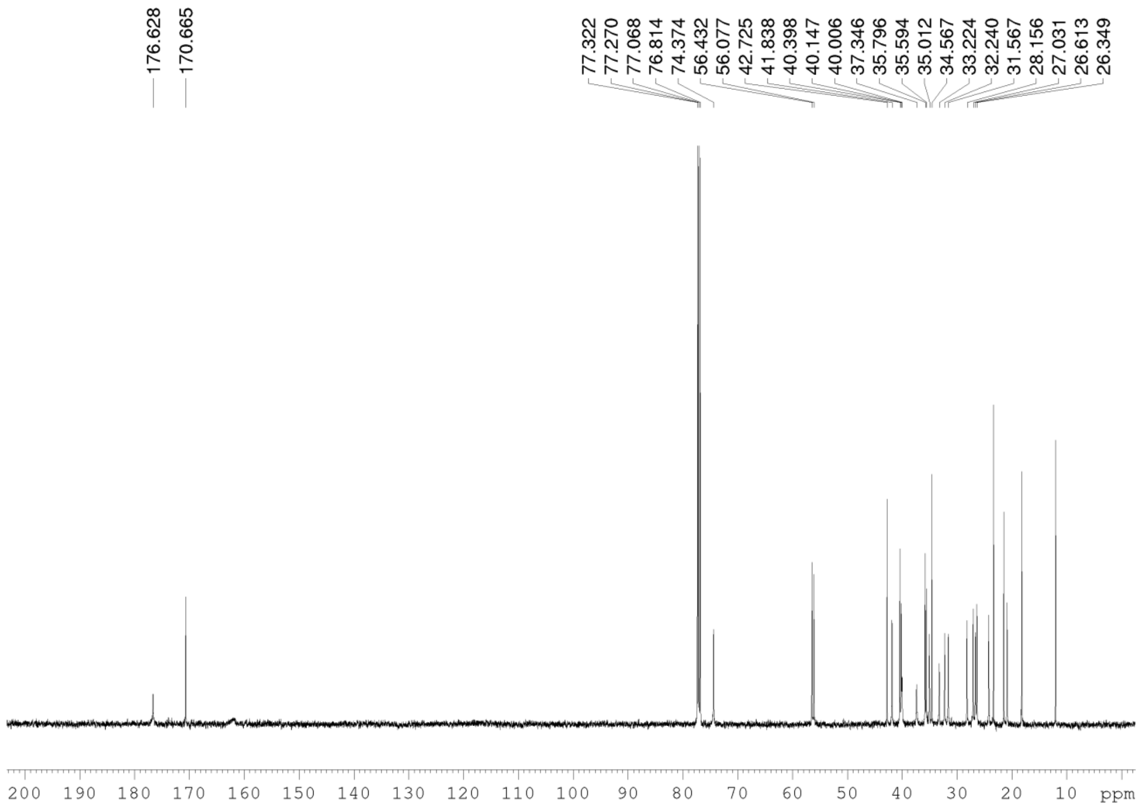

Figure S58. ^{1}H NMR Spectrum of compound **21a** (500 MHz, $CDCl_3$)

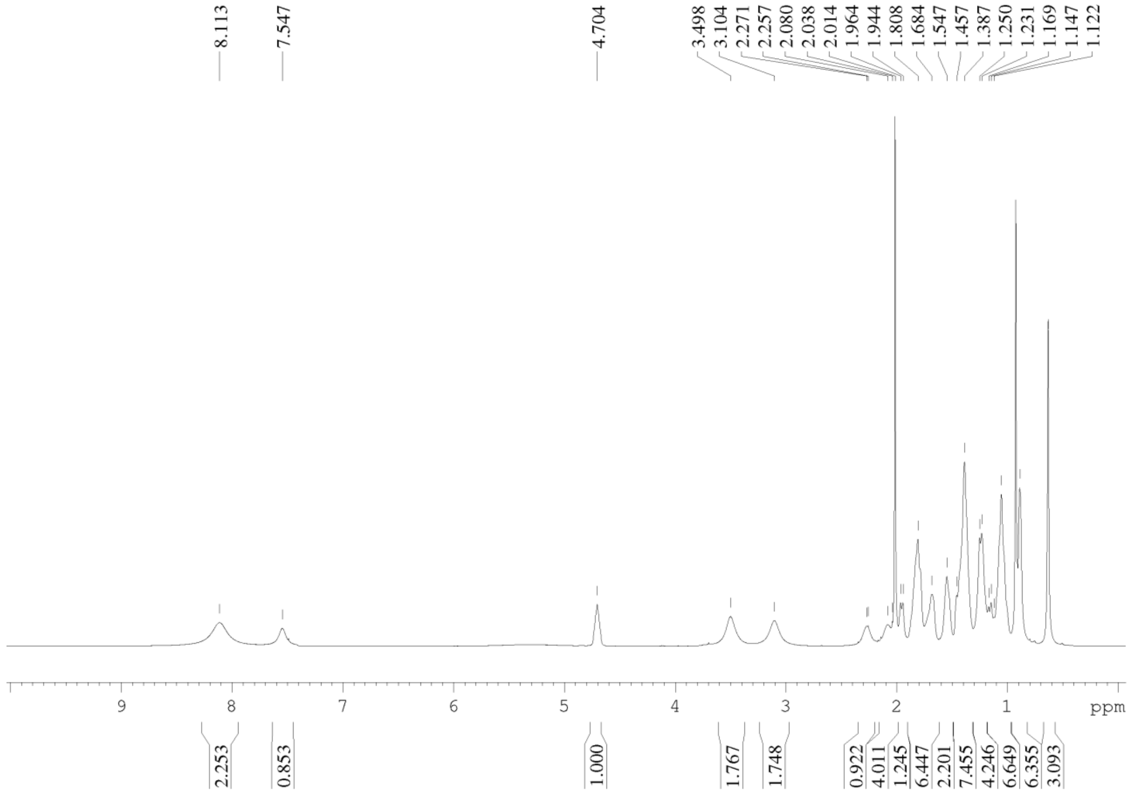

Figure S59. ^{13}C NMR Spectrum of compound **21b** (125 MHz, $CDCl_3$)

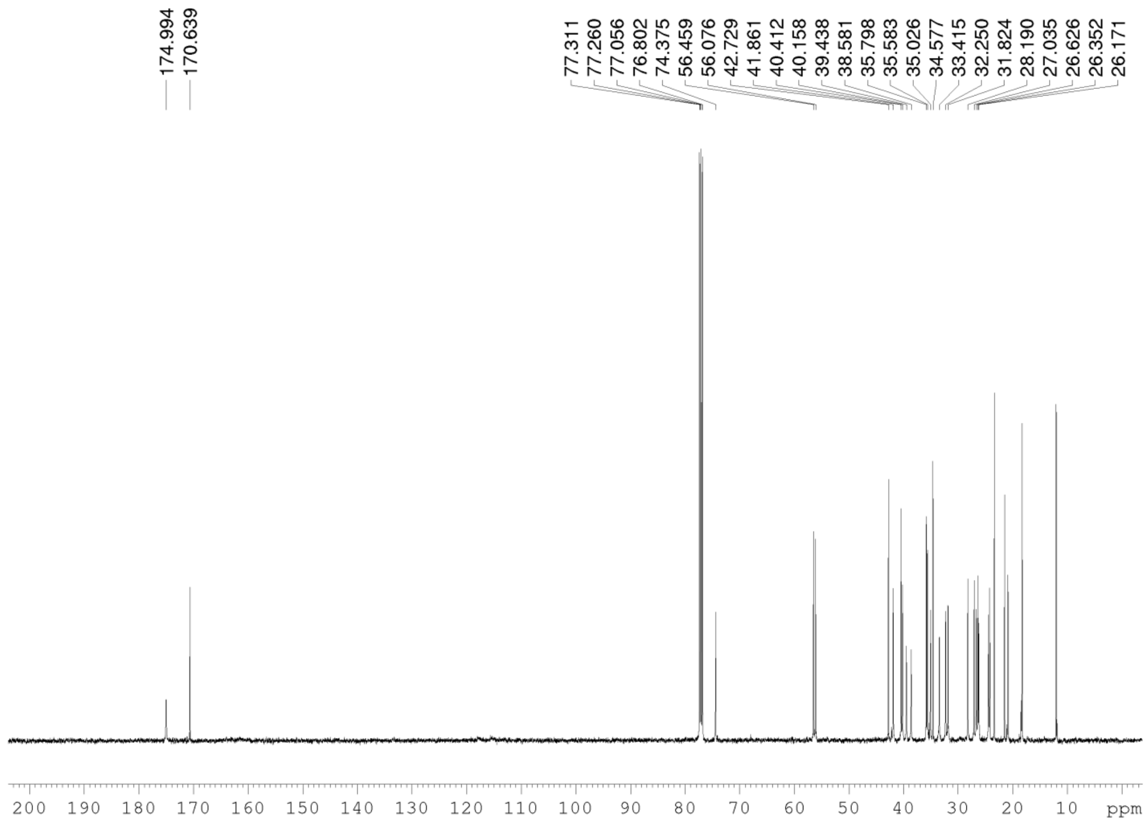

Figure S60. ^{1}H NMR Spectrum of compound **21b** (500 MHz, $CDCl_3$)

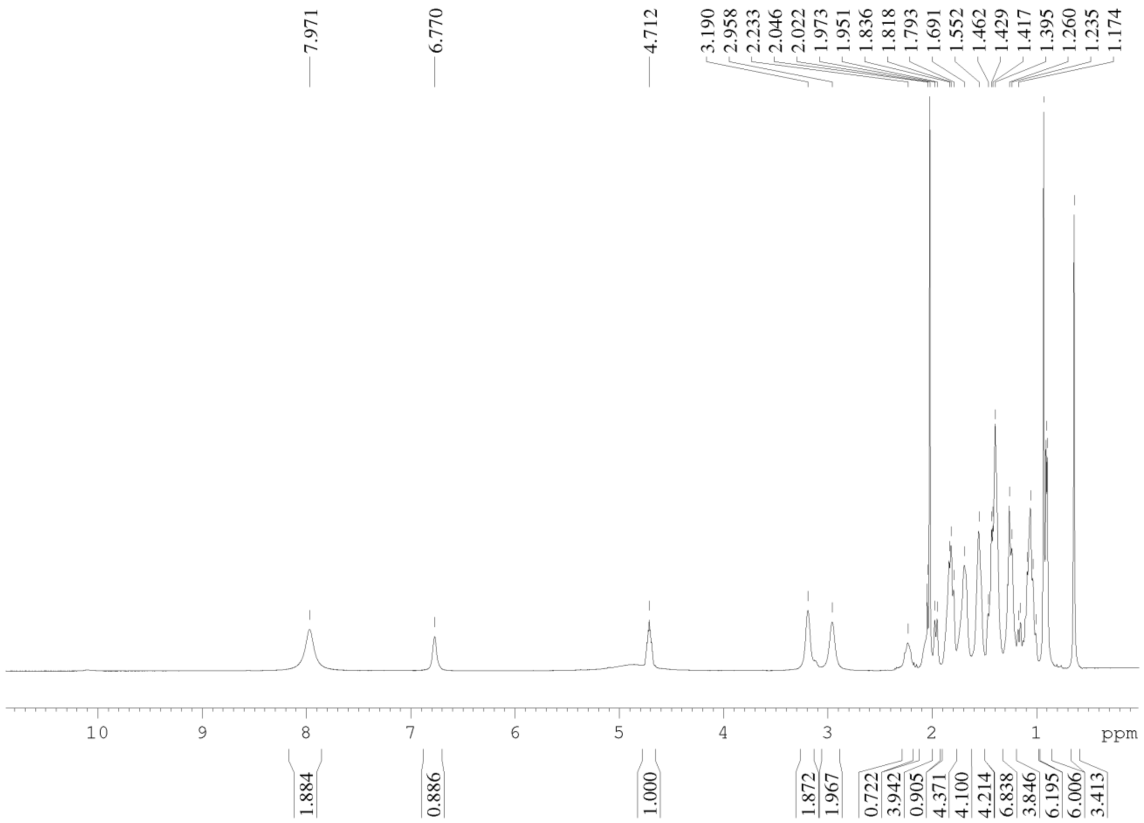

Figure S61. ^{13}C NMR Spectrum of compound **21c** (125 MHz, $CDCl_3$)

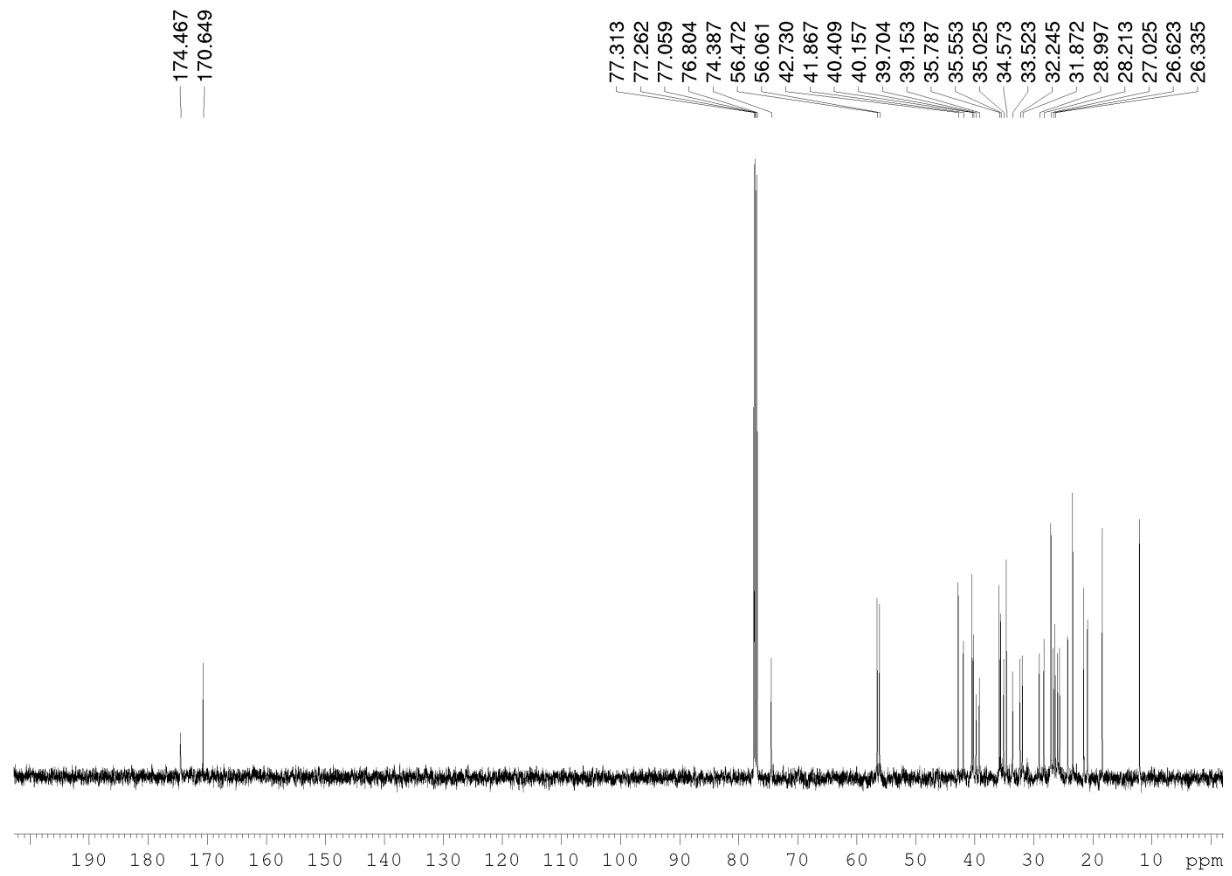

Figure S62. ^{1}H NMR Spectrum of compound **21c** (500 MHz, $CDCl_3$)

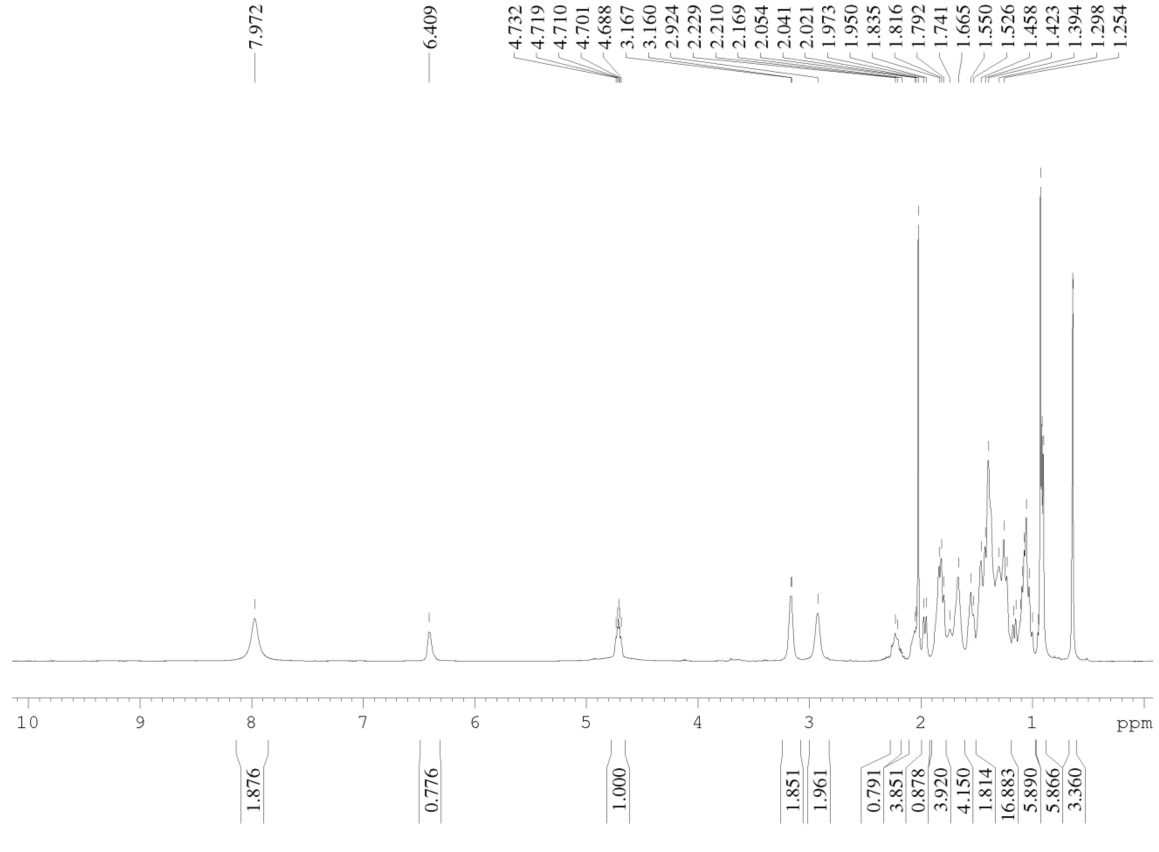

Figure S63. ^{13}C NMR Spectrum of compound **21d** (125 MHz, $CDCl_3$)

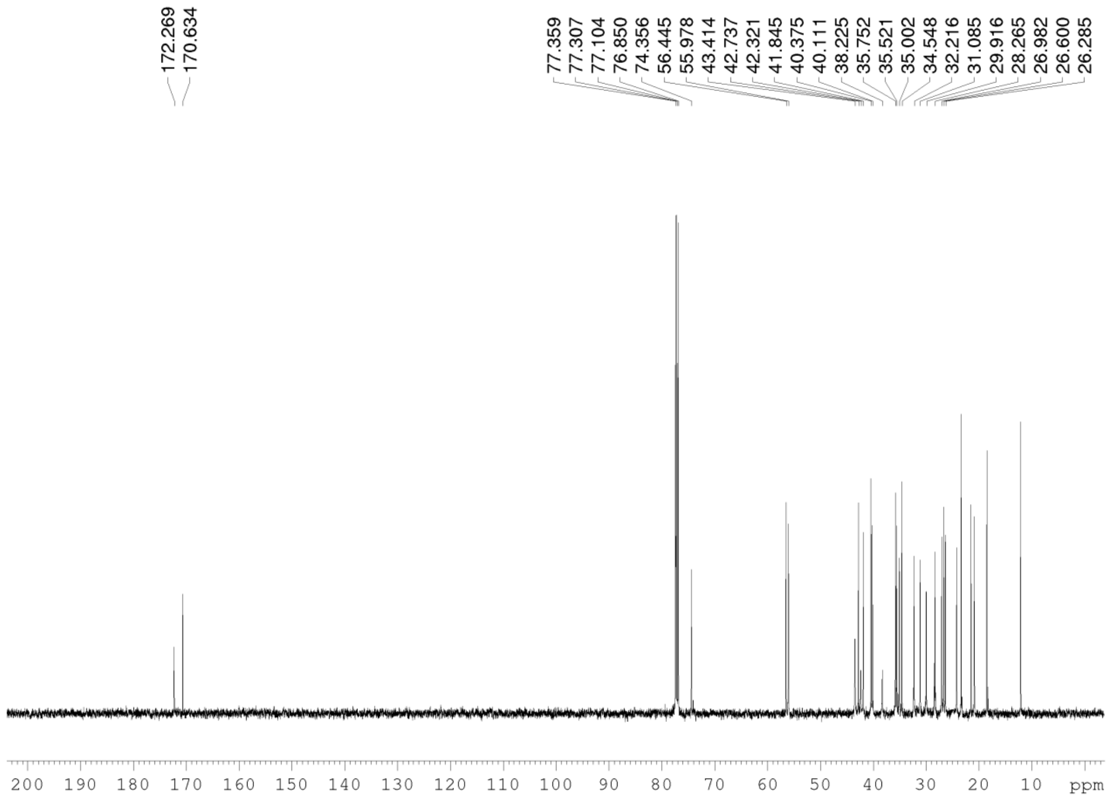

Figure S64. ^{1}H NMR Spectrum of compound **21d** (500 MHz, $CDCl_3$)

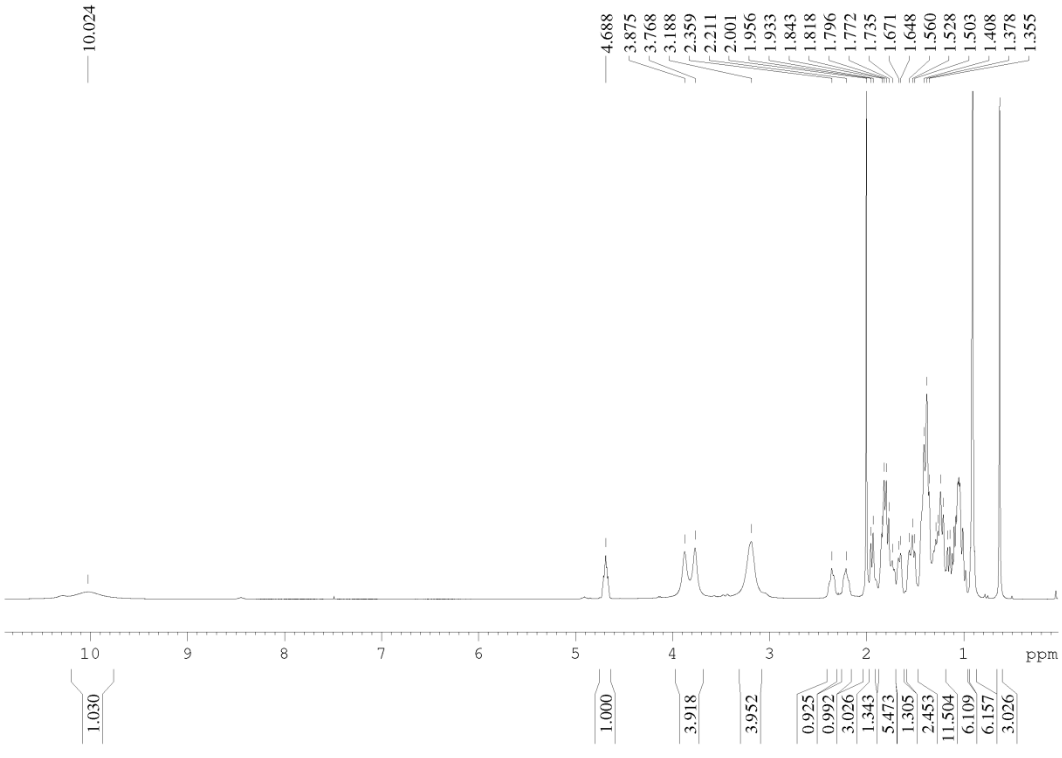

Figure S65. ^{13}C NMR Spectrum of compound **22a** (125 MHz, $CDCl_3$)

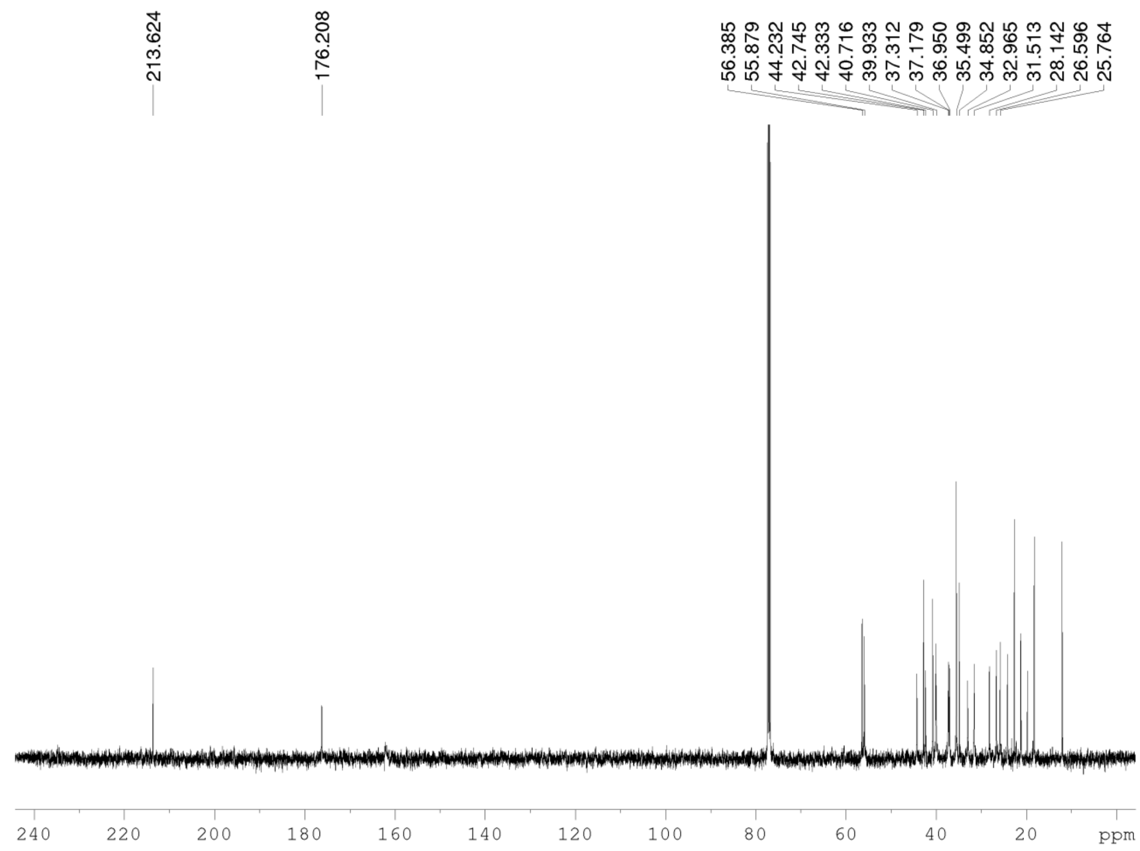

Figure S66. ^{1}H NMR Spectrum of compound **22a** (500 MHz, $CDCl_3$)

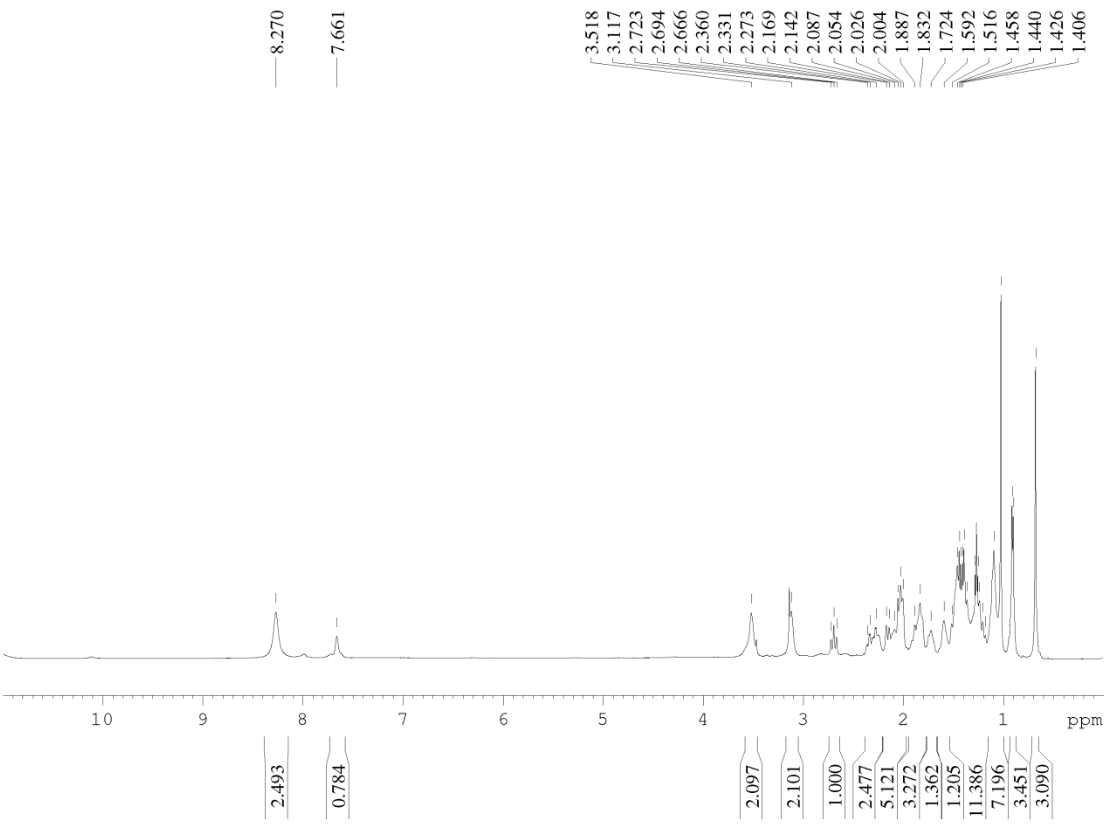

Figure S67. ^{13}C NMR Spectrum of compound **22b** (125 MHz, $CDCl_3$)

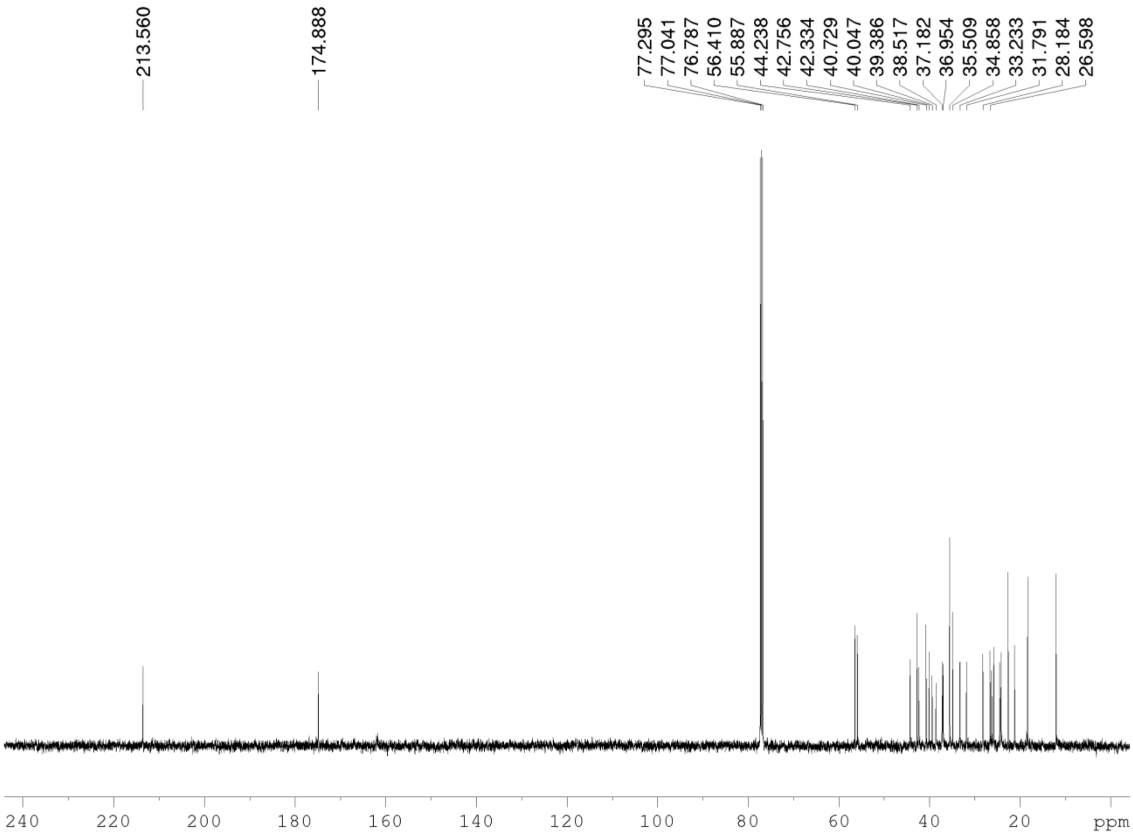

Figure S68. ^{1}H NMR Spectrum of compound **22b** (500 MHz, $CDCl_3$)

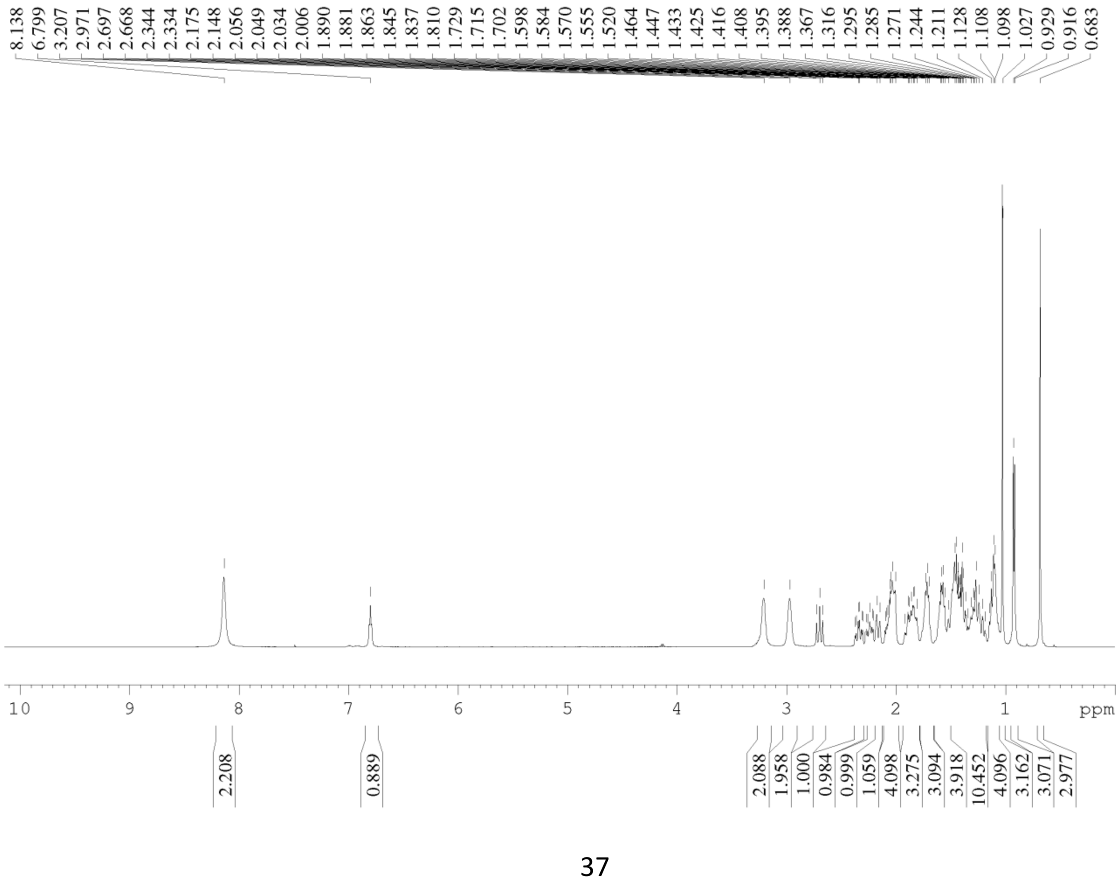

Figure S69. ^{13}C NMR Spectrum of compound **22c** (125 MHz, $CDCl_3$)

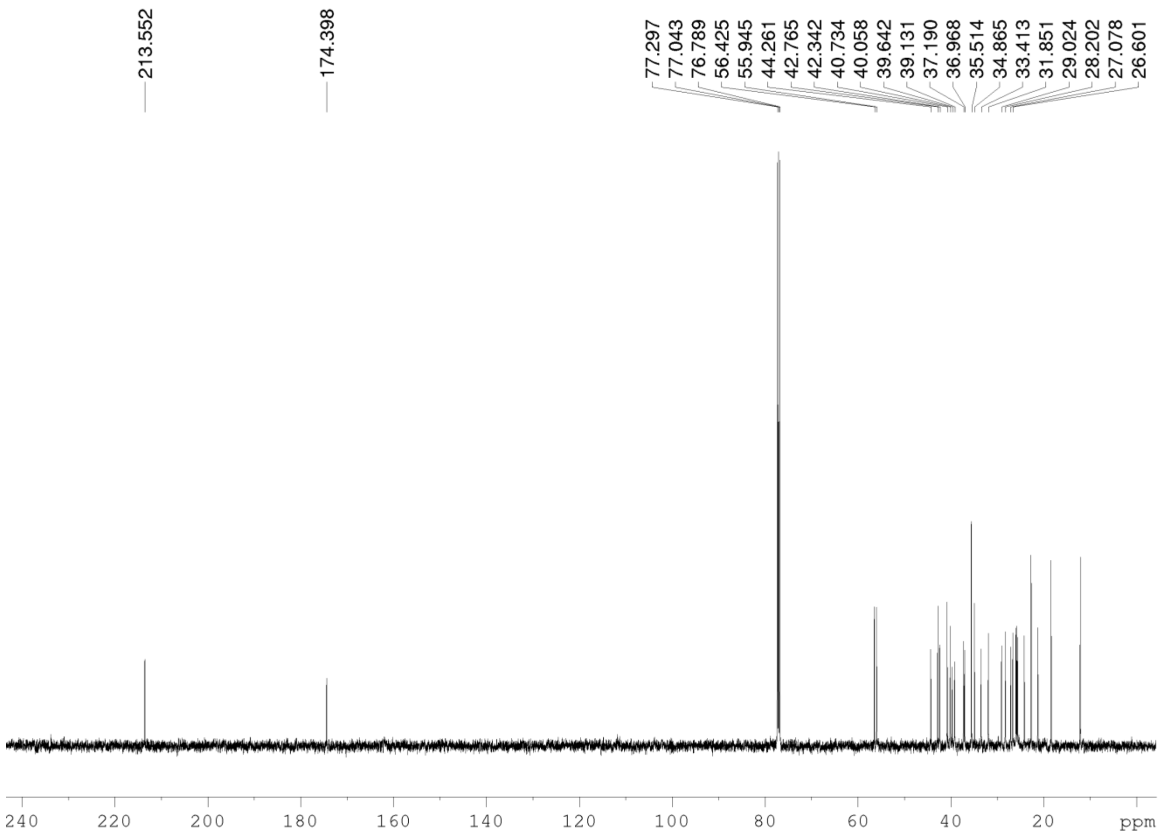

Figure S70. ^{1}H NMR Spectrum of compound **22c** (500 MHz, $CDCl_3$)

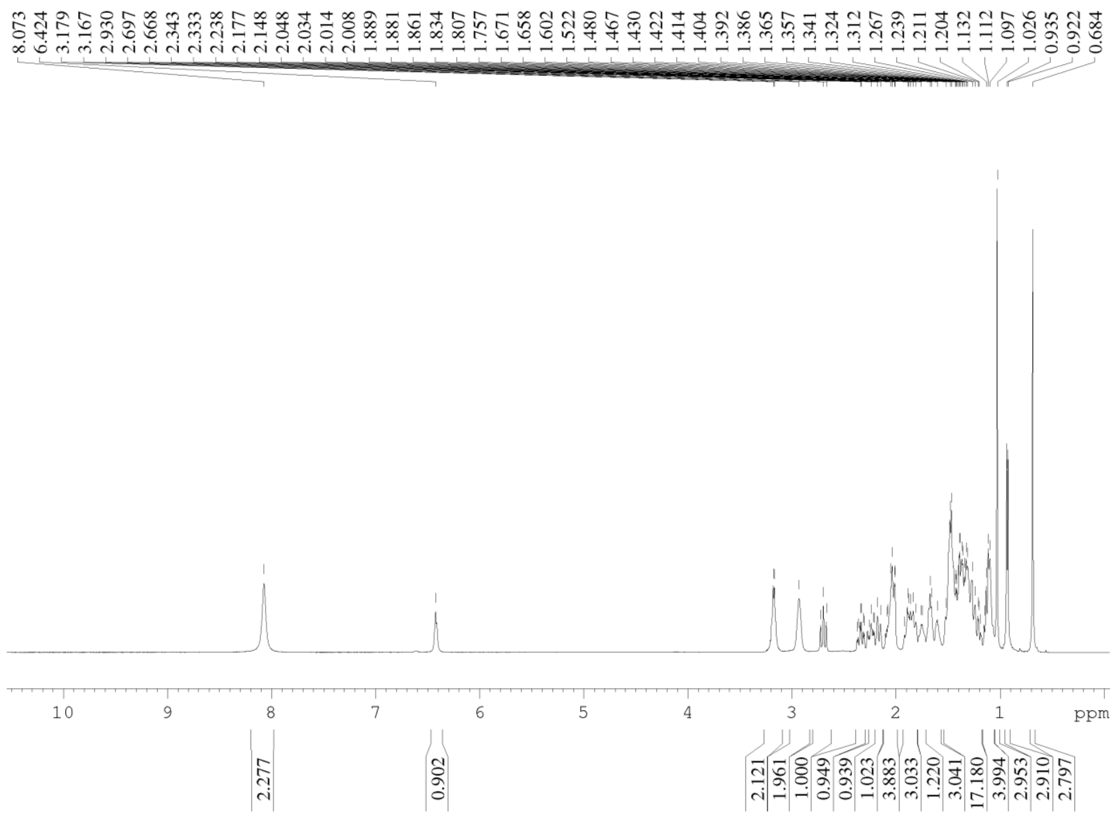

Figure S71. ^{13}C NMR Spectrum of compound **22d** (125 MHz, $CDCl_3$)

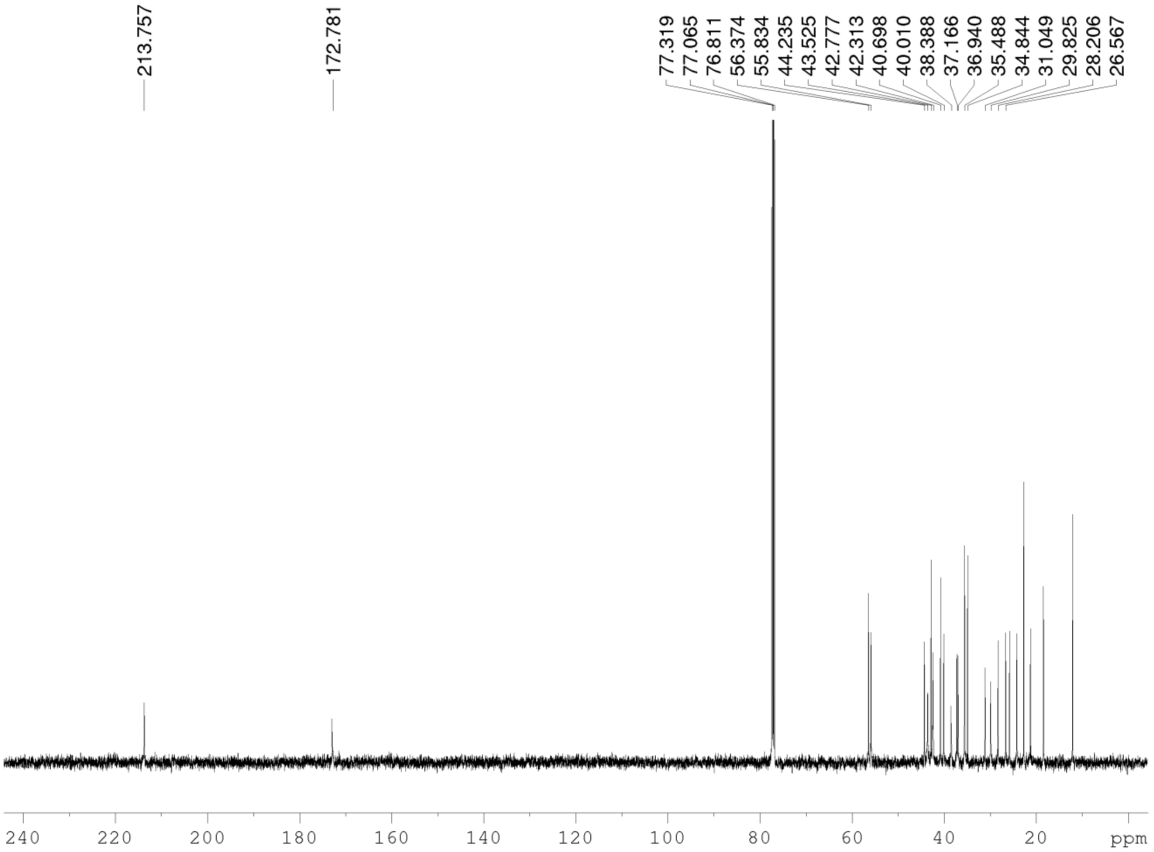

Figure S72. ^{1}H NMR Spectrum of compound **22d** (500 MHz, $CDCl_3$)

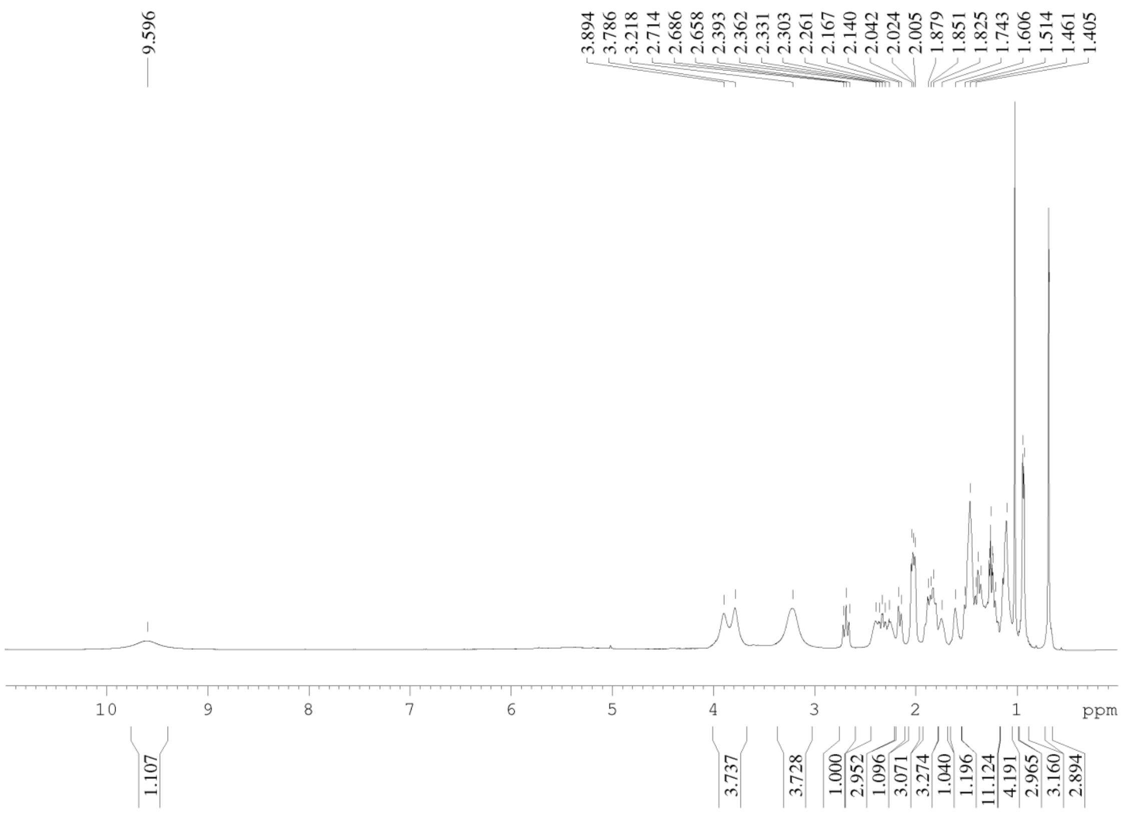

Figure S73. ^{13}C NMR Spectrum of compound **23a** (125 MHz, $CDCl_3$)

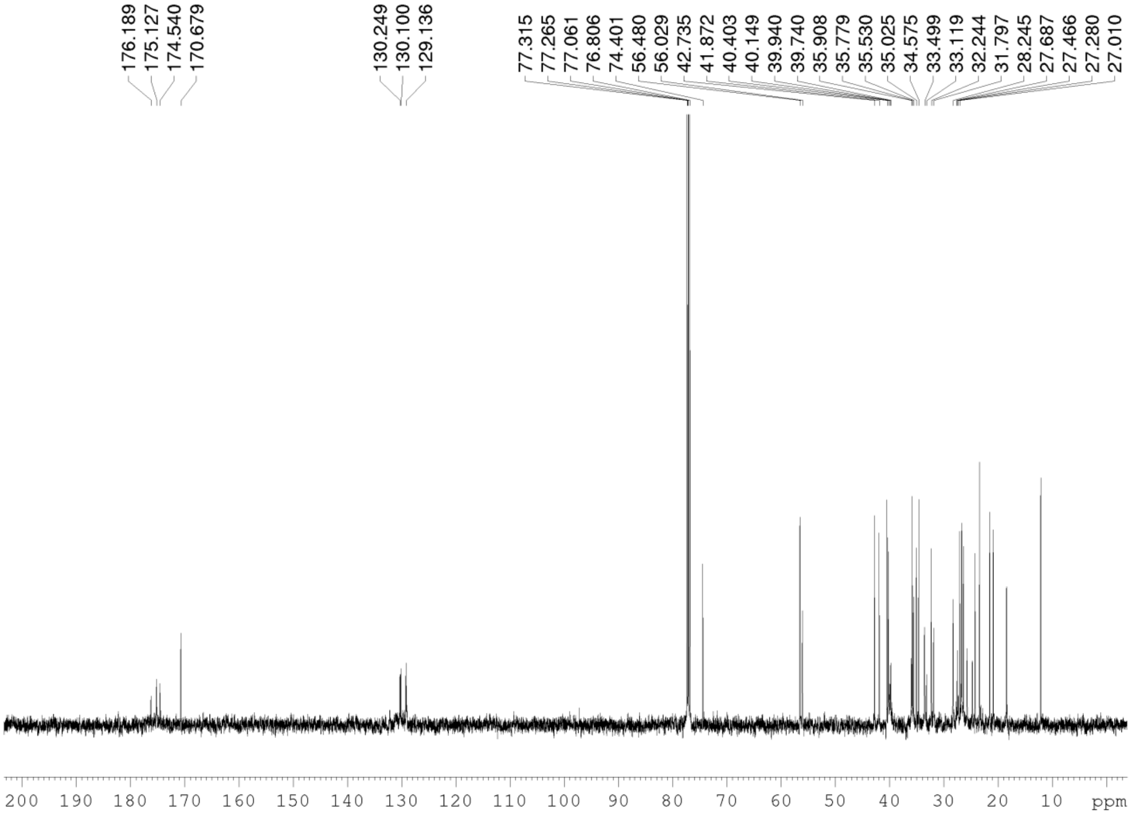

Figure S74. ^{1}H NMR Spectrum of compound **23a** (500 MHz, $CDCl_3$)

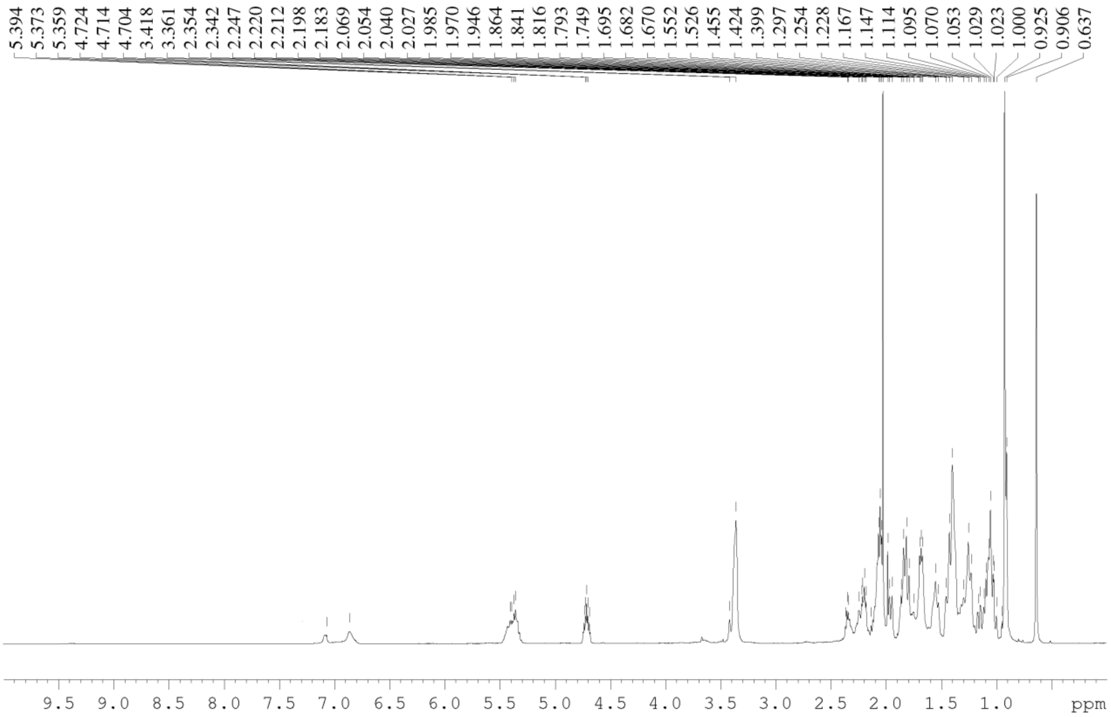

Figure S75. ^{13}C NMR Spectrum of compound **23b** (125 MHz, $CDCl_3$)

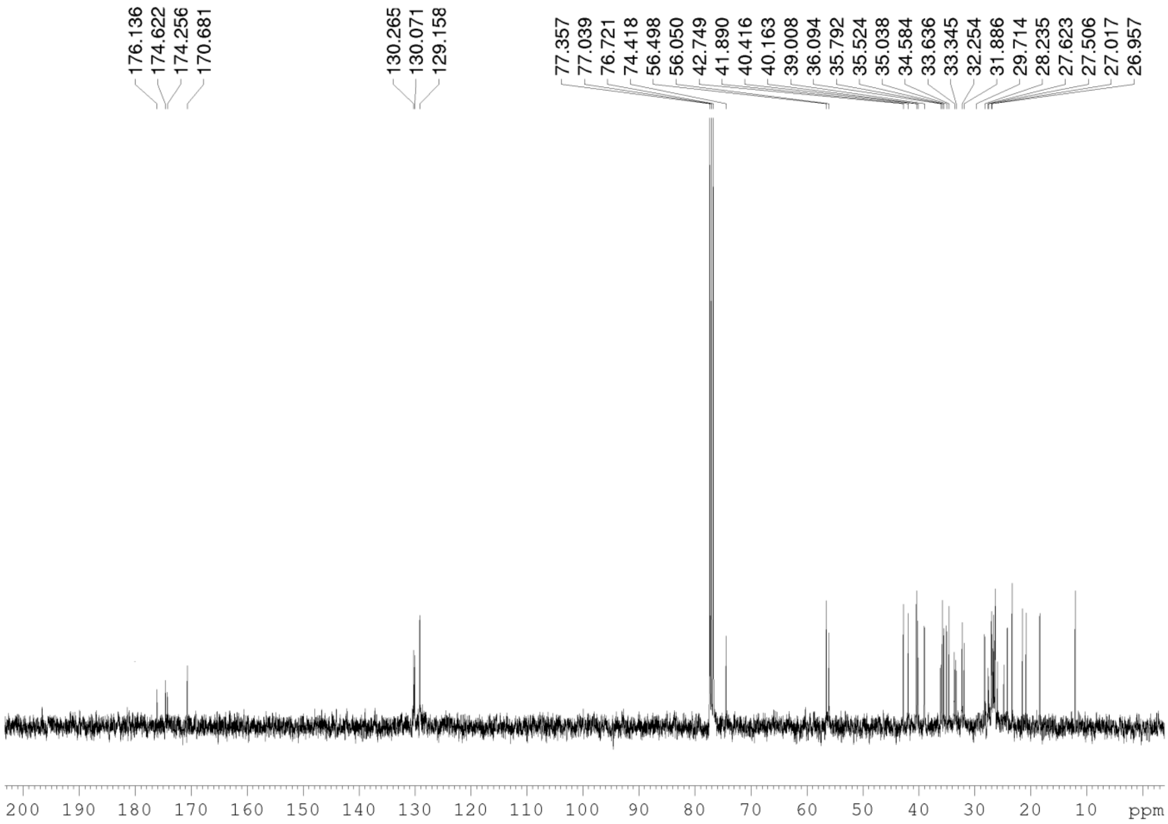

Figure S76. ^{1}H NMR Spectrum of compound **23b** (500 MHz, $CDCl_3$)

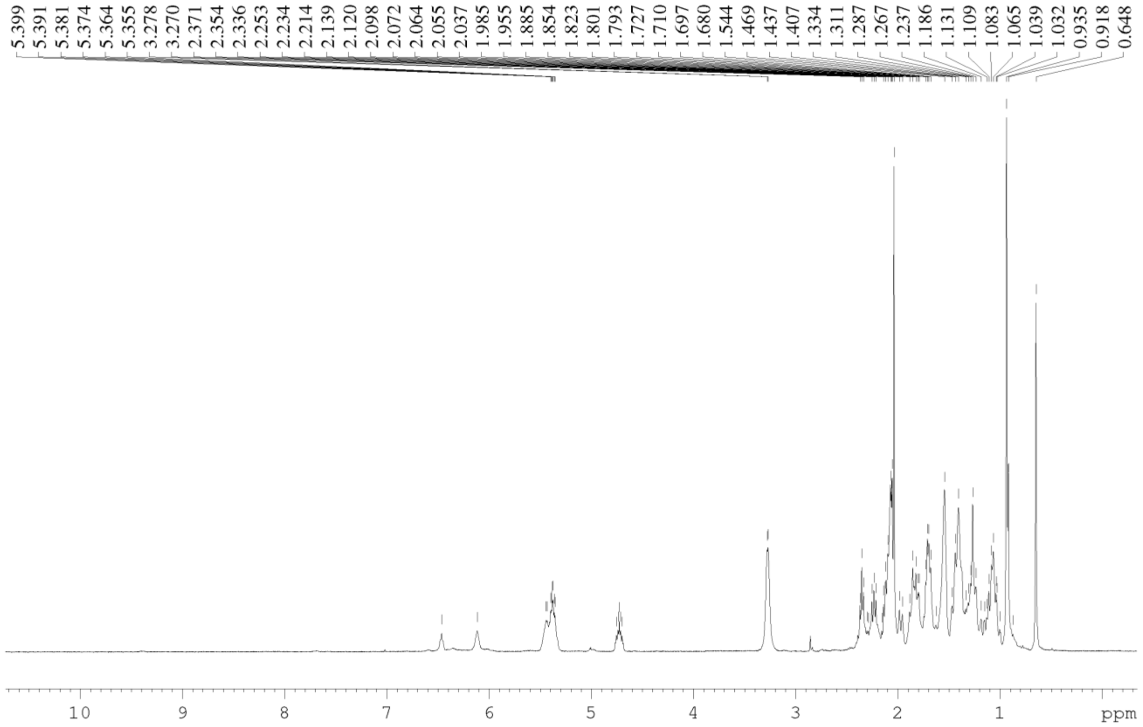

Figure S77. ^{13}C NMR Spectrum of compound **23c** (125 MHz, $CDCl_3$)

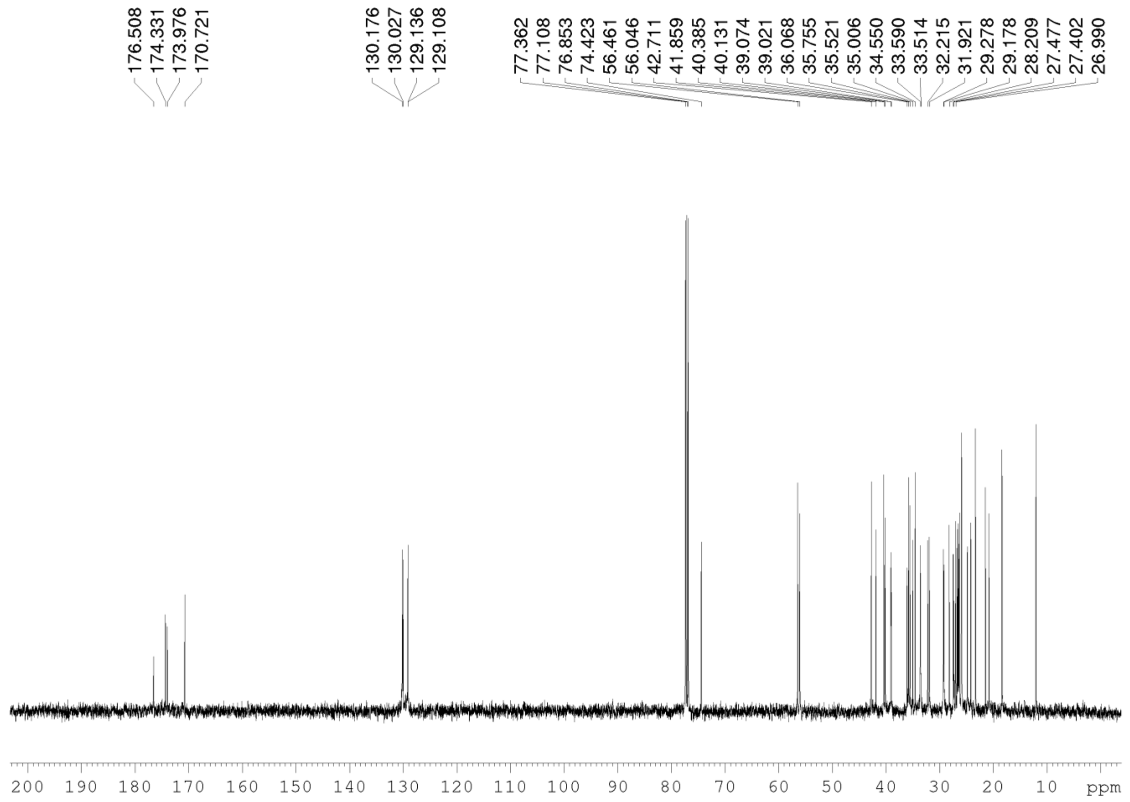

Figure S78. ^{1}H NMR Spectrum of compound **23c** (500 MHz, $CDCl_3$)

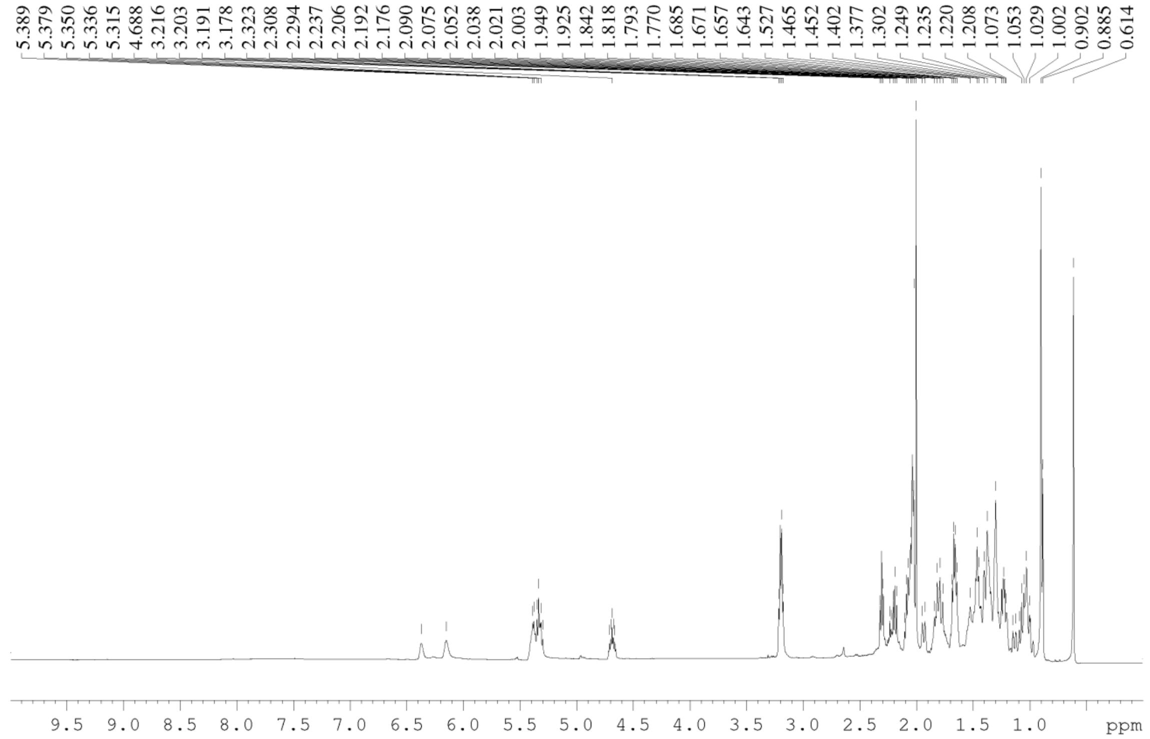

Figure S79. ^{13}C NMR Spectrum of compound **23d** (125 MHz, $CDCl_3$)

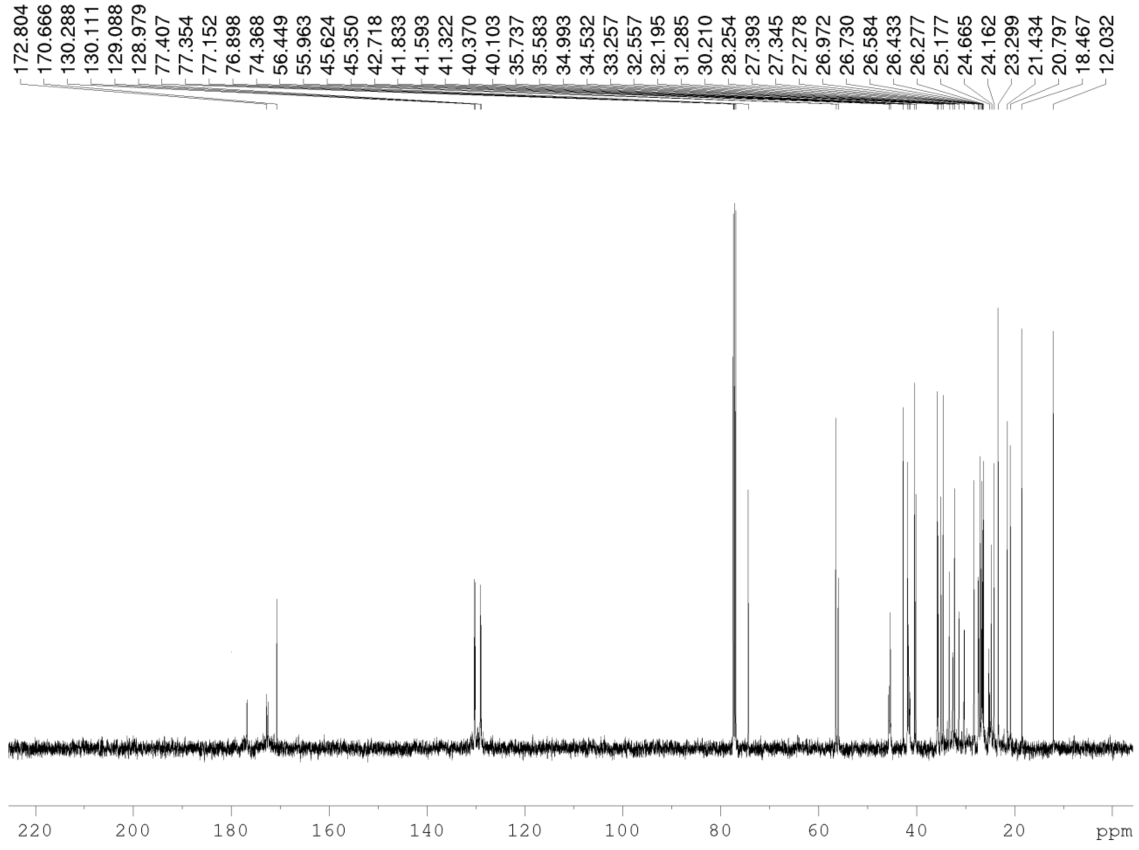

Figure S80. ^{1}H NMR Spectrum of compound **23d** (500 MHz, $CDCl_3$)

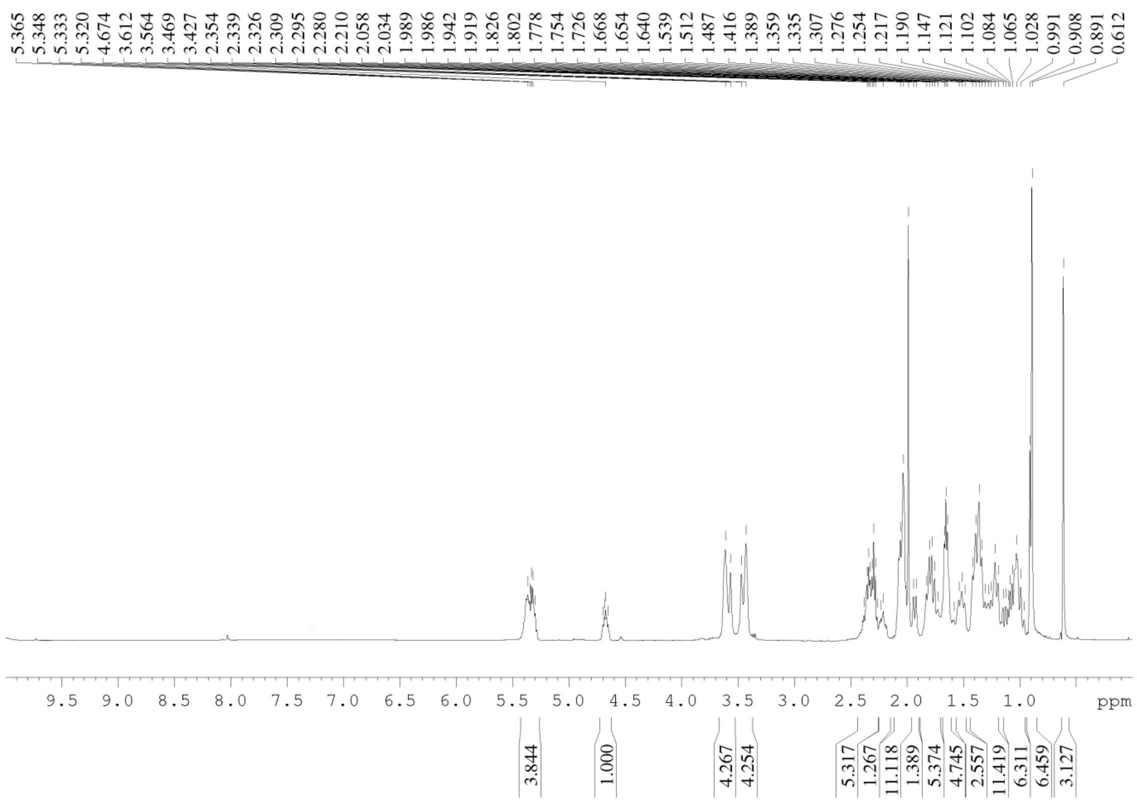

Figure S81. ^{13}C NMR Spectrum of compound **24a** (125 MHz, $CDCl_3$)

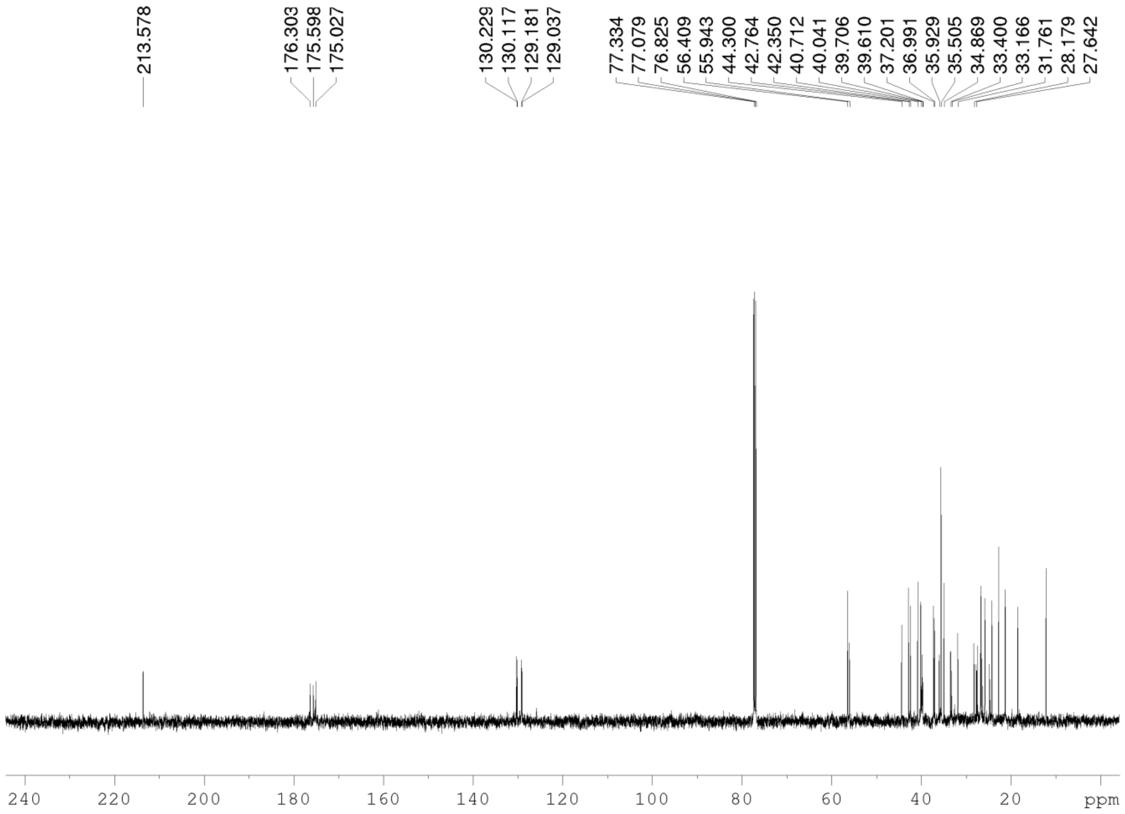

Figure S82. ^{1}H NMR Spectrum of compound **24a** (500 MHz, $CDCl_3$)

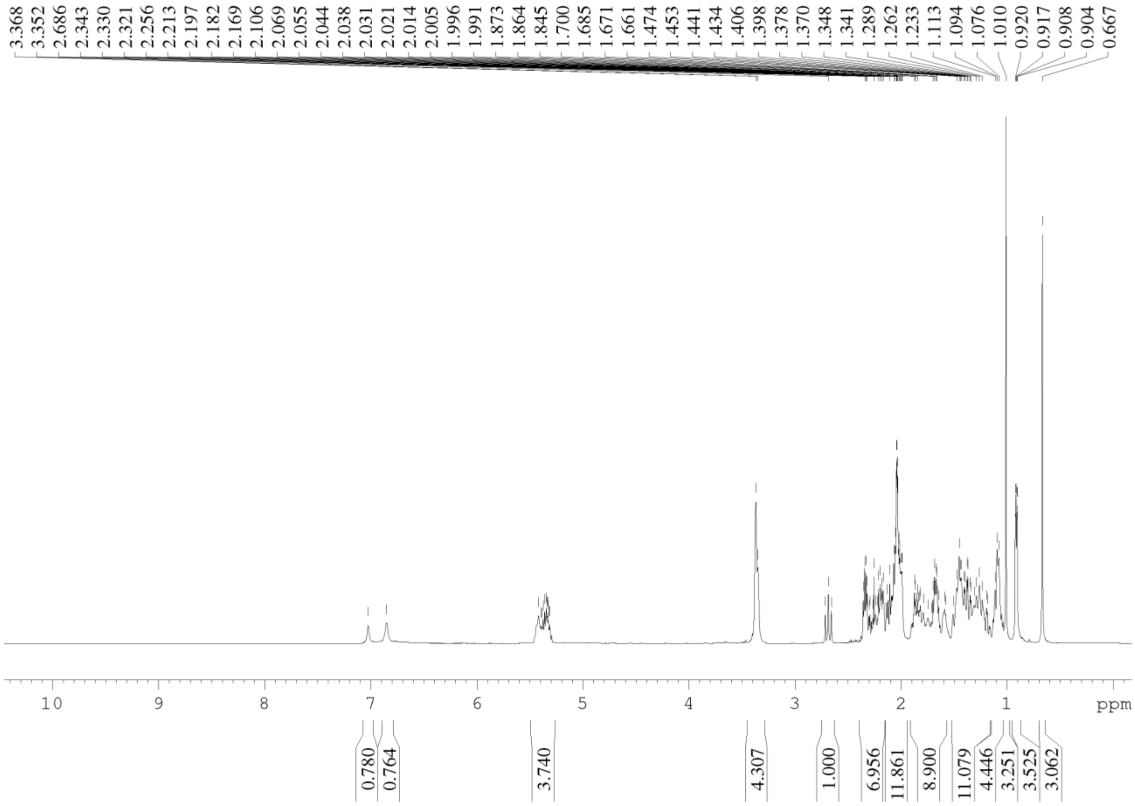

Figure S83. ^{13}C NMR Spectrum of compound **24b** (125 MHz, $CDCl_3$)

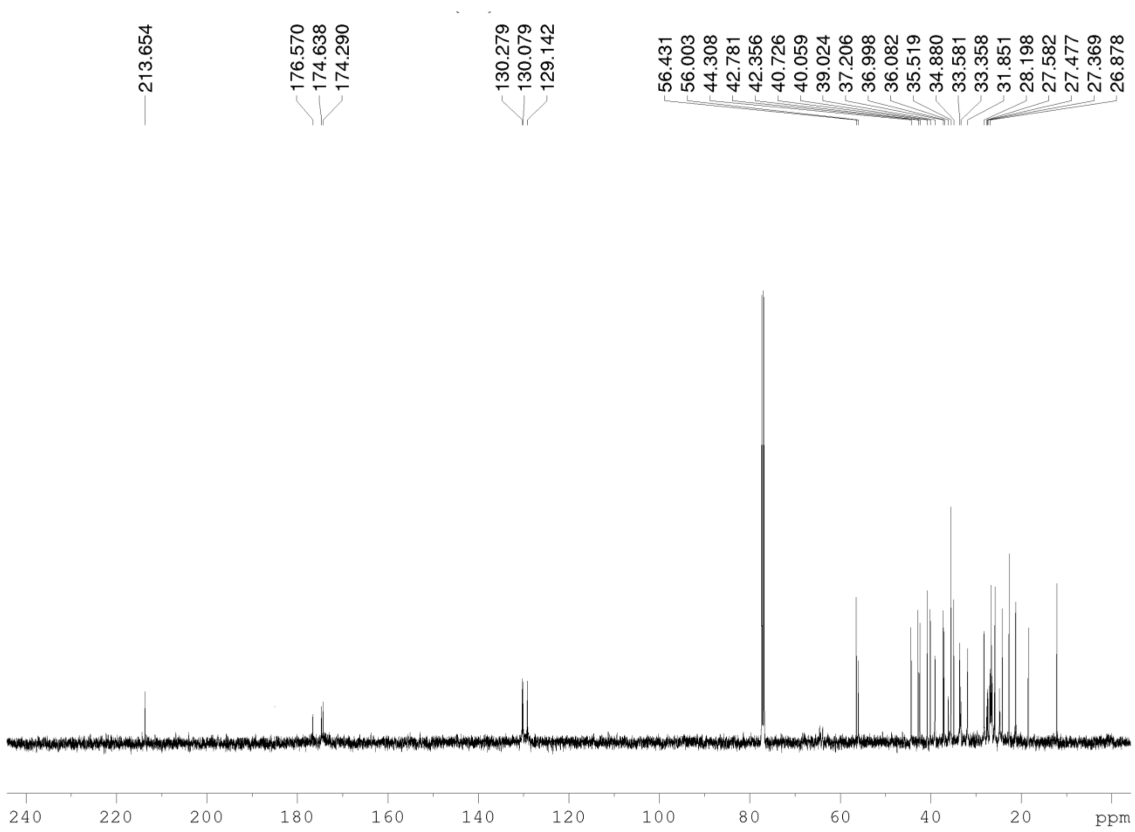

Figure S84. ^{1}H NMR Spectrum of compound **24b** (500 MHz, $CDCl_3$)

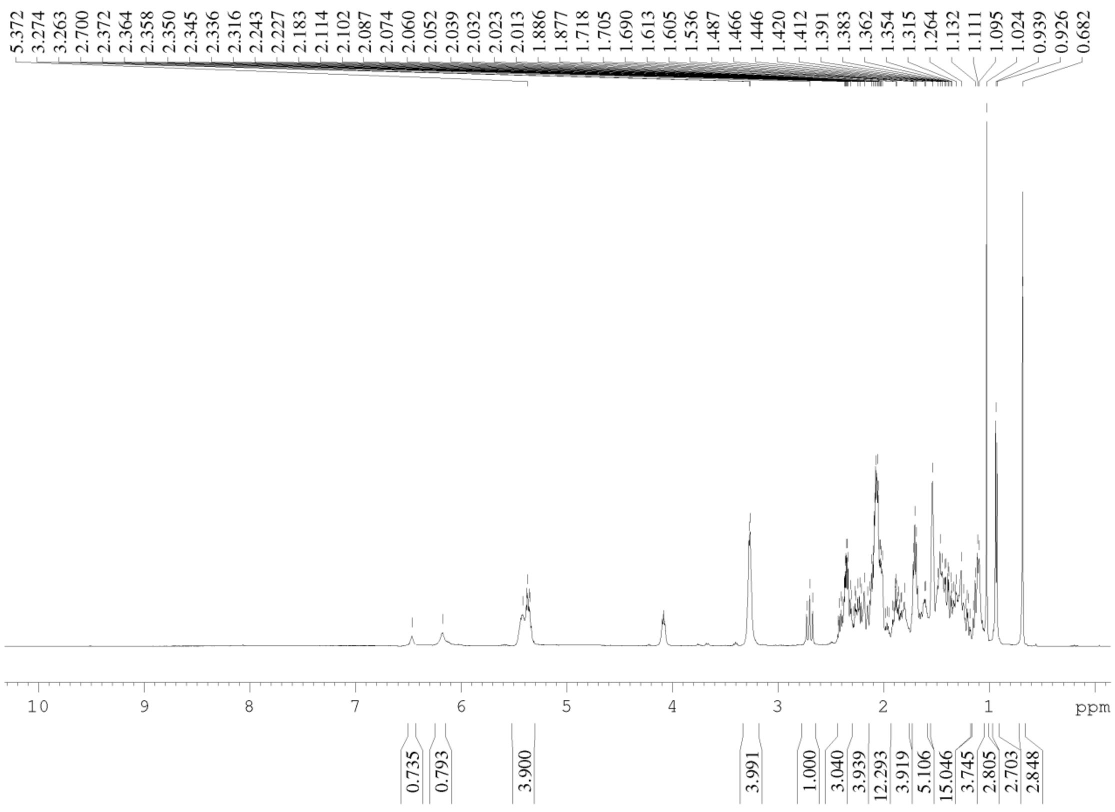

Figure S85. ^{13}C NMR Spectrum of compound **24c** (125 MHz, $CDCl_3$)

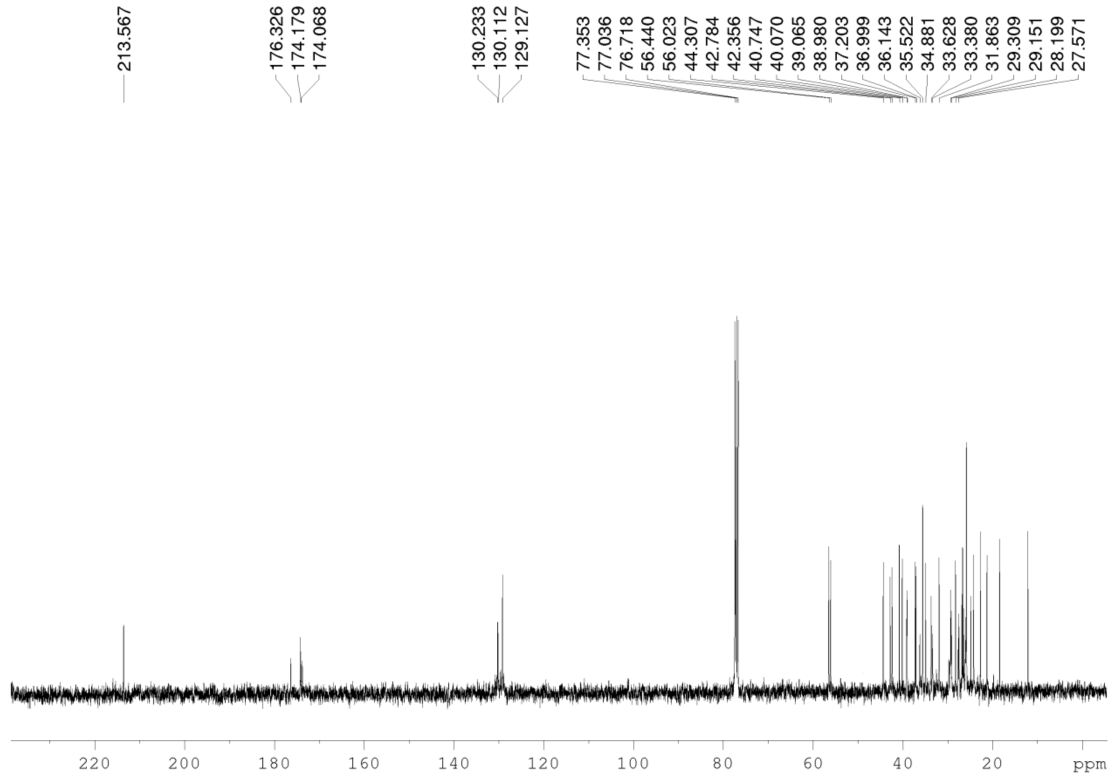

Figure S86. ^{1}H NMR Spectrum of compound **24c** (500 MHz, $CDCl_3$)

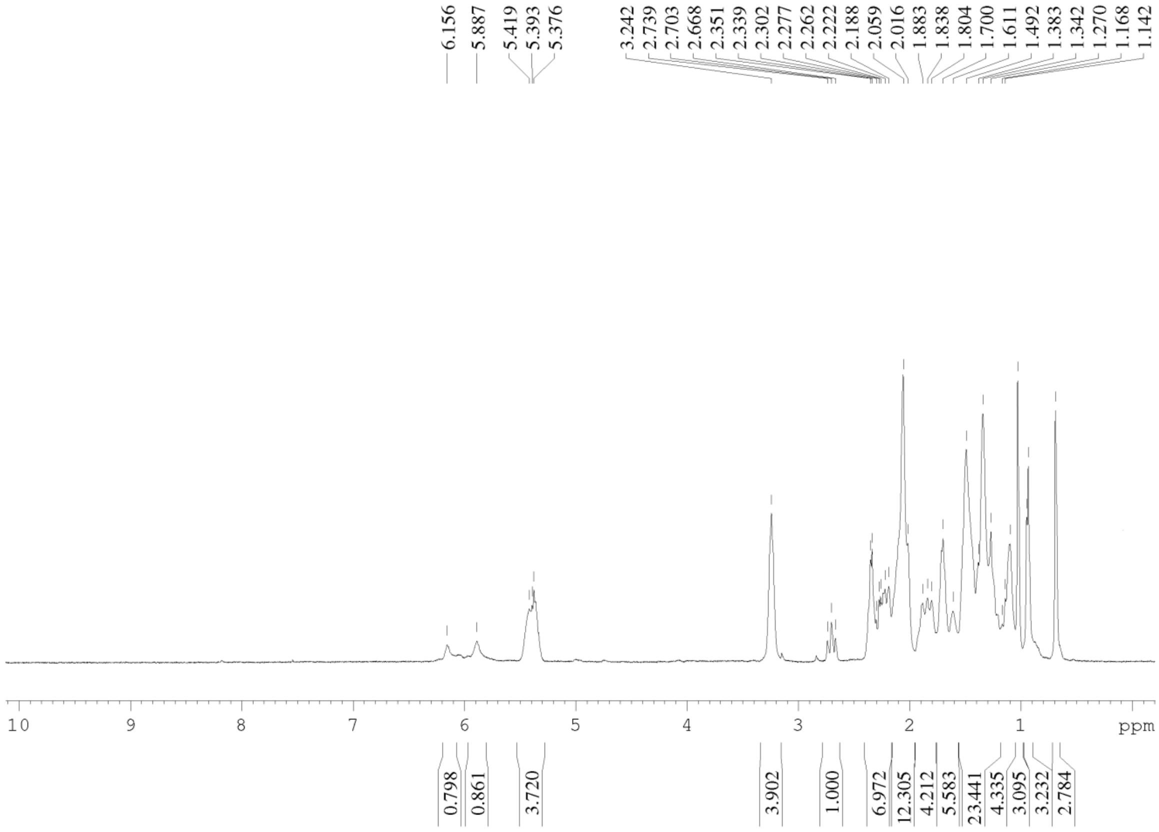

Figure S87. ^{13}C NMR Spectrum of compound **24d** (125 MHz, $CDCl_3$)

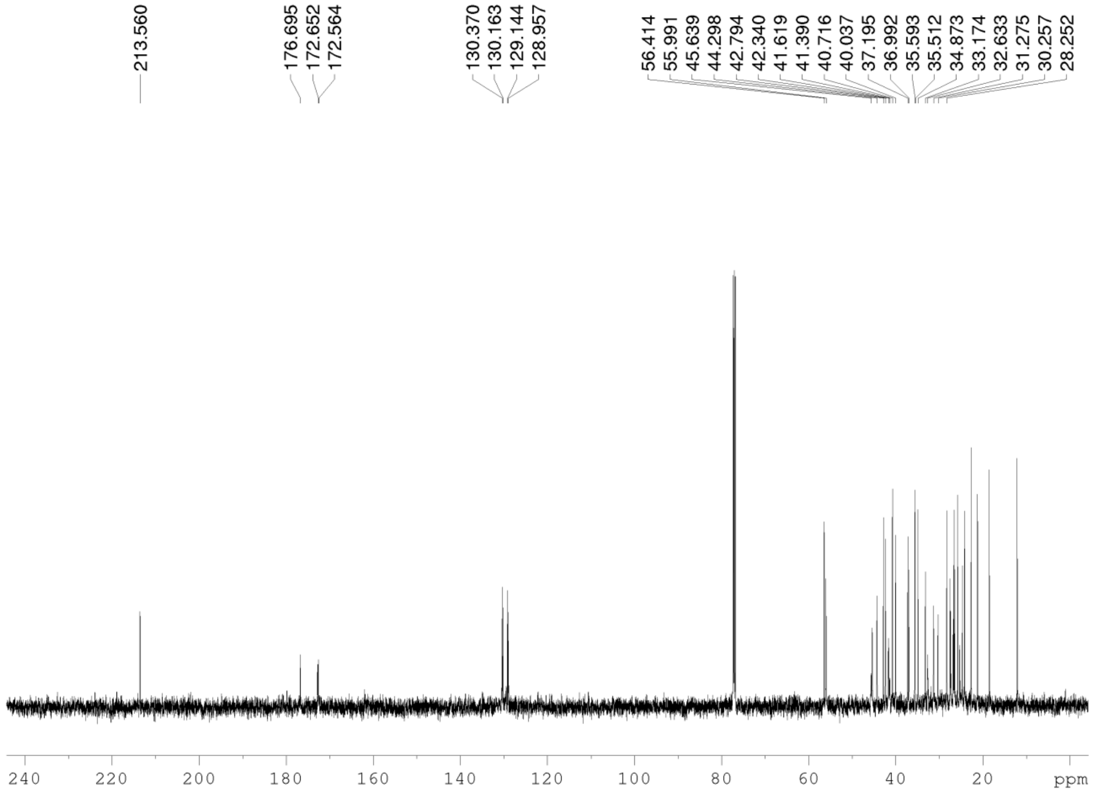

Figure S88. ^{1}H NMR Spectrum of compound **24d** (500 MHz, $CDCl_3$)

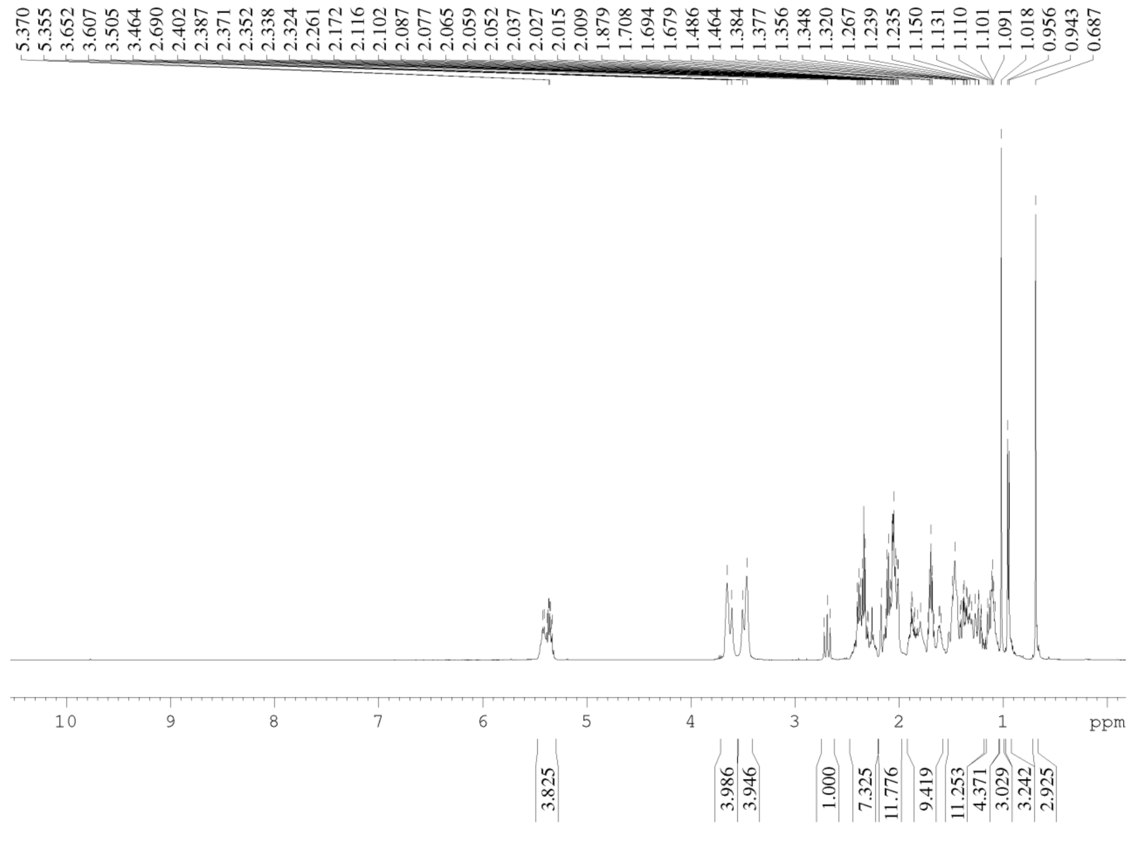

Supplement: Supplementary file 1 [file pharmaceuticals-14-00084-s001.pdf]
